# Inferring Gene Regulatory Networks from a Population of Yeast Segregants

**DOI:** 10.1038/s41598-018-37667-4

**Published:** 2019-02-04

**Authors:** Chen Chen, Dabao Zhang, Tony R. Hazbun, Min Zhang

**Affiliations:** 1 Department of Statistics, Purdue University, West Lafayette, IN 47907 USA; 2 Department of Medicinal Chemistry and Molecular Pharmacology, Purdue University, West Lafayette, IN 47907 USA; 3 Purdue University Center for Cancer Research, Purdue University, West Lafayette, IN 47907 USA

## Abstract

Constructing gene regulatory networks is crucial to unraveling the genetic architecture of complex traits and to understanding the mechanisms of diseases. On the basis of gene expression and single nucleotide polymorphism data in the yeast, *Saccharomyces cerevisiae*, we constructed gene regulatory networks using a two-stage penalized least squares method. A large system of structural equations via optimal prediction of a set of surrogate variables was established at the first stage, followed by consistent selection of regulatory effects at the second stage. Using this approach, we identified subnetworks that were enriched in gene ontology categories, revealing directional regulatory mechanisms controlling these biological pathways. Our mapping and analysis of expression-based quantitative trait loci uncovered a known alteration of gene expression within a biological pathway that results in regulatory effects on companion pathway genes in the phosphocholine network. In addition, we identify nodes in these gene ontology-enriched subnetworks that are coordinately controlled by transcription factors driven by trans-acting expression quantitative trait loci. Altogether, the integration of documented transcription factor regulatory associations with subnetworks defined by a system of structural equations using quantitative trait loci data is an effective means to delineate the transcriptional control of biological pathways.

## Introduction

Gene expression is a fundamental step in the flow of information from an organism’s genotype to phenotype. The genetic information encoded in an organism’s DNA is transferred into a functional gene product (e.g., protein) via the process of gene expression, and gene expression leads to the formation of the organism’s phenotype. Gene expression have been found to be associated with a broad range of complex traits and diseases^1^, and thus play an important role in determining an organism’s development. Numerous efforts have been made to map phenotypes to gene expression in order to dissect their genetic basis.

Genes rarely act in isolation; instead, they interact with each other and make up gene regulatory networks to function as a whole^2^. The study of this mechanism is crucial for understanding the properties and functions of genes, which help reveal the genetic architecture of complex traits and diseases. Although genetic experiments can be conducted to discover interactions among genes, this approach can be costly and time consuming. Alternatively, measurements of gene expression levels reveal gene expression patterns in a specific condition and can be exploited to infer gene regulatory networks. Various approaches have been proposed to infer gene regulatory networks using gene expression data, such as relevance networks^3–7^, Bayesian networks^8–11^, Gaussian graphical models^12–15^, and many others.

Recent advances in sequencing technologies make it feasible to obtain both whole-genome genotype and gene expression for each individual, i.e., genetical genomics data^16^. Combining genetics with gene expression reveals additional information on genetic structure and holds great promise for improving the accuracy of gene regulatory network inference. Numerous genetical genomics experiments, such as the Genotype-Tissue Expression (GTEx) project^17^, have been conducted to collect genetical genomics data.

Much effort has been devoted to using genetical genomics data for genome-wide association (GWA) analysis of gene expression, i.e., expression quantitative trait loci (eQTL) mapping^18^. Mapping of eQTL intends to elucidate variation of expression traits attributed to genomic variation, and to identify chromosomal loci (i.e., eQTL) of genetic polymorphisms associated to the expression of a gene under investigation. An eQTL located within the region of the gene under investigation is called a cis-eQTL, otherwise it is called a trans-eQTL. While the cis effects of a gene represent direct regulations, indirect regulations of trans-eQTL are likely caused by interactions among genes. These eQTL provide insight on the functional sequences of the gene expression, and thus an indirect interrogation of the functional landscape of gene regulations^19^.

Gene regulatory networks can be characterized using a system of structural equations^20^, with each equation describing the causal effects of cis-eQTL and the regulatory effects of other genes on a given gene. Such a framework makes it feasible to take a genome-wide survey and to directly reveal interactions among genes. Application of structural equations in genetical genomics studies have been previously demonstrated^21–24^. Two studies are applicable to constructing gene regulatory networks for a small number of genes^21,22^. However, genetical genomics experiments usually collect whole-genome gene expressions for a very limited number of samples, therefore the number of genes is much larger than the sample size. For such consideration, another study^23^ proposed to apply the adaptive lasso^25^ to construct a sparse gene regulatory network. An additional approach instead proposed to maximize a penalized likelihood for constructing a sparse gene regulatory network^24^.

Here we construct gene regulatory networks in yeast via building up a large system of structural equations with the two-stage penalized least squares (2SPLS) method^26^. We applied the 2SPSLS method to an eQTL dataset derived from a cross between a wild yeast vineyard strain and a laboratory strain^27^. Fitting one linear model for each gene at each stage, the 2SPLS method develops optimal prediction of a set of conditional expectations at the first stage, and consistent selection of regulatory effects from massive candidates at the second stage. It is computationally fast and allows for parallel implementation, outperforming the adaptive lasso based algorithm^23^, and the sparsity-aware maximum likelihood algorithm^24^, in terms of both accuracy and speed, for identifying regulatory effects in different network structures. This parallel implementation makes it feasible to evaluate the significance of regulatory effects via the bootstrap method. Using this approach we identified subnetworks that were enriched in gene ontology categories suggesting an extrinsic regulatory mechanism controlling these biological networks. Our eQTL predictions uncovered a known alteration of gene expression within a biological pathway that results in regulatory effects on companion pathway genes in the phosphocholine network. In addition, we delineate how nodes in these subnetworks are coordinately controlled by a transcription factor driven by trans-acting eQTL. For example, we detail how a proteasomal subnetwork is controlled by the *RPN4* transcription factor, via a trans-acting eQTL, resulting in the coordinated expression of genes in this subnetwork.

## Results and Discussion

### Identified cis-eQTL

To investigate and demonstrate the utility of cis-eQTL to infer regulatory interactions among genes, we performed a genome-wide survey of the budding yeast, *Saccharomyces cerevisiae*. We used a well-established dataset that involved a cross between a laboratory strain (BY4716) and a wild yeast strain (RM11-1A) isolated from a California vineyard. At a significance level of 0.05, we identified 409 genes (out of a total of 5,727 genes), with significant cis-eQTL (Table S1 has each p-value listed). The set of cis-eQTL for each gene was filtered to control the pairwise correlation under 0.90, and then was further filtered to keep a maximum of three cis-eQTL that have the strongest association with the corresponding gene expression. Detailed results are provided in Supplementary Information (Table S1).

### Constructed gene regulatory networks

The constructed network includes a total of 409 nodes and 5,068 edges respectively (Table S2). Among 260 edges repeatedly identified in more than 80% of the 10,000 bootstrap data sets, 258 edges, including 226 positive and 32 negative regulations, were in the 5,068 edges constructed from the original data set. The edges formed a number of subnetworks, among which 12 identified subnetworks have more than 5 genes (Table S3). We examined the 12 subnetworks for gene set enrichment using DAVID and found enrichments in gene ontology categories within each subnetwork (Table S4).

**Figure 1:**
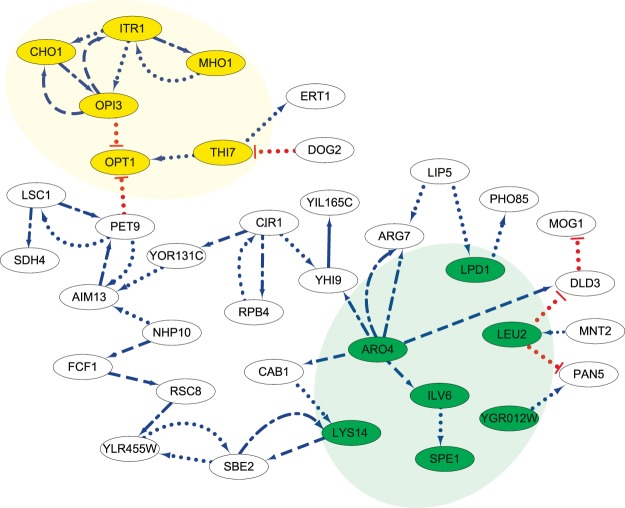
The largest gene regulatory subnetworks in yeast. While the dotted, dash-dotted, dashed, and solid lines implied the corresponding connections were constructed respectively in [80%, 90%), [90%, 95%), [95%, 100%), and 100% of the bootstrapping data sets, the blue arrow- and red bar-headed lines indicate up and down regulations, respectively. Highlighted in yellow is the Inositol subnetwork in which several genes involved in the CDP-DAG/phosphocholine pathway are coordinately repressed by exogenous inositol. Within the amine biosynthetic process subnetwork highlighted in green, *LEU2, LPD1, YGR012W, LYS14, ILV6*, and *ARO4* are involved in multiple biosynthetic processes (as shown in Table S5).

Figure 1 shows the largest subnetwork formed by these 260 edges, other constructed subnetworks are listed in Supplementary Information (Table S3). This large subnetwork (subnetwork 1) was subjected to YeastMine analysis to identify gene ontology enrichments and pathways^28^. This analysis revealed that 17 genes in subnetwork 1 are involved in a variety of biosynthetic pathways (p-value = 4.17E-07) and synthesis of secondary metabolites (Table S5). Many genes within this subnetwork are involved in amino acid synthesis and we also observed a subset of connected genes that were closely associated with phosphocholine metabolism. The enrichment in gene ontology terms for the subnetworks demonstrated that using the 2SPLS method of constructing regulatory cis-eQTL results in identification of clusters of genes with common biological function. The closely connected nodes with genes of common function suggest that genetic polymorphisms commonly result in compensating regulatory events of companion genes.

### Comparison to existing databases (STRING and BioGRID)

To investigate the constructed gene regulations with involvement of downstream protein-protein interactions, we compared the subnetworks to the known and predicted protein-protein interactions in the STRING database (http://string-db.org/)^29^. Developed by a consortium of institutions, the current version of STRING collects information of 9,643,763 proteins from 2,031 organisms. The comparison demonstrated common and enriched processes that parallel the gene ontology enrichments detected via DAVID analysis. For example, subnetwork 6 yielded a highly connected set of nodes that involved proteasome subunits and associated proteins reflecting the molecular architecture of the proteasome complex and this subnetwork is further analyzed in this report. Analysis of Subnetwork 1 with STRING database also revealed that *CHO1*, *ITR1* and *OPI3* are interconnected identically to the phosphocholine network discussed in the following section (highlighted in yellow of Fig. 1). Similar results were obtained when comparing to BioGRID using the YeastMine tool (Table S5)^28,30^. These striking examples of similar network organization observed in STRING with our predictions validated our approach and prompted the examination and integration of these subnetworks with the literature and other functional genomics database information such as mRNA profiling.

### The Phosphocholine subnetwork

**Figure 2:**
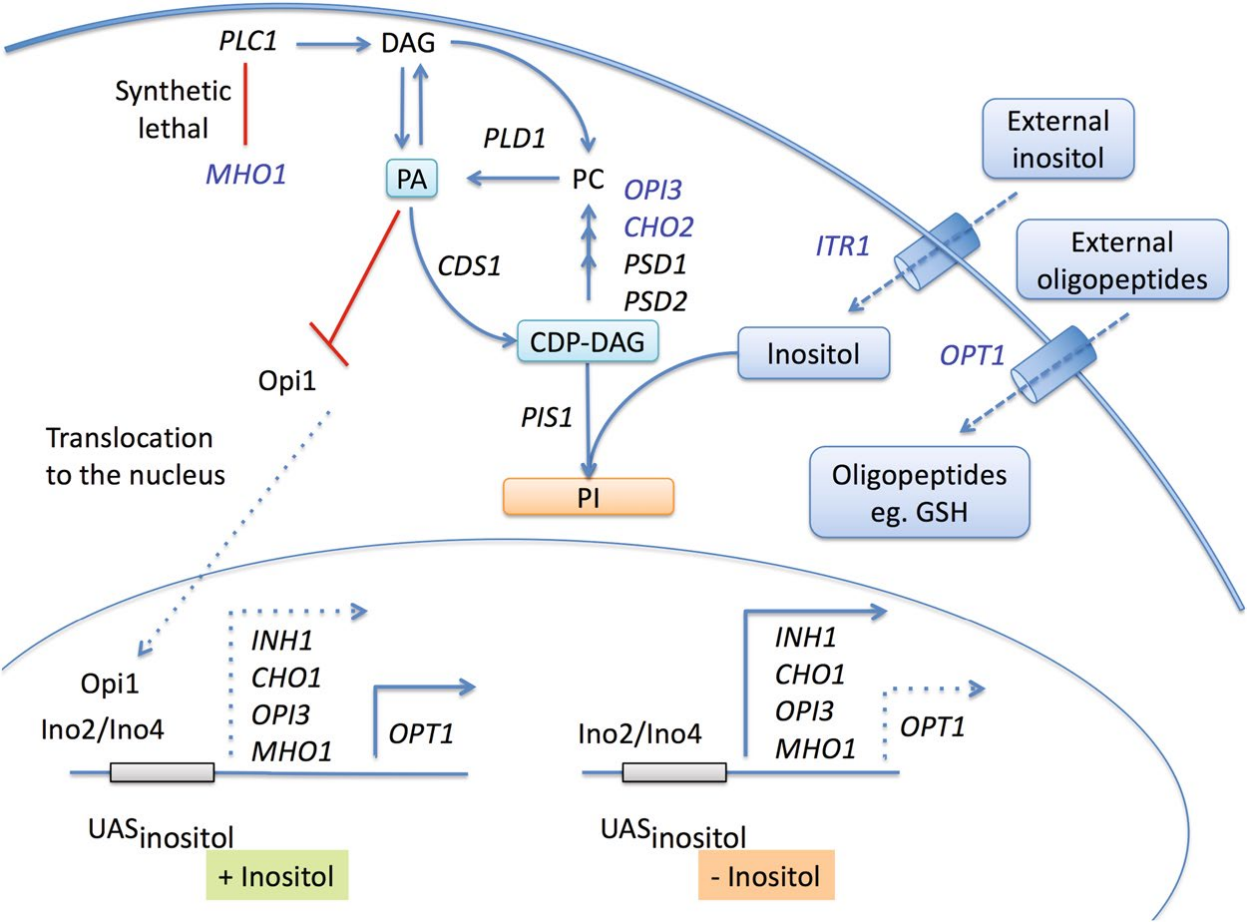
The pertinent features of the phosphocholine pathways. The CDP-DAG phosphocholine pathway shows the involvement of genes implicated in the eQTL–based phosphocholine subnetwork (Blue font) - *CHO1*, *OPI3* and *ITR1* (transport of external inositol). PA inhibits the Opi1 repressor translocation to the nucleus. Low levels of PA result in translocation of Opi1 to the nucleus and the association and repression of the Ino2/Ino4 heterodimeric transcription factor. Low levels of inositol result in activation of transcription of several phosphocholine pathway genes and *MHO1* and repression of *OPT1*.

All of the genes in the phosphocholine subnetwork (highlighted in yellow of Fig. 1), except for *OPT1*, have similar patterns of regulation and are repressed by the presence of inositol or choline in yeast growth medium. The majority of the genes (*MHO1*, *ITR1*, *CHO1* and *OPI3*) are involved in lipid metabolism and are subject to transcriptional regulation by the Opi1 repressor^31^. Strikingly, two of these genes are in a linear metabolic pathway converting cytidine diphosphate diacylglycerol (CDP-DAG) to phosphocholine (*CHO1* and *OPI3*) (Fig. 2)^32^. *ITR1* encodes a transporter that imports exogenous inositol from the growth media. The function of *MHO1* is unclear, but the gene has been shown to be synthetic lethal with *PLC1*, an enzyme involved in the production DAG and inositol trisphosphate (IP3)^33^. The eQTL-based prediction of reciprocal positive regulation between genes within the DAG-phosphocholine pathway indicates a regulatory interdependence of these genes (*MHO1*, *ITR1*, *CHO1* and *OPI3*). Interestingly, these genes are coordinately controlled by the Ino2-Ino4 transcription factor complex via the inositol sensitive upstream activating element (UAS-INO) but additional regulation may be exerted based on mRNA abundance level of pathway components. For example, *CHO1* mRNA stability increased in response to respiratory deficiencies leading to increased phosphatidylserine levels and activities of other CDP-DAG pathway enzymes^34^. The regulatory mechanisms involved for phospholipid synthesis are complex and include biochemical regulation by several phospholipid precursors and products including phosphatidic acid (PA) and CDP-DAG^35^. PA helps to sequester the Opi1 repressor away from the nucleus^36^ and elevated levels of CDP-DAG favors the Opi1-mediated repression of genes under control of the UAS-INO element^35^, shown in Fig. 2.

**Figure 3:**
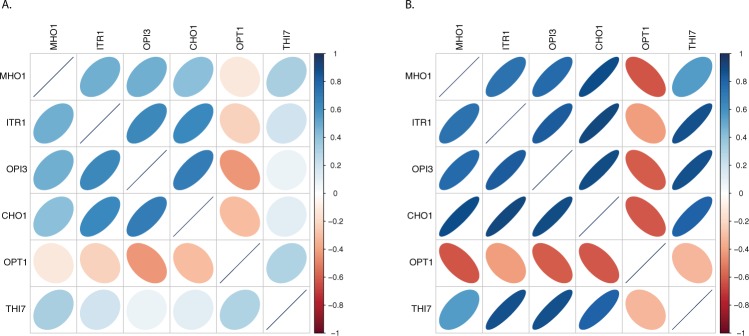
Correlation of expression for genes in the phosphocholine network. (**A**) Pairwise correlation plot between the 6 genes in the phosphocholine subnetwork for the eQTL expression data from parental strain replicates^27^. (**B**) Pairwise correlation plot between the 6 genes involved in phosphocholine subnetwork for independent expression datasets from SPELL^39^. The color indicates the direction of the correlations (blue indicates positive and red indicates negative) and the shape represents the strength of correlation.

In addition, inositol-based regulation has been observed to control various metabolic pathways involved in membrane biogenesis including the activation of *OPT1*, an oligopeptide and glutathione transporter encoding gene^31^. The prediction that *OPI3* negatively regulates *OPT1* expression is consistent with the opposite effects of inositol on these two genes. An examination of the expression pattern of *OPT1* and *OPI3* shows the strong anti-correlated expression pattern between these genes (Fig. 3A). The inferred gene-gene relationships for this phosphocholine subnetwork demonstrate the utility of our eQTL analysis to delineate biologically relevant pathways. In addition, our analysis implicated that a poorly characterized gene, *MHO1*, may have a functional role in the phosphocholine pathway.

Examination of the sequence of the RM and BY parental strains for the genes in the phosphocholine subnetwork revealed a lack of nonsynonymous polymorphisms within the *OPI3* gene and the presence of four single nucleotide polymorphisms (SNPs) in the upstream promoter region (500 bp from the ATG). The identical amino acid sequence of Opi3 present in the RM and BY strains suggests that the differences between strains is due to expression level of the protein but not due to any differences in protein stability or activity. One of the SNPs was located at the -1 position upstream to the start codon, which is a position demonstrated to affect gene expression level. The adenine nucleotide in the BY strain favors a higher expression level compared to guanine for the RM parent based on large scale analysis of variant nucleotides at the -3 to -1 position relative to the start codon^37^. This is reflected in the overall expression levels observed for mRNA levels in the eQTL expression data set from Serial Pattern of Expression Levels Locator (SPELL) database^38^: ~1.5 fold lower expression for 12 RM parent values compared to a BY reference pool (see Tables 1 and S4). The *CHO1* gene exhibited an expression difference of 1.2 fold or lower between the RM and BY parents. Genes with similar mRNA levels between the parent strains do not harbor SNPs that are driving the expression differences evident in the segregant progeny strains suggesting the presence of trans-acting SNPs as discussed in the proteasome subnetwork section below. In addition to SNPs in the promoter region, the other genes in the network exhibited nonsynonymous polymorphisms using the Variant Viewer analysis tool^39^, as shown in Table 1.

**Table 1:** Summary of SNPs and gene expression difference between RM and BY strains for genes in the phosphocholine network.

Gene	Nonsynonymous SNPS	SNPS in Promoter REGION^*a*^	RM/BY Fold Change	P-Value^*b*^
*CHO1*	A9T; L234F	6 (−78; −79; −213; −228; −375; −451)	1.24*	0.02
*ITR1*	C521F	2 (−211; −286)	0.98	ns
*MHO1*	A331T; F164I	4 (−141; −169; −224; −285)	1.11**	0.002
*OPI3*	None	4 (−1; −389; −395; −450)	1.51**	0.008
*OPT1*	A200V; V439I	4 (−108; −142; −143; −333)	0.98	ns

^*a*^The total number of SNPs in the promotor region within 500 bp upstream of the gene start.

^*b*^P-value calculated by comparing 12 RM parent strains to 6 BY parent strains (ns = not significant).

### Validation of expression patterns using independent datasets

From the SPELL database, we input all 6 genes from the phosphocholine subnetwork to identify expression profiling experiments that had correlated data for the query genes. This approach resulted in 7 datasets with relevance weighting larger than 1.0% compared to all other experimental datasets. Among these, several datasets had missing data or very low levels of expression for the 6 genes of interest with the exception of 3 datasets, which were subjected to further analysis. We calculated the pairwise correlation between these 6 genes and visualized the correlation matrix using the R package “corrplot” (https://cran.r-project.org/web/packages/corrplot/index.html) for one of these data sets that focused on hypo-osmotic shock^40^. The pairwise correlation plot^41^ is presented in Fig. 3B. This independent expression data set demonstrated the strong anti-correlation between *OPT1* and the other genes within the phosphocholine subnetwork, which is consistent with the prediction of negative regulation of *OPT1* by *OPI3*. Other genes in the network demonstrated similar correlation plots to the eQTL data from parental replicates with the exception of the *THI7*-*OPT1* pair, which appears to be regulated differently in hypo-osmotic conditions. The *THI7* gene encodes a transporter that facilitates the uptake of thiamine and is upregulated in the hypo-osmotic experiment whereas it is down-regulated in the RM strain compared to the BY parent strain. The regulatory relationship between *THI7*-*OPT1* pair appears complex and is altered depending on environmental conditions and stress.

### The Proteasome subnetwork

**Figure 4:**
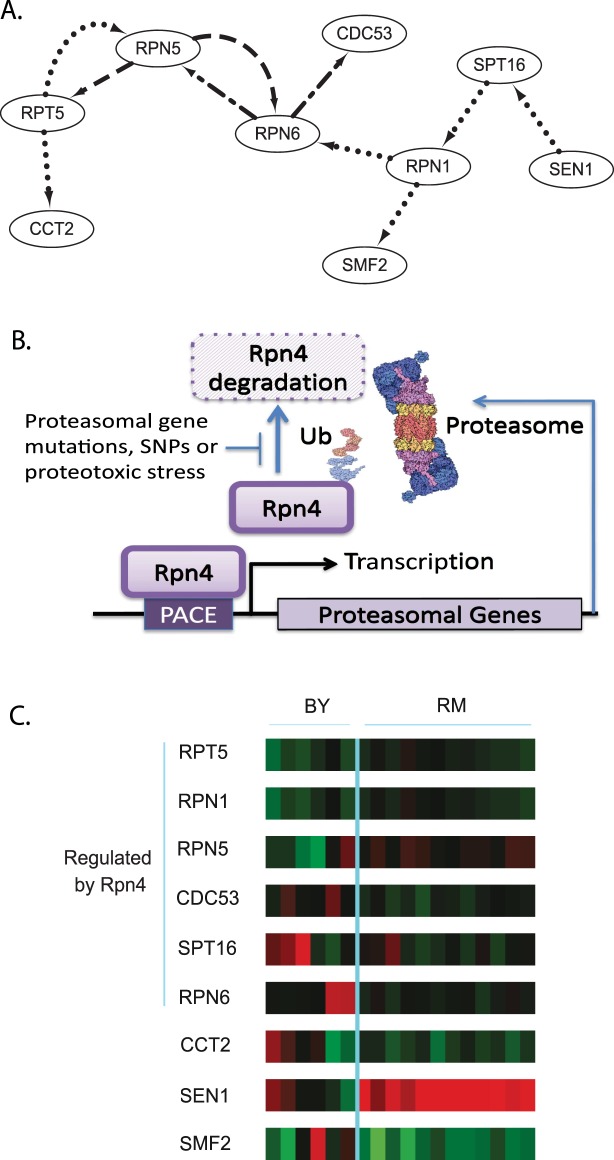
Proteasomal subnetwork is subject to feedback regulation. (**A**) Subnetwork 6 contains four proteasomal genes and other genes enriched for ubiquitin-dependent protein catabolic processes. (**B**) Feedback regulation model depicting the control of proteasomal gene transcription. The *RPN4* transcription factor binds to the promoter of proteasomal genes via the PACE DNA site and initiates proteasomal gene transcription. The *RPN4* transcription factor is modified by ubiquitin (Ub) and degraded by the proteasome. Mutations to proteasomal genes, SNPs or proteotoxic activity result in the inhibition of *RPN4* degradation. (**C**) Heat map depicting the expression level of each strain (6 BY parent strains and 12 RM parent strains^27^) for genes in the proteasomal subnetwork. Six genes within the network have evidence of regulation by *RPN4*. The *RPN4*-regulated genes do not exhibit any difference between BY and RM parent strains suggesting that trans-acting eQTL are impacting expression in segregant strains. Note other genes in the network do demonstrate different expression levels between the parent strains.

Analysis of the genes in subnetwork 6 indicated enrichment in ubiquitin-dependent protein catabolic processes (p-value = 1.25E-04 which is adjusted to 0.014 by applying the Bonferroni method), shown in Table S4. This subnetwork included 4 genes that encode proteasomal subunits. The network structure indicated extensive reciprocal regulation between proteasomal genes (Fig. 4A). The proteasome has key roles in cellular homeostasis and is subject to multiple regulatory mechanisms^42^. This reciprocal regulation predicted by our eQTL analysis is consistent with a proposed feedback circuit in which the *RPN4* transcription factor upregulates proteasomal genes but is also degraded by the proteasome. A similar feedback mechanism exists in higher eukaryotes because deletion of the regulatory S5a/Rpn10/p54 subunit results in extreme and coordinate upregulation of other proteasomal genes^43^. Additional studies with RNA interference in *Drosophila* indicate that knockdown of gene expression of a proteasomal subunit results in upregulation of the companion subunit mRNAs^44,45^. A mechanism underlying mRNA upregulation in higher eukaryotes appears to be dependent upon the 5′ untranslated mRNA region^46^. These and other studies have culminated in a model where factors such as proteotoxic stress, proteasome inhibitors and proteasomal gene mutations have been documented to upregulate proteasome levels via *RPN4* -mediated transcription. *RPN4* is a transcription factor that specifically binds to the *P*roteasome *A*ssociated *C*ontrol *E*lement (PACE) found in most proteasome genes^47,48^ resulting in coordinate regulation of many proteasome genes (Fig. 4B). The positive regulation predictions between proteasome genes outlined in subnetwork 6 (Fig. 4A) may reflect this coordinate regulation. The RM and BY parent strain gene expression data, 6 BY parent strains and 12 RM parent strains, indicated similar expression levels^27^ between the proteasomal genes (Fig. 4C) suggesting that trans-acting polymorphisms are driving the expression differences evident in the segregant progeny strains. The other three genes in this network (*CCT2*, *SEN1* and *SMF1*) have differing expression levels between RM and BY parent strains. The prevalence of trans-acting eQTL has been documented and previously reported for this dataset between 22–48%^49^. The regulatory events observed in subnetwork 6 maybe controlled by *RPN4* because six nodes (*RPN6*, *CDC53*, *RPN5*, *SPT16*, *RPN1* and *RPT5*) have documented regulations by *RPN4* based on the YEASTRACT database^50^, shown in Table S7. The edges in this network may reflect the timing of expression driven by *RPN4* and not the direct regulation of one proteasomal gene by another proteasomal gene. Further examination of all the subnetworks using the YEASTRACT database shows several networks that are controlled by one or more transcription factors (Table S7). In total, this proteasome subnetwork example demonstrates that interpretation of eQTL regulatory information must be integrated with heterologous information such as transcription factor activity. This integrated approach recapitulates the biological networks controlled by transcription factors.

## Conclusions

In this work, we constructed gene regulatory networks in yeast via establishing a large system of structural equations. By integrating genomic information into gene regulatory network construction, we identified subnetworks that were enriched in gene ontology categories revealing regulatory mechanisms controlling these biological pathways. Our eQTL predictions uncovered a known alteration of gene expression within a biological pathway that results in regulatory effects on companion pathway genes in the phosphocholine network. In addition, we delineate how nodes in these subnetworks are coordinately controlled by a transcription factor driven by trans-acting eQTL. Hence, directionality of the edges in the subnetworks may reflect the timing of transcription control of these related genes. We expect that it is possible to build regulatory networks with increased size and accuracy with more extensive datasets of eQTL. For example, several studies have used additional quantitative traits, multi-parent crosses and also integrated other phenotypic markers such as metabolite levels in probing yeast biological networks^51–54^. This study demonstrates that 2SPLS analysis provides insight on understanding regulatory relationships among genes, which reveal the genetic architecture of complex traits and diseases.

## Materials and Methods

### eQTL analysis

We analyzed a yeast data set with 112 segregants from a cross between two strains BY4716 and RM11-la^27^. The study measured mRNA expression combined with genotyping data (2,956 SNPs) from the 112 haploid segregant progeny from the BY4716 and RM11-la cross. The data were obtained from the Gene Expression Omnibus^55^ (GEO; http://www.ncbi.nlm.nih.gov/projects/geo/) with a GEO accession number of GSE1990. A total of 5,727 genes were measured for their expression values, and detailed procedure of normalization was previously described^27^. Briefly, base 2 logarithm transformation of the gene expression ratio (sample/BY4716 reference) was calculated and averaged over duplicated samples. The data were then normalized using MAANOVA package^56^. As previously described^27^, the missing genotype information of the available 2,956 markers was imputed using sample mean prior to analysis. To identify eQTL for each gene, the expression of each gene was regressed against all markers in the gene and within 500 bp upstream of the genetic region, using a simple linear regression model.

### Network construction

Denoting the expression values of *p* genes as *Y* = (*Y*_1_, …, *Y*_*p*_) and the genotypic values of *q* polymorphisms as *X* = (*X*_1_, …, *X*_*q*_), we characterized the gene regulatory network using a system of structural equations,

1 $$Y=Y{\rm{\Gamma }}+X{\rm{\Psi }}+{\rm{{\rm E}}},$$

where the *p* × *p* matrix Γ has zero diagonal elements and contains gene regulatory effects, the *q* × *q* matrix Ψ contains causal genomic effects from cis-eQTL, and E is an *n* × *p* matrix of error terms. We assume that *X* and E are independent of each other, and each component of E is independently distributed as normal with zero mean while its rows are identically distributed.

With the expression levels of the 409 genes and the genotypes of the selected cis-eQTL for each of 112 segregants, we applied the 2SPLS method^26^ to establish the system (1) for constructing a gene regulatory network in yeast. Fitting a single regression model for each endogenous variable at each stage, 2SPLS employs the ridge regression at the first stage to obtain consistent estimation of a set of conditional expectations, and the adaptive lasso^25^ at the second stage to consistently identify regulatory effects among a huge number of candidates.

To evaluate the reliability of constructed gene regulations, we generated a total of 10,000 bootstrap data sets (each with 112 segregants) by randomly sampling the original data with replacement, and applied 2SPLS to each data set to infer the gene regulatory network.

### SPELL - *S. cerevisiae*

To validate the results using independent datasets, we searched the SPELL database (http://spell.yeastgenome.org/)^38^. The phosphocholine subnetwork genes were entered into SPELL and experimental datasets were identified that had expression data for all genes and were highly ranked with relevance weighting larger than 1.0%. Using this approach, we identified three datasets for analysis and demonstrated independent validation of the predicted phosphocholine subnetwork structure.

### Identification of controlling transcription factors

A curated database of yeast transcription factors was used to identify transcription factors that are associated with regulating genes within subnetworks. The Yeast Search for Transcriptional Regulators And Consensus Tracking (YEASTRACT) database includes over 163,000 regulatory associations curated from the literature^50^. Genes within each subnetwork were used as the input gene list to search for transcription factors that are documented or potentially regulate gene within the list. Genes were considered to have a regulatory association with the transcription factor if there was documented DNA binding evidence plus expression evidence. The transcription factors were ranked by percentage of genes regulated by the respective transcription factor and the output for each subnetwork was included in the Supporting Information.

## Supplementary information

Supplementary Information

Genotype Data

## Data Availability

While the gene expression information can be found at Gene Expression Omnibus database with accession no. GSE1990, the genotype data are provided in the Supplemental Material with permission from Leonid Kruglyak. The gene expression of 12 RM and 6 BY parent strains are collected from Serial Pattern of Expression Levels Locator (SPELL) database (http://spell.yeastgenome.org/)^38^. The gene expression from the hypo-osmotic shock experiment^39^ can be downloaded from https://spell.yeastgenome.org/search/dataset_details/1002.
