# Supplementary material for: Inferring Gene Regulatory Networks from a Population of Yeast Segregants
Source: Sci Rep. 2019 Feb 4;9:1197. doi: 10.1038/s41598-018-37667-4 (PMC6361976; doi:10.1038/s41598-018-37667-4)
Supplement: Supplementary file 1 — Supplementary Information [file 41598_2018_37667_MOESM1_ESM.pdf]

Supplementary Information

Inferring Gene Regulatory Networks from a Population of Yeast Segregants

Chen Chen[1], Dabao Zhang[1,3,*], Tony R. Hazbun[2,3,*] and Min Zhang[1,3,*]

[1]Department of Statistics, Purdue University, West Lafayette, IN 47907

[2]Department of Medicinal Chemistry and Molecular Pharmacology, Purdue University, West Lafayette, IN 47907

[3]Purdue University Center for Cancer Research, Purdue University, West Lafayette, IN 47907

[*]Correspondence and requests for materials should be addressed to Dabao Zhang (email: zhangdb@purdue.edu), Tony Hazbun (email: thazbun@purdue.edu) or Min Zhang (email: minzhang@purdue.edu)

Table S1: List of cis eQTLs identified for each gene.

Table S2: List of all pairwise connections and bootstrap frequency and corresponding coefficients.

Table S3: List of subnetwork nodes.

Table S4: Gene ontology enrichment results using DAVID analysis of subnetworks.

Table S5: Results of YeastMine analysis of subnetwork 1.

Table S6: Gene expression values of RM and BY strains for genes in the phosphocholine network and statistical analysis.

Table S7: Identification of transcription factors controlling subnetworks using YEASTRACT.

Table S1: List of cis eQTLs identified for each gene.

ORF Name	Gene Name	Marker ID	p-Value
YCL018W	LEU2	6909_at_x05	2.00E-72
YLR155C	ASP3-1	9825_s_at_x15	1.79E-70
YEL021W	URA3	5724_at_x04	5.30E-62
YDL227C	Ho	6725_at_x14	1.69E-55
YDR038C	ENA5	6453_at_x14	1.42E-47
YBR132C	AGP2	7223_at_x06	1.60E-42
YAR028W	YAR028W	11290_at_x00	2.04E-37
YOL164W	BDS1	8717_at_x06	3.76E-34
YHR043C	DOG2	4472_s_at_x03	5.65E-32
YCR041W	YCR041W	6829_at_x02	8.05E-30
YOL104C	NDJ1	8639_at_x04	1.86E-29
YGL169W	SUA5	5166_at_x10	5.85E-28
YGL201C	MCM6	5224_at_x14	6.73E-28
YOL089C	HAL9	8654_at_x07	3.82E-26
YAR027W	UIP3	11289_at_x14	2.06E-24
YGL053W	PRM8	5054_at_x12	9.96E-22
YNL035C	YNL035C	8906_at_x10	1.62E-21
YIL089W	YIL089W	4177_at_x12	8.74E-21
YDR441C	APT2	6048_at_x08	9.71E-21
YDR367W	KEI1	6109_at_x15	3.66E-20
YPL016W	SWI1	2565_s_at_x02	6.99E-20
YAL056W	GPB2	11392_at_x03	8.10E-20
YOR131C	YOR131C	8423_at_x05	7.10E-17
YOL113W	SKM1	8675_at_x14	7.59E-16
YLR052W	IES3	10261_at_x14	2.95E-15
YDR134C	YDR134C	6323_at_x15	2.99E-15
YJL217W	REE1	11255_at_x07	3.41E-15
YML016C	PPZ1	9648_at_x04	3.53E-15
YDL217C	TIM22	6691_at_x12	4.30E-15
YLR464W	YLR464W	9866_i_at_x02	4.11E-14
YLR330W	CHS5	9809_at_x14	7.49E-14
YBR069C	TAT1	6980_at_x13	1.19E-13
YOL043C	NTG2	8609_at_x00	4.33E-13
YIL015C-A	YIL015C-A	4110_at_x13	6.13E-13
YBR274W	CHK1	7092_at_x08	6.46E-13
YML079W	YML079W	9720_at_x13	1.48E-12
YNL329C	PEX6	9201_at_x04	2.16E-12
YOL094C	RFC4	8649_at_x15	2.54E-12
YOR051C	ETT1	8523_at_x04	3.90E-12
YMR152W	YIM1	9506_at_x13	6.47E-12
YBR067C	TIP1	7290_at_x01	8.55E-12
YGL193C	YGL193C	5186_at_x12	8.57E-12
YGL207W	SPT16	5218_at_x13	1.65E-11

YDL132W	CDC53	6596_at_x01	1.86E-11
YKR080W	MTD1	10505_at_x01	2.95E-11
YHR115C	DMA1	4408_at_x08	3.33E-11
YNL041C	COG6	8900_at_x08	4.94E-11
YFL056C	AAD6	5424_at_x00	5.49E-11
YBR166C	TYR1	7213_at_x01	9.40E-11
YPR022C	SDD4	7729_at_x12	2.30E-10
YIL166C	YIL166C	4285_at_x06	3.13E-10
YJL030W	MAD2	11037_at_x13	3.40E-10
YOL092W	YPQ1	8651_at_x00	3.56E-10
YJL171C	TOH1	11212_at_x03	6.40E-10
YJL154C	VPS35	11184_at_x10	8.75E-10
YGL208W	SIP2	5217_at_x03	9.46E-10
YLR342W	FKS1	2837_s_at_x01	2.48E-09
YIL046W	MET30	4047_at_x02	2.55E-09
YLR389C	STE23	9924_at_x07	2.85E-09
YDR339C	FCF1	5870_at_x14	4.23E-09
YGL025C	PGD1	5038_at_x05	4.61E-09
YGR158C	MTR3	4859_at_x13	4.69E-09
YKL160W	ELF1	10759_at_x07	4.92E-09
YJR032W	CPR7	11007_at_x11	6.94E-09
YLR231C	BNA5	10079_at_x10	8.13E-09
YHL048W	COS8	3188_at_x15	8.75E-09
YCR083W	TRX3	6822_at_x02	9.69E-09
YIL111W	COX5b	4027_at_x14	2.57E-08
YGL051W	MST27	5055_at_x08	2.70E-08
YAL051W	OAF1	11358_at_x01	2.71E-08
YER026C	CHO1	5681_at_x09	4.52E-08
YDL180W	YDL180W	6685_at_x14	5.96E-08
YGL035C	MIG1	5027_at_x05	6.48E-08
YDR034C	LYS14	6448_at_x14	6.92E-08
YIL061C	SNP1	4156_at_x06	8.63E-08
YLR206W	ENT2	10144_at_x01	8.67E-08
YJR084W	YJR084W	10923_at_x14	1.71E-07
YCL026C-A	FRM2	6906_at_x06	2.80E-07
YGR012W	MCY1	4985_at_x15	3.91E-07
YBR163W	EXO5	7210_at_x08	4.37E-07
YJL036W	SNX4	11077_at_x11	5.38E-07
YML004C	GLO1	9665_at_x15	5.46E-07
YJR074W	MOG1	10958_at_x13	5.83E-07
YBL040C	ERD2	7412_at_x14	6.03E-07
YKL096W	CWP1	10463_at_x00	6.10E-07
YGL017W	ATE1	5045_at_x01	6.76E-07
YJR056C	YJR056C	10985_at_x02	6.82E-07
YPL064C	CWC27	7825_at_x01	7.83E-07
YDR144C	MKC7	6335_at_x09	9.82E-07
YDL089W	NUR1	6552_at_x04	9.99E-07

YGR023W	MTL1	4996_at_x03	1.06E-06
YLL028W	TPO1	10358_at_x06	1.13E-06
YLR430W	SEN1	2867_s_at_x00	1.18E-06
YML098W	TAF13	9746_at_x10	1.18E-06
YPL144W	POC4	7881_at_x04	1.43E-06
YGR152C	RSR1	4853_at_x00	1.68E-06
YLR046C	YLR046C	10298_at_x05	1.78E-06
YJL140W	RPB4	11154_at_x15	1.80E-06
YER185W	PUG1	5534_at_x02	1.85E-06
YJL158C	CIS3	11180_at_x06	1.92E-06
YFR037C	RSC8	5336_at_x09	2.01E-06
YPL009C	RQC2	7744_at_x08	2.02E-06
YMR010W	ANY1	9632_at_x09	2.12E-06
YLR455W	PDP3	9859_at_x05	2.72E-06
YOR048C	RAT1	8521_at_x07	3.03E-06
YKL183W	LOT5	10456_at_x09	3.04E-06
YDR531W	CAB1	5955_at_x12	3.06E-06
YBR161W	CSH1	7207_at_x04	3.07E-06
YDR064W	RPS13	6433_at_x13	3.41E-06
YDR524C	AGE1	5925_at_x15	3.64E-06
YHR033W	YHR033W	4507_at_x13	3.66E-06
YLR047C	FRE8	10299_at_x07	4.50E-06
YKR051W	YKR051W	10523_at_x07	5.03E-06
YJL183W	MNN11	11197_at_x12	5.04E-06
YKR085C	MRPL20	10464_at_x00	5.10E-06
YNL046W	YNL046W	8896_at_x09	5.78E-06
YJL126W	NIT2	11124_at_x10	6.16E-06
YMR112C	MED11	9508_at_x12	6.53E-06
YER022W	SRB4	5723_at_x13	6.65E-06
YOR088W	YOR088W	8469_g_at_x03	6.67E-06
YMR115W	MGR3	9511_at_x02	6.98E-06
YCL009C	ILV6	6871_at_x02	6.98E-06
YJL163C	YJL163C	11175_at_x10	7.51E-06
YPL233W	NSL1	7971_at_x09	8.28E-06
YOR144C	ELG1	8391_at_x07	9.94E-06
YKL092C	BUD2	10647_at_x07	1.09E-05
YOL153C	YOL153C	8727_at_x01	1.19E-05
YJR073C	OPI3	10957_at_x00	1.22E-05
YIL040W	APQ12	4131_at_x05	1.42E-05
YLR172C	DPH5	10152_at_x07	1.42E-05
YGL226C-A	OST5	5243_at_x06	1.47E-05
YLR248W	RCK2	2910_at_x05	1.52E-05
YHR034C	PIH1	4508_at_x14	1.97E-05
YJL204C	RCY1	11222_at_x06	1.98E-05
YER037W	PHM8	5449_at_x11	2.13E-05
YNL326C	PFA3	9204_at_x02	2.15E-05
YLR375W	STP3	9956_at_x07	2.80E-05

YLR258W	GSY2	10060_at_x04	3.07E-05
YLL024C	SSA2	10362_at_x10	3.38E-05
YIL082W	Unknown	11428_at_x06	3.43E-05
YGL226W	MTC3	5244_at_x04	3.53E-05
YNL034W	YNL034W	8907_s_at_x03	3.76E-05
YBR147W	RTC2	7193_at_x10	3.89E-05
YDR497C	ITR1	5966_at_x12	4.38E-05
YLR057W	MNL2	10266_at_x05	4.38E-05
YIL119C	RPI1	4192_at_x12	4.54E-05
YBR137W	YBR137W	7228_at_x11	4.56E-05
YBR168W	PEX32	7170_at_x13	4.69E-05
YOR142W	LSC1	8389_at_x15	4.83E-05
YJL178C	ATG27	11202_at_x15	4.96E-05
YNL242W	ATG2	8750_at_x06	5.25E-05
YER144C	UBP5	5584_at_x06	5.73E-05
YLR287C	YLR287C	9783_at_x15	5.85E-05
YML096W	YML096W	9748_at_x13	5.95E-05
YLR443W	ECM7	9893_at_x10	6.77E-05
YML091C	RPM2	9754_at_x01	7.18E-05
YFR013W	IOC3	5354_at_x10	8.04E-05
YGR209C	TRX2	4774_at_x05	8.48E-05
YML013W	UBX2	9652_at_x12	9.30E-05
YGR001C	EFM5	5018_at_x02	9.40E-05
YHR054C	YHR054C	4484_s_at_x02	9.52E-05
YGL192W	IME4	5187_at_x09	1.07E-04
YJR025C	BNA1	10873_at_x15	1.24E-04
YOL130W	ALR1	8704_at_x03	1.28E-04
YLR368W	MDM30	9949_at_x05	1.42E-04
YIL043C	CBR1	4128_at_x12	1.73E-04
YGR289C	MAL11	4719_at_x02	1.81E-04
YLL038C	ENT4	10393_at_x11	1.83E-04
YHR039C	MSC7	4513_at_x10	2.02E-04
YLR426W	TDA5	9873_at_x09	2.28E-04
YOR101W	RAS1	8438_at_x08	2.41E-04
YBR061C	TRM7	7284_at_x12	2.75E-04
YNL135C	FPR1	8987_at_x10	2.80E-04
YLR176C	RFX1	10156_at_x15	2.83E-04
YER035W	EDC2	5690_at_x09	2.86E-04
YGR184C	UBR1	2147_at_x01	2.87E-04
YIL133C	RPL16a	4223_at_x03	2.95E-04
YLR059C	REX2	10268_at_x01	3.03E-04
YER046W	SPO73	5499_at_x02	3.21E-04
YOL073C	DSC2	8624_at_x10	3.62E-04
YOR103C	OST2	8439_at_x11	3.66E-04
YGR168C	YGR168C	4823_at_x00	3.89E-04
YMR123W	PKR1	9522_at_x06	4.79E-04
YER186C	YER186C	5535_at_x08	4.84E-04

YBL007C	SLA1	7354_at_x10	5.01E-04
YEL058W	PCM1	5777_at_x04	5.24E-04
YNR012W	URK1	8860_at_x09	5.74E-04
YDL147W	RPN5	6628_at_x15	6.74E-04
YGR207C	CIR1	4817_at_x04	6.85E-04
YJL051W	IRC8	11062_at_x04	7.08E-04
YKL110C	KTI12	10674_at_x03	7.54E-04
YOR196C	LIP5	8353_at_x08	7.87E-04
YLL061W	MMP1	10415_at_x13	8.20E-04
YER020W	GPA2	5495_at_x13	8.64E-04
YLL035W	GRC3	10351_at_x06	8.70E-04
YDL171C	GLT1	2416_at_x04	8.93E-04
YLR257W	YLR257W	10059_at_x00	8.96E-04
YJR103W	URA8	10942_at_x05	9.19E-04
YDR528W	HLR1	5952_at_x08	9.68E-04
YOL097C	WRS1	8123_at_x07	1.04E-03
YJL212C	OPT1	11260_at_x12	1.15E-03
YGR211W	ZPR1	4776_at_x14	1.15E-03
YIR026C	YVH1	4062_at_x07	1.16E-03
YDR420W	HKR1	2334_s_at_x03	1.17E-03
YJR088C	EMC2	10927_at_x00	1.18E-03
YEL068C	YEL068C	5814_at_x00	1.25E-03
YBR121C	GRS1	7257_at_x09	1.37E-03
YJR068W	RFC2	10952_at_x08	1.38E-03
YPL001W	HAT1	7752_at_x07	1.39E-03
YER116C	SLX8	5601_at_x14	1.43E-03
YGR006W	PRP18	5023_at_x00	1.46E-03
YDL164C	CDC9	6656_at_x14	1.46E-03
YDR319C	YFT2	6150_at_x06	1.55E-03
YJL149W	DAS1	11189_at_x01	1.57E-03
YBL017C	PEP1	2455_at_x06	1.57E-03
YBL020W	RFT1	7387_at_x06	1.74E-03
YDR058C	TGL2	6427_at_x03	1.79E-03
YER023W	PRO3	5678_at_x00	1.97E-03
YDR515W	SLF1	5939_at_x03	2.04E-03
YPR196W	YPR196W	7548_at_x14	2.10E-03
YGR150C	CCM1	4850_at_x02	2.25E-03
YDL135C	RDI1	6639_at_x01	2.33E-03
YIL158W	AIM20	4243_at_x05	2.35E-03
YEL056W	HAT2	5779_at_x08	2.40E-03
YEL020W-A	TIM9	5488_at_x12	2.40E-03
YMR209C	YMR209C	9436_at_x04	2.64E-03
YKR075C	YKR075C	10500_at_x00	2.80E-03
YJL208C	NUC1	11218_at_x15	2.87E-03
YGR028W	MSP1	4956_at_x13	2.95E-03
YJR008W	MHO1	10830_at_x03	3.17E-03
YNL215W	IES2	9088_at_x10	3.21E-03

YBL029W	YBL029W	6998_at_x08	3.31E-03
YNR036C	MRPS12	8839_at_x00	3.50E-03
YLR064W	PER33	10273_at_x08	3.60E-03
YJR108W	ABM1	10901_at_x07	3.60E-03
YER150W	SPI1	5544_at_x08	3.60E-03
YDL111C	RRP42	6575_at_x00	3.68E-03
YER019W	ISC1	5719_at_x13	3.71E-03
YGR222W	PET54	4788_at_x07	4.03E-03
YMR215W	GAS3	9397_at_x01	4.19E-03
YNR034W	SOL1	8788_at_x00	4.27E-03
YBL030C	PET9	7375_at_x15	4.77E-03
YHR016C	YSC84	4535_at_x14	4.91E-03
YIL112W	HOS4	4199_at_x06	5.42E-03
YOR043W	WHI2	8516_at_x00	5.46E-03
YOR251C	TUM1	8318_at_x01	5.65E-03
YNL022C	RCM1	8742_at_x06	5.85E-03
YGL004C	RPN14	5013_at_x15	5.94E-03
YBL079W	NUP170	2446_at_x06	6.10E-03
YOR127W	RGA1	8419_at_x07	6.29E-03
YCR031C	RPS14a	2438_at_x03	6.35E-03
YML071C	COG8	9728_at_x02	6.53E-03
YDR180W	SCC2	6282_at_x03	6.73E-03
YMR062C	ARG7	9592_at_x03	6.84E-03
YDL097C	RPN6	6589_at_x13	7.08E-03
YBR037C	SCO1	7351_at_x00	7.18E-03
YGR149W	GPC1	4849_at_x04	7.23E-03
YGL257C	MNT2	5256_at_x15	7.35E-03
YGL178W	MPT5	5202_at_x00	7.73E-03
YOR371C	GPB1	8170_at_x08	7.76E-03
YBR008C	FLR1	7367_at_x15	7.78E-03
YDL103C	QRI1	6583_at_x15	7.92E-03
YLR442C	SIR3	9891_at_x15	8.00E-03
YHR209W	CRG1	4290_at_x06	8.00E-03
YBR071W	YBR071W	7294_at_x14	8.31E-03
YJR040W	GEF1	10969_at_x03	8.54E-03
YDL155W	CLB3	6620_at_x15	8.82E-03
YML110C	COQ5	9210_at_x05	8.87E-03
YEL055C	POL5	5780_at_x07	8.99E-03
YOL147C	PEX11	8687_at_x14	9.11E-03
YHR050W	SMF2	4480_at_x15	9.15E-03
YNL200C	NNR1	9059_at_x07	9.34E-03
YBR157C	ICS2	7026_at_x11	9.41E-03
YBR193C	MED8	7149_at_x15	9.55E-03
YEL076W-C	Unknown	5808_i_at_x01	9.63E-03
YKL021C	MAK11	10586_at_x01	9.73E-03
YBR126C	TPS1	7217_at_x04	9.88E-03
YDL211C	YDL211C	6697_at_x14	9.89E-03

YIR022W	SEC11	4058_at_x11	1.02E-02
YEL071W	DLD3	5813_at_x00	1.02E-02
YIL066C	RNR3	4045_s_at_x07	1.05E-02
YML007W	YAP1	9662_at_x07	1.05E-02
YBR154C	RPB5	7200_at_x13	1.05E-02
YNL024C	EFM6	8774_at_x00	1.05E-02
YPL031C	PHO85	7767_at_x07	1.10E-02
YNL124W	NAF1	8998_at_x00	1.11E-02
YDL002C	NHP10	6460_at_x04	1.12E-02
YLR435W	TSR2	9882_at_x08	1.14E-02
YIL009W	FAA3	4118_at_x07	1.16E-02
YBR077C	SLM4	7300_at_x03	1.18E-02
YGR258C	RAD2	4596_at_x07	1.20E-02
YER049W	TPA1	5510_at_x14	1.22E-02
YBR229C	ROT2	7139_at_x15	1.24E-02
YLR237W	THI7	10084_at_x10	1.24E-02
YDL047W	SIT4	5852_at_x15	1.27E-02
YKL116C	PRR1	10669_at_x07	1.27E-02
YDR351W	SBE2	6137_at_x10	1.30E-02
YMR046C	YMR046C	2822_at_x03	1.31E-02
YOL018C	TLG2	8126_at_x12	1.32E-02
YBR087W	RFC5	7265_at_x05	1.33E-02
YGL176C	YGL176C	5204_at_x04	1.36E-02
YBL089W	AVT5	7452_at_x15	1.39E-02
YHR029C	YHI9	4298_at_x08	1.40E-02
YOR117W	RPT5	8454_at_x09	1.40E-02
YKR052C	MRS4	10524_at_x01	1.41E-02
YJL047C	RTT101	11066_at_x15	1.42E-02
YJR086W	STE18	10927_at_x12	1.43E-02
YOR264W	DSE3	8284_at_x08	1.44E-02
YGL242C	YGL242C	5271_at_x02	1.46E-02
YIL142W	CCT2	4259_at_x14	1.52E-02
YCL024W	KCC4	6908_at_x10	1.53E-02
YLL040C	VPS13	2932_at_x01	1.59E-02
YOR389W	YOR389W	8143_at_x00	1.62E-02
YKL019W	RAM2	10452_at_x05	1.67E-02
YPL183C	RTT10	7932_at_x02	1.68E-02
YBR225W	YBR225W	7135_at_x15	1.69E-02
YDR481C	PHO8	5995_at_x12	1.70E-02
YNL138W	SRV2	8984_at_x09	1.70E-02
YNL280C	ERG24	9159_at_x00	1.70E-02
YBR249C	ARO4	7114_at_x13	1.75E-02
YML121W	GTR1	9764_at_x09	1.78E-02
YHL007C	STE20	4555_at_x10	1.80E-02
YNL322C	KRE1	9208_at_x15	1.80E-02
YNL321W	VNX1	9163_at_x03	1.82E-02
YKL125W	RRN3	10703_at_x13	1.85E-02

YGR208W	SER2	4818_at_x05	1.88E-02
YOL081W	IRA2	2699_at_x07	1.89E-02
YLR056W	ERG3	10265_at_x10	1.93E-02
YPL226W	NEW1	7978_at_x11	1.94E-02
YKL184W	SPE1	10779_at_x00	1.97E-02
YLR452C	SST2	9856_at_x07	1.97E-02
YDR513W	GRX2	5937_at_x10	1.98E-02
YPL234C	VMA11	7970_at_x07	2.01E-02
YLR181C	VTA1	10162_at_x15	2.05E-02
YBL105C	PKC1	7481_at_x13	2.07E-02
YFL018C	LPD1	5275_i_at_x02	2.10E-02
YPL071C	YPL071C	7818_at_x01	2.11E-02
YCR071C	IMG2	6812_at_x10	2.13E-02
YAL009W	SPO7	11312_at_x00	2.15E-02
YDL150W	RPC53	6624_at_x01	2.16E-02
YGL195W	GCN1	2263_g_at_x07	2.18E-02
YMR158W	MRPS8	9469_at_x13	2.23E-02
YJR097W	JJJ3	10936_at_x15	2.28E-02
YOR092W	ECM3	8474_at_x06	2.30E-02
YPR080W	TEF1	7522_at_x15	2.35E-02
YOR033C	EXO1	8506_at_x05	2.38E-02
YOL093W	TRM10	8650_at_x11	2.40E-02
YJL058C	BIT61	11100_at_x13	2.43E-02
YEL064C	AVT2	5770_at_x06	2.44E-02
YFL047W	RGD2	5433_at_x10	2.45E-02
YFL016C	MDJ1	5370_at_x07	2.48E-02
YOR054C	VHS3	8482_at_x04	2.49E-02
YHR104W	GRE3	4442_at_x03	2.49E-02
YIL171W	YIL171W	4282_f_at_x04	2.50E-02
YDL116W	NUP84	6615_at_x01	2.50E-02
YLR449W	FPR4	9853_at_x01	2.53E-02
YFR004W	RPN11	5346_at_x05	2.58E-02
YFR011C	MIC19	5353_at_x00	2.59E-02
YGR157W	CHO2	4858_at_x11	2.64E-02
YLR096W	KIN2	2883_at_x07	2.65E-02
YML072C	TCB3	9727_at_x12	2.66E-02
YML048W	GSF2	9709_at_x03	2.71E-02
YKL073W	LHS1	10668_at_x04	2.72E-02
YMR154C	RIM13	9465_at_x13	2.74E-02
YFR047C	BNA6	5301_at_x13	2.74E-02
YJL187C	SWE1	11193_at_x15	2.78E-02
YPR015C	YPR015C	7722_at_x06	2.88E-02
YDL167C	NRP1	6653_at_x11	2.94E-02
YDR530C	APA2	5954_at_x03	3.03E-02
YBR222C	PCS60	7132_at_x01	3.09E-02
YDR178W	SDH4	6279_at_x10	3.09E-02
YOR080W	DIA2	8461_at_x00	3.17E-02

YKL067W	YNK1	10630_at_x12	3.23E-02
YJL218W	YJL218W	11254_at_x12	3.27E-02
YMR192W	GYL1	9417_at_x14	3.27E-02
YBR239C	ERT1	6971_at_x12	3.33E-02
YFR042W	KEG1	5341_at_x03	3.39E-02
YHR027C	RPN1	4501_at_x05	3.39E-02
YHR063C	PAN5	4491_at_x08	3.47E-02
YOL026C	MIM1	8581_at_x04	3.48E-02
YMR067C	UBX4	9597_at_x10	3.53E-02
YNL074C	MLF3	8958_at_x14	3.56E-02
YHR023W	MYO1	2110_s_at_x07	3.58E-02
YCL039W	GID7	6893_at_x00	3.59E-02
YER088C	DOT6	5617_at_x13	3.63E-02
YNL278W	CAF120	9161_at_x03	3.64E-02
YLR180W	SAM1	9779_at_x13	3.66E-02
YGR194C	XKS1	4804_at_x15	3.70E-02
YDR383C	NKP1	6081_at_x10	3.72E-02
YBR196C	PGI1	7152_at_x15	3.81E-02
YPL058C	PDR12	7496_at_x02	3.86E-02
YIL165C	YIL165C	4237_at_x12	3.91E-02
YER182W	FMP10	5531_at_x15	3.97E-02
YPL012W	RRP12	7786_at_x15	3.97E-02
YDL133W	SRF1	6595_at_x02	4.08E-02
YBL014C	RRN6	7393_at_x14	4.09E-02
YOL088C	MPD2	8655_at_x03	4.12E-02
YJL118W	YJL118W	11131_at_x05	4.17E-02
YMR005W	TAF4	9627_at_x09	4.22E-02
YPL237W	SUI3	8012_at_x12	4.34E-02
YPL147W	PXA1	7923_at_x15	4.39E-02
YDR321W	ASP1	6152_at_x13	4.42E-02
YGR288W	MAL13	4718_at_x02	4.56E-02
YFL049W	SWP82	5431_at_x14	4.65E-02
YJR036C	HUL4	10966_at_x12	4.71E-02
YML036W	CGI121	9672_at_x06	4.71E-02
YML038C	YMD8	9670_at_x04	4.78E-02
YIR028W	DAL4	4064_at_x02	4.79E-02
YOL062C	APM4	8635_at_x01	4.89E-02

Table S2: List of all pairwise connections and bootstrap frequency and corresponding coefficients.

Source Gene (ORF Name)	Source Gene (Gene Name)	Target Gene (ORF Name)	Target Gene (Gene Name)	Bootstrap Frequency	Coefficient
YEL021W	URA3	YER088C	DOT6	1.0000	-0.4990
YFL047W	RGD2	YLR452C	SST2	1.0000	0.5704
YHR034C	PIH1	YHR033W	YHR033W	1.0000	0.5577
YLR155C	ASP3-1	YIL066C	RNR3	1.0000	-0.4472
YLR181C	VTA1	YLR180W	SAM1	1.0000	0.4847
YLR452C	SST2	YFL047W	RGD2	1.0000	0.6046
YLR464W	YLR464W	YEL076W-C	Unknown	1.0000	0.5714
YKL073W	LHS1	YGR028W	MSP1	0.9999	0.3485
YLR180W	SAM1	YLR181C	VTA1	0.9999	0.5491
YIL133C	RPL16a	YDR064W	RPS13	0.9997	0.6995
YIL119C	RPI1	YDR144C	MKC7	0.9997	-0.4163
YHR033W	YHR033W	YHR034C	PIH1	0.9996	0.6239
YHR043C	DOG2	YLR237W	THI7	0.9992	-0.3227
YBR196C	PGI1	YBL020W	RFT1	0.9988	0.4588
YBL020W	RFT1	YBR196C	PGI1	0.9985	0.4280
YEL056W	HAT2	YEL055C	POL5	0.9981	0.3138
YBR147W	RTC2	YJR025C	BNA1	0.9980	0.6270
YAL056W	GPB2	YOL147C	PEX11	0.9940	-0.4379
YCL018W	LEU2	YEL071W	DLD3	0.9931	-0.2646
YAL051W	OAF1	YGL004C	RPN14	0.9914	0.4359
YDR064W	RPS13	YIL133C	RPL16a	0.9902	0.4493
YEL076W-C	Unknown	YLR464W	YLR464W	0.9894	0.2822
YDL147W	RPN5	YDL097C	RPN6	0.9884	0.5847
YJR073C	OPI3	YDR497C	ITR1	0.9871	0.5756
YGR184C	UBR1	YGL195W	GCN1	0.9863	0.2998
YHR023W	MYO1	YFL049W	SWP82	0.9853	0.4535
YDR497C	ITR1	YJR008W	MHO1	0.9842	0.3354
YIL043C	CBR1	YDR321W	ASP1	0.9823	0.5072
YCR041W	YCR041W	YJL171C	TOH1	0.9822	-0.4116
YGR028W	MSP1	YKL073W	LHS1	0.9820	0.4867
YCL018W	LEU2	YHR063C	PAN5	0.9817	-0.3329
YOR092W	ECM3	YIL119C	RPI1	0.9765	0.3640
YPL064C	CWC27	YKL110C	KTI12	0.9706	0.3136
YFR047C	BNA6	YLR231C	BNA5	0.9705	0.6570
YJL217W	REE1	YFL056C	AAD6	0.9702	-0.2268
YLR430W	SEN1	YGL207W	SPT16	0.9677	0.2521
YLR046C	YLR046C	YLR047C	FRE8	0.9673	0.3440
YDR528W	HLR1	YKL073W	LHS1	0.9671	0.2692
YIL119C	RPI1	YBR157C	ICS2	0.9658	0.3653
YAL051W	OAF1	YEL068C	YEL068C	0.9648	-0.2857
YJR025C	BNA1	YBR147W	RTC2	0.9647	0.2827

YBR249C	ARO4	YEL071W	DLD3	0.9626	0.5022
YLR047C	FRE8	YLR046C	YLR046C	0.9619	0.3519
YBR037C	SCO1	YER182W	FMP10	0.9616	0.4932
YDL147W	RPN5	YOR117W	RPT5	0.9615	0.4508
YLR064W	PER33	YPL234C	VMA11	0.9612	0.3063
YFL049W	SWP82	YHR023W	MYO1	0.9592	0.2609
YGR207C	CIR1	YJL140W	RPB4	0.9589	0.3513
YJR086W	STE18	YKL160W	ELF1	0.9577	0.3497
YBR249C	ARO4	YMR062C	ARG7	0.9575	0.3579
YBR137W	YBR137W	YBR126C	TPS1	0.9570	0.3225
YEL055C	POL5	YEL056W	HAT2	0.9567	0.7187
YGR184C	UBR1	YOL081W	IRA2	0.9567	0.3605
YDL002C	NHP10	YDR339C	FCF1	0.9552	0.4545
YFR037C	RSC8	YLR455W	PDP3	0.9551	0.3732
YCR071C	IMG2	YKR085C	MRPL20	0.9548	0.3513
YKL110C	KTI12	YPL064C	CWC27	0.9526	0.3933
YIL119C	RPI1	YOR092W	ECM3	0.9519	0.2745
YPL234C	VMA11	YLR064W	PER33	0.9509	0.5516
YBR249C	ARO4	YHR029C	YHI9	0.9496	0.4029
YOR251C	TUM1	YBR121C	GRS1	0.9474	0.2804
YBR249C	ARO4	YDR531W	CAB1	0.9472	0.5431
YOL093W	TRM10	YBR154C	RPB5	0.9466	0.4419
YJL047C	RTT101	YGR288W	MAL13	0.9448	0.4420
YBL007C	SLA1	YBR229C	ROT2	0.9435	0.3700
YKL073W	LHS1	YJL036W	SNX4	0.9411	0.3857
YDR351W	SBE2	YDR034C	LYS14	0.9410	0.3304
YBR071W	YBR071W	YBR225W	YBR225W	0.9398	0.2259
YBR132C	AGP2	YOR264W	DSE3	0.9396	0.2817
YBR154C	RPB5	YBR061C	TRM7	0.9390	0.6173
YJR073C	OPI3	YER026C	CHO1	0.9389	0.4519
YEL021W	URA3	YER116C	SLX8	0.9381	0.3273
YHR029C	YHI9	YIL165C	YIL165C	0.9380	0.7819
YGR012W	MCY1	YHR063C	PAN5	0.9358	0.2640
YOL081W	IRA2	YGR184C	UBR1	0.9357	0.4018
YNL200C	NNR1	YHR104W	GRE3	0.9355	0.4475
YGL004C	RPN14	YAL051W	OAF1	0.9354	0.4729
YBR157C	ICS2	YIL119C	RPI1	0.9350	0.2045
YIL111W	COX5b	YER035W	EDC2	0.9327	0.2924
YJR073C	OPI3	YJL212C	OPT1	0.9321	-0.2273
YIL015C-A	YIL015C-A	YMR046C	YMR046C	0.9318	0.2653
YOR117W	RPT5	YDL147W	RPN5	0.9316	0.2321
YIL171W	YIL171W	YDR441C	APT2	0.9307	0.3353
YOR054C	VHS3	YLR206W	ENT2	0.9307	0.3206
YBR077C	SLM4	YBR037C	SCO1	0.9306	0.3047
YMR062C	ARG7	YBR249C	ARO4	0.9300	0.3710
YOR103C	OST2	YGR209C	TRX2	0.9300	0.3146
YJR040W	GEF1	YGR157W	CHO2	0.9298	0.2346

YOR251C	TUM1	YER023W	PRO3	0.9298	0.3531
YHR027C	RPN1	YDL097C	RPN6	0.9297	0.2436
YKR085C	MRPL20	YEL020W-A	TIM9	0.9296	0.3872
YJL208C	NUC1	YFL016C	MDJ1	0.9293	0.4257
YFL018C	LPD1	YPL031C	PHO85	0.9264	0.2966
YOR142W	LSC1	YDR178W	SDH4	0.9253	0.3571
YLR452C	SST2	YDL089W	NUR1	0.9233	0.3505
YBL030C	PET9	YFR011C	MIC19	0.9222	0.2626
YML038C	YMD8	YBR137W	YBR137W	0.9212	-0.1951
YPL233W	NSL1	YLR442C	SIR3	0.9210	0.2528
YNL074C	MLF3	YKR080W	MTD1	0.9205	0.3844
YOL089C	HAL9	YLL028W	TPO1	0.9205	-0.3118
YBL030C	PET9	YOR142W	LSC1	0.9203	0.2974
YML091C	RPM2	YMR046C	YMR046C	0.9196	0.3406
YJL036W	SNX4	YML071C	COG8	0.9175	0.3249
YLR237W	THI7	YBR239C	ERT1	0.9165	0.1998
YBL079W	NUP170	YBR077C	SLM4	0.9164	0.4374
YEL064C	AVT2	YKR051W	YKR051W	0.9160	0.2721
YNL322C	KRE1	YER019W	ISC1	0.9159	0.4340
YIL040W	APQ12	YDR367W	KEI1	0.9153	0.3307
YBR137W	YBR137W	YBR225W	YBR225W	0.9146	0.2766
YDR383C	NKP1	YPL001W	HAT1	0.9130	0.4103
YIL119C	RPI1	YFL047W	RGD2	0.9121	0.1975
YGL195W	GCN1	YGR184C	UBR1	0.9115	0.3798
YDR321W	ASP1	YIL043C	CBR1	0.9109	0.4926
YKL160W	ELF1	YGL226C-A	OST5	0.9101	0.2530
YKL160W	ELF1	YJR086W	STE18	0.9099	0.3127
YER026C	CHO1	YJR073C	OPI3	0.9098	0.3161
YBL017C	PEP1	YBR229C	ROT2	0.9088	0.3147
YEL068C	YEL068C	YAL051W	OAF1	0.9082	-0.2839
YIR028W	DAL4	YJL218W	YJL218W	0.9062	0.4861
YOL062C	APM4	YEL058W	PCM1	0.9054	0.3151
YDR531W	CAB1	YDR034C	LYS14	0.9053	0.3123
YFR011C	MIC19	YBL030C	PET9	0.9035	0.3804
YDR497C	ITR1	YJR073C	OPI3	0.9024	0.2420
YMR046C	YMR046C	YGL035C	MIG1	0.9021	-0.3225
YML048W	GSF2	YMR215W	GAS3	0.9005	0.3793
YOL104C	NDJ1	YLL028W	TPO1	0.9001	-0.4246
YDR319C	YFT2	YDR441C	APT2	0.8987	0.3104
YDR528W	HLR1	YDL211C	YDL211C	0.8977	0.3785
YOR142W	LSC1	YBL030C	PET9	0.8962	0.3817
YIL111W	COX5b	YJR103W	URA8	0.8934	0.3163
YOL093W	TRM10	YLR287C	YLR287C	0.8931	0.3405
YHR034C	PIH1	YKR080W	MTD1	0.8902	-0.2946
YLR056W	ERG3	YNL280C	ERG24	0.8884	0.2795
YOR080W	DIA2	YER144C	UBP5	0.8884	0.2860
YOR196C	LIP5	YMR062C	ARG7	0.8881	0.2247

YBR249C	ARO4	YCL009C	ILV6	0.8879	0.4896
YGR194C	XKS1	YBR126C	TPS1	0.8877	0.4379
YMR215W	GAS3	YML048W	GSF2	0.8867	0.3631
YDL089W	NUR1	YLR452C	SST2	0.8866	0.2250
YBR225W	YBR225W	YBR071W	YBR071W	0.8848	0.3076
YER035W	EDC2	YIL111W	COX5b	0.8844	0.5106
YFL016C	MDJ1	YJL208C	NUC1	0.8841	0.2204
YGR288W	MAL13	YJL047C	RTT101	0.8836	0.5538
YDR528W	HLR1	YIL061C	SNP1	0.8835	0.2293
YLR231C	BNA5	YFR047C	BNA6	0.8833	0.3342
YBR008C	FLR1	YGL192W	IME4	0.8830	0.2570
YGL195W	GCN1	YPL226W	NEW1	0.8828	0.3618
YDL097C	RPN6	YDL147W	RPN5	0.8827	0.4023
YIL111W	COX5b	YPL071C	YPL071C	0.8827	0.3240
YML110C	COQ5	YMR152W	YIM1	0.8819	0.3675
YDL155W	CLB3	YCR031C	RPS14a	0.8800	-0.2578
YER023W	PRO3	YOR251C	TUM1	0.8799	0.4687
YGR207C	CIR1	YOR131C	YOR131C	0.8798	0.4099
YER144C	UBP5	YJR088C	EMC2	0.8788	-0.1530
YML091C	RPM2	YLR096W	KIN2	0.8784	0.1979
YJR008W	MHO1	YDR497C	ITR1	0.8758	0.1467
YBR229C	ROT2	YBL007C	SLA1	0.8732	0.4195
YIL119C	RPI1	YLR231C	BNA5	0.8729	0.1642
YPL226W	NEW1	YGL195W	GCN1	0.8722	0.3996
YNL280C	ERG24	YLR056W	ERG3	0.8718	0.4432
YLR096W	KIN2	YML091C	RPM2	0.8716	0.2913
YBR137W	YBR137W	YML038C	YMD8	0.8708	-0.3063
YEL071W	DLD3	YJR074W	MOG1	0.8704	-0.3677
YCL009C	ILV6	YKL184W	SPE1	0.8685	0.2640
YHL007C	STE20	YIL166C	YIL166C	0.8678	-0.3363
YOL018C	TLG2	YOL043C	NTG2	0.8671	0.4011
YHR034C	PIH1	YER035W	EDC2	0.8663	0.2160
YMR123W	PKR1	YLR172C	DPH5	0.8660	0.1807
YGL207W	SPT16	YHR027C	RPN1	0.8653	0.2126
YLR455W	PDP3	YDR351W	SBE2	0.8613	0.1394
YNL280C	ERG24	YHR039C	MSC7	0.8606	0.3659
YKL096W	CWP1	YDL111C	RRP42	0.8601	0.2670
YGR157W	CHO2	YJR040W	GEF1	0.8600	0.4000
YOR264W	DSE3	YNL322C	KRE1	0.8586	-0.2831
YBL030C	PET9	YJL212C	OPT1	0.8581	-0.3210
YKR085C	MRPL20	YCR071C	IMG2	0.8580	0.3773
YBR225W	YBR225W	YBR137W	YBR137W	0.8574	0.3083
YER182W	FMP10	YBR037C	SCO1	0.8573	0.2997
YOR033C	EXO1	YIL066C	RNR3	0.8572	0.3437
YBL079W	NUP170	YBR121C	GRS1	0.8570	0.5167
YGL035C	MIG1	YMR046C	YMR046C	0.8566	-0.3480
YOR371C	GPB1	YIL046W	MET30	0.8565	0.5163

YDR134C	YDR134C	YCR041W	YCR041W	0.8563	0.4039
YLR464W	YLR464W	YPL016W	SWI1	0.8563	0.1956
YOL062C	APM4	YKL116C	PRR1	0.8561	0.2500
YBR196C	PGI1	YBR222C	PCS60	0.8553	0.2214
YER022W	SRB4	YOR033C	EXO1	0.8551	0.2348
YMR046C	YMR046C	YML091C	RPM2	0.8548	0.4260
YDR034C	LYS14	YDR351W	SBE2	0.8544	0.4336
YOL062C	APM4	YPL009C	RQC2	0.8542	-0.2243
YBR222C	PCS60	YER182W	FMP10	0.8538	-0.3227
YKL116C	PRR1	YGR158C	MTR3	0.8535	0.3225
YGL053W	PRM8	YFL016C	MDJ1	0.8531	0.2099
YAL051W	OAF1	YGR258C	RAD2	0.8522	0.2496
YER019W	ISC1	YNL046W	YNL046W	0.8522	0.3533
YER035W	EDC2	YHR034C	PIH1	0.8516	0.2920
YJL178C	ATG27	YNL024C	EFM6	0.8516	0.4091
YIL082W	Unknown	YGR006W	PRP18	0.8501	-0.2296
YBR037C	SCO1	YGL208W	SIP2	0.8500	0.3450
YER150W	SPI1	YBR126C	TPS1	0.8480	0.2718
YER150W	SPI1	YNL200C	NNR1	0.8474	0.3333
YML007W	YAP1	YML016C	PPZ1	0.8452	0.3824
YMR112C	MED11	YBR067C	TIP1	0.8439	-0.2030
YDL002C	NHP10	YFR011C	MIC19	0.8411	0.2925
YGR028W	MSP1	YLR257W	YLR257W	0.8399	0.4318
YDR351W	SBE2	YLR455W	PDP3	0.8387	0.2975
YCR071C	IMG2	YMR158W	MRPS8	0.8384	0.2472
YGR207C	CIR1	YHR029C	YHI9	0.8383	0.1972
YOR117W	RPT5	YIL142W	CCT2	0.8381	0.1617
YLR237W	THI7	YJL212C	OPT1	0.8373	0.3079
YDR497C	ITR1	YER026C	CHO1	0.8339	0.2062
YJL187C	SWE1	YKL092C	BUD2	0.8338	0.4908
YGL193C	YGL193C	YGL226C-A	OST5	0.8331	0.1783
YHR039C	MSC7	YEL058W	PCM1	0.8330	0.1613
YGL017W	ATE1	YBR067C	TIP1	0.8321	0.1596
YHR104W	GRE3	YNL200C	NNR1	0.8320	0.4033
YLR231C	BNA5	YJR025C	BNA1	0.8319	0.1314
YGL192W	IME4	YBR008C	FLR1	0.8314	0.1277
YOR131C	YOR131C	YFR011C	MIC19	0.8290	0.1847
YJL126W	NIT2	YJR068W	RFC2	0.8276	0.0745
YOR092W	ECM3	YBR067C	TIP1	0.8266	0.2360
YCR031C	RPS14a	YIL133C	RPL16a	0.8243	0.3011
YBR008C	FLR1	YHR115C	DMA1	0.8242	0.3201
YML071C	COG8	YJL036W	SNX4	0.8236	0.2460
YER150W	SPI1	YER037W	PHM8	0.8220	0.5472
YER037W	PHM8	YER150W	SPI1	0.8206	0.2355
YCL024W	KCC4	YDL164C	CDC9	0.8194	0.2251
YOR088W	YOR088W	YJL030W	MAD2	0.8183	-0.2307
YLR180W	SAM1	YLL061W	MMP1	0.8180	0.3512

YJL158C	CIS3	YNL322C	KRE1	0.8179	0.4203
YNL074C	MLF3	YNL322C	KRE1	0.8174	0.2744
YML096W	YML096W	YFR047C	BNA6	0.8172	-0.2679
YNL124W	NAF1	YGL176C	YGL176C	0.8171	0.4122
YEL020W-A	TIM9	YJL208C	NUC1	0.8165	0.3091
YNL024C	EFM6	YJL178C	ATG27	0.8162	0.2736
YLL061W	MMP1	YLR180W	SAM1	0.8139	0.2144
YBR077C	SLM4	YBR008C	FLR1	0.8133	0.1860
YBR229C	ROT2	YBL017C	PEP1	0.8128	0.1871
YGL226C-A	OST5	YGL193C	YGL193C	0.8122	0.3497
YEL064C	AVT2	YIL066C	RNR3	0.8119	0.1470
YGL017W	ATE1	YIL112W	HOS4	0.8104	0.2896
YLR176C	RFX1	YNL138W	SRV2	0.8104	0.2530
YKR080W	MTD1	YML016C	PPZ1	0.8099	0.2169
YHR027C	RPN1	YHR050W	SMF2	0.8097	0.2578
YEL020W-A	TIM9	YKR085C	MRPL20	0.8094	0.2575
YBR163W	EXO5	YBR168W	PEX32	0.8091	0.5054
YDL171C	GLT1	YOR051C	ETT1	0.8090	0.2372
YJR086W	STE18	YJR088C	EMC2	0.8085	0.2640
YDL097C	RPN6	YDL132W	CDC53	0.8083	0.3605
YHR039C	MSC7	YNL280C	ERG24	0.8083	0.2413
YOR196C	LIP5	YFL018C	LPD1	0.8080	0.1984
YOR127W	RGA1	YIL040W	APQ12	0.8070	-0.2871
YBL017C	PEP1	YBL079W	NUP170	0.8069	0.3428
YGR001C	EFM5	YGR222W	PET54	0.8060	0.3240
YKL067W	YNK1	YDR513W	GRX2	0.8058	0.1497
YJL140W	RPB4	YGR207C	CIR1	0.8057	0.1634
YJL171C	TOH1	YPL234C	VMA11	0.8035	0.2069
YDR339C	FCF1	YFR037C	RSC8	0.8024	0.4400
YDL164C	CDC9	YDL132W	CDC53	0.7999	0.1831
YOR251C	TUM1	YGL195W	GCN1	0.7982	0.2253
YGR258C	RAD2	YOR131C	YOR131C	0.7977	-0.3221
YMR005W	TAF4	YML007W	YAP1	0.7976	0.2651
YMR123W	PKR1	YNL035C	YNL035C	0.7954	0.2450
YGR006W	PRP18	YIL082W	Unknown	0.7947	-0.0399
YER186C	YER186C	YBR222C	PCS60	0.7939	-0.2691
YDL211C	YDL211C	YDR528W	HLR1	0.7928	0.3238
YOR131C	YOR131C	YGR207C	CIR1	0.7928	0.2458
YNL322C	KRE1	YJL158C	CIS3	0.7925	0.3069
YCL009C	ILV6	YMR062C	ARG7	0.7923	0.1375
YGR258C	RAD2	YLR047C	FRE8	0.7923	0.1806
YOR264W	DSE3	YHR023W	MYO1	0.7919	0.3740
YNL138W	SRV2	YJR040W	GEF1	0.7914	0.2476
YML038C	YMD8	YMR010W	ANY1	0.7913	0.4206
YML091C	RPM2	YNL135C	FPR1	0.7911	-0.2539
YNL200C	NNR1	YER150W	SPI1	0.7910	0.4473
YLR330W	CHS5	YMR115W	MGR3	0.7901	0.2113

YPL031C	PHO85	YFL018C	LPD1	0.7901	0.2170
YGR207C	CIR1	YGL226W	MTC3	0.7897	0.2988
YPL001W	HAT1	YDR383C	NKP1	0.7889	0.2586
YBR147W	RTC2	YDR058C	TGL2	0.7880	0.5201
YGL226C-A	OST5	YOR103C	OST2	0.7880	0.2939
YAL009W	SPO7	YML071C	COG8	0.7877	-0.1818
YGL207W	SPT16	YLR449W	FPR4	0.7872	0.1438
YBR037C	SCO1	YNR036C	MRPS12	0.7868	0.2772
YJL051W	IRC8	YAR028W	YAR028W	0.7861	-0.1994
YHR027C	RPN1	YGL207W	SPT16	0.7847	0.2271
YPL058C	PDR12	YEL058W	PCM1	0.7845	0.1938
YDR528W	HLR1	YOR131C	YOR131C	0.7844	-0.2432
YDL164C	CDC9	YDR180W	SCC2	0.7834	0.2945
YBL105C	PKC1	YBL089W	AVT5	0.7819	0.2754
YER088C	DOT6	YOR092W	ECM3	0.7801	0.2934
YML091C	RPM2	YDL155W	CLB3	0.7800	0.3042
YIL061C	SNP1	YJR074W	MOG1	0.7790	-0.2423
YOL147C	PEX11	YBR137W	YBR137W	0.7787	0.1069
YNL041C	COG6	YDR351W	SBE2	0.7786	0.1788
YIL166C	YIL166C	YHL007C	STE20	0.7784	-0.2628
YMR115W	MGR3	YMR067C	UBX4	0.7771	0.2289
YBR061C	TRM7	YBR154C	RPB5	0.7764	0.0702
YDR515W	SLF1	YDL217C	TIM22	0.7763	0.2593
YOR088W	YOR088W	YHR016C	YSC84	0.7755	0.2376
YJR073C	OPI3	YER023W	PRO3	0.7754	0.2901
YCL024W	KCC4	YPL016W	SWI1	0.7748	0.3531
YGR012W	MCY1	YDL164C	CDC9	0.7741	0.1791
YFL056C	AAD6	YDR531W	CAB1	0.7738	0.1678
YJR056C	YJR056C	YGR152C	RSR1	0.7725	0.2398
YMR067C	UBX4	YMR115W	MGR3	0.7706	0.3796
YIL043C	CBR1	YGL178W	MPT5	0.7688	-0.2543
YOL026C	MIM1	YNL041C	COG6	0.7685	0.2464
YMR123W	PKR1	YJL183W	MNN11	0.7676	0.2111
YBR229C	ROT2	YBL014C	RRN6	0.7674	0.2063
YJR008W	MHO1	YML110C	COQ5	0.7662	0.3407
YLR435W	TSR2	YKL021C	MAK11	0.7662	0.3348
YIL082W	Unknown	YLR452C	SST2	0.7660	0.1487
YEL076W-C	Unknown	YLR206W	ENT2	0.7659	-0.1983
YMR158W	MRPS8	YDR515W	SLF1	0.7652	0.3256
YJL183W	MNN11	YOR054C	VHS3	0.7649	-0.4360
YLR059C	REX2	YKL184W	SPE1	0.7645	-0.1177
YKL073W	LHS1	YDR528W	HLR1	0.7640	0.1418
YBL020W	RFT1	YIL111W	COX5b	0.7634	-0.1347
YIL119C	RPI1	YMR209C	YMR209C	0.7634	0.1453
YML072C	TCB3	YNL321W	VNX1	0.7627	0.2340
YER023W	PRO3	YJR088C	EMC2	0.7621	0.3689
YFL047W	RGD2	YLR046C	YLR046C	0.7621	-0.1656

YPL147W	PXA1	YHL007C	STE20	0.7612	-0.1698
YBR061C	TRM7	YBR087W	RFC5	0.7602	0.3332
YEL058W	PCM1	YHR209W	CRG1	0.7596	0.1989
YJL208C	NUC1	YEL020W-A	TIM9	0.7586	0.3004
YBR239C	ERT1	YBR249C	ARO4	0.7584	0.0980
YHR209W	CRG1	YJL149W	DAS1	0.7577	0.2219
YPL234C	VMA11	YJL171C	TOH1	0.7573	0.3229
YLR057W	MNL2	YLR096W	KIN2	0.7571	0.2235
YLL038C	ENT4	YGL192W	IME4	0.7568	0.1026
YDR524C	AGE1	YER116C	SLX8	0.7562	-0.2322
YLR181C	VTA1	YDR180W	SCC2	0.7562	0.2785
YDL171C	GLT1	YGL242C	YGL242C	0.7558	-0.2271
YML091C	RPM2	YBL030C	PET9	0.7557	0.2258
YCR031C	RPS14a	YDL155W	CLB3	0.7553	-0.2073
YOR043W	WHI2	YJR056C	YJR056C	0.7553	0.1913
YBL105C	PKC1	YBL017C	PEP1	0.7552	0.1966
YCL009C	ILV6	YBR249C	ARO4	0.7552	0.1831
YPR022C	SDD4	YML016C	PPZ1	0.7550	0.1266
YOR088W	YOR088W	YJR040W	GEF1	0.7546	0.2400
YJL058C	BIT61	YER185W	PUG1	0.7545	0.2383
YKL019W	RAM2	YNL329C	PEX6	0.7541	0.1488
YHL007C	STE20	YPL147W	PXA1	0.7539	-0.2115
YJL058C	BIT61	YOL089C	HAL9	0.7537	0.2410
YEL058W	PCM1	YOL130W	ALR1	0.7535	0.2396
YMR046C	YMR046C	YIL015C-A	YIL015C-A	0.7530	0.3248
YHL048W	COS8	YHR209W	CRG1	0.7525	0.2305
YBL079W	NUP170	YBL014C	RRN6	0.7498	0.3320
YHR209W	CRG1	YHL048W	COS8	0.7487	0.2137
YML016C	PPZ1	YML007W	YAP1	0.7483	0.2180
YHR029C	YHI9	YBR249C	ARO4	0.7482	0.2605
YPL009C	RQC2	YOL062C	APM4	0.7475	-0.1600
YDR441C	APT2	YNL138W	SRV2	0.7471	-0.1776
YNR034W	SOL1	YML098W	TAF13	0.7471	0.2825
YJL118W	YJL118W	YGR023W	MTL1	0.7465	-0.2540
YNL034W	YNL034W	YDL047W	SIT4	0.7463	0.1471
YJR040W	GEF1	YHR050W	SMF2	0.7450	0.2361
YMR158W	MRPS8	YCR071C	IMG2	0.7450	0.2646
YDR144C	MKC7	YIL119C	RPI1	0.7448	-0.3355
YDR515W	SLF1	YER037W	PHM8	0.7446	-0.1344
YJR068W	RFC2	YJL030W	MAD2	0.7443	0.2803
YLR237W	THI7	YLR180W	SAM1	0.7443	0.2122
YBR239C	ERT1	YBL030C	PET9	0.7417	0.2852
YMR152W	YIM1	YKL067W	YNK1	0.7410	0.2244
YER019W	ISC1	YNL322C	KRE1	0.7386	0.0619
YGR208W	SER2	YKR080W	MTD1	0.7385	0.1927
YDR180W	SCC2	YGR184C	UBR1	0.7382	0.2783
YLR452C	SST2	YIL158W	AIM20	0.7377	-0.2455

YIL165C	YIL165C	YDL180W	YDL180W	0.7375	0.2433
YHR027C	RPN1	YPL234C	VMA11	0.7373	0.2895
YLR342W	FKS1	YJL158C	CIS3	0.7371	0.1931
YKR085C	MRPL20	YNR036C	MRPS12	0.7369	0.1767
YEL020W-A	TIM9	YER186C	YER186C	0.7368	0.3740
YER023W	PRO3	YEL058W	PCM1	0.7368	0.3413
YIL046W	MET30	YGR168C	YGR168C	0.7366	-0.1882
YAL009W	SPO7	YKL160W	ELF1	0.7362	0.2039
YBR137W	YBR137W	YNL022C	RCM1	0.7361	-0.2962
YNL242W	ATG2	YAL056W	GPB2	0.7358	0.2246
YLL061W	MMP1	YJL212C	OPT1	0.7357	0.3066
YHR104W	GRE3	YGR194C	XKS1	0.7354	0.3871
YKL073W	LHS1	YML079W	YML079W	0.7345	0.2287
YOR196C	LIP5	YGR012W	MCY1	0.7343	0.2608
YNR036C	MRPS12	YBR037C	SCO1	0.7339	0.2608
YBR147W	RTC2	YMR062C	ARG7	0.7332	0.3128
YML079W	YML079W	YPL058C	PDR12	0.7329	-0.2427
YJR073C	OPI3	YLR172C	DPH5	0.7324	0.2061
YFL016C	MDJ1	YHL007C	STE20	0.7318	0.2042
YOL130W	ALR1	YDR441C	APT2	0.7318	0.2883
YJL171C	TOH1	YER037W	PHM8	0.7314	0.2357
YHR209W	CRG1	YPL147W	PXA1	0.7313	0.2489
YPL144W	POC4	YPL031C	PHO85	0.7313	0.1757
YHR016C	YSC84	YML110C	COQ5	0.7295	0.2138
YNL138W	SRV2	YLR176C	RFX1	0.7284	0.2045
YGR194C	XKS1	YHR016C	YSC84	0.7278	0.2222
YOR264W	DSE3	YNL326C	PFA3	0.7278	0.3181
YKL110C	KTI12	YLR047C	FRE8	0.7265	-0.3308
YOR131C	YOR131C	YML013W	UBX2	0.7262	-0.2232
YKL073W	LHS1	YMR112C	MED11	0.7258	0.3299
YER046W	SPO73	YBR008C	FLR1	0.7255	-0.2047
YMR158W	MRPS8	YGR028W	MSP1	0.7253	0.3467
YMR209C	YMR209C	YDL150W	RPC53	0.7249	0.1485
YDL147W	RPN5	YFR004W	RPN11	0.7245	0.3425
YPL071C	YPL071C	YMR123W	PKR1	0.7244	-0.1852
YIL171W	YIL171W	YGR289C	MAL11	0.7242	0.2241
YFL047W	RGD2	YIL119C	RPI1	0.7239	0.0867
YBL030C	PET9	YGL017W	ATE1	0.7228	-0.1335
YPL226W	NEW1	YMR215W	GAS3	0.7226	0.2751
YER088C	DOT6	YGR158C	MTR3	0.7224	-0.2586
YBR071W	YBR071W	YIL171W	YIL171W	0.7212	-0.3180
YBR157C	ICS2	YLR248W	RCK2	0.7210	0.1884
YPL064C	CWC27	YPL234C	VMA11	0.7208	0.1966
YKL092C	BUD2	YER022W	SRB4	0.7206	0.2621
YNL322C	KRE1	YCL009C	ILV6	0.7195	0.2331
YOL062C	APM4	YOL092W	YPQ1	0.7194	-0.1913
YLR248W	RCK2	YLR443W	ECM7	0.7192	0.2899

YBL007C	SLA1	YBR225W	YBR225W	0.7189	0.2894
YDR064W	RPS13	YCR031C	RPS14a	0.7185	0.2025
YPL071C	YPL071C	YDL047W	SIT4	0.7185	-0.1441
YPL064C	CWC27	YOR092W	ECM3	0.7180	-0.1857
YLR056W	ERG3	YNL124W	NAF1	0.7175	0.2866
YLR059C	REX2	YHR023W	MYO1	0.7174	-0.2092
YDL155W	CLB3	YGR222W	PET54	0.7172	0.2034
YOR117W	RPT5	YGR209C	TRX2	0.7169	0.2616
YOR033C	EXO1	YLR368W	MDM30	0.7167	0.3625
YML016C	PPZ1	YGL257C	MNT2	0.7164	0.1445
YGL242C	YGL242C	YJR088C	EMC2	0.7161	0.1741
YBR249C	ARO4	YBR239C	ERT1	0.7150	0.3769
YMR010W	ANY1	YML038C	YMD8	0.7150	0.4639
YBR196C	PGI1	YER035W	EDC2	0.7149	-0.2122
YLR430W	SEN1	YDL164C	CDC9	0.7147	0.1233
YGR150C	CCM1	YOL092W	YPQ1	0.7133	-0.1964
YBR193C	MED8	YBL105C	PKC1	0.7132	0.1416
YJL126W	NIT2	YHR209W	CRG1	0.7131	0.1453
YJL158C	CIS3	YLR342W	FKS1	0.7125	0.2883
YDR441C	APT2	YHR115C	DMA1	0.7118	0.2099
YKL096W	CWP1	YNR036C	MRPS12	0.7113	0.1703
YBR229C	ROT2	YBL089W	AVT5	0.7112	0.4006
YFL018C	LPD1	YMR046C	YMR046C	0.7110	-0.1897
YLR442C	SIR3	YJL204C	RCY1	0.7105	0.2977
YMR158W	MRPS8	YKR085C	MRPL20	0.7104	0.1319
YGL051W	MST27	YCR041W	YCR041W	0.7103	0.1411
YJR088C	EMC2	YGL017W	ATE1	0.7101	-0.2910
YML007W	YAP1	YMR005W	TAF4	0.7101	0.2337
YDL047W	SIT4	YNL034W	YNL034W	0.7093	0.1919
YGL178W	MPT5	YGL051W	MST27	0.7092	-0.2408
YKL092C	BUD2	YOL018C	TLG2	0.7090	0.1276
YBL030C	PET9	YGL192W	IME4	0.7088	0.2430
YER150W	SPI1	YDR321W	ASP1	0.7087	-0.3207
YDL133W	SRF1	YGL176C	YGL176C	0.7082	0.1336
YHR034C	PIH1	YDR319C	YFT2	0.7080	0.2341
YEL058W	PCM1	YOL062C	APM4	0.7079	0.1997
YJR032W	CPR7	YML110C	COQ5	0.7075	0.2721
YIL066C	RNR3	YOR033C	EXO1	0.7070	0.2086
YEL056W	HAT2	YCR071C	IMG2	0.7065	-0.1954
YLR342W	FKS1	YIL061C	SNP1	0.7059	-0.3171
YJR088C	EMC2	YBR196C	PGI1	0.7055	0.2698
YKL184W	SPE1	YDR497C	ITR1	0.7055	-0.1843
YMR067C	UBX4	YHR027C	RPN1	0.7038	0.2533
YBL089W	AVT5	YBL105C	PKC1	0.7036	0.1885
YFL018C	LPD1	YDR178W	SDH4	0.7036	0.0364
YGL193C	YGL193C	YMR067C	UBX4	0.7033	-0.1302
YDL150W	RPC53	YGR289C	MAL11	0.7025	-0.4580

YIL066C	RNR3	YEL064C	AVT2	0.7023	0.1759
YKL096W	CWP1	YGR222W	PET54	0.7020	0.1464
YOL043C	NTG2	YOL018C	TLG2	0.7019	0.2352
YNL280C	ERG24	YPL183C	RTT10	0.7014	0.2640
YIL061C	SNP1	YDL171C	GLT1	0.7010	0.1968
YCR031C	RPS14a	YLR389C	STE23	0.7009	0.1974
YEL020W-A	TIM9	YMR158W	MRPS8	0.7008	0.2369
YDL111C	RRP42	YKL096W	CWP1	0.7005	0.2245
YKL160W	ELF1	YGR207C	CIR1	0.7003	0.0574
YLR443W	ECM7	YLR248W	RCK2	0.7001	0.1928
YML079W	YML079W	YEL020W-A	TIM9	0.6996	0.1706
YDR339C	FCF1	YDL002C	NHP10	0.6991	0.2711
YMR112C	MED11	YDR383C	NKP1	0.6986	0.1755
YDL135C	RDI1	YDL147W	RPN5	0.6983	0.1462
YOL094C	RFC4	YGR023W	MTL1	0.6981	-0.3513
YOR092W	ECM3	YPL064C	CWC27	0.6981	-0.1556
YBR161W	CSH1	YBR163W	EXO5	0.6973	0.2327
YGR289C	MAL11	YIL171W	YIL171W	0.6970	0.3557
YHR209W	CRG1	YEL058W	PCM1	0.6968	0.1311
YIL046W	MET30	YPL071C	YPL071C	0.6966	-0.0375
YOL043C	NTG2	YMR123W	PKR1	0.6963	-0.1930
YBR121C	GRS1	YOR117W	RPT5	0.6958	0.3100
YNL024C	EFM6	YLL035W	GRC3	0.6954	0.1724
YLR375W	STP3	YER035W	EDC2	0.6952	0.2722
YBL014C	RRN6	YBR163W	EXO5	0.6949	0.1824
YLR455W	PDP3	YFR037C	RSC8	0.6942	0.1053
YIR026C	YVH1	YER049W	TPA1	0.6931	0.2639
YMR215W	GAS3	YML072C	TCB3	0.6930	0.2828
YDL047W	SIT4	YKL116C	PRR1	0.6911	0.2875
YOL092W	YPQ1	YIL040W	APQ12	0.6911	0.2517
YJR088C	EMC2	YER144C	UBP5	0.6907	-0.3289
YGR207C	CIR1	YOR196C	LIP5	0.6903	0.2659
YGR207C	CIR1	YDR058C	TGL2	0.6898	0.2438
YNL242W	ATG2	YNL124W	NAF1	0.6892	0.1430
YBR157C	ICS2	YER046W	SPO73	0.6890	-0.1050
YHR023W	MYO1	YDL111C	RRP42	0.6890	-0.2005
YJL171C	TOH1	YGL178W	MPT5	0.6884	0.2106
YEL064C	AVT2	YDL211C	YDL211C	0.6879	0.1065
YBR168W	PEX32	YBR137W	YBR137W	0.6878	0.1551
YLR248W	RCK2	YGL169W	SUA5	0.6874	-0.3496
YML036W	CGI121	YML013W	UBX2	0.6874	0.2111
YGR028W	MSP1	YMR158W	MRPS8	0.6868	0.2241
YIL066C	RNR3	YGR209C	TRX2	0.6868	0.1875
YAL009W	SPO7	YIL171W	YIL171W	0.6865	-0.1515
YBR126C	TPS1	YBR137W	YBR137W	0.6860	0.3115
YEL068C	YEL068C	YBR067C	TIP1	0.6857	0.1372
YHR039C	MSC7	YLR056W	ERG3	0.6856	0.2388

YOL147C	PEX11	YEL058W	PCM1	0.6854	0.1947
YJR108W	ABM1	YOR088W	YOR088W	0.6852	0.2481
YGL178W	MPT5	YJL171C	TOH1	0.6850	0.1668
YJR086W	STE18	YJR025C	BNA1	0.6849	0.1558
YMR115W	MGR3	YJL204C	RCY1	0.6848	0.1290
YEL064C	AVT2	YPL071C	YPL071C	0.6842	0.2244
YDR367W	KEI1	YER144C	UBP5	0.6837	-0.1088
YNL135C	FPR1	YOR117W	RPT5	0.6834	0.0990
YMR158W	MRPS8	YGR150C	CCM1	0.6829	0.0520
YJR040W	GEF1	YOR088W	YOR088W	0.6824	0.1949
YDL180W	YDL180W	YGL193C	YGL193C	0.6808	0.2250
YDR513W	GRX2	YKL067W	YNK1	0.6808	0.2166
YJR097W	JJJ3	YDR530C	APA2	0.6805	-0.1697
YLR206W	ENT2	YOL018C	TLG2	0.6803	0.2390
YER182W	FMP10	YNR036C	MRPS12	0.6800	0.1834
YNL074C	MLF3	YOR101W	RAS1	0.6799	-0.2373
YKL116C	PRR1	YOL062C	APM4	0.6793	0.2321
YNL138W	SRV2	YDL132W	CDC53	0.6792	0.2053
YBL105C	PKC1	YBL020W	RFT1	0.6791	0.2372
YNL041C	COG6	YOL026C	MIM1	0.6791	0.2584
YCR071C	IMG2	YJL158C	CIS3	0.6790	0.2061
YFL056C	AAD6	YML091C	RPM2	0.6789	-0.0973
YNR012W	URK1	YNL022C	RCM1	0.6787	0.3274
YHR023W	MYO1	YLR059C	REX2	0.6784	-0.3874
YMR152W	YIM1	YML110C	COQ5	0.6778	0.3937
YER144C	UBP5	YGR149W	GPC1	0.6777	0.0770
YBR239C	ERT1	YBR154C	RPB5	0.6765	0.1892
YAL009W	SPO7	YER182W	FMP10	0.6763	0.0967
YGR222W	PET54	YNL024C	EFM6	0.6762	0.1377
YNL322C	KRE1	YOR264W	DSE3	0.6754	-0.0701
YAL009W	SPO7	YOR389W	YOR389W	0.6749	-0.0994
YHR023W	MYO1	YPL009C	RQC2	0.6740	0.1841
YER186C	YER186C	YEL020W-A	TIM9	0.6739	0.1954
YGR211W	ZPR1	YOR033C	EXO1	0.6739	0.3958
YGR184C	UBR1	YJR103W	URA8	0.6738	0.2814
YOR264W	DSE3	YJR073C	OPI3	0.6737	-0.2216
YDL002C	NHP10	YDR515W	SLF1	0.6734	0.2731
YJL204C	RCY1	YMR067C	UBX4	0.6730	0.2363
YBR239C	ERT1	YOR264W	DSE3	0.6729	-0.2136
YIL066C	RNR3	YCR083W	TRX3	0.6726	0.0927
YLR059C	REX2	YER116C	SLX8	0.6723	0.2553
YIR028W	DAL4	YNL278W	CAF120	0.6719	0.2230
YNL024C	EFM6	YNL046W	YNL046W	0.6717	0.1927
YGL195W	GCN1	YBL007C	SLA1	0.6710	0.2563
YDR383C	NKP1	YOR131C	YOR131C	0.6707	0.1939
YEL021W	URA3	YOR092W	ECM3	0.6707	-0.0307
YJL149W	DAS1	YHR209W	CRG1	0.6706	0.1788

YNL124W	NAF1	YFR042W	KEG1	0.6706	-0.3412
YEL076W-C	Unknown	YDL171C	GLT1	0.6703	0.1237
YNL322C	KRE1	YNL074C	MLF3	0.6696	0.2734
YLR231C	BNA5	YIL119C	RPI1	0.6691	0.1248
YDL097C	RPN6	YIL040W	APQ12	0.6690	0.3131
YMR152W	YIM1	YKL110C	KTI12	0.6682	-0.1727
YPL237W	SUI3	YOL097C	WRS1	0.6679	0.3638
YDR178W	SDH4	YOR142W	LSC1	0.6677	0.4261
YPL147W	PXA1	YGR006W	PRP18	0.6677	0.2064
YHR050W	SMF2	YHR027C	RPN1	0.6675	0.0781
YDL103C	QRI1	YBL089W	AVT5	0.6669	-0.2625
YBR154C	RPB5	YOL093W	TRM10	0.6662	0.3150
YIL046W	MET30	YGL176C	YGL176C	0.6660	-0.2128
YCL024W	KCC4	YOR144C	ELG1	0.6651	0.2400
YBR126C	TPS1	YHR104W	GRE3	0.6650	0.2037
YBR067C	TIP1	YOL026C	MIM1	0.6643	0.1716
YDR528W	HLR1	YPL071C	YPL071C	0.6643	0.1452
YGL195W	GCN1	YHR027C	RPN1	0.6642	0.2738
YGL192W	IME4	YLL038C	ENT4	0.6640	0.3058
YPL058C	PDR12	YKR075C	YKR075C	0.6633	0.0222
YMR010W	ANY1	YML048W	GSF2	0.6630	0.2460
YGL053W	PRM8	YIL082W	Unknown	0.6627	0.1322
YKL092C	BUD2	YML091C	RPM2	0.6627	-0.1689
YNR034W	SOL1	YAR028W	YAR028W	0.6627	-0.1398
YOL018C	TLG2	YGR152C	RSR1	0.6622	0.2105
YBR061C	TRM7	YBL014C	RRN6	0.6619	0.3110
YFR004W	RPN11	YDR528W	HLR1	0.6618	0.2017
YKL160W	ELF1	YJL140W	RPB4	0.6617	0.2797
YER150W	SPI1	YOL153C	YOL153C	0.6613	0.1838
YDL047W	SIT4	YJL183W	MNN11	0.6610	0.1544
YER037W	PHM8	YJR074W	MOG1	0.6610	0.2008
YML091C	RPM2	YFL018C	LPD1	0.6609	0.1738
YOR127W	RGA1	YGL257C	MNT2	0.6597	0.1126
YKR080W	MTD1	YML098W	TAF13	0.6596	0.1230
YNL024C	EFM6	YOR251C	TUM1	0.6590	0.0356
YOR264W	DSE3	YGL035C	MIG1	0.6586	0.2421
YHR016C	YSC84	YOL153C	YOL153C	0.6583	0.2584
YGR288W	MAL13	YDR034C	LYS14	0.6582	0.2823
YCL018W	LEU2	YHR209W	CRG1	0.6580	0.1587
YAR028W	YAR028W	YGL051W	MST27	0.6579	0.1500
YER088C	DOT6	YLR172C	DPH5	0.6579	-0.0445
YDL147W	RPN5	YDL133W	SRF1	0.6578	0.3890
YBR196C	PGI1	YJL171C	TOH1	0.6576	0.1459
YDR058C	TGL2	YOR080W	DIA2	0.6575	0.1445
YDR524C	AGE1	YDL047W	SIT4	0.6574	0.1290
YDL135C	RDI1	YOR051C	ETT1	0.6572	0.1883
YPL147W	PXA1	YIL165C	YIL165C	0.6571	-0.0200

YGR207C	CIR1	YKL160W	ELF1	0.6568	0.1877
YDR319C	YFT2	YGL025C	PGD1	0.6567	0.1729
YML013W	UBX2	YML036W	CGI121	0.6564	0.3624
YGR168C	YGR168C	YIL046W	MET30	0.6563	-0.0767
YOR371C	GPB1	YGR012W	MCY1	0.6563	0.3522
YER037W	PHM8	YML121W	GTR1	0.6558	0.2528
YFL047W	RGD2	YJR086W	STE18	0.6557	0.0894
YGL226C-A	OST5	YIL040W	APQ12	0.6557	0.0792
YJL036W	SNX4	YKL073W	LHS1	0.6554	0.1713
YKL073W	LHS1	YDL132W	CDC53	0.6550	0.2263
YJR103W	URA8	YPL147W	PXA1	0.6548	0.2444
YLR443W	ECM7	YOR054C	VHS3	0.6548	0.1629
YDR351W	SBE2	YFL056C	AAD6	0.6546	0.1910
YDL002C	NHP10	YER022W	SRB4	0.6545	0.1534
YKR075C	YKR075C	YPL058C	PDR12	0.6543	0.1511
YBR037C	SCO1	YGR150C	CCM1	0.6540	0.3712
YGR023W	MTL1	YBR229C	ROT2	0.6538	0.1480
YGL226C-A	OST5	YOL026C	MIM1	0.6535	0.2936
YIL040W	APQ12	YDL180W	YDL180W	0.6534	0.1467
YLR047C	FRE8	YGR258C	RAD2	0.6533	0.1949
YBR196C	PGI1	YBL040C	ERD2	0.6532	0.2473
YIL112W	HOS4	YIL046W	MET30	0.6532	0.0377
YER144C	UBP5	YOR080W	DIA2	0.6530	0.1921
YBL017C	PEP1	YBR161W	CSH1	0.6529	0.3228
YGL053W	PRM8	YGR258C	RAD2	0.6522	0.1322
YER088C	DOT6	YNL321W	VNX1	0.6518	0.2752
YNL074C	MLF3	YDL133W	SRF1	0.6517	-0.2923
YPL237W	SUI3	YLR435W	TSR2	0.6515	0.5150
YPR080W	TEF1	YGL025C	PGD1	0.6512	0.0172
YFR042W	KEG1	YKL110C	KTI12	0.6505	0.2995
YJR108W	ABM1	YHR054C	YHR054C	0.6500	-0.1201
YJR074W	MOG1	YKR051W	YKR051W	0.6498	0.1862
YMR005W	TAF4	YMR067C	UBX4	0.6492	0.1782
YFL018C	LPD1	YML091C	RPM2	0.6491	0.2822
YLL035W	GRC3	YNL024C	EFM6	0.6487	0.0987
YKR075C	YKR075C	YGL035C	MIG1	0.6486	0.0892
YPL234C	VMA11	YOL092W	YPQ1	0.6484	0.1763
YGL025C	PGD1	YER020W	GPA2	0.6483	0.1809
YPL234C	VMA11	YJR032W	CPR7	0.6482	0.1850
YFR004W	RPN11	YDL147W	RPN5	0.6480	0.0675
YGR001C	EFM5	YMR209C	YMR209C	0.6478	0.2048
YML036W	CGI121	YDL211C	YDL211C	0.6476	0.1905
YIL171W	YIL171W	YBR071W	YBR071W	0.6473	-0.1699
YDL147W	RPN5	YOL088C	MPD2	0.6471	0.2248
YFR042W	KEG1	YAL051W	OAF1	0.6465	0.1216
YHR039C	MSC7	YMR209C	YMR209C	0.6463	0.1496
YER144C	UBP5	YHR115C	DMA1	0.6462	0.1309

YAL051W	OAF1	YOL147C	PEX11	0.6459	0.1448
YFR042W	KEG1	YNL124W	NAF1	0.6458	-0.3216
YIL112W	HOS4	YDR351W	SBE2	0.6457	0.1991
YMR209C	YMR209C	YOL088C	MPD2	0.6457	0.1948
YJL171C	TOH1	YBR196C	PGI1	0.6456	0.1149
YBL079W	NUP170	YBL017C	PEP1	0.6453	0.2385
YJL154C	VPS35	YBL014C	RRN6	0.6451	0.1371
YAL056W	GPB2	YHR039C	MSC7	0.6449	-0.0920
YLR155C	ASP3-1	YHR054C	YHR054C	0.6448	0.0640
YNL124W	NAF1	YGR168C	YGR168C	0.6447	-0.2193
YPL234C	VMA11	YJL178C	ATG27	0.6443	0.2281
YFL016C	MDJ1	YFR013W	IOC3	0.6440	0.3159
YDR383C	NKP1	YGR157W	CHO2	0.6439	-0.1555
YLR342W	FKS1	YJL187C	SWE1	0.6432	0.2234
YLR375W	STP3	YDR515W	SLF1	0.6432	-0.1985
YBR229C	ROT2	YBR225W	YBR225W	0.6431	0.1472
YJR097W	JJJ3	YDL180W	YDL180W	0.6429	-0.2307
YKL067W	YNK1	YMR152W	YIM1	0.6428	0.0614
YBR193C	MED8	YBR168W	PEX32	0.6425	0.1364
YFR042W	KEG1	YKL092C	BUD2	0.6425	-0.1936
YFR042W	KEG1	YLL038C	ENT4	0.6423	-0.2740
YIR022W	SEC11	YIL043C	CBR1	0.6423	0.2883
YLR059C	REX2	YOL130W	ALR1	0.6420	0.1579
YIL133C	RPL16a	YDR367W	KEI1	0.6417	0.3441
YDR383C	NKP1	YDL147W	RPN5	0.6416	0.1194
YLR206W	ENT2	YDL217C	TIM22	0.6413	-0.2319
YGL226C-A	OST5	YGR209C	TRX2	0.6412	0.1927
YOR092W	ECM3	YMR192W	GYL1	0.6411	-0.1621
YBR126C	TPS1	YER150W	SPI1	0.6405	0.1972
YGL242C	YGL242C	YDL171C	GLT1	0.6402	-0.1150
YOR080W	DIA2	YOL081W	IRA2	0.6400	0.0059
YPL183C	RTT10	YLR287C	YLR287C	0.6400	0.3935
YML121W	GTR1	YML016C	PPZ1	0.6398	0.2980
YPL071C	YPL071C	YGL178W	MPT5	0.6390	-0.2048
YBL079W	NUP170	YBR163W	EXO5	0.6387	0.3056
YBR222C	PCS60	YOR101W	RAS1	0.6382	-0.1074
YJR073C	OPI3	YGR149W	GPC1	0.6382	0.1884
YNL035C	YNL035C	YNL041C	COG6	0.6382	0.1902
YCL018W	LEU2	YBR069C	TAT1	0.6379	-0.1957
YBR071W	YBR071W	YOR051C	ETT1	0.6376	-0.2687
YPL058C	PDR12	YOL104C	NDJ1	0.6376	0.1952
YIL165C	YIL165C	YDL171C	GLT1	0.6372	0.1828
YJR036C	HUL4	YGL004C	RPN14	0.6372	0.2389
YBR077C	SLM4	YBR163W	EXO5	0.6360	0.1545
YEL056W	HAT2	YOR196C	LIP5	0.6353	0.1762
YNL046W	YNL046W	YCR041W	YCR041W	0.6352	0.1254
YGL053W	PRM8	YLL024C	SSA2	0.6351	0.1764

YOL094C	RFC4	YIL142W	CCT2	0.6351	0.1836
YIL066C	RNR3	YMR209C	YMR209C	0.6345	-0.2056
YML072C	TCB3	YML004C	GLO1	0.6343	0.1101
YER088C	DOT6	YHR050W	SMF2	0.6337	0.0780
YLR257W	YLR257W	YGR028W	MSP1	0.6336	0.2665
YDL089W	NUR1	YDR528W	HLR1	0.6334	0.2462
YML048W	GSF2	YMR010W	ANY1	0.6333	0.3524
YOR142W	LSC1	YFR011C	MIC19	0.6332	0.2136
YGL035C	MIG1	YBR196C	PGI1	0.6331	-0.1491
YIL119C	RPI1	YER046W	SPO73	0.6329	-0.1306
YMR158W	MRPS8	YFR011C	MIC19	0.6324	0.0318
YBR193C	MED8	YGR001C	EFM5	0.6323	0.4244
YOL043C	NTG2	YBL020W	RFT1	0.6319	-0.1897
YLR046C	YLR046C	YJL051W	IRC8	0.6317	0.1562
YDR441C	APT2	YKL096W	CWP1	0.6313	-0.1455
YER186C	YER186C	YFL047W	RGD2	0.6313	0.1854
YBR077C	SLM4	YBR166C	TYR1	0.6312	0.2107
YLL038C	ENT4	YGL226W	MTC3	0.6305	0.0925
YBL105C	PKC1	YBR274W	CHK1	0.6303	0.1463
YBR077C	SLM4	YGR258C	RAD2	0.6303	-0.1951
YDR180W	SCC2	YDL164C	CDC9	0.6301	0.3062
YKL067W	YNK1	YHR104W	GRE3	0.6301	0.0685
YGR184C	UBR1	YOR088W	YOR088W	0.6299	0.0061
YIL040W	APQ12	YOR127W	RGA1	0.6295	-0.0147
YDL171C	GLT1	YIL061C	SNP1	0.6291	0.2151
YDL047W	SIT4	YLR176C	RFX1	0.6286	0.2533
YBL079W	NUP170	YBR222C	PCS60	0.6283	0.3103
YGR028W	MSP1	YCR071C	IMG2	0.6280	0.1828
YKR052C	MRS4	YLR426W	TDA5	0.6278	-0.2470
YDL167C	NRP1	YDR351W	SBE2	0.6277	0.3443
YNL041C	COG6	YKL092C	BUD2	0.6273	0.2210
YDL147W	RPN5	YDL135C	RDI1	0.6271	0.3597
YGL004C	RPN14	YJR036C	HUL4	0.6270	0.3686
YBR193C	MED8	YAR027W	UIP3	0.6268	0.2241
YOR043W	WHI2	YKR051W	YKR051W	0.6267	0.1075
YML036W	CGI121	YGL226W	MTC3	0.6261	0.2862
YLR231C	BNA5	YER020W	GPA2	0.6256	0.1733
YNL326C	PFA3	YNR034W	SOL1	0.6255	-0.1659
YDL135C	RDI1	YEL076W-C	Unknown	0.6252	0.2080
YDR515W	SLF1	YMR158W	MRPS8	0.6252	0.2149
YPR196W	YPR196W	YCL039W	GID7	0.6245	0.2113
YNL124W	NAF1	YOL092W	YPQ1	0.6239	0.0196
YDL150W	RPC53	YMR192W	GYL1	0.6237	0.2056
YEL058W	PCM1	YJR073C	OPI3	0.6237	0.1255
YER182W	FMP10	YKR085C	MRPL20	0.6237	0.1581
YDR524C	AGE1	YOL073C	DSC2	0.6236	-0.1458
YNL329C	PEX6	YBL040C	ERD2	0.6236	-0.0404

YPL071C	YPL071C	YPL009C	RQC2	0.6234	-0.1321
YBR193C	MED8	YDR383C	NKP1	0.6227	-0.1765
YDL164C	CDC9	YLR430W	SEN1	0.6223	0.3482
YBR067C	TIP1	YNL329C	PEX6	0.6222	0.2237
YNL074C	MLF3	YNL138W	SRV2	0.6219	0.1862
YJR103W	URA8	YBR222C	PCS60	0.6218	0.1541
YMR062C	ARG7	YIL166C	YIL166C	0.6215	0.2963
YOR043W	WHI2	YLR426W	TDA5	0.6214	0.1528
YPR022C	SDD4	YLR046C	YLR046C	0.6212	0.1133
YML096W	YML096W	YNL215W	IES2	0.6207	-0.1697
YOR117W	RPT5	YPL064C	CWC27	0.6205	-0.2772
YKR051W	YKR051W	YMR152W	YIM1	0.6204	0.1802
YDR319C	YFT2	YLR059C	REX2	0.6201	0.0327
YER037W	PHM8	YOR080W	DIA2	0.6191	0.1603
YJL212C	OPT1	YBR069C	TAT1	0.6191	0.1801
YBR126C	TPS1	YGR194C	XKS1	0.6178	0.3367
YER088C	DOT6	YJL158C	CIS3	0.6175	0.1357
YBR154C	RPB5	YDR339C	FCF1	0.6174	0.2911
YKL183W	LOT5	YOR088W	YOR088W	0.6170	0.2581
YBR225W	YBR225W	YBL007C	SLA1	0.6168	0.1433
YDR367W	KEI1	YGR289C	MAL11	0.6168	-0.0186
YLR389C	STE23	YMR005W	TAF4	0.6166	-0.1916
YDL135C	RDI1	YFR004W	RPN11	0.6165	0.1630
YMR154C	RIM13	YJR032W	CPR7	0.6159	-0.1693
YLR455W	PDP3	YJL149W	DAS1	0.6157	-0.1748
YOL094C	RFC4	YFR013W	IOC3	0.6152	0.3311
YLR180W	SAM1	YLR237W	THI7	0.6149	0.2497
YMR192W	GYL1	YDL150W	RPC53	0.6148	0.2184
YJR056C	YJR056C	YOR043W	WHI2	0.6147	0.0570
YLL061W	MMP1	YIL066C	RNR3	0.6140	-0.2677
YGL226C-A	OST5	YKL160W	ELF1	0.6137	0.0288
YPL234C	VMA11	YIL061C	SNP1	0.6137	-0.2239
YNL326C	PFA3	YOR264W	DSE3	0.6135	0.1607
YJR008W	MHO1	YKL073W	LHS1	0.6133	0.1966
YDL217C	TIM22	YNL329C	PEX6	0.6132	0.2626
YER022W	SRB4	YOR131C	YOR131C	0.6129	0.1049
YCR071C	IMG2	YGR028W	MSP1	0.6127	0.0720
YGR289C	MAL11	YNL034W	YNL034W	0.6124	-0.2196
YCR071C	IMG2	YJL178C	ATG27	0.6123	-0.2262
YMR123W	PKR1	YDR339C	FCF1	0.6119	0.1923
YNL041C	COG6	YJR086W	STE18	0.6119	0.2350
YEL056W	HAT2	YNL035C	YNL035C	0.6118	0.2105
YBR137W	YBR137W	YBR168W	PEX32	0.6117	0.1133
YGR209C	TRX2	YCL024W	KCC4	0.6117	-0.2417
YJR086W	STE18	YJL212C	OPT1	0.6114	-0.1968
YFR004W	RPN11	YPL009C	RQC2	0.6113	0.1108
YER023W	PRO3	YJR073C	OPI3	0.6112	0.1037

YOR127W	RGA1	YCR071C	IMG2	0.6110	0.1146
YML038C	YMD8	YBR061C	TRM7	0.6108	-0.1823
YFR047C	BNA6	YHR016C	YSC84	0.6106	0.1580
YOL147C	PEX11	YEL068C	YEL068C	0.6104	-0.0785
YPR080W	TEF1	YJL140W	RPB4	0.6104	-0.1446
YMR154C	RIM13	YLR426W	TDA5	0.6101	0.1428
YBR229C	ROT2	YBR274W	CHK1	0.6074	0.3573
YNL329C	PEX6	YKL019W	RAM2	0.6072	0.0931
YML036W	CGI121	YDL171C	GLT1	0.6071	-0.1517
YHR039C	MSC7	YBR087W	RFC5	0.6068	0.1304
YBR193C	MED8	YGR208W	SER2	0.6067	0.1741
YNL138W	SRV2	YGL207W	SPT16	0.6067	0.1827
YDL155W	CLB3	YML091C	RPM2	0.6065	0.0591
YNL215W	IES2	YLR180W	SAM1	0.6065	-0.0741
YER020W	GPA2	YGL208W	SIP2	0.6064	0.0745
YIL111W	COX5b	YBR157C	ICS2	0.6056	0.0584
YFR011C	MIC19	YDL002C	NHP10	0.6054	0.1984
YOL094C	RFC4	YOL097C	WRS1	0.6051	0.0480
YPL144W	POC4	YBR225W	YBR225W	0.6048	-0.1709
YDL097C	RPN6	YDR515W	SLF1	0.6047	0.0673
YOR054C	VHS3	YJL183W	MNN11	0.6046	-0.1166
YLL035W	GRC3	YGR211W	ZPR1	0.6042	0.2607
YJL212C	OPT1	YER088C	DOT6	0.6036	0.1789
YGL257C	MNT2	YML016C	PPZ1	0.6035	0.1119
YKL125W	RRN3	YOR144C	ELG1	0.6033	0.1655
YLR056W	ERG3	YKL125W	RRN3	0.6028	0.2210
YOR117W	RPT5	YML048W	GSF2	0.6028	0.0921
YDL171C	GLT1	YCL009C	ILV6	0.6025	0.0929
YPL233W	NSL1	YIL165C	YIL165C	0.6023	0.2501
YPL071C	YPL071C	YDR528W	HLR1	0.6023	0.0257
YDR515W	SLF1	YDL097C	RPN6	0.6017	0.1053
YOL089C	HAL9	YOL026C	MIM1	0.6017	-0.2540
YKL019W	RAM2	YML072C	TCB3	0.6011	0.0124
YEL064C	AVT2	YLR096W	KIN2	0.6010	-0.0967
YGR149W	GPC1	YOL094C	RFC4	0.6005	-0.2766
YJL171C	TOH1	YEL058W	PCM1	0.5997	0.1292
YAL056W	GPB2	YLR180W	SAM1	0.5996	-0.1810
YNR036C	MRPS12	YGL226W	MTC3	0.5996	0.3081
YLR452C	SST2	YIL082W	Unknown	0.5994	0.2119
YKL019W	RAM2	YLR426W	TDA5	0.5992	0.1614
YBR168W	PEX32	YBR163W	EXO5	0.5991	0.1247
YDR513W	GRX2	YLR258W	GSY2	0.5990	0.1894
YNL322C	KRE1	YGR150C	CCM1	0.5989	-0.1284
YHR050W	SMF2	YER088C	DOT6	0.5988	0.0984
YNL322C	KRE1	YML013W	UBX2	0.5988	0.1623
YDL103C	QRI1	YIL142W	CCT2	0.5987	0.2472
YJR088C	EMC2	YJR086W	STE18	0.5985	0.1231

YLR426W	TDA5	YKL019W	RAM2	0.5983	0.0315
YLR443W	ECM7	YHL048W	COS8	0.5976	-0.0320
YGL208W	SIP2	YBR037C	SCO1	0.5974	0.1685
YGR207C	CIR1	YGR208W	SER2	0.5973	0.1055
YML098W	TAF13	YNR034W	SOL1	0.5972	0.1756
YNL041C	COG6	YDL047W	SIT4	0.5970	-0.2084
YKL160W	ELF1	YAL009W	SPO7	0.5967	0.2224
YGR157W	CHO2	YDR383C	NKP1	0.5956	-0.2730
YKR080W	MTD1	YOL073C	DSC2	0.5954	-0.1493
YGR208W	SER2	YOR144C	ELG1	0.5953	0.0367
YMR152W	YIM1	YLR057W	MNL2	0.5951	-0.2303
YPL058C	PDR12	YLR176C	RFX1	0.5946	-0.1852
YFR004W	RPN11	YJL030W	MAD2	0.5945	0.1561
YNL322C	KRE1	YKL184W	SPE1	0.5944	0.1424
YDR420W	HKR1	YDL167C	NRP1	0.5940	0.2089
YIL089W	YIL089W	YGL193C	YGL193C	0.5931	-0.1873
YDL147W	RPN5	YPL234C	VMA11	0.5930	-0.1507
YIL171W	YIL171W	YAL009W	SPO7	0.5928	-0.2024
YDR515W	SLF1	YDR524C	AGE1	0.5925	0.3616
YBL040C	ERD2	YNL329C	PEX6	0.5923	-0.1930
YER150W	SPI1	YOL081W	IRA2	0.5923	0.3199
YML071C	COG8	YMR005W	TAF4	0.5915	0.1514
YEL064C	AVT2	YJR036C	HUL4	0.5913	0.1984
YFL018C	LPD1	YNR034W	SOL1	0.5913	-0.2009
YJR108W	ABM1	YEL076W-C	Unknown	0.5913	0.1351
YPL001W	HAT1	YDL135C	RDI1	0.5912	-0.0841
YHR016C	YSC84	YLR258W	GSY2	0.5911	0.3547
YNL215W	IES2	YGR207C	CIR1	0.5910	0.1710
YOR103C	OST2	YCL024W	KCC4	0.5909	-0.1170
YBR137W	YBR137W	YPL183C	RTT10	0.5906	-0.1684
YBR196C	PGI1	YEL071W	DLD3	0.5906	0.1251
YGR222W	PET54	YDL155W	CLB3	0.5905	0.1153
YMR192W	GYL1	YPL016W	SWI1	0.5902	0.2077
YOR117W	RPT5	YNL135C	FPR1	0.5902	0.1555
YBL089W	AVT5	YBL029W	YBL029W	0.5897	0.2045
YOL113W	SKM1	YLR047C	FRE8	0.5896	0.1803
YLR176C	RFX1	YKR080W	MTD1	0.5893	-0.1716
YML098W	TAF13	YKL116C	PRR1	0.5887	-0.0573
YGL053W	PRM8	YIL165C	YIL165C	0.5883	0.1473
YKL160W	ELF1	YDL164C	CDC9	0.5883	-0.1550
YML036W	CGI121	YER019W	ISC1	0.5883	0.1950
YGL195W	GCN1	YNL035C	YNL035C	0.5882	0.1885
YGR258C	RAD2	YAL051W	OAF1	0.5876	0.0404
YKL183W	LOT5	YLR330W	CHS5	0.5871	0.0286
YHL048W	COS8	YLR443W	ECM7	0.5862	-0.1705
YDL135C	RDI1	YHR054C	YHR054C	0.5856	-0.1759
YKL096W	CWP1	YJR074W	MOG1	0.5853	0.1497

YOR142W	LSC1	YOL073C	DSC2	0.5851	0.2264
YJR040W	GEF1	YNL138W	SRV2	0.5850	0.1045
YDR528W	HLR1	YDR530C	APA2	0.5849	0.2549
YER037W	PHM8	YLL061W	MMP1	0.5839	-0.1776
YLR430W	SEN1	YDR319C	YFT2	0.5839	-0.1793
YBR154C	RPB5	YBR249C	ARO4	0.5838	0.2219
YML038C	YMD8	YPL009C	RQC2	0.5837	0.1448
YOL147C	PEX11	YGL208W	SIP2	0.5837	0.1565
YJR108W	ABM1	YPL016W	SWI1	0.5835	-0.1822
YNR034W	SOL1	YFR011C	MIC19	0.5832	-0.1169
YLR442C	SIR3	YNL022C	RCM1	0.5829	0.1540
YBR163W	EXO5	YBR161W	CSH1	0.5824	0.2151
YOR080W	DIA2	YCL024W	KCC4	0.5816	-0.0897
YBL030C	PET9	YLL024C	SSA2	0.5814	-0.0616
YDL171C	GLT1	YKL110C	KTI12	0.5813	0.1635
YDR441C	APT2	YGR006W	PRP18	0.5813	-0.1812
YER023W	PRO3	YLR206W	ENT2	0.5813	-0.2002
YJR025C	BNA1	YJR086W	STE18	0.5810	0.1785
YMR209C	YMR209C	YIL066C	RNR3	0.5808	-0.1584
YOL062C	APM4	YJL036W	SNX4	0.5798	0.0376
YFR013W	IOC3	YNL041C	COG6	0.5796	0.2247
YDL132W	CDC53	YFL018C	LPD1	0.5795	0.1721
YBR071W	YBR071W	YER144C	UBP5	0.5794	0.2942
YBR229C	ROT2	YBR161W	CSH1	0.5790	0.1524
YKR075C	YKR075C	YER186C	YER186C	0.5790	-0.2055
YLR375W	STP3	YOL073C	DSC2	0.5790	0.3643
YDL211C	YDL211C	YEL064C	AVT2	0.5788	0.0703
YJR036C	HUL4	YOR048C	RAT1	0.5779	-0.0779
YBR067C	TIP1	YEL064C	AVT2	0.5778	0.0869
YGL176C	YGL176C	YNL124W	NAF1	0.5777	0.2004
YBR239C	ERT1	YER116C	SLX8	0.5776	-0.2397
YOL113W	SKM1	YER150W	SPI1	0.5776	-0.0911
YOR264W	DSE3	YHR115C	DMA1	0.5776	-0.2492
YNL322C	KRE1	YER116C	SLX8	0.5775	-0.1570
YHR027C	RPN1	YMR067C	UBX4	0.5772	0.2237
YJR073C	OPI3	YJR103W	URA8	0.5772	0.2145
YNL074C	MLF3	YLR426W	TDA5	0.5771	0.1760
YOR103C	OST2	YGL226C-A	OST5	0.5771	0.1450
YML072C	TCB3	YLR342W	FKS1	0.5768	0.0752
YER116C	SLX8	YPL058C	PDR12	0.5766	-0.0993
YGL226W	MTC3	YMR154C	RIM13	0.5765	0.1539
YAL009W	SPO7	YJR074W	MOG1	0.5758	0.0878
YGL053W	PRM8	YAR027W	UIP3	0.5756	0.1092
YOL062C	APM4	YMR010W	ANY1	0.5756	0.1829
YEL071W	DLD3	YHR050W	SMF2	0.5754	-0.2858
YGL193C	YGL193C	YOL113W	SKM1	0.5742	0.1033
YGR168C	YGR168C	YAL056W	GPB2	0.5741	-0.1508

YER046W	SPO73	YEL021W	URA3	0.5740	-0.2254
YPL071C	YPL071C	YEL064C	AVT2	0.5740	0.1774
YKR080W	MTD1	YGL193C	YGL193C	0.5739	-0.1701
YFR047C	BNA6	YML096W	YML096W	0.5737	-0.2609
YIR028W	DAL4	YLR368W	MDM30	0.5729	-0.2019
YCL024W	KCC4	YOR103C	OST2	0.5728	-0.1710
YIL082W	Unknown	YGL178W	MPT5	0.5724	0.2040
YBR069C	TAT1	YPL058C	PDR12	0.5723	-0.1473
YER019W	ISC1	YJL030W	MAD2	0.5715	0.2135
YGL017W	ATE1	YIL158W	AIM20	0.5715	-0.1138
YAL009W	SPO7	YBL020W	RFT1	0.5712	-0.1085
YPL001W	HAT1	YDL211C	YDL211C	0.5712	0.1429
YJL217W	REE1	YGL169W	SUA5	0.5711	-0.1749
YHR115C	DMA1	YOL062C	APM4	0.5710	0.1118
YFR013W	IOC3	YOL094C	RFC4	0.5709	0.0797
YOR117W	RPT5	YGR184C	UBR1	0.5709	0.0712
YOL026C	MIM1	YOL089C	HAL9	0.5708	-0.1025
YOR117W	RPT5	YHR027C	RPN1	0.5708	0.2055
YDL047W	SIT4	YNL041C	COG6	0.5707	-0.1769
YLR056W	ERG3	YHR039C	MSC7	0.5707	0.1011
YDL217C	TIM22	YKL096W	CWP1	0.5706	-0.1620
YBR249C	ARO4	YGR150C	CCM1	0.5703	-0.2929
YER116C	SLX8	YNL322C	KRE1	0.5703	-0.0521
YHR029C	YHI9	YGR207C	CIR1	0.5703	0.2164
YCR083W	TRX3	YIL066C	RNR3	0.5702	0.1501
YDL103C	QRI1	YLR375W	STP3	0.5702	-0.2021
YML016C	PPZ1	YGL178W	MPT5	0.5702	0.1354
YMR123W	PKR1	YPL071C	YPL071C	0.5702	-0.0960
YDR441C	APT2	YLL035W	GRC3	0.5697	0.2438
YKR080W	MTD1	YNL074C	MLF3	0.5697	0.1885
YOR131C	YOR131C	YJL030W	MAD2	0.5694	0.1350
YCL009C	ILV6	YLR426W	TDA5	0.5690	0.0965
YLR452C	SST2	YBR157C	ICS2	0.5687	-0.0734
YOR196C	LIP5	YHR063C	PAN5	0.5687	0.2375
YBL007C	SLA1	YBL017C	PEP1	0.5682	0.1778
YOL026C	MIM1	YLR455W	PDP3	0.5682	0.2264
YBR225W	YBR225W	YBR229C	ROT2	0.5681	0.1055
YLL035W	GRC3	YGR207C	CIR1	0.5674	-0.2209
YLR330W	CHS5	YOL018C	TLG2	0.5673	0.0630
YHR050W	SMF2	YOL147C	PEX11	0.5672	0.2769
YFL049W	SWP82	YPL064C	CWC27	0.5670	0.1924
YNL278W	CAF120	YML079W	YML079W	0.5669	-0.0196
YIL061C	SNP1	YDL097C	RPN6	0.5668	0.0854
YCR031C	RPS14a	YDR064W	RPS13	0.5666	0.2234
YAL009W	SPO7	YJL208C	NUC1	0.5662	-0.0388
YDR524C	AGE1	YDL217C	TIM22	0.5662	0.2062
YJR088C	EMC2	YER023W	PRO3	0.5659	0.0960

YLR176C	RFX1	YLR057W	MNL2	0.5653	-0.0923
YNL322C	KRE1	YOL073C	DSC2	0.5651	0.0655
YJR088C	EMC2	YGL242C	YGL242C	0.5649	0.3433
YAL009W	SPO7	YDL133W	SRF1	0.5646	0.1377
YFR037C	RSC8	YGL025C	PGD1	0.5645	0.1082
YLR248W	RCK2	YDL133W	SRF1	0.5643	0.1611
YDL171C	GLT1	YMR005W	TAF4	0.5641	-0.1863
YML096W	YML096W	YMR123W	PKR1	0.5639	0.1691
YDL133W	SRF1	YNL074C	MLF3	0.5637	-0.2261
YPL016W	SWI1	YAR028W	YAR028W	0.5636	-0.1726
YHL007C	STE20	YER049W	TPA1	0.5635	0.1073
YHR104W	GRE3	YOL153C	YOL153C	0.5633	0.1879
YNL035C	YNL035C	YAL051W	OAF1	0.5633	0.0642
YOL088C	MPD2	YJL030W	MAD2	0.5633	0.1445
YLL035W	GRC3	YLR443W	ECM7	0.5631	0.1925
YML004C	GLO1	YIL066C	RNR3	0.5630	0.0473
YML096W	YML096W	YOL130W	ALR1	0.5625	0.1565
YNL326C	PFA3	YML038C	YMD8	0.5624	0.0838
YCL026C-A	FRM2	YLR330W	CHS5	0.5623	-0.2236
YPL237W	SUI3	YKL160W	ELF1	0.5622	0.3659
YMR158W	MRPS8	YER182W	FMP10	0.5621	0.1136
YDL047W	SIT4	YPL071C	YPL071C	0.5619	-0.1820
YPL147W	PXA1	YJR103W	URA8	0.5607	0.0785
YGL178W	MPT5	YLL040C	VPS13	0.5606	0.1372
YKL021C	MAK11	YEL055C	POL5	0.5606	0.4058
YBL007C	SLA1	YBR087W	RFC5	0.5605	0.2358
YER046W	SPO73	YKL183W	LOT5	0.5604	-0.1483
YEL055C	POL5	YBR121C	GRS1	0.5603	0.2107
YOL081W	IRA2	YBL017C	PEP1	0.5600	0.2186
YGR012W	MCY1	YIL171W	YIL171W	0.5598	-0.0911
YHR043C	DOG2	YBR168W	PEX32	0.5597	0.0937
YLR342W	FKS1	YDL217C	TIM22	0.5596	-0.0615
YOR101W	RAS1	YPL183C	RTT10	0.5592	0.0507
YKL160W	ELF1	YIL166C	YIL166C	0.5590	-0.2240
YBR274W	CHK1	YBL029W	YBL029W	0.5588	0.1900
YGL004C	RPN14	YOL094C	RFC4	0.5588	0.1034
YGL207W	SPT16	YAL009W	SPO7	0.5586	-0.2555
YBR239C	ERT1	YOR142W	LSC1	0.5581	-0.1650
YBR239C	ERT1	YLR237W	THI7	0.5580	0.2883
YBR132C	AGP2	YGR149W	GPC1	0.5575	0.1756
YFL016C	MDJ1	YOR054C	VHS3	0.5574	0.1400
YMR062C	ARG7	YBR147W	RTC2	0.5573	0.2658
YNL046W	YNL046W	YER019W	ISC1	0.5573	0.0572
YBR193C	MED8	YGR158C	MTR3	0.5566	0.2032
YOL164W	BDS1	YAL009W	SPO7	0.5566	-0.1186
YCL039W	GID7	YDL089W	NUR1	0.5563	0.0245
YKL116C	PRR1	YGL169W	SUA5	0.5563	0.1550

YIR022W	SEC11	YOR103C	OST2	0.5560	0.1351
YOL062C	APM4	YAL056W	GPB2	0.5555	0.2557
YLR368W	MDM30	YNL138W	SRV2	0.5552	-0.0946
YNL024C	EFM6	YML072C	TCB3	0.5548	-0.0870
YOL113W	SKM1	YOL104C	NDJ1	0.5547	0.2217
YJL140W	RPB4	YGR150C	CCM1	0.5545	-0.1603
YPL234C	VMA11	YJL183W	MNN11	0.5542	0.1008
YAL051W	OAF1	YDL103C	QRI1	0.5538	0.1765
YPR196W	YPR196W	YNL034W	YNL034W	0.5536	0.2090
YNL135C	FPR1	YNR036C	MRPS12	0.5534	0.1095
YDL133W	SRF1	YIL111W	COX5b	0.5531	0.0809
YBR163W	EXO5	YBR077C	SLM4	0.5530	0.1962
YDR515W	SLF1	YDL002C	NHP10	0.5529	0.1269
YER144C	UBP5	YBR071W	YBR071W	0.5529	0.2042
YOR092W	ECM3	YGL017W	ATE1	0.5529	0.1514
YDR319C	YFT2	YOL089C	HAL9	0.5528	-0.2313
YHR115C	DMA1	YBR137W	YBR137W	0.5528	-0.0821
YJL212C	OPT1	YGL195W	GCN1	0.5528	-0.1526
YOL104C	NDJ1	YOL113W	SKM1	0.5526	0.1963
YML072C	TCB3	YJL051W	IRC8	0.5523	0.0930
YNR012W	URK1	YDL167C	NRP1	0.5522	0.2790
YMR010W	ANY1	YIL119C	RPI1	0.5517	0.1559
YGL201C	MCM6	YGR023W	MTL1	0.5516	0.1224
YLR330W	CHS5	YOR054C	VHS3	0.5516	0.0135
YDR513W	GRX2	YGR209C	TRX2	0.5515	0.1591
YGL025C	PGD1	YDR319C	YFT2	0.5515	0.1903
YDR058C	TGL2	YDR530C	APA2	0.5510	0.1946
YBR157C	ICS2	YGL226C-A	OST5	0.5507	0.1585
YJR074W	MOG1	YKL096W	CWP1	0.5505	0.1462
YBL017C	PEP1	YLR389C	STE23	0.5502	0.4165
YDR180W	SCC2	YLR181C	VTA1	0.5499	0.1193
YOL094C	RFC4	YLR172C	DPH5	0.5495	0.1012
YDR319C	YFT2	YGR012W	MCY1	0.5493	-0.1134
YNL135C	FPR1	YGR149W	GPC1	0.5493	-0.1249
YER020W	GPA2	YBR121C	GRS1	0.5490	-0.0942
YMR005W	TAF4	YLR046C	YLR046C	0.5490	-0.1446
YGL226W	MTC3	YML079W	YML079W	0.5489	0.2444
YMR154C	RIM13	YJL047C	RTT101	0.5484	0.1228
YOL018C	TLG2	YJL036W	SNX4	0.5477	0.0641
YIL166C	YIL166C	YNL034W	YNL034W	0.5476	0.1592
YOR054C	VHS3	YER020W	GPA2	0.5476	0.1382
YJR068W	RFC2	YNL035C	YNL035C	0.5474	0.1808
YHR063C	PAN5	YGR012W	MCY1	0.5473	0.1336
YKL092C	BUD2	YJL187C	SWE1	0.5469	0.1246
YDL133W	SRF1	YHR039C	MSC7	0.5466	-0.0922
YPL183C	RTT10	YOR101W	RAS1	0.5466	0.3543
YNL034W	YNL034W	YIL111W	COX5b	0.5465	-0.1222

YOR054C	VHS3	YIL009W	FAA3	0.5465	-0.2251
YER037W	PHM8	YAL056W	GPB2	0.5464	0.1844
YFL049W	SWP82	YKL116C	PRR1	0.5464	0.0671
YKL073W	LHS1	YJR008W	MHO1	0.5459	0.0070
YML071C	COG8	YOL018C	TLG2	0.5459	0.2014
YBL007C	SLA1	YBR008C	FLR1	0.5456	0.3189
YBR166C	TYR1	YNL041C	COG6	0.5456	0.2361
YMR046C	YMR046C	YDR178W	SDH4	0.5452	-0.1416
YOL147C	PEX11	YHR050W	SMF2	0.5449	0.1131
YKR080W	MTD1	YGR207C	CIR1	0.5446	0.0692
YNL326C	PFA3	YLR057W	MNL2	0.5446	0.1920
YDR178W	SDH4	YMR046C	YMR046C	0.5445	-0.2114
YLR368W	MDM30	YJL118W	YJL118W	0.5444	-0.0793
YJL154C	VPS35	YKL160W	ELF1	0.5443	-0.0973
YPL144W	POC4	YOL092W	YPQ1	0.5437	0.0688
YGR184C	UBR1	YEL056W	HAT2	0.5435	0.2162
YJL118W	YJL118W	YIR022W	SEC11	0.5433	0.0707
YGR152C	RSR1	YML071C	COG8	0.5432	0.3669
YDR441C	APT2	YDR319C	YFT2	0.5431	0.1372
YLR172C	DPH5	YDR420W	HKR1	0.5420	-0.3064
YKL184W	SPE1	YCL009C	ILV6	0.5419	0.0905
YJL187C	SWE1	YGR288W	MAL13	0.5418	-0.2085
YCR083W	TRX3	YNR036C	MRPS12	0.5413	0.0992
YGL176C	YGL176C	YBR166C	TYR1	0.5411	-0.2225
YJL158C	CIS3	YER088C	DOT6	0.5407	0.2728
YAL056W	GPB2	YGR149W	GPC1	0.5402	0.1885
YBR147W	RTC2	YJR008W	MHO1	0.5401	0.3489
YIL061C	SNP1	YPL064C	CWC27	0.5400	-0.1703
YJL187C	SWE1	YJR008W	MHO1	0.5400	-0.1421
YGR157W	CHO2	YDR058C	TGL2	0.5393	-0.0219
YGR209C	TRX2	YDR513W	GRX2	0.5393	0.1467
YPL012W	RRP12	YPL226W	NEW1	0.5393	0.4334
YHR023W	MYO1	YKL092C	BUD2	0.5392	0.0051
YJL217W	REE1	YFR013W	IOC3	0.5390	-0.1791
YGL176C	YGL176C	YDL133W	SRF1	0.5389	0.0930
YJL204C	RCY1	YDR420W	HKR1	0.5386	0.0439
YMR192W	GYL1	YOR092W	ECM3	0.5385	-0.0877
YBL089W	AVT5	YOL092W	YPQ1	0.5384	0.0770
YBR193C	MED8	YOR033C	EXO1	0.5382	0.1467
YHR033W	YHR033W	YOR144C	ELG1	0.5382	-0.1784
YLR052W	IES3	YJR073C	OPI3	0.5374	-0.0928
YOL093W	TRM10	YNL242W	ATG2	0.5371	-0.3496
YDL133W	SRF1	YLR248W	RCK2	0.5368	0.0298
YKL116C	PRR1	YGL193C	YGL193C	0.5368	-0.2062
YJR097W	JJJ3	YML091C	RPM2	0.5367	-0.0093
YOR048C	RAT1	YDR178W	SDH4	0.5367	-0.3231
YHR034C	PIH1	YGR149W	GPC1	0.5364	0.1331

YDR515W	SLF1	YLR375W	STP3	0.5361	-0.1724
YIR022W	SEC11	YJL118W	YJL118W	0.5360	0.2846
YDL103C	QRI1	YDL116W	NUP84	0.5358	0.4378
YPL233W	NSL1	YML048W	GSF2	0.5357	-0.1218
YOR117W	RPT5	YNL138W	SRV2	0.5356	0.0445
YKL092C	BUD2	YML036W	CGI121	0.5354	0.1314
YBR193C	MED8	YGL176C	YGL176C	0.5350	0.1755
YMR005W	TAF4	YJL212C	OPT1	0.5348	0.1737
YMR067C	UBX4	YNL046W	YNL046W	0.5347	-0.1677
YIL133C	RPL16a	YCR031C	RPS14a	0.5344	0.5321
YMR010W	ANY1	YNR034W	SOL1	0.5344	-0.1879
YMR010W	ANY1	YKR051W	YKR051W	0.5343	0.0927
YAL056W	GPB2	YPL058C	PDR12	0.5341	0.1915
YEL076W-C	Unknown	YBR071W	YBR071W	0.5341	-0.0843
YER020W	GPA2	YFL016C	MDJ1	0.5340	0.1713
YNL124W	NAF1	YER046W	SPO73	0.5324	-0.2594
YKL110C	KTI12	YJL208C	NUC1	0.5317	0.0898
YNL321W	VNX1	YJL212C	OPT1	0.5315	0.2902
YKL019W	RAM2	YEL056W	HAT2	0.5312	0.1384
YNL280C	ERG24	YDR321W	ASP1	0.5311	0.1278
YDR524C	AGE1	YLL040C	VPS13	0.5310	-0.1912
YLR287C	YLR287C	YLR435W	TSR2	0.5305	0.1775
YFR042W	KEG1	YDR528W	HLR1	0.5304	0.0646
YOR033C	EXO1	YGR211W	ZPR1	0.5303	0.1046
YBR239C	ERT1	YMR215W	GAS3	0.5296	-0.1763
YEL056W	HAT2	YHL007C	STE20	0.5293	0.1075
YNL322C	KRE1	YNL138W	SRV2	0.5286	0.1343
YPL031C	PHO85	YER020W	GPA2	0.5280	0.1379
YDR319C	YFT2	YIL040W	APQ12	0.5278	0.1475
YGL017W	ATE1	YOR117W	RPT5	0.5278	0.1634
YGR208W	SER2	YGL257C	MNT2	0.5276	0.2272
YIL165C	YIL165C	YPL147W	PXA1	0.5274	-0.0558
YJR086W	STE18	YDR524C	AGE1	0.5271	-0.2107
YBL105C	PKC1	YLL040C	VPS13	0.5270	0.1351
YKL067W	YNK1	YJR103W	URA8	0.5265	0.0445
YLL035W	GRC3	YLR452C	SST2	0.5265	0.1943
YDR515W	SLF1	YPL234C	VMA11	0.5264	-0.2420
YDR058C	TGL2	YDR383C	NKP1	0.5259	0.0388
YDL147W	RPN5	YGL242C	YGL242C	0.5256	0.0187
YGL004C	RPN14	YLR442C	SIR3	0.5256	0.2743
YGR023W	MTL1	YJL126W	NIT2	0.5256	-0.1959
YJL158C	CIS3	YBR166C	TYR1	0.5255	-0.0478
YDR420W	HKR1	YDL171C	GLT1	0.5252	0.1157
YCR041W	YCR041W	YGL035C	MIG1	0.5248	-0.1185
YMR067C	UBX4	YML048W	GSF2	0.5246	0.0449
YBR161W	CSH1	YGR207C	CIR1	0.5242	-0.1707
YGR157W	CHO2	YLR206W	ENT2	0.5240	0.3428

YOL026C	MIM1	YGL226C-A	OST5	0.5238	0.1884
YLR056W	ERG3	YBR061C	TRM7	0.5237	0.1188
YMR005W	TAF4	YOR103C	OST2	0.5234	-0.1475
YJR036C	HUL4	YGL178W	MPT5	0.5233	-0.2105
YDL164C	CDC9	YDL047W	SIT4	0.5230	0.1935
YMR154C	RIM13	YML121W	GTR1	0.5229	0.1857
YNL326C	PFA3	YGR258C	RAD2	0.5226	0.2047
YAL056W	GPB2	YIL040W	APQ12	0.5225	0.1749
YJR097W	JJJ3	YMR115W	MGR3	0.5224	0.2137
YBR222C	PCS60	YFR011C	MIC19	0.5222	-0.2519
YNR034W	SOL1	YKL096W	CWP1	0.5222	0.1866
YNL322C	KRE1	YLR375W	STP3	0.5220	0.2049
YPL233W	NSL1	YGL051W	MST27	0.5219	0.1436
YDL097C	RPN6	YDL103C	QRI1	0.5211	0.4151
YLL024C	SSA2	YGL226C-A	OST5	0.5211	0.0606
YML004C	GLO1	YML072C	TCB3	0.5211	0.0000
YOL153C	YOL153C	YLR449W	FPR4	0.5210	-0.3070
YPR080W	TEF1	YOL073C	DSC2	0.5208	-0.0773
YPR196W	YPR196W	YCL009C	ILV6	0.5208	-0.0354
YGL242C	YGL242C	YJL051W	IRC8	0.5204	-0.1121
YBL029W	YBL029W	YBR137W	YBR137W	0.5203	0.0694
YFR047C	BNA6	YLR442C	SIR3	0.5203	-0.2762
YGR258C	RAD2	YBR077C	SLM4	0.5202	-0.1614
YDR351W	SBE2	YLR389C	STE23	0.5201	0.0409
YJR084W	YJR084W	YJL218W	YJL218W	0.5200	-0.0201
YLR248W	RCK2	YML096W	YML096W	0.5199	-0.2554
YER186C	YER186C	YHR063C	PAN5	0.5194	-0.1607
YOR054C	VHS3	YFL016C	MDJ1	0.5194	0.2036
YFR013W	IOC3	YOL104C	NDJ1	0.5193	-0.0110
YIL119C	RPI1	YLR452C	SST2	0.5193	-0.1173
YOL097C	WRS1	YPL237W	SUI3	0.5192	0.3817
YGR149W	GPC1	YER144C	UBP5	0.5191	0.1243
YGR209C	TRX2	YOL073C	DSC2	0.5190	0.1939
YBR037C	SCO1	YOL062C	APM4	0.5188	0.1942
YOR251C	TUM1	YIR022W	SEC11	0.5184	0.3682
YLR287C	YLR287C	YER186C	YER186C	0.5183	0.1716
YOL018C	TLG2	YML004C	GLO1	0.5179	0.0607
YBL040C	ERD2	YCL024W	KCC4	0.5177	-0.1025
YGR207C	CIR1	YHL007C	STE20	0.5176	-0.2630
YBR274W	CHK1	YBR077C	SLM4	0.5173	0.1185
YNR012W	URK1	YNL280C	ERG24	0.5172	0.2477
YOL026C	MIM1	YML098W	TAF13	0.5170	0.2215
YOR117W	RPT5	YGL051W	MST27	0.5169	0.1865
YNL135C	FPR1	YLR330W	CHS5	0.5165	-0.1378
YEL064C	AVT2	YHR023W	MYO1	0.5162	0.0305
YEL021W	URA3	YFR004W	RPN11	0.5161	0.2854
YOL092W	YPQ1	YNL046W	YNL046W	0.5157	0.1156

YIL040W	APQ12	YBR121C	GRS1	0.5156	-0.0370
YHR027C	RPN1	YER020W	GPA2	0.5155	-0.1777
YLR059C	REX2	YDR319C	YFT2	0.5155	0.1741
YLR442C	SIR3	YLR057W	MNL2	0.5154	0.2358
YBR168W	PEX32	YOR127W	RGA1	0.5153	-0.2187
YDL002C	NHP10	YLL035W	GRC3	0.5150	-0.3234
YGR209C	TRX2	YGL226C-A	OST5	0.5150	0.0175
YGR184C	UBR1	YIL040W	APQ12	0.5144	-0.2220
YIL165C	YIL165C	YOR371C	GPB1	0.5144	0.1409
YDL180W	YDL180W	YOR043W	WHI2	0.5143	0.0065
YNL041C	COG6	YJL051W	IRC8	0.5140	-0.2957
YER150W	SPI1	YLR258W	GSY2	0.5138	0.1118
YDR515W	SLF1	YDR383C	NKP1	0.5132	0.2225
YOL062C	APM4	YDR134C	YDR134C	0.5132	-0.1515
YDR515W	SLF1	YOL092W	YPQ1	0.5130	-0.1888
YGR023W	MTL1	YLR342W	FKS1	0.5128	0.2744
YER019W	ISC1	YLR443W	ECM7	0.5127	0.1891
YML079W	YML079W	YMR152W	YIM1	0.5127	0.1028
YLL040C	VPS13	YHR016C	YSC84	0.5125	0.2988
YGL192W	IME4	YGL193C	YGL193C	0.5124	0.2363
YJL212C	OPT1	YMR005W	TAF4	0.5117	0.0856
YBR132C	AGP2	YNL321W	VNX1	0.5115	-0.0620
YDR319C	YFT2	YCL009C	ILV6	0.5114	0.0283
YBR161W	CSH1	YBL040C	ERD2	0.5113	0.2576
YNR034W	SOL1	YOR131C	YOR131C	0.5112	0.0926
YFL047W	RGD2	YER186C	YER186C	0.5110	0.1940
YKL184W	SPE1	YOL062C	APM4	0.5105	0.1315
YBR196C	PGI1	YOR101W	RAS1	0.5102	-0.1745
YBR071W	YBR071W	YNL024C	EFM6	0.5101	-0.1477
YJR040W	GEF1	YNL329C	PEX6	0.5101	0.1131
YLR059C	REX2	YER185W	PUG1	0.5101	-0.1536
YOL062C	APM4	YBR166C	TYR1	0.5101	0.1337
YDR178W	SDH4	YHR033W	YHR033W	0.5100	0.3133
YML071C	COG8	YNL215W	IES2	0.5100	0.1649
YGL053W	PRM8	YER185W	PUG1	0.5097	0.1767
YIL061C	SNP1	YBR229C	ROT2	0.5095	-0.0774
YJL154C	VPS35	YML036W	CGI121	0.5092	-0.1861
YDL089W	NUR1	YGL178W	MPT5	0.5091	0.2069
YLR206W	ENT2	YOR054C	VHS3	0.5086	0.0270
YOL081W	IRA2	YPL071C	YPL071C	0.5086	-0.2865
YER144C	UBP5	YDL133W	SRF1	0.5084	0.2158
YGL025C	PGD1	YPL031C	PHO85	0.5084	0.1761
YOR264W	DSE3	YGR149W	GPC1	0.5084	0.0180
YLR231C	BNA5	YHL048W	COS8	0.5083	0.2391
YDL164C	CDC9	YDL211C	YDL211C	0.5082	0.0889
YKL116C	PRR1	YGR157W	CHO2	0.5078	0.1062
YBR249C	ARO4	YOL097C	WRS1	0.5076	0.1167

YJL047C	RTT101	YER023W	PRO3	0.5076	-0.1320
YEL055C	POL5	YLR172C	DPH5	0.5075	0.3240
YDL135C	RDI1	YDL132W	CDC53	0.5074	0.0954
YIR022W	SEC11	YJL149W	DAS1	0.5070	-0.2825
YLR064W	PER33	YOR092W	ECM3	0.5070	0.2097
YKR051W	YKR051W	YEL064C	AVT2	0.5069	0.1930
YCL009C	ILV6	YLR052W	IES3	0.5068	-0.2125
YBL020W	RFT1	YBL105C	PKC1	0.5066	0.1220
YEL021W	URA3	YGL176C	YGL176C	0.5066	0.0794
YLR096W	KIN2	YEL064C	AVT2	0.5059	-0.0929
YNR034W	SOL1	YDR530C	APA2	0.5059	0.1551
YOR051C	ETT1	YBR071W	YBR071W	0.5056	-0.1127
YHR054C	YHR054C	YPL058C	PDR12	0.5055	0.1182
YDL132W	CDC53	YER116C	SLX8	0.5054	0.0165
YBR222C	PCS60	YOL093W	TRM10	0.5053	-0.2805
YOL062C	APM4	YOR196C	LIP5	0.5053	0.0842
YDL047W	SIT4	YDR524C	AGE1	0.5052	0.0888
YGL207W	SPT16	YGL208W	SIP2	0.5052	-0.1631
YGR150C	CCM1	YDL111C	RRP42	0.5050	-0.1069
YDL116W	NUP84	YGL176C	YGL176C	0.5049	0.3060
YOR144C	ELG1	YGR208W	SER2	0.5048	0.1779
YLR257W	YLR257W	YLR248W	RCK2	0.5043	0.2104
YGR289C	MAL11	YDL150W	RPC53	0.5038	-0.0975
YHR027C	RPN1	YLR430W	SEN1	0.5038	0.0189
YIL142W	CCT2	YLR172C	DPH5	0.5038	0.0017
YDR441C	APT2	YBR157C	ICS2	0.5036	-0.0121
YBR087W	RFC5	YBR274W	CHK1	0.5033	0.3093
YGL192W	IME4	YOL164W	BDS1	0.5033	0.2246
YPL147W	PXA1	YBL089W	AVT5	0.5030	0.0544
YJL149W	DAS1	YKL116C	PRR1	0.5027	0.0707
YMR115W	MGR3	YIL112W	HOS4	0.5027	-0.2042
YFR011C	MIC19	YLR046C	YLR046C	0.5024	-0.2087
YMR005W	TAF4	YNL138W	SRV2	0.5024	-0.1051
YPL233W	NSL1	YMR112C	MED11	0.5018	-0.3436
YBR163W	EXO5	YLR180W	SAM1	0.5016	0.2455
YCL018W	LEU2	YJR040W	GEF1	0.5016	0.0230
YBR163W	EXO5	YOR264W	DSE3	0.5013	0.2320
YLR176C	RFX1	YPL058C	PDR12	0.5013	-0.0861
YLR064W	PER33	YKL096W	CWP1	0.5012	0.0749
YJL217W	REE1	YOL089C	HAL9	0.5010	0.0416
YGL051W	MST27	YDL116W	NUP84	0.5008	0.2002
YDL155W	CLB3	YJL187C	SWE1	0.5004	0.0930
YBL014C	RRN6	YBL079W	NUP170	0.5002	0.1368
YGL257C	MNT2	YGL035C	MIG1	0.4997	-0.1049
YLR449W	FPR4	YJR068W	RFC2	0.4997	0.2779
YOR080W	DIA2	YER037W	PHM8	0.4997	0.1083
YOL062C	APM4	YLL035W	GRC3	0.4996	0.1431

YGR006W	PRP18	YIL061C	SNP1	0.4995	0.0828
YFL049W	SWP82	YLR258W	GSY2	0.4991	0.1552
YLL024C	SSA2	YKL116C	PRR1	0.4990	0.1278
YNL124W	NAF1	YJL208C	NUC1	0.4990	0.1163
YML038C	YMD8	YML036W	CGI121	0.4987	0.1982
YJL149W	DAS1	YML016C	PPZ1	0.4983	0.1593
YJR084W	YJR084W	YGR012W	MCY1	0.4982	0.1221
YBR225W	YBR225W	YIL158W	AIM20	0.4981	-0.1399
YER035W	EDC2	YJR032W	CPR7	0.4977	-0.1694
YGL051W	MST27	YBR157C	ICS2	0.4977	-0.1058
YJL178C	ATG27	YNL215W	IES2	0.4977	0.0219
YJR084W	YJR084W	YLR057W	MNL2	0.4976	-0.0949
YBR071W	YBR071W	YIL066C	RNR3	0.4975	-0.1884
YOL164W	BDS1	YDL111C	RRP42	0.4975	0.0967
YJL047C	RTT101	YLR430W	SEN1	0.4972	0.1148
YJL208C	NUC1	YNL124W	NAF1	0.4971	0.2499
YFL016C	MDJ1	YER020W	GPA2	0.4966	0.0954
YBR121C	GRS1	YIL046W	MET30	0.4963	-0.1224
YDR513W	GRX2	YML079W	YML079W	0.4963	0.2278
YJL051W	IRC8	YGL242C	YGL242C	0.4962	-0.1474
YDR321W	ASP1	YIL089W	YIL089W	0.4957	-0.0363
YEL058W	PCM1	YFL047W	RGD2	0.4957	0.1452
YIL089W	YIL089W	YER185W	PUG1	0.4954	-0.0091
YLR057W	MNL2	YNL074C	MLF3	0.4949	0.0221
YDL147W	RPN5	YBL030C	PET9	0.4948	-0.1737
YDL116W	NUP84	YNR034W	SOL1	0.4946	-0.1230
YHR115C	DMA1	YML013W	UBX2	0.4943	0.0576
YNL322C	KRE1	YMR115W	MGR3	0.4941	-0.0718
YDR420W	HKR1	YPL058C	PDR12	0.4935	0.0567
YLR059C	REX2	YNL321W	VNX1	0.4935	0.1328
YNL138W	SRV2	YNL074C	MLF3	0.4934	0.0815
YOR092W	ECM3	YLR342W	FKS1	0.4934	0.1481
YAL051W	OAF1	YJL149W	DAS1	0.4933	0.1126
YPL064C	CWC27	YJR032W	CPR7	0.4933	0.0204
YBL014C	RRN6	YLR452C	SST2	0.4930	-0.1680
YDR351W	SBE2	YNL041C	COG6	0.4930	0.0515
YDR497C	ITR1	YLR368W	MDM30	0.4930	-0.2964
YER186C	YER186C	YGR023W	MTL1	0.4930	-0.0881
YNL280C	ERG24	YER186C	YER186C	0.4924	-0.2413
YGL004C	RPN14	YGL169W	SUA5	0.4922	-0.1182
YDR058C	TGL2	YNL135C	FPR1	0.4920	-0.1448
YLR237W	THI7	YML016C	PPZ1	0.4916	-0.1012
YLR442C	SIR3	YJL030W	MAD2	0.4913	0.1199
YMR192W	GYL1	YPL147W	PXA1	0.4911	0.0004
YPL147W	PXA1	YGL176C	YGL176C	0.4911	0.1262
YBR229C	ROT2	YIL061C	SNP1	0.4910	-0.2013
YHR033W	YHR033W	YDR134C	YDR134C	0.4907	0.1371

YKL067W	YNK1	YML007W	YAP1	0.4907	-0.2082
YLR231C	BNA5	YBR239C	ERT1	0.4906	0.2178
YML048W	GSF2	YGL051W	MST27	0.4905	0.1952
YML072C	TCB3	YMR215W	GAS3	0.4903	0.1033
YJL204C	RCY1	YER088C	DOT6	0.4901	0.0823
YEL056W	HAT2	YOR088W	YOR088W	0.4900	0.2195
YBR147W	RTC2	YLL024C	SSA2	0.4899	-0.1580
YGR012W	MCY1	YOR048C	RAT1	0.4899	0.0255
YGR222W	PET54	YGR194C	XKS1	0.4899	0.0675
YOL130W	ALR1	YBR137W	YBR137W	0.4899	-0.0507
YLR368W	MDM30	YOL088C	MPD2	0.4898	0.0940
YFL018C	LPD1	YLL035W	GRC3	0.4896	-0.1763
YJL140W	RPB4	YDR351W	SBE2	0.4896	-0.0743
YDL002C	NHP10	YDL147W	RPN5	0.4894	0.1362
YGR194C	XKS1	YHR104W	GRE3	0.4888	0.2549
YHR039C	MSC7	YML007W	YAP1	0.4888	-0.1063
YEL058W	PCM1	YHR039C	MSC7	0.4887	0.0786
YMR154C	RIM13	YDR321W	ASP1	0.4886	-0.0024
YHR115C	DMA1	YBR008C	FLR1	0.4882	0.1426
YIL061C	SNP1	YOR371C	GPB1	0.4880	0.1487
YGR209C	TRX2	YOR103C	OST2	0.4879	0.1340
YKL110C	KTI12	YGR158C	MTR3	0.4878	0.1239
YLR231C	BNA5	YFL047W	RGD2	0.4874	0.0724
YGL176C	YGL176C	YDL116W	NUP84	0.4873	0.2244
YGR208W	SER2	YGR207C	CIR1	0.4872	0.1261
YMR112C	MED11	YPL031C	PHO85	0.4871	0.1533
YBR067C	TIP1	YLL028W	TPO1	0.4870	0.0032
YEL021W	URA3	YPL016W	SWI1	0.4868	0.0114
YCR041W	YCR041W	YGL051W	MST27	0.4866	0.1621
YLR368W	MDM30	YOL094C	RFC4	0.4866	-0.0906
YNL326C	PFA3	YOR371C	GPB1	0.4864	-0.1734
YGR023W	MTL1	YGL035C	MIG1	0.4862	0.1289
YHR054C	YHR054C	YMR115W	MGR3	0.4861	-0.0916
YBL017C	PEP1	YBL089W	AVT5	0.4860	0.0119
YJL154C	VPS35	YGR184C	UBR1	0.4859	0.0945
YJL154C	VPS35	YNL135C	FPR1	0.4858	-0.0096
YJL140W	RPB4	YLR057W	MNL2	0.4858	-0.1256
YKL125W	RRN3	YPL237W	SUI3	0.4856	0.0935
YGL051W	MST27	YPL226W	NEW1	0.4853	0.0560
YER150W	SPI1	YLR172C	DPH5	0.4852	-0.3567
YDL002C	NHP10	YGR168C	YGR168C	0.4849	0.1799
YBR008C	FLR1	YBR077C	SLM4	0.4848	0.1064
YOL088C	MPD2	YBR161W	CSH1	0.4846	0.1744
YKL019W	RAM2	YJL140W	RPB4	0.4844	0.0640
YGR001C	EFM5	YJL036W	SNX4	0.4840	-0.1273
YJL163C	YJL163C	YIL089W	YIL089W	0.4838	0.2997
YLR435W	TSR2	YKL125W	RRN3	0.4838	0.2965

YPL064C	CWC27	YPL071C	YPL071C	0.4838	0.1675
YGL025C	PGD1	YLR206W	ENT2	0.4837	0.0592
YHR034C	PIH1	YAR028W	YAR028W	0.4836	-0.1124
YMR215W	GAS3	YOR103C	OST2	0.4833	0.2453
YKL021C	MAK11	YOL093W	TRM10	0.4830	0.4156
YER046W	SPO73	YDL103C	QRI1	0.4828	-0.1193
YBR229C	ROT2	YBR157C	ICS2	0.4827	0.3228
YDR178W	SDH4	YFL018C	LPD1	0.4827	0.1754
YJL204C	RCY1	YML048W	GSF2	0.4826	0.1676
YDL171C	GLT1	YEL071W	DLD3	0.4825	0.0414
YEL064C	AVT2	YDR134C	YDR134C	0.4824	0.1046
YOR088W	YOR088W	YBL007C	SLA1	0.4822	0.0455
YML038C	YMD8	YML071C	COG8	0.4820	0.2255
YML036W	CGI121	YML038C	YMD8	0.4817	0.0928
YLR342W	FKS1	YJL204C	RCY1	0.4816	0.2848
YCR071C	IMG2	YBR037C	SCO1	0.4815	0.1316
YDL147W	RPN5	YDR383C	NKP1	0.4813	0.1547
YPL009C	RQC2	YPL071C	YPL071C	0.4813	-0.0882
YFL049W	SWP82	YER022W	SRB4	0.4809	0.1827
YJL208C	NUC1	YPL012W	RRP12	0.4808	0.2509
YOR043W	WHI2	YHR050W	SMF2	0.4808	0.1882
YML048W	GSF2	YLR180W	SAM1	0.4806	0.0665
YOL018C	TLG2	YER022W	SRB4	0.4803	0.1102
YLL028W	TPO1	YNL321W	VNX1	0.4800	0.0592
YDL135C	RDI1	YKR085C	MRPL20	0.4798	0.0851
YDR319C	YFT2	YPL144W	POC4	0.4796	0.1326
YLR206W	ENT2	YEL076W-C	Unknown	0.4795	-0.0869
YOL164W	BDS1	YNL329C	PEX6	0.4795	0.1241
YDL002C	NHP10	YDR383C	NKP1	0.4793	0.2022
YLR176C	RFX1	YOR103C	OST2	0.4791	0.1407
YBR037C	SCO1	YKR085C	MRPL20	0.4790	0.0805
YEL064C	AVT2	YIL061C	SNP1	0.4789	0.0571
YGR023W	MTL1	YLR206W	ENT2	0.4787	0.0959
YBR121C	GRS1	YGL195W	GCN1	0.4786	0.0334
YDR178W	SDH4	YLR248W	RCK2	0.4785	0.2907
YPL031C	PHO85	YKL019W	RAM2	0.4785	0.1007
YLR287C	YLR287C	YPL237W	SUI3	0.4780	0.3347
YBL029W	YBL029W	YOL147C	PEX11	0.4779	0.1234
YGL242C	YGL242C	YLR330W	CHS5	0.4779	0.1082
YJR025C	BNA1	YLR064W	PER33	0.4777	-0.1847
YPL234C	VMA11	YPL064C	CWC27	0.4777	0.1312
YOR054C	VHS3	YLR443W	ECM7	0.4776	0.1355
YML121W	GTR1	YBR121C	GRS1	0.4775	-0.1621
YPL234C	VMA11	YHR027C	RPN1	0.4773	0.1586
YER019W	ISC1	YKL019W	RAM2	0.4772	0.0903
YKL019W	RAM2	YML091C	RPM2	0.4772	-0.1036
YHR039C	MSC7	YLR375W	STP3	0.4770	-0.2313

YJR086W	STE18	YFL047W	RGD2	0.4770	0.1156
YBR071W	YBR071W	YDR178W	SDH4	0.4769	0.0435
YML038C	YMD8	YML098W	TAF13	0.4766	0.1096
YMR154C	RIM13	YLR449W	FPR4	0.4765	-0.1295
YGR152C	RSR1	YJR056C	YJR056C	0.4763	0.2036
YHR016C	YSC84	YOR088W	YOR088W	0.4763	0.3981
YDR180W	SCC2	YDL211C	YDL211C	0.4761	0.2529
YIL009W	FAA3	YDR420W	HKR1	0.4758	0.0000
YCL009C	ILV6	YDL171C	GLT1	0.4757	0.1444
YML048W	GSF2	YPL064C	CWC27	0.4754	-0.0839
YBL030C	PET9	YBL020W	RFT1	0.4749	0.0502
YLR155C	ASP3-1	YBR077C	SLM4	0.4749	0.0603
YJL149W	DAS1	YLL040C	VPS13	0.4747	0.1094
YFL056C	AAD6	YOR196C	LIP5	0.4746	0.1097
YLL024C	SSA2	YBR147W	RTC2	0.4744	-0.0254
YOR043W	WHI2	YLR052W	IES3	0.4744	0.1012
YGR150C	CCM1	YNL135C	FPR1	0.4738	-0.1302
YLR426W	TDA5	YER186C	YER186C	0.4738	0.0636
YDL211C	YDL211C	YDL164C	CDC9	0.4734	0.1313
YLR248W	RCK2	YKR051W	YKR051W	0.4734	0.0927
YHL007C	STE20	YDL171C	GLT1	0.4732	0.2057
YER046W	SPO73	YBR077C	SLM4	0.4729	0.1313
YEL064C	AVT2	YIL119C	RPI1	0.4728	-0.1463
YNL326C	PFA3	YCL024W	KCC4	0.4726	0.0136
YNL074C	MLF3	YNL329C	PEX6	0.4725	0.2361
YBR163W	EXO5	YBR249C	ARO4	0.4724	0.0522
YHR016C	YSC84	YGL208W	SIP2	0.4722	0.2489
YDR531W	CAB1	YBR249C	ARO4	0.4720	0.0021
YMR010W	ANY1	YER023W	PRO3	0.4720	0.1110
YDL135C	RDI1	YOR048C	RAT1	0.4718	0.0611
YBR069C	TAT1	YNR034W	SOL1	0.4717	-0.2066
YIL066C	RNR3	YHR054C	YHR054C	0.4717	-0.1467
YMR046C	YMR046C	YFL018C	LPD1	0.4717	-0.1513
YBR154C	RPB5	YBR239C	ERT1	0.4716	0.3342
YBR196C	PGI1	YGL035C	MIG1	0.4715	-0.0744
YOR117W	RPT5	YBR121C	GRS1	0.4715	0.0338
YGR222W	PET54	YGR150C	CCM1	0.4712	0.0796
YOL026C	MIM1	YGR209C	TRX2	0.4712	0.0075
YCR031C	RPS14a	YLR455W	PDP3	0.4711	0.1978
YCR071C	IMG2	YLL061W	MMP1	0.4711	-0.1210
YLR057W	MNL2	YGR023W	MTL1	0.4711	0.1931
YDR339C	FCF1	YIL133C	RPL16a	0.4710	0.2521
YLR389C	STE23	YJL140W	RPB4	0.4710	-0.0430
YML072C	TCB3	YBR126C	TPS1	0.4708	0.1111
YPL001W	HAT1	YBR067C	TIP1	0.4706	-0.2137
YEL055C	POL5	YOL097C	WRS1	0.4703	0.1613
YDR441C	APT2	YMR046C	YMR046C	0.4695	-0.1297

YER026C	CHO1	YKR075C	YKR075C	0.4693	-0.2264
YJR068W	RFC2	YJL126W	NIT2	0.4692	0.1310
YIL119C	RPI1	YDR441C	APT2	0.4690	-0.2220
YER020W	GPA2	YGL025C	PGD1	0.4689	0.2540
YHR033W	YHR033W	YKL183W	LOT5	0.4689	-0.1449
YML072C	TCB3	YOR117W	RPT5	0.4688	0.1328
YBR121C	GRS1	YML121W	GTR1	0.4686	-0.3563
YOL097C	WRS1	YER049W	TPA1	0.4685	0.2245
YOR196C	LIP5	YEL056W	HAT2	0.4685	0.1516
YJL171C	TOH1	YMR067C	UBX4	0.4683	-0.0671
YFR004W	RPN11	YOR048C	RAT1	0.4682	0.1552
YDR134C	YDR134C	YJL208C	NUC1	0.4681	-0.1379
YMR067C	UBX4	YGL193C	YGL193C	0.4677	-0.1432
YDL227C	Ho	YIL082W	Unknown	0.4673	0.0382
YFL016C	MDJ1	YGL176C	YGL176C	0.4673	-0.2047
YOR144C	ELG1	YJR056C	YJR056C	0.4673	0.2749
YER023W	PRO3	YMR010W	ANY1	0.4672	0.1904
YIR028W	DAL4	YBR166C	TYR1	0.4672	0.1460
YJR103W	URA8	YKL067W	YNK1	0.4672	0.1173
YNL322C	KRE1	YPL031C	PHO85	0.4672	0.0525
YNL321W	VNX1	YML072C	TCB3	0.4672	0.2035
YDL111C	RRP42	YMR209C	YMR209C	0.4670	0.1929
YDR528W	HLR1	YOR264W	DSE3	0.4667	-0.0999
YOL164W	BDS1	YGL192W	IME4	0.4667	0.0219
YCL024W	KCC4	YFL016C	MDJ1	0.4666	0.2349
YKR075C	YKR075C	YER026C	CHO1	0.4665	-0.0851
YGR006W	PRP18	YOR371C	GPB1	0.4662	0.1142
YNL215W	IES2	YOL093W	TRM10	0.4662	0.0070
YNL138W	SRV2	YNL322C	KRE1	0.4661	0.1060
YOL147C	PEX11	YBR069C	TAT1	0.4661	0.0350
YPL058C	PDR12	YER116C	SLX8	0.4661	-0.1236
YOL062C	APM4	YKL184W	SPE1	0.4660	0.0524
YJR086W	STE18	YNL041C	COG6	0.4656	0.1902
YDL180W	YDL180W	YOL113W	SKM1	0.4654	0.0024
YLR330W	CHS5	YNL280C	ERG24	0.4654	-0.1114
YMR209C	YMR209C	YGR001C	EFM5	0.4649	0.1154
YLR287C	YLR287C	YIR026C	YVH1	0.4645	0.3219
YPR015C	YPR015C	YBR087W	RFC5	0.4645	-0.0845
YGR222W	PET54	YGL051W	MST27	0.4644	0.3078
YKL183W	LOT5	YPL234C	VMA11	0.4644	0.0761
YNL124W	NAF1	YAR027W	UIP3	0.4644	-0.1708
YOL088C	MPD2	YER116C	SLX8	0.4642	0.2254
YOR196C	LIP5	YOL062C	APM4	0.4641	0.1886
YPL233W	NSL1	YJL149W	DAS1	0.4641	0.1241
YPL016W	SWI1	YNL135C	FPR1	0.4640	-0.1587
YDL132W	CDC53	YBR077C	SLM4	0.4638	0.0666
YOL062C	APM4	YKR051W	YKR051W	0.4638	0.1604

YAR028W	YAR028W	YBR196C	PGI1	0.4637	0.1514
YOR033C	EXO1	YER022W	SRB4	0.4634	0.1223
YJR088C	EMC2	YNL138W	SRV2	0.4633	0.1807
YLR057W	MNL2	YBR069C	TAT1	0.4633	0.1395
YPL071C	YPL071C	YJL171C	TOH1	0.4632	-0.1567
YJL140W	RPB4	YKL160W	ELF1	0.4631	0.0142
YNL215W	IES2	YML007W	YAP1	0.4630	0.1293
YKL092C	BUD2	YOL089C	HAL9	0.4628	0.1251
YOL147C	PEX11	YGL025C	PGD1	0.4628	-0.0008
YBR249C	ARO4	YBR166C	TYR1	0.4627	0.2528
YFR047C	BNA6	YER150W	SPI1	0.4627	0.0548
YGL257C	MNT2	YOR127W	RGA1	0.4627	0.0341
YNL329C	PEX6	YDL217C	TIM22	0.4627	0.1006
YJR056C	YJR056C	YPL183C	RTT10	0.4626	0.0052
YML079W	YML079W	YLR047C	FRE8	0.4624	-0.1392
YMR152W	YIM1	YML079W	YML079W	0.4624	0.0412
YLL035W	GRC3	YOL062C	APM4	0.4621	0.1075
YDR528W	HLR1	YLR237W	THI7	0.4620	0.2066
YHR104W	GRE3	YPL031C	PHO85	0.4620	0.2040
YBR121C	GRS1	YBL079W	NUP170	0.4617	0.3119
YGL025C	PGD1	YAR028W	YAR028W	0.4615	-0.0950
YJL030W	MAD2	YMR112C	MED11	0.4614	0.2930
YLR389C	STE23	YLR342W	FKS1	0.4614	0.0852
YKL116C	PRR1	YJL149W	DAS1	0.4613	0.1128
YBR137W	YBR137W	YBR077C	SLM4	0.4612	0.1987
YLR330W	CHS5	YPL071C	YPL071C	0.4612	-0.0778
YDL133W	SRF1	YAL009W	SPO7	0.4610	0.0750
YOL164W	BDS1	YLR389C	STE23	0.4610	0.0297
YMR005W	TAF4	YDL171C	GLT1	0.4609	-0.0477
YER182W	FMP10	YGL195W	GCN1	0.4606	-0.1792
YOL088C	MPD2	YER022W	SRB4	0.4605	0.1470
YGL051W	MST27	YOR251C	TUM1	0.4604	-0.0714
YBL020W	RFT1	YBL030C	PET9	0.4603	0.0736
YBR008C	FLR1	YBR222C	PCS60	0.4603	0.1606
YCL026C-A	FRM2	YDL002C	NHP10	0.4603	0.2834
YDL217C	TIM22	YKL183W	LOT5	0.4602	0.1131
YCR041W	YCR041W	YJR103W	URA8	0.4601	0.0418
YKL096W	CWP1	YDL116W	NUP84	0.4601	-0.1566
YNL024C	EFM6	YDR497C	ITR1	0.4601	0.1218
YKL116C	PRR1	YML038C	YMD8	0.4600	-0.2373
YOR080W	DIA2	YDR058C	TGL2	0.4599	0.0474
YMR115W	MGR3	YOR127W	RGA1	0.4596	0.1647
YOR033C	EXO1	YPL226W	NEW1	0.4595	0.1107
YOL089C	HAL9	YJL047C	RTT101	0.4594	0.1656
YOL088C	MPD2	YOL094C	RFC4	0.4590	0.0445
YJL126W	NIT2	YLR368W	MDM30	0.4589	-0.2336
YFL016C	MDJ1	YGL169W	SUA5	0.4588	0.1487

YOR092W	ECM3	YGR207C	CIR1	0.4586	-0.1243
YBL014C	RRN6	YBR061C	TRM7	0.4584	0.1011
YGL025C	PGD1	YNL321W	VNX1	0.4580	0.0346
YML036W	CGI121	YMR123W	PKR1	0.4580	0.1593
YDL135C	RDI1	YPL001W	HAT1	0.4579	-0.1951
YHR063C	PAN5	YHR023W	MYO1	0.4579	0.0855
YDR530C	APA2	YDR528W	HLR1	0.4576	0.0738
YDR058C	TGL2	YGR207C	CIR1	0.4575	0.1047
YLR342W	FKS1	YOL081W	IRA2	0.4575	0.0487
YER019W	ISC1	YDL167C	NRP1	0.4574	-0.0674
YER049W	TPA1	YKL021C	MAK11	0.4573	0.2983
YFL018C	LPD1	YNL138W	SRV2	0.4573	0.1625
YJR084W	YJR084W	YGR001C	EFM5	0.4573	-0.1221
YIL112W	HOS4	YIL111W	COX5b	0.4572	0.1883
YGL051W	MST27	YHR054C	YHR054C	0.4571	-0.0103
YMR158W	MRPS8	YEL020W-A	TIM9	0.4570	0.0951
YAL056W	GPB2	YGR168C	YGR168C	0.4569	-0.1681
YJR068W	RFC2	YLR449W	FPR4	0.4568	0.0725
YLL028W	TPO1	YER088C	DOT6	0.4568	0.0735
YDL164C	CDC9	YFL016C	MDJ1	0.4567	0.0629
YMR067C	UBX4	YBR077C	SLM4	0.4566	-0.2164
YGR150C	CCM1	YGR012W	MCY1	0.4560	0.1033
YDL167C	NRP1	YPL012W	RRP12	0.4559	0.1193
YDR513W	GRX2	YOR048C	RAT1	0.4555	-0.2508
YLR287C	YLR287C	YKL125W	RRN3	0.4554	0.2646
YFR047C	BNA6	YLR096W	KIN2	0.4553	-0.2893
YMR154C	RIM13	YGL226W	MTC3	0.4553	0.0001
YGR149W	GPC1	YLR258W	GSY2	0.4552	0.0146
YIL112W	HOS4	YGL017W	ATE1	0.4552	0.1384
YLL061W	MMP1	YOL093W	TRM10	0.4550	0.0496
YBR137W	YBR137W	YPL012W	RRP12	0.4546	-0.0867
YJL187C	SWE1	YHR050W	SMF2	0.4543	0.0811
YJR097W	JJJ3	YLR047C	FRE8	0.4541	-0.1314
YLL040C	VPS13	YDR524C	AGE1	0.4541	-0.1832
YML038C	YMD8	YEL064C	AVT2	0.4541	0.1379
YDR038C	ENA5	YIL009W	FAA3	0.4540	0.0209
YHR023W	MYO1	YOR264W	DSE3	0.4540	0.0010
YCL024W	KCC4	YOR131C	YOR131C	0.4539	0.2369
YGL051W	MST27	YMR112C	MED11	0.4539	-0.1861
YBR132C	AGP2	YIL009W	FAA3	0.4537	0.1667
YDR144C	MKC7	YDR367W	KEI1	0.4537	0.1795
YFL049W	SWP82	YER088C	DOT6	0.4537	-0.0375
YBR222C	PCS60	YNL138W	SRV2	0.4536	0.1556
YML013W	UBX2	YHR115C	DMA1	0.4536	0.1641
YMR062C	ARG7	YOR196C	LIP5	0.4536	0.2447
YNL326C	PFA3	YJL126W	NIT2	0.4536	-0.1030
YNL022C	RCM1	YNR012W	URK1	0.4535	0.3673

YGL242C	YGL242C	YJL030W	MAD2	0.4532	0.1122
YDR497C	ITR1	YLR181C	VTA1	0.4531	-0.0013
YGL193C	YGL193C	YGL192W	IME4	0.4527	0.1086
YIL066C	RNR3	YLL061W	MMP1	0.4526	-0.1305
YOL092W	YPQ1	YBL089W	AVT5	0.4526	0.1871
YOL094C	RFC4	YMR005W	TAF4	0.4524	0.0625
YGL226W	MTC3	YNR036C	MRPS12	0.4523	0.0921
YLR052W	IES3	YLR046C	YLR046C	0.4522	-0.1409
YNL135C	FPR1	YGR150C	CCM1	0.4522	-0.1812
YFR013W	IOC3	YER026C	CHO1	0.4520	0.1328
YGR207C	CIR1	YJR068W	RFC2	0.4516	0.0032
YLR231C	BNA5	YGL226W	MTC3	0.4516	0.0880
YEL056W	HAT2	YOL147C	PEX11	0.4511	-0.0685
YEL068C	YEL068C	YPL001W	HAT1	0.4510	-0.0667
YFL016C	MDJ1	YAL009W	SPO7	0.4510	-0.1638
YFR011C	MIC19	YJR086W	STE18	0.4509	0.0586
YGR184C	UBR1	YEL068C	YEL068C	0.4506	-0.1133
YNL329C	PEX6	YOR264W	DSE3	0.4505	0.0962
YEL068C	YEL068C	YJL149W	DAS1	0.4503	-0.0782
YLR452C	SST2	YHR050W	SMF2	0.4503	-0.1975
YLR056W	ERG3	YGR289C	MAL11	0.4501	-0.0435
YGL226C-A	OST5	YDR420W	HKR1	0.4499	-0.0052
YNL326C	PFA3	YDR420W	HKR1	0.4498	0.0313
YJR040W	GEF1	YOL073C	DSC2	0.4497	0.1498
YLR257W	YLR257W	YGR149W	GPC1	0.4497	0.2746
YGR158C	MTR3	YJL126W	NIT2	0.4496	0.1986
YPL009C	RQC2	YFR004W	RPN11	0.4495	0.1543
YDR513W	GRX2	YMR152W	YIM1	0.4493	0.2068
YER049W	TPA1	YOL097C	WRS1	0.4493	0.0442
YMR005W	TAF4	YER150W	SPI1	0.4493	0.0382
YNL321W	VNX1	YLL024C	SSA2	0.4492	-0.1644
YGR028W	MSP1	YIR026C	YVH1	0.4491	-0.1020
YBR193C	MED8	YOR371C	GPB1	0.4490	0.1376
YMR158W	MRPS8	YBR037C	SCO1	0.4489	0.0483
YER046W	SPO73	YJL140W	RPB4	0.4488	0.0365
YJL058C	BIT61	YOL113W	SKM1	0.4488	0.0311
YLR237W	THI7	YHR043C	DOG2	0.4486	-0.1340
YGR001C	EFM5	YDL167C	NRP1	0.4484	0.0673
YBR008C	FLR1	YBR225W	YBR225W	0.4482	0.0789
YHR043C	DOG2	YLR248W	RCK2	0.4482	0.0442
YPL147W	PXA1	YKL073W	LHS1	0.4481	-0.0788
YJR068W	RFC2	YLR287C	YLR287C	0.4477	0.1673
YER023W	PRO3	YLR452C	SST2	0.4475	0.1623
YPR015C	YPR015C	YHR054C	YHR054C	0.4470	0.0246
YPL183C	RTT10	YER049W	TPA1	0.4465	0.2982
YDL089W	NUR1	YDL047W	SIT4	0.4462	0.0921
YMR209C	YMR209C	YLR237W	THI7	0.4462	-0.0323

YOR144C	ELG1	YHR033W	YHR033W	0.4462	-0.0344
YDL089W	NUR1	YIL015C-A	YIL015C-A	0.4461	0.0172
YMR067C	UBX4	YMR005W	TAF4	0.4459	0.2027
YLR064W	PER33	YJL178C	ATG27	0.4458	0.0260
YDL132W	CDC53	YDL097C	RPN6	0.4457	0.1267
YLR368W	MDM30	YBL017C	PEP1	0.4454	0.1091
YOR088W	YOR088W	YGL193C	YGL193C	0.4451	0.0864
YFL056C	AAD6	YLR452C	SST2	0.4450	-0.0926
YAR028W	YAR028W	YJL036W	SNX4	0.4447	0.0948
YIL119C	RPI1	YOR101W	RAS1	0.4446	-0.0265
YOR080W	DIA2	YHR016C	YSC84	0.4446	0.0347
YGL004C	RPN14	YBR137W	YBR137W	0.4444	0.0911
YGR157W	CHO2	YEL058W	PCM1	0.4444	0.0721
YGR207C	CIR1	YDL111C	RRP42	0.4443	-0.1792
YJR097W	JJJ3	YNR012W	URK1	0.4443	0.0220
YDL116W	NUP84	YBR222C	PCS60	0.4442	-0.1487
YBR067C	TIP1	YER020W	GPA2	0.4441	-0.0414
YBL030C	PET9	YJL140W	RPB4	0.4440	-0.0613
YBR077C	SLM4	YBL007C	SLA1	0.4440	0.1248
YGL176C	YGL176C	YNL322C	KRE1	0.4440	-0.1493
YLR426W	TDA5	YNL074C	MLF3	0.4439	0.0495
YML091C	RPM2	YAL009W	SPO7	0.4439	-0.1350
YGL193C	YGL193C	YLR455W	PDP3	0.4433	0.1589
YJR056C	YJR056C	YLR176C	RFX1	0.4431	0.0601
YNR036C	MRPS12	YGR157W	CHO2	0.4431	-0.2271
YER020W	GPA2	YNR034W	SOL1	0.4430	0.2378
YML096W	YML096W	YPL183C	RTT10	0.4428	0.0092
YEL068C	YEL068C	YML079W	YML079W	0.4426	0.0981
YKL183W	LOT5	YOR051C	ETT1	0.4426	0.2256
YDR383C	NKP1	YFR037C	RSC8	0.4425	0.0949
YFL016C	MDJ1	YDR441C	APT2	0.4425	0.1915
YBR008C	FLR1	YER046W	SPO73	0.4424	-0.1586
YML110C	COQ5	YDR531W	CAB1	0.4422	-0.0777
YDL111C	RRP42	YHR023W	MYO1	0.4418	-0.0652
YNL035C	YNL035C	YHR027C	RPN1	0.4417	0.0286
YML016C	PPZ1	YNL074C	MLF3	0.4416	0.1092
YGR258C	RAD2	YBR061C	TRM7	0.4415	-0.0806
YIL089W	YIL089W	YGR023W	MTL1	0.4415	0.0759
YLR443W	ECM7	YOL026C	MIM1	0.4415	0.2167
YNL135C	FPR1	YLR059C	REX2	0.4414	0.1069
YJR097W	JJJ3	YGR288W	MAL13	0.4413	-0.1285
YNR034W	SOL1	YDL116W	NUP84	0.4413	-0.0062
YEL068C	YEL068C	YJL030W	MAD2	0.4412	0.1110
YFL047W	RGD2	YGL017W	ATE1	0.4412	0.0888
YEL056W	HAT2	YOL097C	WRS1	0.4410	0.1214
YLR287C	YLR287C	YPL183C	RTT10	0.4407	0.2435
YGL178W	MPT5	YPL071C	YPL071C	0.4406	-0.0043

YHR209W	CRG1	YGL017W	ATE1	0.4406	-0.1517
YCL039W	GID7	YLR442C	SIR3	0.4401	-0.0625
YJL140W	RPB4	YPL144W	POC4	0.4399	0.0853
YGR012W	MCY1	YOR196C	LIP5	0.4398	0.0527
YKL110C	KTI12	YDL111C	RRP42	0.4398	0.2721
YOR043W	WHI2	YGL176C	YGL176C	0.4398	0.1182
YGL242C	YGL242C	YKL092C	BUD2	0.4396	0.2074
YDR383C	NKP1	YMR112C	MED11	0.4394	0.0625
YEL020W-A	TIM9	YOR371C	GPB1	0.4394	-0.1339
YGL051W	MST27	YFR013W	IOC3	0.4393	-0.1925
YOR043W	WHI2	YBR229C	ROT2	0.4392	-0.0766
YPL009C	RQC2	YHR023W	MYO1	0.4391	0.0179
YNL074C	MLF3	YJL149W	DAS1	0.4387	0.1747
YLR096W	KIN2	YKR052C	MRS4	0.4384	-0.1088
YMR062C	ARG7	YHR029C	YHI9	0.4382	0.3046
YLR452C	SST2	YJL187C	SWE1	0.4381	-0.1351
YBR157C	ICS2	YDL155W	CLB3	0.4379	-0.0851
YHL048W	COS8	YER046W	SPO73	0.4379	0.1447
YMR046C	YMR046C	YIL082W	Unknown	0.4379	0.2705
YEL068C	YEL068C	YLR452C	SST2	0.4376	0.0406
YOL088C	MPD2	YIR022W	SEC11	0.4375	0.1677
YBL007C	SLA1	YBL079W	NUP170	0.4374	0.0921
YBR008C	FLR1	YBR071W	YBR071W	0.4374	0.0599
YCL024W	KCC4	YIL166C	YIL166C	0.4372	-0.0649
YJL036W	SNX4	YMR215W	GAS3	0.4371	-0.1416
YML110C	COQ5	YHR016C	YSC84	0.4371	0.1234
YEL056W	HAT2	YJL187C	SWE1	0.4370	0.1691
YGR157W	CHO2	YER020W	GPA2	0.4369	-0.0066
YJL178C	ATG27	YJL126W	NIT2	0.4369	0.2154
YHR016C	YSC84	YLR176C	RFX1	0.4368	0.4242
YDL150W	RPC53	YMR209C	YMR209C	0.4367	0.2399
YJR056C	YJR056C	YFR013W	IOC3	0.4367	0.1577
YBR249C	ARO4	YJR025C	BNA1	0.4366	0.1131
YDL132W	CDC53	YGL035C	MIG1	0.4366	-0.1265
YER046W	SPO73	YBR137W	YBR137W	0.4366	0.0658
YBL017C	PEP1	YBL105C	PKC1	0.4365	0.3278
YGR168C	YGR168C	YMR005W	TAF4	0.4365	-0.1329
YDL155W	CLB3	YDL167C	NRP1	0.4364	0.0564
YIL133C	RPL16a	YBR087W	RFC5	0.4363	0.2149
YKR085C	MRPL20	YLR455W	PDP3	0.4363	0.1984
YDL133W	SRF1	YMR158W	MRPS8	0.4362	-0.0630
YBR193C	MED8	YJL051W	IRC8	0.4360	-0.0792
YHR039C	MSC7	YGL257C	MNT2	0.4359	0.0347
YLR375W	STP3	YPL031C	PHO85	0.4359	0.2638
YGR006W	PRP18	YJL051W	IRC8	0.4358	-0.0830
YJL187C	SWE1	YBR137W	YBR137W	0.4356	-0.1583
YGR258C	RAD2	YLL040C	VPS13	0.4355	0.0409

YBL040C	ERD2	YFL018C	LPD1	0.4354	-0.1229
YLR452C	SST2	YEL068C	YEL068C	0.4353	0.2356
YPL058C	PDR12	YDR420W	HKR1	0.4352	0.1750
YMR046C	YMR046C	YKL160W	ELF1	0.4348	-0.1533
YJR074W	MOG1	YIL061C	SNP1	0.4346	-0.0426
YER088C	DOT6	YGR001C	EFM5	0.4345	-0.1440
YJL126W	NIT2	YOL081W	IRA2	0.4345	-0.1418
YKL116C	PRR1	YDR144C	MKC7	0.4343	0.0651
YNL035C	YNL035C	YJR103W	URA8	0.4341	0.1627
YPL233W	NSL1	YGR222W	PET54	0.4341	0.0364
YPL226W	NEW1	YDR034C	LYS14	0.4340	-0.2765
YDR530C	APA2	YGL192W	IME4	0.4338	0.1388
YNL322C	KRE1	YPL147W	PXA1	0.4337	-0.1723
YML121W	GTR1	YOL073C	DSC2	0.4334	0.0696
YJL036W	SNX4	YHR027C	RPN1	0.4333	0.1320
YML098W	TAF13	YJL149W	DAS1	0.4332	-0.1415
YOR264W	DSE3	YFL016C	MDJ1	0.4332	-0.1933
YKR080W	MTD1	YKL096W	CWP1	0.4331	-0.1554
YJL126W	NIT2	YGR012W	MCY1	0.4329	0.1244
YOL147C	PEX11	YER186C	YER186C	0.4327	-0.0498
YGR023W	MTL1	YLR057W	MNL2	0.4325	0.2028
YBR071W	YBR071W	YDR367W	KEI1	0.4321	-0.1428
YJR103W	URA8	YOL026C	MIM1	0.4320	0.2677
YGR028W	MSP1	YMR112C	MED11	0.4318	0.1026
YJL204C	RCY1	YKL116C	PRR1	0.4316	0.1909
YDL150W	RPC53	YKR085C	MRPL20	0.4315	0.0602
YML071C	COG8	YKL092C	BUD2	0.4313	0.1599
YDL147W	RPN5	YDL116W	NUP84	0.4311	0.0550
YGR208W	SER2	YNL321W	VNX1	0.4310	-0.2231
YFR042W	KEG1	YGR023W	MTL1	0.4307	-0.0321
YMR154C	RIM13	YAL056W	GPB2	0.4307	0.0849
YLL038C	ENT4	YNL135C	FPR1	0.4305	-0.0617
YOL130W	ALR1	YNR034W	SOL1	0.4302	-0.1420
YJL218W	YJL218W	YIL066C	RNR3	0.4300	-0.0360
YBL017C	PEP1	YBR222C	PCS60	0.4299	0.0315
YGL025C	PGD1	YIL112W	HOS4	0.4299	0.0003
YDR339C	FCF1	YIL043C	CBR1	0.4295	0.2615
YKR080W	MTD1	YDR134C	YDR134C	0.4294	-0.0818
YJL208C	NUC1	YGR150C	CCM1	0.4293	0.2918
YJL047C	RTT101	YOL089C	HAL9	0.4293	0.0119
YBL007C	SLA1	YBR071W	YBR071W	0.4289	0.1889
YGL178W	MPT5	YKL067W	YNK1	0.4289	-0.0650
YJL163C	YJL163C	YBR147W	RTC2	0.4282	0.1821
YIL171W	YIL171W	YHL048W	COS8	0.4281	0.1547
YMR046C	YMR046C	YIL040W	APQ12	0.4280	-0.2086
YMR152W	YIM1	YML004C	GLO1	0.4278	0.2184
YPL233W	NSL1	YLR057W	MNL2	0.4278	0.0187

YLR064W	PER33	YOR103C	OST2	0.4276	0.0108
YLR096W	KIN2	YHR039C	MSC7	0.4276	0.1349
YCL009C	ILV6	YNL074C	MLF3	0.4275	0.0836
YOR196C	LIP5	YLR443W	ECM7	0.4272	0.1772
YMR215W	GAS3	YDR134C	YDR134C	0.4271	0.1927
YNL046W	YNL046W	YOL092W	YPQ1	0.4268	0.0686
YDR513W	GRX2	YLL040C	VPS13	0.4267	0.2413
YMR215W	GAS3	YBL029W	YBL029W	0.4267	-0.2731
YNL034W	YNL034W	YOL104C	NDJ1	0.4265	0.0021
YNL278W	CAF120	YOL104C	NDJ1	0.4263	-0.0121
YOR196C	LIP5	YKL110C	KTI12	0.4263	0.1247
YPL016W	SWI1	YGL035C	MIG1	0.4263	0.0816
YJR088C	EMC2	YPL237W	SUI3	0.4261	0.1449
YML036W	CGI121	YLR452C	SST2	0.4261	-0.1402
YOR043W	WHI2	YDR383C	NKP1	0.4260	0.1418
YIR022W	SEC11	YDL132W	CDC53	0.4258	0.0336
YOR131C	YOR131C	YIL040W	APQ12	0.4256	0.0020
YHR027C	RPN1	YNL322C	KRE1	0.4255	0.1604
YAL009W	SPO7	YCR083W	TRX3	0.4254	0.1241
YBR067C	TIP1	YEL056W	HAT2	0.4253	-0.1036
YHR115C	DMA1	YGR258C	RAD2	0.4253	-0.1713
YGR157W	CHO2	YIL171W	YIL171W	0.4252	0.0169
YBR157C	ICS2	YER116C	SLX8	0.4251	0.0583
YLR368W	MDM30	YJL036W	SNX4	0.4250	-0.1114
YBR071W	YBR071W	YBL007C	SLA1	0.4249	0.0946
YDL180W	YDL180W	YGL176C	YGL176C	0.4249	0.0915
YJL047C	RTT101	YOR127W	RGA1	0.4248	0.1520
YLR057W	MNL2	YLR176C	RFX1	0.4248	-0.1867
YML098W	TAF13	YML038C	YMD8	0.4248	0.0934
YOL147C	PEX11	YNL329C	PEX6	0.4246	0.1623
YOR117W	RPT5	YDR351W	SBE2	0.4246	0.1308
YGL169W	SUA5	YGL192W	IME4	0.4243	0.0002
YLR180W	SAM1	YJR036C	HUL4	0.4240	-0.1985
YMR062C	ARG7	YJR073C	OPI3	0.4240	-0.2233
YLR375W	STP3	YOL097C	WRS1	0.4237	-0.1479
YOR117W	RPT5	YFL018C	LPD1	0.4237	0.1554
YDR319C	YFT2	YHR034C	PIH1	0.4235	0.0125
YOR088W	YOR088W	YKL116C	PRR1	0.4234	-0.0662
YNL034W	YNL034W	YBL020W	RFT1	0.4233	0.0801
YBR008C	FLR1	YDR481C	PHO8	0.4232	0.2132
YDR481C	PHO8	YDR441C	APT2	0.4231	0.1664
YJR036C	HUL4	YER116C	SLX8	0.4231	0.0974
YLR375W	STP3	YGR150C	CCM1	0.4230	-0.2548
YNL074C	MLF3	YMR046C	YMR046C	0.4230	0.1661
YOL018C	TLG2	YLR206W	ENT2	0.4229	0.0773
YNL041C	COG6	YFR013W	IOC3	0.4228	0.2912
YDR178W	SDH4	YGR208W	SER2	0.4227	-0.1842

YOR092W	ECM3	YIL066C	RNR3	0.4225	0.0966
YER116C	SLX8	YHR050W	SMF2	0.4224	-0.0386
YBL020W	RFT1	YBR037C	SCO1	0.4223	0.1185
YKL184W	SPE1	YGL051W	MST27	0.4222	0.2986
YER182W	FMP10	YOR048C	RAT1	0.4220	-0.0896
YHR027C	RPN1	YMR062C	ARG7	0.4220	-0.0331
YER023W	PRO3	YKL073W	LHS1	0.4219	0.0738
YKL116C	PRR1	YDL047W	SIT4	0.4219	0.0706
YHR115C	DMA1	YDR420W	HKR1	0.4218	0.1710
YKL184W	SPE1	YNL322C	KRE1	0.4218	0.1317
YHR029C	YHI9	YDR481C	PHO8	0.4217	0.3588
YLR435W	TSR2	YLR287C	YLR287C	0.4216	0.0507
YBR121C	GRS1	YIL040W	APQ12	0.4215	-0.1683
YGR207C	CIR1	YOR103C	OST2	0.4215	-0.1185
YIL089W	YIL089W	YGL207W	SPT16	0.4215	-0.0317
YKL116C	PRR1	YJL204C	RCY1	0.4215	0.0265
YGL004C	RPN14	YHR209W	CRG1	0.4214	0.0223
YMR062C	ARG7	YDR531W	CAB1	0.4213	0.0506
YML048W	GSF2	YMR123W	PKR1	0.4212	0.0973
YMR154C	RIM13	YPL071C	YPL071C	0.4212	0.0564
YER186C	YER186C	YKL073W	LHS1	0.4211	-0.1520
YDL116W	NUP84	YDL103C	QRI1	0.4206	0.0843
YPL031C	PHO85	YGL025C	PGD1	0.4206	0.0223
YOL018C	TLG2	YBL020W	RFT1	0.4202	-0.0998
YBR147W	RTC2	YGL169W	SUA5	0.4199	-0.2015
YHR050W	SMF2	YMR215W	GAS3	0.4198	0.1657
YFL047W	RGD2	YNR034W	SOL1	0.4196	-0.0628
YBL089W	AVT5	YJL118W	YJL118W	0.4195	-0.0810
YLR096W	KIN2	YPL012W	RRP12	0.4194	0.0766
YHR027C	RPN1	YIL142W	CCT2	0.4192	0.1441
YDL164C	CDC9	YML013W	UBX2	0.4191	0.1334
YMR115W	MGR3	YML036W	CGI121	0.4191	0.0778
YGL242C	YGL242C	YNL138W	SRV2	0.4189	0.0641
YJR074W	MOG1	YAL009W	SPO7	0.4189	0.0407
YHR023W	MYO1	YDR441C	APT2	0.4188	-0.1609
YIL015C-A	YIL015C-A	YOL104C	NDJ1	0.4184	0.0381
YLR047C	FRE8	YOL113W	SKM1	0.4184	0.1311
YPL234C	VMA11	YPL016W	SWI1	0.4184	-0.0889
YJL047C	RTT101	YOR101W	RAS1	0.4182	-0.1959
YER020W	GPA2	YOR054C	VHS3	0.4181	0.0559
YNL326C	PFA3	YML013W	UBX2	0.4180	-0.0939
YLR046C	YLR046C	YLR052W	IES3	0.4177	-0.1318
YGR157W	CHO2	YKL073W	LHS1	0.4173	0.0566
YMR046C	YMR046C	YJL051W	IRC8	0.4173	-0.0467
YJL171C	TOH1	YEL068C	YEL068C	0.4170	-0.1166
YDL133W	SRF1	YER186C	YER186C	0.4168	0.1415
YPR022C	SDD4	YBR067C	TIP1	0.4168	-0.0691

YNL135C	FPR1	YOL043C	NTG2	0.4165	-0.0027
YML013W	UBX2	YGR012W	MCY1	0.4164	0.1957
YGR258C	RAD2	YIL043C	CBR1	0.4160	-0.0275
YJL036W	SNX4	YKR051W	YKR051W	0.4158	0.0050
YNL326C	PFA3	YOR051C	ETT1	0.4158	0.1334
YCL018W	LEU2	YJL212C	OPT1	0.4157	-0.0281
YPR196W	YPR196W	YLR047C	FRE8	0.4157	0.0954
YBL089W	AVT5	YDL103C	QRI1	0.4155	-0.0591
YML038C	YMD8	YKL116C	PRR1	0.4154	-0.1437
YIL066C	RNR3	YDR420W	HKR1	0.4150	0.2467
YKL116C	PRR1	YER144C	UBP5	0.4150	0.1257
YAL009W	SPO7	YOL147C	PEX11	0.4149	0.0571
YCR031C	RPS14a	YLR342W	FKS1	0.4148	0.1995
YNL322C	KRE1	YGL176C	YGL176C	0.4148	-0.0114
YNL321W	VNX1	YBR229C	ROT2	0.4148	0.0354
YOL026C	MIM1	YOR043W	WHI2	0.4144	-0.0329
YCL024W	KCC4	YNL041C	COG6	0.4143	-0.1630
YGR207C	CIR1	YOR092W	ECM3	0.4143	-0.1030
YIL015C-A	YIL015C-A	YGL193C	YGL193C	0.4142	-0.0568
YDL164C	CDC9	YLR258W	GSY2	0.4141	-0.1796
YPR196W	YPR196W	YFL047W	RGD2	0.4141	0.0787
YML079W	YML079W	YML036W	CGI121	0.4140	0.2794
YBR168W	PEX32	YOR088W	YOR088W	0.4139	0.1119
YJR074W	MOG1	YML098W	TAF13	0.4139	0.0326
YLR047C	FRE8	YNL329C	PEX6	0.4139	-0.0707
YGL195W	GCN1	YOR080W	DIA2	0.4137	0.1915
YGL176C	YGL176C	YOR144C	ELG1	0.4137	0.1432
YML038C	YMD8	YDL002C	NHP10	0.4137	-0.1294
YOL164W	BDS1	YJL171C	TOH1	0.4133	0.0927
YLL061W	MMP1	YHR050W	SMF2	0.4130	0.1732
YGR006W	PRP18	YDR420W	HKR1	0.4128	-0.0757
YPR015C	YPR015C	YJR088C	EMC2	0.4128	0.0215
YBR193C	MED8	YLR426W	TDA5	0.4127	0.0887
YOR196C	LIP5	YIL089W	YIL089W	0.4125	-0.1922
YAL056W	GPB2	YJL163C	YJL163C	0.4124	0.1816
YER020W	GPA2	YFR037C	RSC8	0.4124	0.2073
YMR152W	YIM1	YOR048C	RAT1	0.4123	-0.1348
YBR222C	PCS60	YER186C	YER186C	0.4120	-0.0305
YJL030W	MAD2	YBL040C	ERD2	0.4120	0.1030
YJR084W	YJR084W	YMR062C	ARG7	0.4119	-0.0771
YBR161W	CSH1	YBR229C	ROT2	0.4118	0.0293
YKL184W	SPE1	YGR222W	PET54	0.4117	-0.2445
YOR048C	RAT1	YML079W	YML079W	0.4116	-0.1686
YIR022W	SEC11	YOL088C	MPD2	0.4115	0.1548
YNL200C	NNR1	YLR248W	RCK2	0.4115	0.2585
YLR206W	ENT2	YIL119C	RPI1	0.4113	0.0556
YOR088W	YOR088W	YJR036C	HUL4	0.4112	0.0498

YEL021W	URA3	YBR069C	TAT1	0.4111	0.1476
YJL154C	VPS35	YJR086W	STE18	0.4110	-0.0073
YGL025C	PGD1	YHR115C	DMA1	0.4109	0.0002
YBR087W	RFC5	YDR339C	FCF1	0.4108	0.1135
YLL024C	SSA2	YPL226W	NEW1	0.4108	0.0884
YLR455W	PDP3	YCR031C	RPS14a	0.4105	0.0181
YJL208C	NUC1	YKL110C	KTI12	0.4104	0.0666
YGL226C-A	OST5	YNR034W	SOL1	0.4103	-0.2410
YHR063C	PAN5	YOR196C	LIP5	0.4102	0.0354
YKL021C	MAK11	YHL007C	STE20	0.4102	0.4280
YLR096W	KIN2	YLR430W	SEN1	0.4102	0.1920
YDL217C	TIM22	YFR004W	RPN11	0.4101	-0.1116
YDR497C	ITR1	YKR080W	MTD1	0.4100	-0.0435
YLR452C	SST2	YOL113W	SKM1	0.4100	-0.0630
YNL322C	KRE1	YDR383C	NKP1	0.4098	0.0879
YNL242W	ATG2	YBR161W	CSH1	0.4096	-0.0407
YOR144C	ELG1	YCL024W	KCC4	0.4091	0.2751
YIL112W	HOS4	YDR034C	LYS14	0.4089	0.0001
YJR025C	BNA1	YJR032W	CPR7	0.4087	-0.1471
YBR077C	SLM4	YBR154C	RPB5	0.4086	0.1508
YOR196C	LIP5	YJR074W	MOG1	0.4084	0.1781
YFR047C	BNA6	YGR211W	ZPR1	0.4083	-0.0435
YGR184C	UBR1	YNL074C	MLF3	0.4083	-0.0785
YHR209W	CRG1	YDR134C	YDR134C	0.4081	-0.0871
YNL242W	ATG2	YNL329C	PEX6	0.4081	0.1261
YOR131C	YOR131C	YEL076W-C	Unknown	0.4081	-0.1559
YDL132W	CDC53	YPL064C	CWC27	0.4079	-0.1027
YIL009W	FAA3	YDL211C	YDL211C	0.4079	0.1286
YHR063C	PAN5	YLR443W	ECM7	0.4078	0.2086
YPL233W	NSL1	YLR237W	THI7	0.4077	-0.1132
YLR248W	RCK2	YMR112C	MED11	0.4075	-0.2308
YBR163W	EXO5	YPL058C	PDR12	0.4072	-0.1454
YNL215W	IES2	YLR442C	SIR3	0.4072	0.0602
YOR144C	ELG1	YLR257W	YLR257W	0.4071	-0.1492
YOR043W	WHI2	YLR375W	STP3	0.4069	0.0243
YLR442C	SIR3	YOL130W	ALR1	0.4068	0.0456
YJL126W	NIT2	YJR032W	CPR7	0.4067	0.2729
YBR067C	TIP1	YBR071W	YBR071W	0.4065	-0.1127
YLR248W	RCK2	YNL200C	NNR1	0.4065	0.1189
YGR208W	SER2	YPL237W	SUI3	0.4064	0.0498
YJL187C	SWE1	YJL051W	IRC8	0.4064	0.0608
YBR249C	ARO4	YGR288W	MAL13	0.4062	0.0906
YGL004C	RPN14	YIL089W	YIL089W	0.4062	0.1186
YER116C	SLX8	YOR051C	ETT1	0.4061	0.1379
YHR034C	PIH1	YLR057W	MNL2	0.4061	-0.1497
YHR023W	MYO1	YMR123W	PKR1	0.4059	-0.1293
YIL166C	YIL166C	YPL144W	POC4	0.4058	-0.1183

YJL030W	MAD2	YPL016W	SWI1	0.4058	-0.0552
YBR168W	PEX32	YLL024C	SSA2	0.4057	0.1662
YLR452C	SST2	YLR435W	TSR2	0.4055	0.1250
YMR046C	YMR046C	YAR028W	YAR028W	0.4050	0.1301
YBR166C	TYR1	YER144C	UBP5	0.4049	0.2481
YDR420W	HKR1	YLR172C	DPH5	0.4047	-0.0427
YHR034C	PIH1	YNL280C	ERG24	0.4047	0.0032
YJL030W	MAD2	YLR052W	IES3	0.4047	0.1961
YOR043W	WHI2	YNL124W	NAF1	0.4047	0.0683
YCL018W	LEU2	YNL074C	MLF3	0.4046	0.1936
YGR211W	ZPR1	YBR132C	AGP2	0.4046	-0.0521
YLR059C	REX2	YGR207C	CIR1	0.4044	-0.1174
YAL056W	GPB2	YOR033C	EXO1	0.4041	-0.0717
YHL048W	COS8	YEL076W-C	Unknown	0.4041	0.0645
YMR005W	TAF4	YLR389C	STE23	0.4041	-0.0151
YDR351W	SBE2	YIL112W	HOS4	0.4039	0.1711
YLR435W	TSR2	YGR158C	MTR3	0.4037	0.2195
YJR032W	CPR7	YHL007C	STE20	0.4036	0.0260
YOL104C	NDJ1	YLR237W	THI7	0.4034	-0.1187
YOR371C	GPB1	YDR134C	YDR134C	0.4034	-0.1504
YIL043C	CBR1	YLR206W	ENT2	0.4033	-0.1530
YJL187C	SWE1	YDL155W	CLB3	0.4032	0.1068
YJL178C	ATG27	YPL234C	VMA11	0.4032	0.0810
YLR368W	MDM30	YOL130W	ALR1	0.4030	0.0019
YER023W	PRO3	YLR442C	SIR3	0.4029	-0.2117
YLR172C	DPH5	YGR208W	SER2	0.4029	0.0900
YDR134C	YDR134C	YBL079W	NUP170	0.4027	-0.0622
YGL242C	YGL242C	YDL147W	RPN5	0.4024	0.0642
YOR101W	RAS1	YNL074C	MLF3	0.4023	-0.2061
YKL073W	LHS1	YLR257W	YLR257W	0.4021	0.0768
YPL064C	CWC27	YIL061C	SNP1	0.4021	-0.0653
YHR054C	YHR054C	YGR222W	PET54	0.4020	-0.0987
YIL046W	MET30	YOR196C	LIP5	0.4020	0.0985
YCL024W	KCC4	YOL104C	NDJ1	0.4019	-0.1277
YMR112C	MED11	YGR168C	YGR168C	0.4019	0.0778
YHR023W	MYO1	YOR054C	VHS3	0.4017	0.0748
YJR036C	HUL4	YLR368W	MDM30	0.4017	0.0834
YEL064C	AVT2	YOL147C	PEX11	0.4016	0.1768
YNL242W	ATG2	YLR330W	CHS5	0.4015	-0.0023
YPL237W	SUI3	YGR208W	SER2	0.4013	0.3697
YCL024W	KCC4	YBL040C	ERD2	0.4012	-0.0946
YDR481C	PHO8	YBR008C	FLR1	0.4012	0.2890
YGR157W	CHO2	YHR029C	YHI9	0.4012	-0.1239
YOR088W	YOR088W	YJL047C	RTT101	0.4012	-0.0346
YKL184W	SPE1	YER023W	PRO3	0.4011	0.0356
YDL132W	CDC53	YDL164C	CDC9	0.4010	0.0987
YBR163W	EXO5	YFL016C	MDJ1	0.4009	-0.0193

YLR287C	YLR287C	YHR115C	DMA1	0.4008	-0.0055
YOL062C	APM4	YGR157W	CHO2	0.4006	0.0899
YGL257C	MNT2	YGR157W	CHO2	0.4005	0.0671
YGL035C	MIG1	YLR047C	FRE8	0.4005	-0.0517
YCR041W	YCR041W	YOL026C	MIM1	0.4004	-0.0952
YDL180W	YDL180W	YIL040W	APQ12	0.4004	0.0466
YDL133W	SRF1	YDR531W	CAB1	0.4003	0.1044
YGR152C	RSR1	YCL024W	KCC4	0.4003	0.0190
YDL089W	NUR1	YBR274W	CHK1	0.4002	0.0352
YLR180W	SAM1	YFR037C	RSC8	0.4002	0.0949
YIL171W	YIL171W	YLR096W	KIN2	0.4001	-0.0766
YOL094C	RFC4	YJR068W	RFC2	0.4001	0.0212
YGL017W	ATE1	YOR131C	YOR131C	0.4000	0.1668
YOL094C	RFC4	YGR149W	GPC1	0.4000	-0.2534
YGL169W	SUA5	YHL048W	COS8	0.3999	-0.0672
YGL176C	YGL176C	YGR006W	PRP18	0.3998	0.0293
YBL040C	ERD2	YDL150W	RPC53	0.3996	-0.1393
YGR012W	MCY1	YFR004W	RPN11	0.3995	0.2194
YKR051W	YKR051W	YOR048C	RAT1	0.3994	-0.0587
YDR383C	NKP1	YDR058C	TGL2	0.3993	0.0607
YFL018C	LPD1	YDL116W	NUP84	0.3993	0.0428
YIL061C	SNP1	YEL064C	AVT2	0.3993	0.1318
YJL149W	DAS1	YEL020W-A	TIM9	0.3991	-0.0550
YOR080W	DIA2	YPL031C	PHO85	0.3991	-0.0405
YJL158C	CIS3	YDR319C	YFT2	0.3989	0.0524
YMR215W	GAS3	YPL009C	RQC2	0.3988	0.2858
YER037W	PHM8	YER035W	EDC2	0.3986	0.1130
YLR052W	IES3	YJL036W	SNX4	0.3986	-0.0407
YOR131C	YOR131C	YGR184C	UBR1	0.3986	-0.0888
YLR452C	SST2	YER020W	GPA2	0.3984	-0.1311
YNL326C	PFA3	YOL093W	TRM10	0.3983	0.0634
YKL092C	BUD2	YPL009C	RQC2	0.3982	0.0335
YGL035C	MIG1	YML013W	UBX2	0.3980	-0.0921
YBR168W	PEX32	YLR248W	RCK2	0.3979	0.0957
YDR420W	HKR1	YIL066C	RNR3	0.3979	0.0442
YLR449W	FPR4	YEL055C	POL5	0.3978	0.1166
YLR455W	PDP3	YLR206W	ENT2	0.3978	0.1324
YBR061C	TRM7	YGL257C	MNT2	0.3976	-0.1136
YHR023W	MYO1	YOR142W	LSC1	0.3975	0.2339
YJL154C	VPS35	YGR150C	CCM1	0.3975	0.0882
YGR168C	YGR168C	YHR115C	DMA1	0.3974	-0.1460
YER049W	TPA1	YIR026C	YVH1	0.3973	0.0841
YJL171C	TOH1	YDR481C	PHO8	0.3973	0.0645
YEL064C	AVT2	YNL135C	FPR1	0.3971	-0.0668
YBR071W	YBR071W	YKL096W	CWP1	0.3970	-0.1129
YFR047C	BNA6	YML007W	YAP1	0.3970	-0.1515
YKR075C	YKR075C	YHR043C	DOG2	0.3969	-0.1460

YLR176C	RFX1	YBR087W	RFC5	0.3969	-0.1070
YOL093W	TRM10	YJR008W	MHO1	0.3969	-0.1496
YPR080W	TEF1	YOR131C	YOR131C	0.3969	-0.0683
YOL113W	SKM1	YDL147W	RPN5	0.3968	0.0062
YGL226W	MTC3	YMR158W	MRPS8	0.3967	0.1465
YLR096W	KIN2	YLR442C	SIR3	0.3967	0.1829
YGL195W	GCN1	YPL009C	RQC2	0.3965	0.0904
YGL169W	SUA5	YDR528W	HLR1	0.3965	0.1390
YDR034C	LYS14	YDR531W	CAB1	0.3963	0.1579
YPL001W	HAT1	YCR083W	TRX3	0.3963	-0.0790
YGL051W	MST27	YJL030W	MAD2	0.3959	0.0009
YLR443W	ECM7	YLL024C	SSA2	0.3958	0.0233
YDR319C	YFT2	YDL103C	QRI1	0.3956	0.0849
YKL116C	PRR1	YBR147W	RTC2	0.3956	-0.1004
YLR430W	SEN1	YOR371C	GPB1	0.3956	0.0228
YML016C	PPZ1	YLL024C	SSA2	0.3956	-0.1455
YNL321W	VNX1	YJL163C	YJL163C	0.3956	0.0679
YJR097W	JJJ3	YPL012W	RRP12	0.3952	0.1030
YOR054C	VHS3	YNL322C	KRE1	0.3952	-0.1189
YDL150W	RPC53	YLR248W	RCK2	0.3951	0.0602
YFL016C	MDJ1	YCL024W	KCC4	0.3950	0.1614
YJL036W	SNX4	YJL158C	CIS3	0.3950	-0.1097
YNL135C	FPR1	YNR034W	SOL1	0.3949	0.1561
YIL066C	RNR3	YAR028W	YAR028W	0.3946	0.1045
YOR251C	TUM1	YMR158W	MRPS8	0.3946	-0.0294
YBR163W	EXO5	YBR154C	RPB5	0.3945	0.0444
YEL064C	AVT2	YOR092W	ECM3	0.3944	0.0555
YGL201C	MCM6	YJL118W	YJL118W	0.3944	0.0946
YGL178W	MPT5	YIL082W	Unknown	0.3943	0.0901
YKL184W	SPE1	YBR067C	TIP1	0.3941	0.0978
YBR274W	CHK1	YBL030C	PET9	0.3938	0.0460
YOR101W	RAS1	YOR371C	GPB1	0.3938	-0.1846
YDR420W	HKR1	YGR023W	MTL1	0.3937	0.0864
YBL105C	PKC1	YNL280C	ERG24	0.3935	-0.0518
YJL118W	YJL118W	YBL089W	AVT5	0.3935	-0.0383
YJL058C	BIT61	YOL147C	PEX11	0.3932	0.0428
YOL164W	BDS1	YGR258C	RAD2	0.3932	0.0014
YDR515W	SLF1	YOR371C	GPB1	0.3931	-0.0961
YBR239C	ERT1	YBR147W	RTC2	0.3930	0.0771
YJL047C	RTT101	YOR051C	ETT1	0.3930	0.0206
YKR085C	MRPL20	YGR028W	MSP1	0.3930	0.0206
YNL024C	EFM6	YBR157C	ICS2	0.3930	0.0884
YBR229C	ROT2	YIL089W	YIL089W	0.3929	0.1134
YDL147W	RPN5	YGR157W	CHO2	0.3929	0.1528
YLR452C	SST2	YPL058C	PDR12	0.3929	0.0584
YJL030W	MAD2	YJR068W	RFC2	0.3927	0.0704
YBR161W	CSH1	YBL017C	PEP1	0.3926	0.0537

YMR067C	UBX4	YDL150W	RPC53	0.3925	0.2431
YGR258C	RAD2	YHL048W	COS8	0.3922	0.0643
YDL111C	RRP42	YGR207C	CIR1	0.3921	-0.0985
YMR112C	MED11	YLR248W	RCK2	0.3920	-0.0283
YNL215W	IES2	YDR515W	SLF1	0.3920	0.0260
YDL133W	SRF1	YML038C	YMD8	0.3919	0.0299
YJL051W	IRC8	YDL103C	QRI1	0.3919	-0.0312
YLR237W	THI7	YOR131C	YOR131C	0.3918	-0.0741
YML072C	TCB3	YBR069C	TAT1	0.3917	0.1442
YJR088C	EMC2	YJL140W	RPB4	0.3916	0.0925
YCL026C-A	FRM2	YIL112W	HOS4	0.3915	0.1309
YJR088C	EMC2	YGL025C	PGD1	0.3915	-0.2303
YLR064W	PER33	YBL079W	NUP170	0.3913	-0.0315
YHR115C	DMA1	YML038C	YMD8	0.3911	0.1185
YJL154C	VPS35	YOL092W	YPQ1	0.3911	-0.0924
YBR166C	TYR1	YLR052W	IES3	0.3910	-0.0716
YML110C	COQ5	YML079W	YML079W	0.3910	0.0590
YOR389W	YOR389W	YNR034W	SOL1	0.3909	-0.0351
YLR096W	KIN2	YLR057W	MNL2	0.3908	0.0420
YLL024C	SSA2	YHR054C	YHR054C	0.3906	0.1193
YJL208C	NUC1	YPL183C	RTT10	0.3905	0.2686
YJL163C	YJL163C	YJR008W	MHO1	0.3905	0.0036
YNL041C	COG6	YLR389C	STE23	0.3905	0.1084
YEL071W	DLD3	YOR101W	RAS1	0.3902	-0.1074
YDL132W	CDC53	YDL147W	RPN5	0.3901	0.0552
YHR027C	RPN1	YDR034C	LYS14	0.3901	-0.0683
YCR041W	YCR041W	YHR043C	DOG2	0.3900	0.0285
YBR071W	YBR071W	YBR067C	TIP1	0.3899	-0.0977
YKR085C	MRPL20	YEL068C	YEL068C	0.3899	0.2646
YJL204C	RCY1	YJL030W	MAD2	0.3897	0.0305
YKL160W	ELF1	YDR351W	SBE2	0.3897	-0.0582
YLR206W	ENT2	YHR115C	DMA1	0.3896	0.0009
YLR056W	ERG3	YML091C	RPM2	0.3895	-0.1313
YMR215W	GAS3	YJL036W	SNX4	0.3894	-0.3734
YER022W	SRB4	YBR239C	ERT1	0.3893	-0.1309
YLR330W	CHS5	YFR004W	RPN11	0.3893	0.1557
YIR022W	SEC11	YKR075C	YKR075C	0.3892	-0.0749
YOR048C	RAT1	YIR026C	YVH1	0.3892	0.0228
YMR010W	ANY1	YML013W	UBX2	0.3891	0.0638
YKL183W	LOT5	YKR051W	YKR051W	0.3890	-0.0835
YEL068C	YEL068C	YOL147C	PEX11	0.3887	-0.1315
YER088C	DOT6	YNL124W	NAF1	0.3886	0.0940
YBL029W	YBL029W	YER185W	PUG1	0.3885	0.0389
YNR036C	MRPS12	YKL184W	SPE1	0.3885	-0.1800
YIL142W	CCT2	YIR026C	YVH1	0.3883	0.1319
YOL147C	PEX11	YEL056W	HAT2	0.3882	-0.0125
YER026C	CHO1	YNL124W	NAF1	0.3879	-0.1339

YDL002C	NHP10	YIL158W	AIM20	0.3878	0.0518
YML096W	YML096W	YOR054C	VHS3	0.3877	-0.0899
YOR088W	YOR088W	YFR047C	BNA6	0.3875	0.1406
YJL140W	RPB4	YOR127W	RGA1	0.3874	-0.0048
YHR016C	YSC84	YLL040C	VPS13	0.3870	0.4130
YIL089W	YIL089W	YEL076W-C	Unknown	0.3868	0.1001
YPL233W	NSL1	YOL081W	IRA2	0.3867	-0.0266
YOL062C	APM4	YPL234C	VMA11	0.3866	-0.0682
YER116C	SLX8	YLR059C	REX2	0.3864	0.1209
YNL041C	COG6	YJL118W	YJL118W	0.3863	-0.0282
YGL004C	RPN14	YHR043C	DOG2	0.3861	0.1025
YBR037C	SCO1	YLR449W	FPR4	0.3860	-0.0962
YCR071C	IMG2	YFR011C	MIC19	0.3858	0.1356
YGR168C	YGR168C	YMR112C	MED11	0.3854	0.0354
YKL116C	PRR1	YMR010W	ANY1	0.3854	0.0469
YER019W	ISC1	YLR426W	TDA5	0.3852	0.1576
YER037W	PHM8	YHR209W	CRG1	0.3851	0.0696
YEL020W-A	TIM9	YKL067W	YNK1	0.3850	0.1754
YLR231C	BNA5	YOL113W	SKM1	0.3848	-0.0332
YMR005W	TAF4	YGL193C	YGL193C	0.3847	-0.1104
YER022W	SRB4	YCR083W	TRX3	0.3846	-0.0610
YML004C	GLO1	YPL147W	PXA1	0.3846	-0.0607
YAR028W	YAR028W	YJL047C	RTT101	0.3845	-0.0171
YFL018C	LPD1	YDL103C	QRI1	0.3841	0.0329
YER019W	ISC1	YML036W	CGI121	0.3839	0.0285
YJR088C	EMC2	YBR137W	YBR137W	0.3839	0.2279
YMR115W	MGR3	YNL322C	KRE1	0.3836	-0.0637
YDR383C	NKP1	YNL329C	PEX6	0.3834	-0.1390
YLL040C	VPS13	YGR023W	MTL1	0.3832	0.1165
YOL026C	MIM1	YBR067C	TIP1	0.3832	0.0104
YPL234C	VMA11	YOR127W	RGA1	0.3831	-0.2021
YML048W	GSF2	YML004C	GLO1	0.3830	0.0225
YBR166C	TYR1	YEL058W	PCM1	0.3828	0.1487
YFR004W	RPN11	YOL130W	ALR1	0.3828	-0.1101
YOL073C	DSC2	YNL322C	KRE1	0.3828	0.0983
YDL132W	CDC53	YML013W	UBX2	0.3827	0.0029
YKL096W	CWP1	YGL176C	YGL176C	0.3826	0.1847
YBR132C	AGP2	YCR031C	RPS14a	0.3823	-0.0788
YBR163W	EXO5	YLR237W	THI7	0.3823	-0.3576
YGL193C	YGL193C	YGL017W	ATE1	0.3822	-0.0866
YGR012W	MCY1	YMR115W	MGR3	0.3822	0.1925
YJL036W	SNX4	YNL035C	YNL035C	0.3822	0.0199
YPL144W	POC4	YDR420W	HKR1	0.3820	-0.0355
YDR497C	ITR1	YNL024C	EFM6	0.3819	0.1775
YIL089W	YIL089W	YOR196C	LIP5	0.3819	-0.1672
YPL012W	RRP12	YJL208C	NUC1	0.3817	0.3334
YBR071W	YBR071W	YOL062C	APM4	0.3816	0.0325

YDR513W	GRX2	YOL153C	YOL153C	0.3815	0.1816
YPL183C	RTT10	YPL226W	NEW1	0.3813	0.1689
YDL133W	SRF1	YDL147W	RPN5	0.3811	0.0428
YKL073W	LHS1	YGR157W	CHO2	0.3811	0.1343
YFR042W	KEG1	YLL024C	SSA2	0.3810	0.1439
YLR052W	IES3	YKL092C	BUD2	0.3808	0.0361
YLR237W	THI7	YDL111C	RRP42	0.3808	-0.1060
YMR112C	MED11	YLR426W	TDA5	0.3806	0.0299
YGR150C	CCM1	YBL017C	PEP1	0.3805	0.0768
YHR039C	MSC7	YBR163W	EXO5	0.3804	-0.0920
YDL171C	GLT1	YML036W	CGI121	0.3803	-0.1932
YLR443W	ECM7	YLR206W	ENT2	0.3803	0.1067
YFL056C	AAD6	YOL062C	APM4	0.3802	0.0599
YJR068W	RFC2	YGL178W	MPT5	0.3799	-0.1337
YLR181C	VTA1	YJL030W	MAD2	0.3799	0.0828
YGL178W	MPT5	YDL150W	RPC53	0.3798	0.0340
YPL226W	NEW1	YPL183C	RTT10	0.3798	0.0643
YIL040W	APQ12	YDL097C	RPN6	0.3793	0.0708
YKL073W	LHS1	YBR154C	RPB5	0.3791	-0.1371
YOL018C	TLG2	YOL113W	SKM1	0.3790	0.2030
YML004C	GLO1	YML048W	GSF2	0.3787	0.1559
YMR154C	RIM13	YOL097C	WRS1	0.3787	-0.0792
YML079W	YML079W	YER035W	EDC2	0.3786	0.0682
YDL135C	RDI1	YOL147C	PEX11	0.3785	0.1341
YOL026C	MIM1	YNL135C	FPR1	0.3785	0.1444
YDL227C	Ho	YMR046C	YMR046C	0.3784	-0.0013
YPL016W	SWI1	YKL067W	YNK1	0.3784	-0.1189
YPR080W	TEF1	YDL217C	TIM22	0.3782	0.1005
YBL029W	YBL029W	YBL089W	AVT5	0.3781	0.1223
YKL019W	RAM2	YPL071C	YPL071C	0.3780	-0.0395
YNL326C	PFA3	YNL022C	RCM1	0.3778	0.0905
YJL126W	NIT2	YJL047C	RTT101	0.3776	0.1705
YJL051W	IRC8	YGR006W	PRP18	0.3776	-0.0110
YER026C	CHO1	YBR166C	TYR1	0.3775	-0.1908
YJL047C	RTT101	YJL183W	MNN11	0.3774	-0.0449
YPL031C	PHO85	YLR375W	STP3	0.3774	0.1908
YDR383C	NKP1	YDL047W	SIT4	0.3773	0.1049
YLR430W	SEN1	YDR524C	AGE1	0.3773	-0.0064
YNL034W	YNL034W	YPL031C	PHO85	0.3773	-0.0184
YCL009C	ILV6	YOL081W	IRA2	0.3771	0.1444
YOR127W	RGA1	YFL047W	RGD2	0.3771	0.0854
YGR289C	MAL11	YML098W	TAF13	0.3770	-0.0608
YIL165C	YIL165C	YGR001C	EFM5	0.3770	0.1909
YJL217W	REE1	YML007W	YAP1	0.3770	0.0582
YNL022C	RCM1	YPL012W	RRP12	0.3770	0.0414
YBL014C	RRN6	YBR229C	ROT2	0.3767	0.0894
YFR011C	MIC19	YFL018C	LPD1	0.3767	0.0582

YHR104W	GRE3	YDR513W	GRX2	0.3765	0.2003
YLR449W	FPR4	YGR158C	MTR3	0.3765	0.1958
YML004C	GLO1	YMR152W	YIM1	0.3765	0.0484
YBR239C	ERT1	YBL007C	SLA1	0.3764	0.0971
YER022W	SRB4	YBR274W	CHK1	0.3764	-0.0143
YLR435W	TSR2	YKL110C	KTI12	0.3764	0.1477
YPL237W	SUI3	YEL055C	POL5	0.3764	0.1662
YBR071W	YBR071W	YDR319C	YFT2	0.3762	0.1915
YGR149W	GPC1	YJR073C	OPI3	0.3759	0.1485
YJL140W	RPB4	YOR088W	YOR088W	0.3757	0.0912
YLR206W	ENT2	YOL062C	APM4	0.3757	0.1123
YBR222C	PCS60	YBL079W	NUP170	0.3756	0.0547
YKL073W	LHS1	YJR032W	CPR7	0.3752	0.1220
YJR025C	BNA1	YMR062C	ARG7	0.3751	0.0467
YFL049W	SWP82	YIL112W	HOS4	0.3750	-0.0329
YDL111C	RRP42	YKL110C	KTI12	0.3749	0.0994
YGL192W	IME4	YFL016C	MDJ1	0.3749	0.1090
YOL094C	RFC4	YMR123W	PKR1	0.3749	0.1590
YBR137W	YBR137W	YJL187C	SWE1	0.3747	-0.1492
YDR144C	MKC7	YLR375W	STP3	0.3744	-0.0935
YHR034C	PIH1	YER046W	SPO73	0.3744	0.0337
YJR073C	OPI3	YPL234C	VMA11	0.3744	0.0010
YKL160W	ELF1	YDR420W	HKR1	0.3744	-0.1207
YBR193C	MED8	YPL058C	PDR12	0.3743	-0.0648
YIL040W	APQ12	YER116C	SLX8	0.3742	-0.1122
YJL212C	OPT1	YLL061W	MMP1	0.3741	0.0648
YJL154C	VPS35	YER037W	PHM8	0.3739	0.1063
YMR112C	MED11	YPL144W	POC4	0.3739	0.1701
YOL018C	TLG2	YNL041C	COG6	0.3739	0.0157
YER046W	SPO73	YIL119C	RPI1	0.3738	-0.0248
YMR154C	RIM13	YML013W	UBX2	0.3738	0.1164
YLR047C	FRE8	YML079W	YML079W	0.3736	-0.0709
YJR040W	GEF1	YDR420W	HKR1	0.3735	0.0729
YFL016C	MDJ1	YLR330W	CHS5	0.3734	0.1452
YNR012W	URK1	YJR097W	JJJ3	0.3734	0.2188
YOL147C	PEX11	YEL076W-C	Unknown	0.3734	0.0858
YPL016W	SWI1	YIL043C	CBR1	0.3733	-0.0787
YER144C	UBP5	YMR209C	YMR209C	0.3731	-0.1215
YBR154C	RPB5	YBL020W	RFT1	0.3730	0.2170
YLR231C	BNA5	YLR443W	ECM7	0.3730	-0.1153
YFL056C	AAD6	YKL184W	SPE1	0.3729	0.0864
YGL053W	PRM8	YJL047C	RTT101	0.3729	0.0684
YKL019W	RAM2	YLR046C	YLR046C	0.3729	0.0722
YOR054C	VHS3	YDR351W	SBE2	0.3728	0.1104
YER185W	PUG1	YLR368W	MDM30	0.3727	0.0679
YCL026C-A	FRM2	YFL018C	LPD1	0.3726	-0.1989
YER019W	ISC1	YOR051C	ETT1	0.3726	-0.0439

YLR047C	FRE8	YER020W	GPA2	0.3726	0.0741
YBR193C	MED8	YDR530C	APA2	0.3725	0.0701
YER022W	SRB4	YOL018C	TLG2	0.3725	0.1568
YDL047W	SIT4	YBR161W	CSH1	0.3724	0.1676
YHL007C	STE20	YEL076W-C	Unknown	0.3724	0.3705
YOR196C	LIP5	YOR101W	RAS1	0.3724	-0.1573
YER022W	SRB4	YJL140W	RPB4	0.3721	0.0122
YBR163W	EXO5	YBR061C	TRM7	0.3718	0.0736
YFR013W	IOC3	YGR006W	PRP18	0.3718	0.0940
YBR222C	PCS60	YBR196C	PGI1	0.3717	0.2413
YJR068W	RFC2	YML007W	YAP1	0.3717	-0.2048
YML110C	COQ5	YER144C	UBP5	0.3717	-0.1895
YLR287C	YLR287C	YLL035W	GRC3	0.3715	0.3155
YBR168W	PEX32	YEL064C	AVT2	0.3714	0.0900
YMR158W	MRPS8	YDL133W	SRF1	0.3714	-0.2231
YDL147W	RPN5	YNR036C	MRPS12	0.3713	-0.0876
YDL135C	RDI1	YJR040W	GEF1	0.3711	-0.0131
YLR096W	KIN2	YIL171W	YIL171W	0.3711	-0.0003
YJL187C	SWE1	YLR342W	FKS1	0.3710	0.1286
YLR231C	BNA5	YLR442C	SIR3	0.3710	-0.0862
YML004C	GLO1	YIL119C	RPI1	0.3710	-0.1333
YPL234C	VMA11	YKL183W	LOT5	0.3710	0.1257
YLR180W	SAM1	YDR144C	MKC7	0.3709	0.0577
YML096W	YML096W	YKL125W	RRN3	0.3709	0.1334
YEL020W-A	TIM9	YGR222W	PET54	0.3708	0.1653
YFL016C	MDJ1	YLR452C	SST2	0.3707	-0.1404
YFR047C	BNA6	YBR168W	PEX32	0.3707	0.2120
YIL112W	HOS4	YIL165C	YIL165C	0.3707	0.0064
YJL058C	BIT61	YOR051C	ETT1	0.3706	0.0605
YDL147W	RPN5	YPL009C	RQC2	0.3705	0.1489
YIL112W	HOS4	YNL200C	NNR1	0.3705	0.0746
YLR464W	YLR464W	YBR168W	PEX32	0.3705	-0.0582
YGL051W	MST27	YML007W	YAP1	0.3703	0.0789
YDR441C	APT2	YML036W	CGI121	0.3700	0.0758
YML079W	YML079W	YOR033C	EXO1	0.3700	-0.1623
YJR008W	MHO1	YPL012W	RRP12	0.3699	-0.2376
YIL043C	CBR1	YGR258C	RAD2	0.3697	-0.2023
YCR071C	IMG2	YIL112W	HOS4	0.3695	-0.1332
YGR289C	MAL11	YLR258W	GSY2	0.3692	0.0073
YDL002C	NHP10	YDR531W	CAB1	0.3691	0.0908
YFL047W	RGD2	YBL040C	ERD2	0.3691	-0.0966
YGL035C	MIG1	YJL149W	DAS1	0.3690	0.0481
YJL154C	VPS35	YPL001W	HAT1	0.3690	-0.0701
YOR092W	ECM3	YEL058W	PCM1	0.3690	-0.0776
YOR101W	RAS1	YNL280C	ERG24	0.3689	0.1170
YIL166C	YIL166C	YLL061W	MMP1	0.3687	-0.0013
YGL004C	RPN14	YDL155W	CLB3	0.3686	0.0259

YGR152C	RSR1	YBR071W	YBR071W	0.3686	0.0276
YJL178C	ATG27	YFL047W	RGD2	0.3686	-0.2243
YLR057W	MNL2	YOL088C	MPD2	0.3685	0.1002
YMR112C	MED11	YAR027W	UIP3	0.3684	-0.0673
YAL056W	GPB2	YGL207W	SPT16	0.3682	-0.0976
YML048W	GSF2	YMR067C	UBX4	0.3682	0.0016
YOL097C	WRS1	YOL094C	RFC4	0.3679	0.2807
YOR043W	WHI2	YIL015C-A	YIL015C-A	0.3679	-0.1626
YGL257C	MNT2	YBR061C	TRM7	0.3678	-0.0026
YJL178C	ATG27	YDR319C	YFT2	0.3675	-0.2044
YJL047C	RTT101	YNL041C	COG6	0.3675	0.0553
YJL118W	YJL118W	YBL014C	RRN6	0.3674	0.0364
YNR034W	SOL1	YBR249C	ARO4	0.3673	0.0422
YOL104C	NDJ1	YPL058C	PDR12	0.3671	0.0708
YCL024W	KCC4	YJL187C	SWE1	0.3670	0.1789
YPR022C	SDD4	YJL149W	DAS1	0.3670	0.0711
YFR013W	IOC3	YJR103W	URA8	0.3669	-0.1064
YJL118W	YJL118W	YGR288W	MAL13	0.3669	-0.0389
YML007W	YAP1	YDR515W	SLF1	0.3669	0.0689
YOR127W	RGA1	YPL234C	VMA11	0.3669	-0.0207
YOR051C	ETT1	YBL079W	NUP170	0.3668	-0.1310
YJL178C	ATG27	YPL016W	SWI1	0.3667	-0.1755
YKL073W	LHS1	YOL088C	MPD2	0.3667	0.1132
YML007W	YAP1	YJR073C	OPI3	0.3667	-0.0324
YDR319C	YFT2	YDL217C	TIM22	0.3666	0.1197
YJL154C	VPS35	YOR371C	GPB1	0.3664	0.1254
YAR027W	UIP3	YGR006W	PRP18	0.3663	-0.0055
YGL208W	SIP2	YGL207W	SPT16	0.3658	-0.1717
YMR112C	MED11	YGL208W	SIP2	0.3658	-0.0870
YBL020W	RFT1	YBR154C	RPB5	0.3657	0.1279
YJL178C	ATG27	YPL237W	SUI3	0.3655	0.0433
YJR056C	YJR056C	YEL076W-C	Unknown	0.3655	-0.1577
YML110C	COQ5	YJR008W	MHO1	0.3654	0.1303
YFL049W	SWP82	YOR101W	RAS1	0.3653	-0.0748
YGL257C	MNT2	YOL081W	IRA2	0.3652	-0.0042
YNL326C	PFA3	YDR134C	YDR134C	0.3652	0.0609
YML091C	RPM2	YLR237W	THI7	0.3650	-0.1951
YJL204C	RCY1	YLR368W	MDM30	0.3649	-0.2339
YKL019W	RAM2	YJL204C	RCY1	0.3644	0.0947
YGL242C	YGL242C	YGL207W	SPT16	0.3643	0.0476
YLL028W	TPO1	YJL183W	MNN11	0.3643	0.0745
YOR251C	TUM1	YNL024C	EFM6	0.3643	0.1073
YBL105C	PKC1	YJR097W	JJJ3	0.3642	-0.0029
YIL046W	MET30	YOL113W	SKM1	0.3642	0.1179
YOR054C	VHS3	YJL047C	RTT101	0.3642	0.1258
YBR157C	ICS2	YLL061W	MMP1	0.3641	-0.0680
YLR426W	TDA5	YHR023W	MYO1	0.3639	-0.0650

YER037W	PHM8	YJL171C	TOH1	0.3638	0.1256
YBR137W	YBR137W	YLR046C	YLR046C	0.3636	-0.2237
YCR041W	YCR041W	YLR057W	MNL2	0.3635	0.0334
YDR134C	YDR134C	YOR196C	LIP5	0.3635	-0.0697
YEL056W	HAT2	YJL051W	IRC8	0.3634	0.1960
YBR166C	TYR1	YGL226W	MTC3	0.3630	-0.0078
YGR288W	MAL13	YDL164C	CDC9	0.3630	-0.1381
YPL016W	SWI1	YJR086W	STE18	0.3630	-0.1385
YFL018C	LPD1	YOR196C	LIP5	0.3629	0.0130
YOL089C	HAL9	YKL096W	CWP1	0.3629	-0.0750
YIR022W	SEC11	YBR249C	ARO4	0.3628	-0.0735
YGR157W	CHO2	YML098W	TAF13	0.3626	-0.0864
YJL118W	YJL118W	YLR389C	STE23	0.3626	-0.0658
YCL039W	GID7	YJR086W	STE18	0.3625	0.0348
YDR144C	MKC7	YKL116C	PRR1	0.3625	0.0186
YMR215W	GAS3	YJL183W	MNN11	0.3620	0.1378
YGR006W	PRP18	YJR103W	URA8	0.3619	0.0039
YBR037C	SCO1	YEL058W	PCM1	0.3617	-0.2293
YIL089W	YIL089W	YBR274W	CHK1	0.3617	0.0219
YER144C	UBP5	YBR229C	ROT2	0.3616	-0.0556
YER150W	SPI1	YFR047C	BNA6	0.3616	0.1764
YNR012W	URK1	YPL012W	RRP12	0.3616	0.0618
YMR005W	TAF4	YJR074W	MOG1	0.3611	0.0647
YOL092W	YPQ1	YML098W	TAF13	0.3610	0.1329
YCR083W	TRX3	YOL147C	PEX11	0.3609	0.0915
YIR028W	DAL4	YGR288W	MAL13	0.3609	-0.0645
YBL014C	RRN6	YBL007C	SLA1	0.3607	0.1233
YJL178C	ATG27	YML098W	TAF13	0.3605	0.0672
YPL147W	PXA1	YPL001W	HAT1	0.3604	-0.1508
YIL043C	CBR1	YHL048W	COS8	0.3603	-0.1611
YOL088C	MPD2	YJL118W	YJL118W	0.3603	-0.1476
YMR215W	GAS3	YML110C	COQ5	0.3601	0.2512
YER037W	PHM8	YEL071W	DLD3	0.3597	-0.1658
YBR274W	CHK1	YMR152W	YIM1	0.3596	-0.0933
YFL047W	RGD2	YDR180W	SCC2	0.3594	-0.1536
YPL016W	SWI1	YBL040C	ERD2	0.3594	-0.1129
YOR144C	ELG1	YOL088C	MPD2	0.3593	-0.2444
YGR288W	MAL13	YML036W	CGI121	0.3592	-0.1521
YFR004W	RPN11	YOL018C	TLG2	0.3591	0.0109
YGR150C	CCM1	YPL147W	PXA1	0.3591	0.0470
YHR115C	DMA1	YGR222W	PET54	0.3591	-0.0171
YGR012W	MCY1	YDR058C	TGL2	0.3590	0.0002
YBL030C	PET9	YLR442C	SIR3	0.3589	-0.0446
YKL096W	CWP1	YBL105C	PKC1	0.3588	-0.0394
YBL014C	RRN6	YJL118W	YJL118W	0.3584	0.1039
YIL015C-A	YIL015C-A	YKL092C	BUD2	0.3584	-0.0860
YOL093W	TRM10	YKL021C	MAK11	0.3584	0.1427

YHR027C	RPN1	YGR288W	MAL13	0.3583	-0.1002
YBL020W	RFT1	YLR176C	RFX1	0.3581	-0.0639
YMR046C	YMR046C	YLR442C	SIR3	0.3581	-0.0282
YGL201C	MCM6	YKL110C	KTI12	0.3580	0.0284
YGR012W	MCY1	YOL113W	SKM1	0.3579	0.1438
YKL092C	BUD2	YHR023W	MYO1	0.3578	0.0799
YDL132W	CDC53	YJL212C	OPT1	0.3577	-0.0835
YNL215W	IES2	YJL178C	ATG27	0.3577	0.0755
YDR064W	RPS13	YNL135C	FPR1	0.3574	0.0909
YGR157W	CHO2	YGL257C	MNT2	0.3574	0.1112
YGL004C	RPN14	YMR115W	MGR3	0.3572	-0.1538
YJL187C	SWE1	YGR152C	RSR1	0.3572	0.1865
YKL067W	YNK1	YCR083W	TRX3	0.3569	0.0069
YBR168W	PEX32	YKL092C	BUD2	0.3567	0.0780
YDR528W	HLR1	YNL321W	VNX1	0.3563	-0.1675
YHR023W	MYO1	YLL040C	VPS13	0.3563	0.0087
YMR209C	YMR209C	YJL171C	TOH1	0.3562	-0.0923
YAL056W	GPB2	YBL040C	ERD2	0.3561	-0.0970
YGR184C	UBR1	YLL040C	VPS13	0.3561	0.0263
YKL092C	BUD2	YER046W	SPO73	0.3561	0.0317
YNL074C	MLF3	YBR168W	PEX32	0.3561	-0.0776
YDR420W	HKR1	YOL026C	MIM1	0.3560	-0.0253
YGR194C	XKS1	YGR222W	PET54	0.3558	0.3154
YOL147C	PEX11	YLR046C	YLR046C	0.3558	0.2366
YNL215W	IES2	YPL064C	CWC27	0.3554	0.0308
YOR389W	YOR389W	YFR037C	RSC8	0.3552	-0.0249
YML038C	YMD8	YOL092W	YPQ1	0.3551	0.0598
YGR289C	MAL11	YGL051W	MST27	0.3549	0.0813
YBL030C	PET9	YNL321W	VNX1	0.3548	0.0000
YMR123W	PKR1	YGR001C	EFM5	0.3548	0.0013
YGR288W	MAL13	YBR087W	RFC5	0.3547	-0.0779
YIL165C	YIL165C	YJR025C	BNA1	0.3546	0.0740
YDL047W	SIT4	YOR142W	LSC1	0.3545	-0.0547
YGL193C	YGL193C	YCL024W	KCC4	0.3544	0.0429
YLR064W	PER33	YML079W	YML079W	0.3542	0.0340
YGR184C	UBR1	YLR052W	IES3	0.3541	-0.0887
YCL009C	ILV6	YBR222C	PCS60	0.3540	0.0930
YHR039C	MSC7	YLR096W	KIN2	0.3540	0.1348
YML036W	CGI121	YMR209C	YMR209C	0.3540	0.2056
YOL113W	SKM1	YLR426W	TDA5	0.3539	0.1590
YBR067C	TIP1	YEL068C	YEL068C	0.3536	0.0772
YGR150C	CCM1	YOL018C	TLG2	0.3535	0.0294
YDR497C	ITR1	YLR057W	MNL2	0.3534	-0.0396
YER144C	UBP5	YML110C	COQ5	0.3534	-0.0734
YDL103C	QRI1	YGR288W	MAL13	0.3531	-0.0593
YJR086W	STE18	YLL024C	SSA2	0.3531	0.1308
YHR054C	YHR054C	YEL064C	AVT2	0.3530	0.0714

YAL051W	OAF1	YOL092W	YPQ1	0.3526	-0.0390
YFR013W	IOC3	YCL009C	ILV6	0.3526	-0.0218
YDL089W	NUR1	YEL068C	YEL068C	0.3525	0.0633
YKL096W	CWP1	YHR063C	PAN5	0.3524	-0.1304
YER019W	ISC1	YLR248W	RCK2	0.3523	-0.0870
YCR031C	RPS14a	YJL183W	MNN11	0.3522	0.1427
YEL058W	PCM1	YPL058C	PDR12	0.3522	0.0699
YGR194C	XKS1	YNL035C	YNL035C	0.3522	-0.2750
YBR274W	CHK1	YMR005W	TAF4	0.3521	0.0545
YFR042W	KEG1	YOR103C	OST2	0.3520	0.0041
YGR207C	CIR1	YKL092C	BUD2	0.3520	0.2168
YNL138W	SRV2	YGL242C	YGL242C	0.3520	0.1425
YIL165C	YIL165C	YIL112W	HOS4	0.3516	0.1058
YJL047C	RTT101	YLR237W	THI7	0.3516	-0.1885
YIL015C-A	YIL015C-A	YIL061C	SNP1	0.3515	-0.0275
YPL009C	RQC2	YBR225W	YBR225W	0.3515	-0.1192
YJL158C	CIS3	YPL226W	NEW1	0.3514	0.0617
YIL066C	RNR3	YML004C	GLO1	0.3512	0.0624
YLR452C	SST2	YFL016C	MDJ1	0.3512	-0.1163
YPL016W	SWI1	YML036W	CGI121	0.3512	-0.0088
YFR042W	KEG1	YMR115W	MGR3	0.3511	-0.1292
YPL071C	YPL071C	YGR006W	PRP18	0.3511	0.0424
YJL058C	BIT61	YLL035W	GRC3	0.3510	0.0171
YML007W	YAP1	YKL067W	YNK1	0.3510	-0.1224
YMR005W	TAF4	YHR063C	PAN5	0.3510	-0.1498
YDR420W	HKR1	YBL030C	PET9	0.3508	0.1547
YIL009W	FAA3	YNL034W	YNL034W	0.3507	-0.0611
YJL051W	IRC8	YJL187C	SWE1	0.3506	0.0322
YML038C	YMD8	YDR497C	ITR1	0.3502	0.0587
YDR178W	SDH4	YHR050W	SMF2	0.3501	0.2689
YDR383C	NKP1	YNL322C	KRE1	0.3501	0.0358
YNL074C	MLF3	YGR208W	SER2	0.3500	0.1615
YJR040W	GEF1	YLR206W	ENT2	0.3498	0.0048
YGR208W	SER2	YGR209C	TRX2	0.3493	0.1047
YPR015C	YPR015C	YOL018C	TLG2	0.3493	-0.0636
YJR084W	YJR084W	YBR274W	CHK1	0.3492	0.0549
YMR209C	YMR209C	YHR023W	MYO1	0.3492	-0.0789
YDL171C	GLT1	YIL009W	FAA3	0.3490	0.0727
YBR137W	YBR137W	YER046W	SPO73	0.3487	0.0539
YIL166C	YIL166C	YNL041C	COG6	0.3486	0.0033
YJL208C	NUC1	YML004C	GLO1	0.3486	-0.1667
YER186C	YER186C	YGR001C	EFM5	0.3483	0.0359
YDR180W	SCC2	YKL021C	MAK11	0.3482	0.2316
YHL048W	COS8	YFR013W	IOC3	0.3482	0.1618
YEL071W	DLD3	YNL280C	ERG24	0.3481	-0.1821
YER037W	PHM8	YBR147W	RTC2	0.3481	0.0845
YLR172C	DPH5	YMR123W	PKR1	0.3481	0.1443

YBR126C	TPS1	YML072C	TCB3	0.3480	0.2783
YCR031C	RPS14a	YNL135C	FPR1	0.3479	0.1769
YDR134C	YDR134C	YKL110C	KTI12	0.3479	-0.0493
YGL207W	SPT16	YDR420W	HKR1	0.3479	0.3112
YJR074W	MOG1	YML091C	RPM2	0.3479	-0.0778
YDL133W	SRF1	YER046W	SPO73	0.3478	0.1772
YGR157W	CHO2	YNL215W	IES2	0.3478	-0.2843
YOR131C	YOR131C	YPL064C	CWC27	0.3477	0.1276
YBL029W	YBL029W	YGR258C	RAD2	0.3475	0.1024
YCL018W	LEU2	YGL257C	MNT2	0.3475	0.1335
YMR209C	YMR209C	YFR013W	IOC3	0.3473	-0.1177
YOR101W	RAS1	YML013W	UBX2	0.3473	-0.2264
YOR131C	YOR131C	YGR258C	RAD2	0.3473	-0.0542
YML038C	YMD8	YOR196C	LIP5	0.3472	0.0972
YNL322C	KRE1	YFR004W	RPN11	0.3472	0.1934
YOL164W	BDS1	YJL149W	DAS1	0.3469	0.0712
YJR040W	GEF1	YLR443W	ECM7	0.3466	0.0813
YBR008C	FLR1	YER022W	SRB4	0.3465	-0.1418
YGL178W	MPT5	YBR196C	PGI1	0.3465	0.0281
YGR222W	PET54	YGR001C	EFM5	0.3465	0.0965
YML098W	TAF13	YJR074W	MOG1	0.3465	0.0694
YOR251C	TUM1	YDR180W	SCC2	0.3463	0.0975
YBL020W	RFT1	YJR032W	CPR7	0.3462	-0.2169
YOL092W	YPQ1	YDL111C	RRP42	0.3462	0.0003
YJR036C	HUL4	YGL035C	MIG1	0.3461	-0.0093
YGL226C-A	OST5	YDR481C	PHO8	0.3460	-0.0907
YGL178W	MPT5	YNR034W	SOL1	0.3460	-0.0669
YFL049W	SWP82	YML098W	TAF13	0.3459	0.1938
YDR321W	ASP1	YLL024C	SSA2	0.3458	0.2065
YFR011C	MIC19	YNL321W	VNX1	0.3456	0.1926
YBR274W	CHK1	YER035W	EDC2	0.3455	-0.0365
YGR012W	MCY1	YBR161W	CSH1	0.3455	-0.1510
YHR050W	SMF2	YJL187C	SWE1	0.3453	0.0248
YLR155C	ASP3-1	YIL158W	AIM20	0.3452	-0.0066
YIL165C	YIL165C	YKL116C	PRR1	0.3449	-0.2459
YJL036W	SNX4	YJR008W	MHO1	0.3446	0.1074
YBR196C	PGI1	YML071C	COG8	0.3444	-0.0313
YGL193C	YGL193C	YKL116C	PRR1	0.3444	-0.0325
YIL133C	RPL16a	YDR144C	MKC7	0.3443	0.2724
YKR085C	MRPL20	YNL046W	YNL046W	0.3443	-0.1945
YOR251C	TUM1	YNL046W	YNL046W	0.3443	0.1038
YER019W	ISC1	YLR176C	RFX1	0.3442	0.1063
YEL076W-C	Unknown	YHR063C	PAN5	0.3441	-0.0185
YJR084W	YJR084W	YDR383C	NKP1	0.3441	0.0323
YBR163W	EXO5	YBR121C	GRS1	0.3440	0.0576
YJL204C	RCY1	YLR206W	ENT2	0.3439	-0.0530
YCL026C-A	FRM2	YLR455W	PDP3	0.3438	-0.1218

YJL187C	SWE1	YOR144C	ELG1	0.3437	0.0716
YDR351W	SBE2	YML038C	YMD8	0.3436	0.1006
YMR154C	RIM13	YDR383C	NKP1	0.3435	0.1047
YDL116W	NUP84	YML098W	TAF13	0.3434	-0.0362
YGR168C	YGR168C	YBR161W	CSH1	0.3434	0.0416
YIL040W	APQ12	YGL226C-A	OST5	0.3434	0.1208
YJL217W	REE1	YIL061C	SNP1	0.3434	-0.0097
YPL001W	HAT1	YDR178W	SDH4	0.3434	-0.0525
YJL154C	VPS35	YGR258C	RAD2	0.3432	-0.0003
YDR524C	AGE1	YLR257W	YLR257W	0.3431	0.0186
YMR067C	UBX4	YGL169W	SUA5	0.3429	0.2048
YGR211W	ZPR1	YNL200C	NNR1	0.3428	-0.0277
YER046W	SPO73	YHR034C	PIH1	0.3427	0.0054
YGL169W	SUA5	YOR088W	YOR088W	0.3427	0.1261
YIL111W	COX5b	YPL009C	RQC2	0.3427	-0.1568
YBR154C	RPB5	YBR087W	RFC5	0.3426	0.0342
YCR083W	TRX3	YAL009W	SPO7	0.3425	0.1332
YJR032W	CPR7	YKL184W	SPE1	0.3425	0.1545
YKR085C	MRPL20	YNL074C	MLF3	0.3424	-0.2494
YLR096W	KIN2	YJL047C	RTT101	0.3423	0.0958
YOR371C	GPB1	YKL183W	LOT5	0.3421	0.1616
YJR040W	GEF1	YMR115W	MGR3	0.3420	-0.0575
YPL031C	PHO85	YDR513W	GRX2	0.3420	0.0288
YEL058W	PCM1	YHR043C	DOG2	0.3416	0.0524
YBR196C	PGI1	YGL226W	MTC3	0.3415	-0.0581
YIR022W	SEC11	YGL195W	GCN1	0.3414	0.0868
YBL029W	YBL029W	YML071C	COG8	0.3413	-0.0052
YCR083W	TRX3	YML091C	RPM2	0.3412	-0.0959
YKL160W	ELF1	YGL192W	IME4	0.3411	-0.1119
YBR147W	RTC2	YAL056W	GPB2	0.3410	0.1927
YBR239C	ERT1	YBR166C	TYR1	0.3410	0.0838
YML098W	TAF13	YDL116W	NUP84	0.3410	-0.0975
YKL019W	RAM2	YNL041C	COG6	0.3407	-0.1071
YGL208W	SIP2	YOL147C	PEX11	0.3406	0.1046
YPL233W	NSL1	YFL018C	LPD1	0.3406	-0.0054
YOL088C	MPD2	YOR092W	ECM3	0.3405	0.0001
YOL081W	IRA2	YJL126W	NIT2	0.3405	-0.0653
YJR008W	MHO1	YOL130W	ALR1	0.3403	-0.1635
YOL113W	SKM1	YOL018C	TLG2	0.3402	0.0893
YFR004W	RPN11	YLR330W	CHS5	0.3401	0.1337
YGL004C	RPN14	YPL064C	CWC27	0.3401	-0.0258
YGR288W	MAL13	YJL187C	SWE1	0.3401	-0.1892
YHR027C	RPN1	YLR426W	TDA5	0.3401	-0.2109
YGR001C	EFM5	YER186C	YER186C	0.3400	0.0406
YJR036C	HUL4	YJL036W	SNX4	0.3400	0.1765
YLR096W	KIN2	YOL088C	MPD2	0.3400	-0.1680
YGL193C	YGL193C	YHL007C	STE20	0.3399	-0.0503

YIL066C	RNR3	YLR426W	TDA5	0.3399	-0.0953
YER049W	TPA1	YIL142W	CCT2	0.3396	0.2509
YKL019W	RAM2	YOR051C	ETT1	0.3396	-0.1049
YGL192W	IME4	YKL110C	KTI12	0.3395	-0.0666
YOL062C	APM4	YIR026C	YVH1	0.3394	-0.1087
YOR144C	ELG1	YLR435W	TSR2	0.3394	0.2177
YOR092W	ECM3	YHR023W	MYO1	0.3393	-0.1156
YAL009W	SPO7	YBR077C	SLM4	0.3390	0.0007
YJL126W	NIT2	YDL103C	QRI1	0.3390	0.0680
YJL118W	YJL118W	YGL017W	ATE1	0.3390	-0.0365
YOR054C	VHS3	YDR144C	MKC7	0.3390	-0.1053
YBR229C	ROT2	YGR152C	RSR1	0.3389	-0.1004
YKL116C	PRR1	YGR168C	YGR168C	0.3388	0.1345
YOR131C	YOR131C	YGL169W	SUA5	0.3387	-0.1160
YGR157W	CHO2	YKL116C	PRR1	0.3385	0.1473
YHL048W	COS8	YHR054C	YHR054C	0.3385	0.0868
YLR375W	STP3	YDL103C	QRI1	0.3384	-0.1096
YDR531W	CAB1	YMR067C	UBX4	0.3383	-0.1145
YNR034W	SOL1	YLR248W	RCK2	0.3383	-0.0897
YBR161W	CSH1	YGR012W	MCY1	0.3382	-0.0497
YEL056W	HAT2	YIL061C	SNP1	0.3382	-0.2116
YLR452C	SST2	YDR058C	TGL2	0.3382	-0.0683
YKL021C	MAK11	YPL183C	RTT10	0.3379	0.0960
YLR443W	ECM7	YDR441C	APT2	0.3377	-0.0349
YDR441C	APT2	YMR152W	YIM1	0.3376	-0.0128
YKR075C	YKR075C	YOL026C	MIM1	0.3376	-0.0831
YML110C	COQ5	YJR032W	CPR7	0.3373	0.0843
YBR087W	RFC5	YLR176C	RFX1	0.3371	-0.1386
YER116C	SLX8	YGL178W	MPT5	0.3371	0.1745
YFL016C	MDJ1	YOL088C	MPD2	0.3369	-0.1227
YIL040W	APQ12	YMR046C	YMR046C	0.3369	-0.0495
YOL093W	TRM10	YDL002C	NHP10	0.3369	0.2100
YOL088C	MPD2	YGR168C	YGR168C	0.3369	0.1172
YLR052W	IES3	YLR455W	PDP3	0.3368	0.0484
YDR058C	TGL2	YGR157W	CHO2	0.3367	-0.1224
YKL019W	RAM2	YOR117W	RPT5	0.3367	0.0770
YPL144W	POC4	YOR142W	LSC1	0.3367	0.0957
YGR149W	GPC1	YJR008W	MHO1	0.3366	0.1130
YDL155W	CLB3	YLL024C	SSA2	0.3364	0.0471
YDL133W	SRF1	YLR046C	YLR046C	0.3364	0.1310
YER023W	PRO3	YJL158C	CIS3	0.3364	0.2692
YIL061C	SNP1	YDR515W	SLF1	0.3364	0.0686
YCL018W	LEU2	YLL024C	SSA2	0.3363	0.1204
YDR134C	YDR134C	YGR023W	MTL1	0.3361	0.0562
YKL096W	CWP1	YDL217C	TIM22	0.3360	-0.1458
YDR367W	KEI1	YBR071W	YBR071W	0.3356	-0.0325
YPL058C	PDR12	YLR455W	PDP3	0.3356	-0.1542

YBR061C	TRM7	YBR166C	TYR1	0.3355	0.1761
YIL111W	COX5b	YNL034W	YNL034W	0.3354	-0.0620
YKL021C	MAK11	YGR028W	MSP1	0.3354	-0.3002
YGL053W	PRM8	YER035W	EDC2	0.3353	0.0444
YBR071W	YBR071W	YBL040C	ERD2	0.3352	0.0062
YDR058C	TGL2	YLL024C	SSA2	0.3352	-0.1481
YFR013W	IOC3	YOR131C	YOR131C	0.3352	0.0456
YLR452C	SST2	YKL116C	PRR1	0.3352	0.1071
YMR005W	TAF4	YER046W	SPO73	0.3352	-0.0122
YDL211C	YDL211C	YMR005W	TAF4	0.3351	-0.0163
YDL103C	QRI1	YGR006W	PRP18	0.3351	0.1708
YER022W	SRB4	YKL021C	MAK11	0.3351	0.0553
YLL061W	MMP1	YER035W	EDC2	0.3351	-0.0640
YLL024C	SSA2	YJR086W	STE18	0.3351	0.1445
YOL089C	HAL9	YGL226W	MTC3	0.3351	-0.0896
YBR071W	YBR071W	YBR161W	CSH1	0.3350	0.0038
YGR149W	GPC1	YGR194C	XKS1	0.3350	0.0424
YNL329C	PEX6	YDL150W	RPC53	0.3350	-0.0752
YIL046W	MET30	YHR043C	DOG2	0.3349	-0.1135
YAR028W	YAR028W	YBR239C	ERT1	0.3348	-0.0177
YLR389C	STE23	YAL009W	SPO7	0.3348	-0.0231
YGR288W	MAL13	YNL041C	COG6	0.3346	0.2265
YKR052C	MRS4	YLR096W	KIN2	0.3346	-0.0001
YJL058C	BIT61	YLR206W	ENT2	0.3342	-0.0245
YCL024W	KCC4	YOR264W	DSE3	0.3341	0.1961
YDR513W	GRX2	YNL329C	PEX6	0.3341	-0.1329
YFR037C	RSC8	YGL035C	MIG1	0.3338	0.1486
YHR016C	YSC84	YDR339C	FCF1	0.3337	-0.0757
YML110C	COQ5	YPL234C	VMA11	0.3337	0.1023
YER023W	PRO3	YML013W	UBX2	0.3336	0.1554
YHL048W	COS8	YFR047C	BNA6	0.3335	0.0922
YLR181C	VTA1	YIL166C	YIL166C	0.3335	0.2330
YPL031C	PHO85	YJL030W	MAD2	0.3335	0.0039
YIL061C	SNP1	YDR497C	ITR1	0.3334	0.0651
YLR248W	RCK2	YBR168W	PEX32	0.3334	0.1098
YHL048W	COS8	YGR001C	EFM5	0.3333	0.1298
YML038C	YMD8	YHR043C	DOG2	0.3333	-0.1734
YEL068C	YEL068C	YBR274W	CHK1	0.3330	0.1069
YOL018C	TLG2	YNL035C	YNL035C	0.3329	0.1608
YDR531W	CAB1	YFL056C	AAD6	0.3328	0.2299
YJL218W	YJL218W	YKL125W	RRN3	0.3326	0.0010
YOR051C	ETT1	YDL002C	NHP10	0.3322	0.1276
YGL004C	RPN14	YPL001W	HAT1	0.3319	0.0529
YFR013W	IOC3	YFL016C	MDJ1	0.3316	0.0044
YJR036C	HUL4	YGR258C	RAD2	0.3315	0.0961
YJL058C	BIT61	YKL073W	LHS1	0.3313	0.0488
YHR115C	DMA1	YJR068W	RFC2	0.3311	-0.0155

YJL212C	OPT1	YJL051W	IRC8	0.3311	0.1309
YJR025C	BNA1	YER144C	UBP5	0.3311	-0.1011
YJL149W	DAS1	YAL009W	SPO7	0.3310	-0.0396
YJR097W	JJJ3	YBL105C	PKC1	0.3309	-0.1134
YIL166C	YIL166C	YDL164C	CDC9	0.3308	-0.0044
YOR054C	VHS3	YGR150C	CCM1	0.3307	0.0334
YHR039C	MSC7	YGR149W	GPC1	0.3306	0.1716
YBR087W	RFC5	YBR161W	CSH1	0.3303	0.1143
YER019W	ISC1	YJR040W	GEF1	0.3303	0.0144
YJR036C	HUL4	YIL040W	APQ12	0.3302	-0.0332
YML091C	RPM2	YJR086W	STE18	0.3301	0.1022
YIL165C	YIL165C	YJL158C	CIS3	0.3300	-0.0545
YOL130W	ALR1	YDR524C	AGE1	0.3300	0.1539
YEL021W	URA3	YOL088C	MPD2	0.3299	-0.0668
YML007W	YAP1	YFR013W	IOC3	0.3299	-0.0026
YJL208C	NUC1	YIR022W	SEC11	0.3298	-0.0157
YJL212C	OPT1	YOR101W	RAS1	0.3296	0.0711
YOL147C	PEX11	YOR043W	WHI2	0.3296	-0.0294
YBR077C	SLM4	YOL062C	APM4	0.3295	0.0964
YGL025C	PGD1	YGR168C	YGR168C	0.3295	0.0842
YDL164C	CDC9	YGL207W	SPT16	0.3294	0.1128
YPL234C	VMA11	YDL147W	RPN5	0.3294	-0.0563
YHR023W	MYO1	YLR375W	STP3	0.3291	-0.1466
YOR043W	WHI2	YGR006W	PRP18	0.3291	0.0600
YDL103C	QRI1	YJL126W	NIT2	0.3289	0.1476
YGR157W	CHO2	YOR051C	ETT1	0.3289	0.1360
YKR080W	MTD1	YHR034C	PIH1	0.3289	-0.0931
YGL053W	PRM8	YML048W	GSF2	0.3288	-0.0127
YDR528W	HLR1	YBR137W	YBR137W	0.3287	0.0411
YIL046W	MET30	YBR121C	GRS1	0.3287	-0.0645
YJL030W	MAD2	YFR004W	RPN11	0.3287	0.1281
YLR176C	RFX1	YBL020W	RFT1	0.3287	-0.0015
YJL149W	DAS1	YER144C	UBP5	0.3286	0.0342
YJR032W	CPR7	YIL158W	AIM20	0.3286	0.1070
YDL171C	GLT1	YBL040C	ERD2	0.3285	-0.1073
YOR251C	TUM1	YPL144W	POC4	0.3284	0.2893
YDL180W	YDL180W	YLR057W	MNL2	0.3283	0.0302
YFR047C	BNA6	YHR054C	YHR054C	0.3283	0.0516
YLR047C	FRE8	YJL163C	YJL163C	0.3283	0.0864
YLR181C	VTA1	YLR455W	PDP3	0.3282	0.1193
YLR056W	ERG3	YJR074W	MOG1	0.3274	0.0844
YNL242W	ATG2	YOL147C	PEX11	0.3274	0.0488
YDR524C	AGE1	YIL009W	FAA3	0.3272	0.1538
YNL124W	NAF1	YMR005W	TAF4	0.3272	0.0582
YIL111W	COX5b	YBL020W	RFT1	0.3271	-0.0581
YLL040C	VPS13	YOL062C	APM4	0.3271	-0.0669
YDL155W	CLB3	YBR157C	ICS2	0.3270	-0.0027

YDR367W	KEI1	YKR051W	YKR051W	0.3270	0.0684
YOL089C	HAL9	YNL135C	FPR1	0.3270	-0.0897
YOL026C	MIM1	YEL055C	POL5	0.3270	0.0012
YEL020W-A	TIM9	YHR023W	MYO1	0.3269	-0.2196
YIL061C	SNP1	YER022W	SRB4	0.3268	-0.1457
YLL028W	TPO1	YGL208W	SIP2	0.3268	0.1086
YFL047W	RGD2	YEL058W	PCM1	0.3266	0.0837
YLR426W	TDA5	YCL009C	ILV6	0.3266	0.0335
YGR168C	YGR168C	YDL116W	NUP84	0.3265	0.0050
YOL026C	MIM1	YCR031C	RPS14a	0.3265	0.0432
YGL207W	SPT16	YHR023W	MYO1	0.3264	0.0776
YBR077C	SLM4	YEL056W	HAT2	0.3262	0.0087
YLR375W	STP3	YJR074W	MOG1	0.3262	0.1587
YOL073C	DSC2	YOR142W	LSC1	0.3261	0.1967
YHR016C	YSC84	YOR144C	ELG1	0.3259	-0.2002
YDL133W	SRF1	YDR058C	TGL2	0.3258	0.0237
YER116C	SLX8	YDL132W	CDC53	0.3258	0.0504
YLR176C	RFX1	YIL009W	FAA3	0.3258	0.0219
YLR231C	BNA5	YNL322C	KRE1	0.3256	0.0037
YJR056C	YJR056C	YLR455W	PDP3	0.3255	0.0004
YNL321W	VNX1	YKL067W	YNK1	0.3254	-0.0390
YER185W	PUG1	YNL135C	FPR1	0.3252	-0.0267
YML038C	YMD8	YDL133W	SRF1	0.3249	0.0363
YJL183W	MNN11	YJL030W	MAD2	0.3248	0.0554
YBR071W	YBR071W	YEL076W-C	Unknown	0.3243	-0.1383
YJL058C	BIT61	YOR033C	EXO1	0.3242	-0.0424
YKL183W	LOT5	YML091C	RPM2	0.3242	-0.1428
YLL040C	VPS13	YBL014C	RRN6	0.3242	-0.0005
YGL207W	SPT16	YGR152C	RSR1	0.3241	0.0844
YOR080W	DIA2	YOR251C	TUM1	0.3241	0.1769
YER088C	DOT6	YLR368W	MDM30	0.3240	-0.1378
YJL204C	RCY1	YBR163W	EXO5	0.3240	0.0125
YML007W	YAP1	YHR043C	DOG2	0.3238	-0.1278
YDR497C	ITR1	YDR513W	GRX2	0.3237	0.0638
YIR022W	SEC11	YLL024C	SSA2	0.3235	0.0583
YGR152C	RSR1	YBR229C	ROT2	0.3233	-0.0010
YLR059C	REX2	YBL030C	PET9	0.3232	0.1346
YLR452C	SST2	YBL014C	RRN6	0.3229	-0.0489
YOR251C	TUM1	YGL035C	MIG1	0.3229	-0.1350
YIL061C	SNP1	YDR528W	HLR1	0.3228	0.0077
YJR008W	MHO1	YER046W	SPO73	0.3227	0.0878
YLR181C	VTA1	YOL062C	APM4	0.3227	0.1427
YMR215W	GAS3	YMR209C	YMR209C	0.3226	0.2233
YBL079W	NUP170	YKL096W	CWP1	0.3225	-0.1894
YER037W	PHM8	YDR144C	MKC7	0.3225	-0.0899
YGR012W	MCY1	YDR319C	YFT2	0.3225	-0.2171
YLR330W	CHS5	YMR046C	YMR046C	0.3225	0.0038

YNR012W	URK1	YBL014C	RRN6	0.3224	0.1029
YGR006W	PRP18	YGL208W	SIP2	0.3223	0.0522
YHR033W	YHR033W	YIL040W	APQ12	0.3223	0.0505
YHR115C	DMA1	YFL016C	MDJ1	0.3222	0.0428
YOL088C	MPD2	YDR339C	FCF1	0.3222	0.0002
YDR319C	YFT2	YLR176C	RFX1	0.3220	-0.0789
YER182W	FMP10	YIL158W	AIM20	0.3220	0.0405
YBR067C	TIP1	YKL092C	BUD2	0.3218	-0.0928
YDL171C	GLT1	YDL116W	NUP84	0.3217	0.1286
YGL035C	MIG1	YOL113W	SKM1	0.3217	-0.0338
YDL132W	CDC53	YIR022W	SEC11	0.3214	0.0036
YNL074C	MLF3	YDR367W	KEI1	0.3214	-0.0018
YPR022C	SDD4	YDR134C	YDR134C	0.3214	-0.0765
YDL167C	NRP1	YDR420W	HKR1	0.3213	0.2568
YOR371C	GPB1	YML013W	UBX2	0.3213	0.0186
YMR005W	TAF4	YGR168C	YGR168C	0.3209	-0.0625
YBL029W	YBL029W	YJL126W	NIT2	0.3208	0.1058
YER185W	PUG1	YLR059C	REX2	0.3208	-0.1091
YDL171C	GLT1	YJL187C	SWE1	0.3207	-0.1539
YGR194C	XKS1	YML004C	GLO1	0.3207	0.0712
YJR103W	URA8	YFR013W	IOC3	0.3206	-0.1966
YAL009W	SPO7	YGL192W	IME4	0.3203	-0.0630
YMR192W	GYL1	YKL096W	CWP1	0.3202	-0.0262
YHR023W	MYO1	YDR178W	SDH4	0.3201	-0.2056
YOL081W	IRA2	YGR012W	MCY1	0.3201	-0.1768
YBR126C	TPS1	YML004C	GLO1	0.3199	0.1734
YLR231C	BNA5	YJR040W	GEF1	0.3199	0.0101
YOR117W	RPT5	YKL116C	PRR1	0.3199	-0.0534
YOL043C	NTG2	YNL041C	COG6	0.3198	0.0418
YPL009C	RQC2	YER182W	FMP10	0.3198	-0.0782
YFR047C	BNA6	YGR168C	YGR168C	0.3193	-0.1306
YOL164W	BDS1	YPL237W	SUI3	0.3192	-0.0442
YOR054C	VHS3	YJL204C	RCY1	0.3191	-0.2462
YJR086W	STE18	YNL322C	KRE1	0.3189	0.0664
YER037W	PHM8	YDR515W	SLF1	0.3187	-0.0887
YER185W	PUG1	YOR371C	GPB1	0.3186	0.0026
YHR029C	YHI9	YDR530C	APA2	0.3186	0.2060
YMR067C	UBX4	YGL017W	ATE1	0.3185	0.0590
YPL144W	POC4	YDR481C	PHO8	0.3185	-0.0436
YJR040W	GEF1	YOR196C	LIP5	0.3184	-0.0768
YOL062C	APM4	YFL047W	RGD2	0.3184	0.0357
YGL226W	MTC3	YMR067C	UBX4	0.3183	0.0400
YML098W	TAF13	YGL226W	MTC3	0.3183	0.0625
YPL147W	PXA1	YOR142W	LSC1	0.3183	0.0039
YDL133W	SRF1	YGR157W	CHO2	0.3182	0.0362
YPL234C	VMA11	YER023W	PRO3	0.3180	0.0429
YBL020W	RFT1	YGL051W	MST27	0.3179	0.0938

YER019W	ISC1	YER088C	DOT6	0.3177	0.0297
YJL051W	IRC8	YLR046C	YLR046C	0.3175	0.0104
YJL171C	TOH1	YOR088W	YOR088W	0.3173	-0.1109
YER186C	YER186C	YMR046C	YMR046C	0.3172	-0.0164
YIR022W	SEC11	YKL160W	ELF1	0.3171	0.0087
YJL171C	TOH1	YJL126W	NIT2	0.3171	-0.1273
YLL024C	SSA2	YLR443W	ECM7	0.3171	0.0486
YMR062C	ARG7	YJL171C	TOH1	0.3171	0.1695
YOR389W	YOR389W	YAL009W	SPO7	0.3171	-0.0468
YNR034W	SOL1	YBR069C	TAT1	0.3170	-0.0419
YGL017W	ATE1	YGR207C	CIR1	0.3168	-0.0730
YOL062C	APM4	YOR088W	YOR088W	0.3166	-0.0966
YOR033C	EXO1	YDL002C	NHP10	0.3165	0.1422
YCR071C	IMG2	YML098W	TAF13	0.3163	-0.1288
YJL158C	CIS3	YKR080W	MTD1	0.3163	-0.1831
YJL030W	MAD2	YOL088C	MPD2	0.3160	0.0904
YJL154C	VPS35	YER144C	UBP5	0.3159	0.0710
YGR150C	CCM1	YOR103C	OST2	0.3157	-0.0471
YIL111W	COX5b	YJL047C	RTT101	0.3155	0.0913
YJL149W	DAS1	YMR152W	YIM1	0.3153	0.0323
YBR067C	TIP1	YJL163C	YJL163C	0.3151	0.1073
YFR013W	IOC3	YLR206W	ENT2	0.3151	0.0211
YMR067C	UBX4	YDR144C	MKC7	0.3150	0.0424
YFR013W	IOC3	YGL192W	IME4	0.3149	0.0750
YBL040C	ERD2	YOR051C	ETT1	0.3147	-0.0441
YDR178W	SDH4	YGR168C	YGR168C	0.3146	-0.1976
YMR112C	MED11	YMR123W	PKR1	0.3146	0.0460
YDL097C	RPN6	YML098W	TAF13	0.3145	-0.0582
YIL043C	CBR1	YDL111C	RRP42	0.3144	0.2140
YKL110C	KTI12	YGR258C	RAD2	0.3144	-0.0527
YOL113W	SKM1	YKL096W	CWP1	0.3144	-0.0544
YPR015C	YPR015C	YJL178C	ATG27	0.3144	-0.0357
YLL035W	GRC3	YFL018C	LPD1	0.3141	-0.1035
YNL326C	PFA3	YLR455W	PDP3	0.3141	0.0344
YOL092W	YPQ1	YOL097C	WRS1	0.3139	0.1217
YPR196W	YPR196W	YLR057W	MNL2	0.3139	-0.0145
YJL047C	RTT101	YMR209C	YMR209C	0.3138	0.2110
YKL125W	RRN3	YDL167C	NRP1	0.3138	0.2016
YER026C	CHO1	YNR036C	MRPS12	0.3136	0.0226
YDR339C	FCF1	YER022W	SRB4	0.3135	0.1228
YFL049W	SWP82	YLR172C	DPH5	0.3135	-0.0570
YGL226C-A	OST5	YPL237W	SUI3	0.3135	0.0723
YLR426W	TDA5	YLR237W	THI7	0.3135	-0.0529
YBL105C	PKC1	YML036W	CGI121	0.3134	-0.0212
YBL030C	PET9	YGR208W	SER2	0.3134	-0.0748
YFR047C	BNA6	YOL130W	ALR1	0.3134	-0.1415
YGL051W	MST27	YML036W	CGI121	0.3133	-0.0149

YMR112C	MED11	YKL073W	LHS1	0.3131	0.0287
YDL089W	NUR1	YDR441C	APT2	0.3129	-0.0791
YHR029C	YHI9	YMR010W	ANY1	0.3129	-0.1692
YMR123W	PKR1	YMR112C	MED11	0.3128	0.0233
YPL144W	POC4	YGR258C	RAD2	0.3128	-0.0288
YBR222C	PCS60	YJR103W	URA8	0.3127	0.0202
YDR351W	SBE2	YER182W	FMP10	0.3127	-0.0270
YDR383C	NKP1	YOL147C	PEX11	0.3127	-0.0136
YBL020W	RFT1	YJL171C	TOH1	0.3124	0.0001
YJR103W	URA8	YJL149W	DAS1	0.3123	0.1734
YNL326C	PFA3	YLR430W	SEN1	0.3123	-0.0619
YKL184W	SPE1	YLR046C	YLR046C	0.3122	-0.2135
YDR321W	ASP1	YNL280C	ERG24	0.3121	0.1044
YGR152C	RSR1	YDL047W	SIT4	0.3119	0.0903
YMR067C	UBX4	YJL204C	RCY1	0.3119	0.1476
YOR043W	WHI2	YBR196C	PGI1	0.3119	-0.0434
YHR050W	SMF2	YPL058C	PDR12	0.3117	-0.1133
YIL066C	RNR3	YOL081W	IRA2	0.3117	0.0603
YLR455W	PDP3	YGR158C	MTR3	0.3117	0.0718
YPL237W	SUI3	YJR068W	RFC2	0.3117	0.4250
YCL009C	ILV6	YGL208W	SIP2	0.3116	-0.0508
YIL166C	YIL166C	YHR029C	YHI9	0.3113	0.0328
YER144C	UBP5	YIL082W	Unknown	0.3112	-0.0008
YEL076W-C	Unknown	YLR443W	ECM7	0.3111	-0.0367
YCL018W	LEU2	YJR074W	MOG1	0.3109	0.0003
YIL043C	CBR1	YDR144C	MKC7	0.3108	0.1003
YOR127W	RGA1	YFR004W	RPN11	0.3106	0.1821
YGR209C	TRX2	YEL068C	YEL068C	0.3105	0.1189
YER144C	UBP5	YJL149W	DAS1	0.3104	0.0085
YPL226W	NEW1	YLR257W	YLR257W	0.3104	-0.1501
YCL009C	ILV6	YGR288W	MAL13	0.3103	0.1312
YGR150C	CCM1	YJL187C	SWE1	0.3102	0.0239
YGR157W	CHO2	YOL062C	APM4	0.3102	0.0365
YBL007C	SLA1	YBR239C	ERT1	0.3101	0.0003
YJL212C	OPT1	YNL321W	VNX1	0.3100	0.0923
YLR442C	SIR3	YOR033C	EXO1	0.3099	0.0976
YJL118W	YJL118W	YOL088C	MPD2	0.3098	-0.1063
YPR022C	SDD4	YNL329C	PEX6	0.3098	0.1060
YFR037C	RSC8	YHL007C	STE20	0.3097	0.0341
YDR383C	NKP1	YBL020W	RFT1	0.3096	0.0331
YJL126W	NIT2	YBR061C	TRM7	0.3095	0.0371
YOR371C	GPB1	YFR011C	MIC19	0.3095	-0.0600
YHR115C	DMA1	YDR441C	APT2	0.3092	0.0005
YMR154C	RIM13	YBR168W	PEX32	0.3091	0.0519
YDL227C	Ho	YLR443W	ECM7	0.3090	-0.0766
YJL047C	RTT101	YOR088W	YOR088W	0.3090	-0.1673
YLR368W	MDM30	YBR166C	TYR1	0.3090	0.0140

YDL002C	NHP10	YER116C	SLX8	0.3088	0.1401
YJL149W	DAS1	YML013W	UBX2	0.3088	0.0195
YOR054C	VHS3	YGR001C	EFM5	0.3087	-0.0456
YGL025C	PGD1	YBR069C	TAT1	0.3085	-0.0850
YDR420W	HKR1	YBR069C	TAT1	0.3084	-0.1551
YFL016C	MDJ1	YER182W	FMP10	0.3084	0.0273
YNL041C	COG6	YCL024W	KCC4	0.3084	-0.1188
YER022W	SRB4	YBR087W	RFC5	0.3083	0.1008
YBL020W	RFT1	YOL018C	TLG2	0.3082	-0.0278
YER049W	TPA1	YOL092W	YPQ1	0.3080	0.4118
YOL088C	MPD2	YDR180W	SCC2	0.3080	-0.0298
YLL035W	GRC3	YLR287C	YLR287C	0.3078	0.0182
YKR080W	MTD1	YGR222W	PET54	0.3077	-0.1102
YLR368W	MDM30	YOL018C	TLG2	0.3077	0.0798
YPL001W	HAT1	YML098W	TAF13	0.3077	0.0630
YGL195W	GCN1	YGL207W	SPT16	0.3076	0.0598
YGL004C	RPN14	YHR050W	SMF2	0.3074	-0.0804
YGR150C	CCM1	YBR249C	ARO4	0.3074	-0.0107
YNL329C	PEX6	YDR420W	HKR1	0.3074	-0.0632
YKR051W	YKR051W	YOR092W	ECM3	0.3073	0.0904
YNL034W	YNL034W	YBL089W	AVT5	0.3070	0.0271
YPL009C	RQC2	YKL067W	YNK1	0.3070	-0.0157
YDL150W	RPC53	YBL040C	ERD2	0.3069	-0.0661
YOR048C	RAT1	YGR012W	MCY1	0.3069	0.0515
YJL208C	NUC1	YMR158W	MRPS8	0.3068	0.1434
YOR142W	LSC1	YBR222C	PCS60	0.3068	0.0851
YBL030C	PET9	YDR513W	GRX2	0.3067	0.0655
YOR117W	RPT5	YOL093W	TRM10	0.3067	-0.0465
YPL183C	RTT10	YNR012W	URK1	0.3067	0.2666
YNL124W	NAF1	YGR157W	CHO2	0.3066	-0.1111
YOR043W	WHI2	YOL147C	PEX11	0.3066	-0.2263
YML091C	RPM2	YFR013W	IOC3	0.3065	0.0743
YOL092W	YPQ1	YLR430W	SEN1	0.3065	0.1350
YCR083W	TRX3	YDR420W	HKR1	0.3062	0.0904
YMR158W	MRPS8	YMR067C	UBX4	0.3061	0.2671
YCL009C	ILV6	YER116C	SLX8	0.3060	-0.1240
YER019W	ISC1	YMR115W	MGR3	0.3060	-0.0478
YIL015C-A	YIL015C-A	YOR033C	EXO1	0.3059	-0.0233
YGL176C	YGL176C	YKL125W	RRN3	0.3055	0.0709
YHR016C	YSC84	YJL208C	NUC1	0.3055	-0.1941
YDR134C	YDR134C	YEL071W	DLD3	0.3054	-0.0987
YML013W	UBX2	YLR287C	YLR287C	0.3053	-0.0500
YGR209C	TRX2	YJR025C	BNA1	0.3052	-0.1683
YGR222W	PET54	YML098W	TAF13	0.3051	-0.0418
YNL138W	SRV2	YFL018C	LPD1	0.3050	0.0492
YKR080W	MTD1	YDL047W	SIT4	0.3047	-0.0155
YHR023W	MYO1	YLR181C	VTA1	0.3041	0.0363

YNL024C	EFM6	YFL047W	RGD2	0.3041	-0.0013
YGL053W	PRM8	YOR131C	YOR131C	0.3039	-0.0164
YIL171W	YIL171W	YPL144W	POC4	0.3037	-0.1399
YNL326C	PFA3	YLR046C	YLR046C	0.3035	-0.0936
YML004C	GLO1	YJR032W	CPR7	0.3033	-0.2059
YDR528W	HLR1	YDL164C	CDC9	0.3032	0.0831
YPL237W	SUI3	YBR154C	RPB5	0.3032	0.2654
YGL053W	PRM8	YGL226C-A	OST5	0.3031	0.0552
YHR104W	GRE3	YKL067W	YNK1	0.3031	0.1987
YBR154C	RPB5	YBR166C	TYR1	0.3030	0.0616
YHR039C	MSC7	YBR067C	TIP1	0.3030	-0.0909
YJL047C	RTT101	YHR043C	DOG2	0.3030	0.1806
YDR180W	SCC2	YIL142W	CCT2	0.3029	0.0456
YDR034C	LYS14	YIL166C	YIL166C	0.3028	0.0236
YLR046C	YLR046C	YBR137W	YBR137W	0.3024	-0.0556
YDL103C	QRI1	YDL167C	NRP1	0.3022	0.1351
YML110C	COQ5	YGR194C	XKS1	0.3022	0.0762
YGR208W	SER2	YNL074C	MLF3	0.3021	0.0931
YKR051W	YKR051W	YDL217C	TIM22	0.3021	-0.1325
YNR034W	SOL1	YER035W	EDC2	0.3021	0.0047
YBL030C	PET9	YML091C	RPM2	0.3020	0.1391
YGR012W	MCY1	YEL056W	HAT2	0.3018	0.0003
YLR172C	DPH5	YIR026C	YVH1	0.3018	0.1282
YGR158C	MTR3	YGR157W	CHO2	0.3017	0.1631
YPL226W	NEW1	YGL051W	MST27	0.3015	0.2353
YDR528W	HLR1	YIL066C	RNR3	0.3014	0.0278
YGR288W	MAL13	YER185W	PUG1	0.3014	0.1029
YJL051W	IRC8	YLR180W	SAM1	0.3014	-0.0407
YDL097C	RPN6	YMR067C	UBX4	0.3012	0.0838
YDR064W	RPS13	YOR103C	OST2	0.3012	0.0618
YGR149W	GPC1	YDR178W	SDH4	0.3012	0.1413
YIL112W	HOS4	YDR383C	NKP1	0.3012	0.0367
YOR142W	LSC1	YDL155W	CLB3	0.3010	-0.0090
YDL089W	NUR1	YPL058C	PDR12	0.3007	0.1274
YER186C	YER186C	YNL280C	ERG24	0.3007	-0.0585
YGR012W	MCY1	YOR371C	GPB1	0.3007	0.1039
YER019W	ISC1	YDL211C	YDL211C	0.3006	0.0806
YCR031C	RPS14a	YDL150W	RPC53	0.3004	0.1109
YJR086W	STE18	YGR157W	CHO2	0.3003	0.0825
YOL130W	ALR1	YLR059C	REX2	0.3002	0.2514
YDR144C	MKC7	YOR101W	RAS1	0.3001	0.1031
YBR069C	TAT1	YLR176C	RFX1	0.3000	-0.0851
YMR005W	TAF4	YAL051W	OAF1	0.3000	-0.0425
YLR342W	FKS1	YDL002C	NHP10	0.2998	-0.0482
YDR383C	NKP1	YIL112W	HOS4	0.2997	0.0052
YGL257C	MNT2	YLR176C	RFX1	0.2996	-0.0384
YNL041C	COG6	YLR047C	FRE8	0.2996	-0.1714

YPL064C	CWC27	YDR144C	MKC7	0.2996	-0.0790
YDR319C	YFT2	YER186C	YER186C	0.2995	0.1203
YLR426W	TDA5	YJL030W	MAD2	0.2994	0.0919
YML096W	YML096W	YDR383C	NKP1	0.2994	-0.1222
YOL088C	MPD2	YMR209C	YMR209C	0.2993	0.0068
YNL215W	IES2	YHL007C	STE20	0.2991	0.0006
YER020W	GPA2	YGR194C	XKS1	0.2989	0.0541
YJL051W	IRC8	YGL193C	YGL193C	0.2989	0.0942
YGL242C	YGL242C	YOL081W	IRA2	0.2988	-0.0361
YNL022C	RCM1	YJL163C	YJL163C	0.2987	-0.4155
YDL150W	RPC53	YJL171C	TOH1	0.2986	-0.1556
YGR012W	MCY1	YLR257W	YLR257W	0.2986	-0.0877
YBR067C	TIP1	YNL124W	NAF1	0.2985	0.1074
YOL073C	DSC2	YJR040W	GEF1	0.2985	0.0662
YEL076W-C	Unknown	YJL140W	RPB4	0.2979	0.0275
YMR209C	YMR209C	YNR034W	SOL1	0.2979	0.0571
YLR248W	RCK2	YKR075C	YKR075C	0.2978	-0.1999
YJL187C	SWE1	YJL158C	CIS3	0.2977	0.1318
YOR103C	OST2	YEL076W-C	Unknown	0.2976	0.0719
YMR215W	GAS3	YER026C	CHO1	0.2975	0.0676
YNL280C	ERG24	YMR123W	PKR1	0.2975	0.1769
YOL092W	YPQ1	YPL058C	PDR12	0.2975	0.0042
YDR038C	ENA5	YJL051W	IRC8	0.2974	-0.0552
YOL130W	ALR1	YML004C	GLO1	0.2974	-0.2000
YGR211W	ZPR1	YLL035W	GRC3	0.2972	0.2082
YIL171W	YIL171W	YLR052W	IES3	0.2971	-0.1429
YHR054C	YHR054C	YLR176C	RFX1	0.2970	0.0008
YLR287C	YLR287C	YLR052W	IES3	0.2969	0.0856
YMR115W	MGR3	YGL257C	MNT2	0.2967	0.0972
YKR080W	MTD1	YGR208W	SER2	0.2966	0.0842
YOL092W	YPQ1	YJR040W	GEF1	0.2966	0.0462
YJR086W	STE18	YKL073W	LHS1	0.2964	-0.0376
YNR034W	SOL1	YER020W	GPA2	0.2964	0.0212
YGL201C	MCM6	YJR068W	RFC2	0.2963	-0.0638
YBR067C	TIP1	YNL135C	FPR1	0.2961	0.0121
YML036W	CGI121	YLR172C	DPH5	0.2960	0.0723
YDL171C	GLT1	YOR092W	ECM3	0.2959	0.0675
YOL147C	PEX11	YDR383C	NKP1	0.2959	-0.0205
YOR264W	DSE3	YCL024W	KCC4	0.2959	0.2229
YDL103C	QRI1	YDL155W	CLB3	0.2958	0.0601
YKL116C	PRR1	YML098W	TAF13	0.2958	-0.1891
YDL171C	GLT1	YGL226C-A	OST5	0.2957	-0.0502
YGR209C	TRX2	YGR150C	CCM1	0.2957	-0.0627
YDR367W	KEI1	YKL184W	SPE1	0.2955	-0.0009
YOL130W	ALR1	YNL200C	NNR1	0.2955	-0.0451
YFL056C	AAD6	YFR037C	RSC8	0.2954	-0.0632
YGR168C	YGR168C	YDR178W	SDH4	0.2952	-0.0921

YMR154C	RIM13	YDL150W	RPC53	0.2952	-0.1188
YER186C	YER186C	YGR258C	RAD2	0.2951	-0.0863
YBR071W	YBR071W	YKR051W	YKR051W	0.2950	-0.0314
YDR420W	HKR1	YGR006W	PRP18	0.2949	-0.0079
YDR481C	PHO8	YGL226C-A	OST5	0.2949	-0.0703
YDR528W	HLR1	YOL081W	IRA2	0.2949	-0.1515
YIL133C	RPL16a	YNL242W	ATG2	0.2949	-0.4022
YFR013W	IOC3	YGL207W	SPT16	0.2948	0.0132
YMR010W	ANY1	YGR194C	XKS1	0.2947	-0.0401
YPL009C	RQC2	YLR368W	MDM30	0.2947	-0.1267
YBR239C	ERT1	YOR196C	LIP5	0.2946	0.1240
YCL039W	GID7	YML048W	GSF2	0.2946	-0.1001
YML072C	TCB3	YMR209C	YMR209C	0.2945	0.0085
YFL047W	RGD2	YJL163C	YJL163C	0.2944	-0.0806
YML016C	PPZ1	YDR441C	APT2	0.2941	-0.0385
YNL278W	CAF120	YDR531W	CAB1	0.2940	-0.0861
YHL007C	STE20	YFR037C	RSC8	0.2939	0.1542
YIL015C-A	YIL015C-A	YPL071C	YPL071C	0.2939	0.0390
YPL016W	SWI1	YGL226C-A	OST5	0.2939	-0.0813
YJL212C	OPT1	YEL058W	PCM1	0.2938	-0.0787
YNR036C	MRPS12	YFL016C	MDJ1	0.2938	0.1069
YLR330W	CHS5	YEL076W-C	Unknown	0.2937	0.0638
YPL234C	VMA11	YDR515W	SLF1	0.2933	-0.2146
YML013W	UBX2	YLR047C	FRE8	0.2931	0.1148
YBL017C	PEP1	YDR420W	HKR1	0.2930	0.1106
YCR041W	YCR041W	YFR004W	RPN11	0.2930	0.1873
YGL025C	PGD1	YJL051W	IRC8	0.2930	0.0503
YHR029C	YHI9	YKL073W	LHS1	0.2930	-0.0773
YGR001C	EFM5	YPL071C	YPL071C	0.2928	-0.0300
YLR430W	SEN1	YOR048C	RAT1	0.2928	0.0597
YHR209W	CRG1	YOL018C	TLG2	0.2927	0.2049
YER019W	ISC1	YBR069C	TAT1	0.2925	0.1356
YHR039C	MSC7	YDL133W	SRF1	0.2925	-0.0400
YFL016C	MDJ1	YGL193C	YGL193C	0.2924	0.0094
YIL133C	RPL16a	YIL158W	AIM20	0.2923	0.4198
YOR389W	YOR389W	YFR004W	RPN11	0.2923	-0.0601
YGL242C	YGL242C	YGR207C	CIR1	0.2922	0.1191
YGL035C	MIG1	YOR264W	DSE3	0.2921	0.0284
YFL049W	SWP82	YDR441C	APT2	0.2918	-0.0251
YBR061C	TRM7	YNL022C	RCM1	0.2917	0.2370
YCL026C-A	FRM2	YPL183C	RTT10	0.2917	-0.0249
YDR038C	ENA5	YLR176C	RFX1	0.2916	0.0664
YOR251C	TUM1	YLR052W	IES3	0.2916	-0.0676
YEL064C	AVT2	YNL138W	SRV2	0.2915	-0.0074
YPL058C	PDR12	YPL147W	PXA1	0.2914	-0.0323
YDR339C	FCF1	YDL103C	QRI1	0.2913	0.2330
YPR015C	YPR015C	YOR251C	TUM1	0.2913	0.0291

YDR531W	CAB1	YML110C	COQ5	0.2912	-0.0855
YEL021W	URA3	YNL135C	FPR1	0.2912	-0.0404
YNL322C	KRE1	YJL149W	DAS1	0.2912	0.0530
YDR178W	SDH4	YDL155W	CLB3	0.2910	-0.1840
YJL204C	RCY1	YPL064C	CWC27	0.2910	-0.0317
YJL058C	BIT61	YGL169W	SUA5	0.2910	0.0356
YML091C	RPM2	YLR452C	SST2	0.2907	0.0016
YCL026C-A	FRM2	YIL165C	YIL165C	0.2906	0.1086
YMR062C	ARG7	YLR059C	REX2	0.2903	-0.1154
YEL056W	HAT2	YNL329C	PEX6	0.2902	0.2078
YDR383C	NKP1	YHL007C	STE20	0.2900	-0.0717
YHR063C	PAN5	YER049W	TPA1	0.2900	0.0099
YGL226C-A	OST5	YOR092W	ECM3	0.2898	0.0295
YBR222C	PCS60	YLR052W	IES3	0.2897	-0.1799
YOL062C	APM4	YJR040W	GEF1	0.2895	-0.0222
YBL020W	RFT1	YNL329C	PEX6	0.2894	-0.0232
YJR108W	ABM1	YMR115W	MGR3	0.2893	-0.0485
YDL167C	NRP1	YDL111C	RRP42	0.2892	0.1142
YML016C	PPZ1	YOR043W	WHI2	0.2892	0.0035
YKL110C	KTI12	YLR052W	IES3	0.2891	0.0522
YDL180W	YDL180W	YML007W	YAP1	0.2889	0.0616
YHR027C	RPN1	YPL226W	NEW1	0.2888	0.0097
YBR147W	RTC2	YLR052W	IES3	0.2885	-0.1936
YDL155W	CLB3	YOL088C	MPD2	0.2884	-0.1777
YPL031C	PHO85	YGL192W	IME4	0.2884	0.1786
YIL089W	YIL089W	YPL001W	HAT1	0.2883	-0.0006
YFR004W	RPN11	YBL079W	NUP170	0.2881	0.0508
YGL017W	ATE1	YDL217C	TIM22	0.2881	-0.0504
YDL002C	NHP10	YIL133C	RPL16a	0.2880	0.0474
YKL019W	RAM2	YGR023W	MTL1	0.2878	-0.0425
YIL046W	MET30	YNL124W	NAF1	0.2877	0.1482
YOR389W	YOR389W	YOR264W	DSE3	0.2876	-0.0317
YIL166C	YIL166C	YBR222C	PCS60	0.2875	0.0036
YDR481C	PHO8	YPL058C	PDR12	0.2874	0.1293
YOL018C	TLG2	YNL329C	PEX6	0.2874	0.1301
YMR112C	MED11	YGL051W	MST27	0.2873	-0.1198
YBR037C	SCO1	YLL040C	VPS13	0.2871	-0.2488
YGL017W	ATE1	YML048W	GSF2	0.2868	0.0349
YNL046W	YNL046W	YIL040W	APQ12	0.2867	0.0144
YDR339C	FCF1	YOL088C	MPD2	0.2866	0.1910
YEL068C	YEL068C	YMR005W	TAF4	0.2865	0.0655
YGR207C	CIR1	YDL047W	SIT4	0.2864	-0.1537
YOL081W	IRA2	YGL017W	ATE1	0.2864	0.0612
YGL051W	MST27	YIL009W	FAA3	0.2863	-0.0952
YOR051C	ETT1	YER022W	SRB4	0.2863	0.1675
YML007W	YAP1	YBR069C	TAT1	0.2860	0.0556
YBR157C	ICS2	YBR067C	TIP1	0.2859	0.0728

YGL051W	MST27	YKL184W	SPE1	0.2856	0.0155
YBL079W	NUP170	YOR131C	YOR131C	0.2855	-0.1239
YDL116W	NUP84	YDR420W	HKR1	0.2855	0.0375
YJL030W	MAD2	YOR131C	YOR131C	0.2855	0.0201
YBR087W	RFC5	YPL001W	HAT1	0.2854	0.1842
YBR163W	EXO5	YHR115C	DMA1	0.2854	-0.0207
YJR073C	OPI3	YOR264W	DSE3	0.2853	-0.0290
YGR288W	MAL13	YJL118W	YJL118W	0.2852	-0.2450
YKR051W	YKR051W	YJR086W	STE18	0.2852	-0.0386
YJL187C	SWE1	YIL142W	CCT2	0.2851	0.0632
YOR054C	VHS3	YGR258C	RAD2	0.2851	0.0370
YDL116W	NUP84	YIL158W	AIM20	0.2850	0.0488
YDR530C	APA2	YPL012W	RRP12	0.2850	-0.0079
YBR132C	AGP2	YEL064C	AVT2	0.2848	0.0229
YMR005W	TAF4	YMR152W	YIM1	0.2848	0.0891
YKR085C	MRPL20	YLR442C	SIR3	0.2847	0.1693
YLR258W	GSY2	YKR051W	YKR051W	0.2847	0.2988
YOL113W	SKM1	YPR080W	TEF1	0.2847	0.0242
YJR008W	MHO1	YGR149W	GPC1	0.2846	0.0159
YLR442C	SIR3	YFR047C	BNA6	0.2846	-0.0234
YJL154C	VPS35	YEL056W	HAT2	0.2844	0.0425
YBR132C	AGP2	YMR209C	YMR209C	0.2843	0.0717
YCR031C	RPS14a	YKR085C	MRPL20	0.2843	0.1252
YDR034C	LYS14	YOL026C	MIM1	0.2841	-0.1567
YML079W	YML079W	YOL026C	MIM1	0.2841	0.0034
YOR103C	OST2	YIR022W	SEC11	0.2840	0.0682
YGL176C	YGL176C	YBL014C	RRN6	0.2839	0.0842
YLR056W	ERG3	YJL204C	RCY1	0.2839	-0.1997
YOL094C	RFC4	YDL111C	RRP42	0.2839	0.1490
YHR115C	DMA1	YER144C	UBP5	0.2838	0.0596
YKL160W	ELF1	YJR074W	MOG1	0.2838	0.0026
YDR367W	KEI1	YJL047C	RTT101	0.2837	-0.0564
YLR064W	PER33	YJR025C	BNA1	0.2837	-0.0015
YFR013W	IOC3	YER022W	SRB4	0.2834	0.0117
YJL118W	YJL118W	YFR013W	IOC3	0.2834	-0.1059
YEL064C	AVT2	YDL116W	NUP84	0.2833	-0.0385
YNL321W	VNX1	YMR158W	MRPS8	0.2833	0.0336
YER046W	SPO73	YBR168W	PEX32	0.2832	0.0598
YER182W	FMP10	YJL208C	NUC1	0.2832	0.0324
YGR289C	MAL11	YBR239C	ERT1	0.2832	0.2455
YLR176C	RFX1	YML013W	UBX2	0.2830	0.0140
YGL207W	SPT16	YLR430W	SEN1	0.2828	0.1004
YPL009C	RQC2	YLR430W	SEN1	0.2828	0.0213
YBR249C	ARO4	YKL184W	SPE1	0.2824	0.1685
YHR027C	RPN1	YGL226C-A	OST5	0.2822	0.0771
YHR209W	CRG1	YLL040C	VPS13	0.2822	0.0113
YOR080W	DIA2	YDL133W	SRF1	0.2817	0.0051

YNL035C	YNL035C	YDR319C	YFT2	0.2816	-0.0072
YJL126W	NIT2	YLR258W	GSY2	0.2813	-0.0715
YJL047C	RTT101	YPL001W	HAT1	0.2813	0.2472
YER046W	SPO73	YDL171C	GLT1	0.2810	0.0613
YDL002C	NHP10	YKL073W	LHS1	0.2807	-0.0152
YER037W	PHM8	YKL096W	CWP1	0.2806	0.0376
YLR057W	MNL2	YOR092W	ECM3	0.2804	0.2001
YML098W	TAF13	YOL026C	MIM1	0.2804	0.2091
YJL187C	SWE1	YNL329C	PEX6	0.2803	0.0610
YMR123W	PKR1	YLR180W	SAM1	0.2801	0.1447
YGL176C	YGL176C	YFL016C	MDJ1	0.2800	-0.0472
YGR001C	EFM5	YPL144W	POC4	0.2798	0.0786
YJL178C	ATG27	YOR251C	TUM1	0.2797	0.2753
YOR127W	RGA1	YDR134C	YDR134C	0.2797	-0.0127
YLR426W	TDA5	YHR115C	DMA1	0.2796	0.0007
YNL034W	YNL034W	YJL149W	DAS1	0.2795	0.0710
YNL135C	FPR1	YMR209C	YMR209C	0.2794	-0.1608
YJR025C	BNA1	YHR054C	YHR054C	0.2793	0.1940
YFL018C	LPD1	YLL040C	VPS13	0.2791	0.0219
YMR192W	GYL1	YBR161W	CSH1	0.2790	-0.0130
YIL111W	COX5b	YJL212C	OPT1	0.2786	0.0735
YBR166C	TYR1	YPL001W	HAT1	0.2784	0.0639
YER037W	PHM8	YOR048C	RAT1	0.2783	-0.0564
YPR080W	TEF1	YDL211C	YDL211C	0.2783	0.0024
YLR181C	VTA1	YMR067C	UBX4	0.2782	-0.0888
YNR012W	URK1	YBR077C	SLM4	0.2781	-0.0874
YDL047W	SIT4	YGR152C	RSR1	0.2779	0.0629
YDR351W	SBE2	YBR008C	FLR1	0.2779	0.0389
YLR155C	ASP3-1	YJL047C	RTT101	0.2778	0.0756
YDL002C	NHP10	YIL043C	CBR1	0.2777	0.1277
YLR248W	RCK2	YJR097W	JJJ3	0.2777	-0.1979
YOR048C	RAT1	YKL183W	LOT5	0.2776	0.2834
YIL112W	HOS4	YHR050W	SMF2	0.2775	-0.0639
YBR168W	PEX32	YNL074C	MLF3	0.2774	-0.0591
YDL211C	YDL211C	YER186C	YER186C	0.2772	-0.1290
YJR056C	YJR056C	YJL140W	RPB4	0.2771	0.0567
YGL195W	GCN1	YOL147C	PEX11	0.2769	-0.3098
YLR452C	SST2	YDL171C	GLT1	0.2769	0.0704
YDR524C	AGE1	YDR441C	APT2	0.2768	0.0972
YJR097W	JJJ3	YML036W	CGI121	0.2767	0.2021
YJL217W	REE1	YPL001W	HAT1	0.2766	-0.1163
YLR057W	MNL2	YGL035C	MIG1	0.2766	0.0287
YBR193C	MED8	YDL133W	SRF1	0.2765	-0.0667
YLR231C	BNA5	YDR420W	HKR1	0.2764	0.0356
YLR464W	YLR464W	YMR046C	YMR046C	0.2763	0.0403
YOR251C	TUM1	YEL056W	HAT2	0.2763	0.0138
YDR339C	FCF1	YNL022C	RCM1	0.2761	0.0447

YLR435W	TSR2	YOR144C	ELG1	0.2761	0.1696
YEL058W	PCM1	YDR383C	NKP1	0.2760	-0.0520
YHR115C	DMA1	YLR443W	ECM7	0.2759	0.0694
YDL155W	CLB3	YEL058W	PCM1	0.2755	-0.0191
YEL056W	HAT2	YGR006W	PRP18	0.2752	0.0936
YGR289C	MAL11	YDR515W	SLF1	0.2752	0.1030
YHR023W	MYO1	YNR036C	MRPS12	0.2752	-0.0524
YLL028W	TPO1	YBR161W	CSH1	0.2751	0.0345
YLR452C	SST2	YHR023W	MYO1	0.2750	0.0752
YFR011C	MIC19	YDL171C	GLT1	0.2747	-0.1563
YGR258C	RAD2	YHR023W	MYO1	0.2747	0.0204
YCR041W	YCR041W	YBL017C	PEP1	0.2746	-0.0139
YOR131C	YOR131C	YML110C	COQ5	0.2746	-0.0280
YDR134C	YDR134C	YGR012W	MCY1	0.2745	-0.0244
YLR231C	BNA5	YBR274W	CHK1	0.2744	-0.0490
YCL009C	ILV6	YDR319C	YFT2	0.2743	0.1287
YIL089W	YIL089W	YOL026C	MIM1	0.2742	-0.0665
YLL028W	TPO1	YPL144W	POC4	0.2741	0.1499
YGL017W	ATE1	YPL001W	HAT1	0.2739	-0.0813
YEL076W-C	Unknown	YOL147C	PEX11	0.2738	0.0196
YGL025C	PGD1	YER035W	EDC2	0.2736	0.0132
YKL184W	SPE1	YLR059C	REX2	0.2736	-0.1901
YNL034W	YNL034W	YDL150W	RPC53	0.2736	0.0260
YLR064W	PER33	YDR441C	APT2	0.2735	0.1580
YGR211W	ZPR1	YBR147W	RTC2	0.2734	-0.1753
YJL218W	YJL218W	YCL009C	ILV6	0.2733	-0.0120
YCR041W	YCR041W	YLR368W	MDM30	0.2732	-0.0201
YJL030W	MAD2	YOR051C	ETT1	0.2732	0.0896
YHR050W	SMF2	YGL017W	ATE1	0.2731	-0.1784
YBR161W	CSH1	YOR092W	ECM3	0.2730	0.0133
YBR161W	CSH1	YDR367W	KEI1	0.2729	0.1690
YDR058C	TGL2	YOL062C	APM4	0.2728	-0.0739
YER022W	SRB4	YPL001W	HAT1	0.2728	0.0571
YHL007C	STE20	YNL215W	IES2	0.2726	0.2757
YNR036C	MRPS12	YNL135C	FPR1	0.2726	0.1871
YER186C	YER186C	YGL051W	MST27	0.2725	0.0115
YGL193C	YGL193C	YJL047C	RTT101	0.2724	-0.0422
YBR249C	ARO4	YIL165C	YIL165C	0.2723	0.0053
YPL237W	SUI3	YJR074W	MOG1	0.2722	0.3362
YCL024W	KCC4	YOR251C	TUM1	0.2720	-0.0913
YER144C	UBP5	YJL158C	CIS3	0.2720	0.1169
YLR231C	BNA5	YER019W	ISC1	0.2720	-0.0154
YLR443W	ECM7	YJR036C	HUL4	0.2720	0.1420
YIL040W	APQ12	YGR157W	CHO2	0.2718	-0.0298
YJL154C	VPS35	YHR016C	YSC84	0.2717	0.0002
YJR008W	MHO1	YLL061W	MMP1	0.2714	-0.1022
YPR015C	YPR015C	YOL062C	APM4	0.2714	-0.0907

YFR011C	MIC19	YEL071W	DLD3	0.2712	-0.0640
YNL322C	KRE1	YJR074W	MOG1	0.2712	-0.0821
YIL040W	APQ12	YDL047W	SIT4	0.2709	0.1259
YHL048W	COS8	YML048W	GSF2	0.2708	-0.0968
YBL040C	ERD2	YGR150C	CCM1	0.2707	-0.0358
YJL036W	SNX4	YML079W	YML079W	0.2706	0.0206
YCL018W	LEU2	YDL167C	NRP1	0.2701	-0.0069
YDR441C	APT2	YIL009W	FAA3	0.2701	0.0264
YGR158C	MTR3	YER088C	DOT6	0.2701	-0.0027
YJL154C	VPS35	YBR274W	CHK1	0.2701	-0.0376
YNL041C	COG6	YBR196C	PGI1	0.2701	0.0335
YGL051W	MST27	YHL007C	STE20	0.2700	0.0498
YKL092C	BUD2	YJR032W	CPR7	0.2700	0.1348
YBR229C	ROT2	YLR059C	REX2	0.2698	0.0067
YIL171W	YIL171W	YBR069C	TAT1	0.2696	-0.0678
YBL017C	PEP1	YER182W	FMP10	0.2695	-0.1055
YBR229C	ROT2	YNL321W	VNX1	0.2695	0.0596
YBR196C	PGI1	YPL064C	CWC27	0.2694	-0.0001
YPL009C	RQC2	YLR442C	SIR3	0.2694	-0.0114
YJR025C	BNA1	YBR196C	PGI1	0.2692	0.1157
YLL040C	VPS13	YBR037C	SCO1	0.2692	-0.0413
YOR251C	TUM1	YHR063C	PAN5	0.2692	0.0833
YOL018C	TLG2	YGR150C	CCM1	0.2690	0.1214
YJR103W	URA8	YDR367W	KEI1	0.2689	0.1236
YML038C	YMD8	YFL018C	LPD1	0.2689	-0.1068
YML048W	GSF2	YMR115W	MGR3	0.2687	0.1038
YML007W	YAP1	YJL036W	SNX4	0.2687	0.0974
YJL187C	SWE1	YFR004W	RPN11	0.2686	-0.2013
YEL020W-A	TIM9	YLL035W	GRC3	0.2685	-0.1953
YER144C	UBP5	YMR123W	PKR1	0.2684	-0.1304
YGL035C	MIG1	YGR023W	MTL1	0.2682	0.0162
YML013W	UBX2	YML038C	YMD8	0.2681	0.0992
YOR088W	YOR088W	YNR034W	SOL1	0.2680	0.0478
YFL018C	LPD1	YKL125W	RRN3	0.2679	-0.0132
YOL026C	MIM1	YDL211C	YDL211C	0.2679	-0.0949
YBR168W	PEX32	YJL036W	SNX4	0.2678	0.1349
YGL017W	ATE1	YPL031C	PHO85	0.2678	-0.1021
YKL125W	RRN3	YJL218W	YJL218W	0.2678	0.0651
YOR088W	YOR088W	YBR168W	PEX32	0.2678	0.0581
YGL017W	ATE1	YOR033C	EXO1	0.2677	0.0161
YOL088C	MPD2	YKL073W	LHS1	0.2677	0.0148
YDR034C	LYS14	YLR368W	MDM30	0.2676	0.2103
YER116C	SLX8	YDL002C	NHP10	0.2674	0.0070
YIL112W	HOS4	YNL041C	COG6	0.2671	0.1904
YNL074C	MLF3	YBL020W	RFT1	0.2669	-0.1261
YJL030W	MAD2	YGR184C	UBR1	0.2668	-0.0575
YER144C	UBP5	YNL329C	PEX6	0.2666	0.1295

YOL026C	MIM1	YDR420W	HKR1	0.2666	-0.2513
YDL135C	RDI1	YLR057W	MNL2	0.2664	-0.0832
YGR023W	MTL1	YGL017W	ATE1	0.2664	0.0638
YJL118W	YJL118W	YER022W	SRB4	0.2664	-0.0174
YKL184W	SPE1	YLL061W	MMP1	0.2664	0.0254
YDL155W	CLB3	YNR036C	MRPS12	0.2663	-0.0234
YPL144W	POC4	YIL066C	RNR3	0.2663	-0.1265
YLR059C	REX2	YDR530C	APA2	0.2662	-0.0667
YOR043W	WHI2	YJL212C	OPT1	0.2662	0.0330
YNL046W	YNL046W	YOL164W	BDS1	0.2661	0.1835
YLR231C	BNA5	YPL058C	PDR12	0.2660	0.1390
YJL204C	RCY1	YJL118W	YJL118W	0.2659	-0.0364
YJL183W	MNN11	YMR123W	PKR1	0.2659	0.0493
YGL226W	MTC3	YBR196C	PGI1	0.2658	-0.0992
YGR168C	YGR168C	YER116C	SLX8	0.2656	0.0453
YGR209C	TRX2	YOL026C	MIM1	0.2656	0.0643
YOL062C	APM4	YGR001C	EFM5	0.2651	-0.1828
YJL178C	ATG27	YFR013W	IOC3	0.2650	-0.0851
YFR011C	MIC19	YER026C	CHO1	0.2649	0.1627
YIL089W	YIL089W	YGL169W	SUA5	0.2647	-0.0368
YDR441C	APT2	YDL002C	NHP10	0.2646	0.0499
YER046W	SPO73	YEL068C	YEL068C	0.2645	0.0620
YLR180W	SAM1	YLL024C	SSA2	0.2645	0.0944
YOL088C	MPD2	YJL208C	NUC1	0.2645	-0.0137
YFR047C	BNA6	YJL187C	SWE1	0.2644	-0.0859
YJL171C	TOH1	YJL149W	DAS1	0.2641	0.1363
YFR047C	BNA6	YHL048W	COS8	0.2640	0.1801
YJL140W	RPB4	YHR034C	PIH1	0.2640	-0.0153
YOR051C	ETT1	YJL051W	IRC8	0.2640	-0.0373
YER182W	FMP10	YBR222C	PCS60	0.2639	-0.0667
YJL187C	SWE1	YLR426W	TDA5	0.2639	-0.1678
YGL242C	YGL242C	YJL036W	SNX4	0.2638	0.0751
YDR531W	CAB1	YDR481C	PHO8	0.2636	0.1019
YML016C	PPZ1	YJL140W	RPB4	0.2636	-0.0791
YGL226C-A	OST5	YHR054C	YHR054C	0.2635	0.0439
YLR237W	THI7	YJL047C	RTT101	0.2634	-0.0298
YCL009C	ILV6	YBR161W	CSH1	0.2631	-0.1080
YGR194C	XKS1	YER020W	GPA2	0.2630	0.2898
YFR011C	MIC19	YJL030W	MAD2	0.2629	0.1057
YBR069C	TAT1	YJR008W	MHO1	0.2628	-0.0310
YDL171C	GLT1	YER046W	SPO73	0.2628	0.0102
YML016C	PPZ1	YKL092C	BUD2	0.2628	-0.0376
YGR194C	XKS1	YLR258W	GSY2	0.2626	0.0997
YNR034W	SOL1	YLR046C	YLR046C	0.2626	0.0143
YBR077C	SLM4	YAL009W	SPO7	0.2625	0.0773
YDL103C	QRI1	YNL138W	SRV2	0.2625	0.1109
YCR041W	YCR041W	YIL119C	RPI1	0.2624	-0.0470

YMR005W	TAF4	YCR083W	TRX3	0.2624	0.0201
YLL038C	ENT4	YJL036W	SNX4	0.2623	-0.0209
YLL061W	MMP1	YDL002C	NHP10	0.2622	-0.2410
YPL234C	VMA11	YJL051W	IRC8	0.2622	0.1351
YBR222C	PCS60	YLR430W	SEN1	0.2620	0.1323
YLR430W	SEN1	YNL124W	NAF1	0.2618	0.0448
YER116C	SLX8	YOL088C	MPD2	0.2617	0.0788
YEL020W-A	TIM9	YJL149W	DAS1	0.2616	-0.0667
YMR154C	RIM13	YJL158C	CIS3	0.2612	0.0321
YHR209W	CRG1	YFL016C	MDJ1	0.2611	0.1498
YHR209W	CRG1	YLR059C	REX2	0.2610	-0.0013
YNR034W	SOL1	YNL135C	FPR1	0.2610	0.0229
YAL056W	GPB2	YNL321W	VNX1	0.2609	0.0148
YEL020W-A	TIM9	YOL089C	HAL9	0.2609	-0.0508
YKL183W	LOT5	YPL058C	PDR12	0.2609	0.1847
YML048W	GSF2	YDR367W	KEI1	0.2609	0.0005
YDL047W	SIT4	YLR443W	ECM7	0.2608	-0.0473
YPL064C	CWC27	YJL126W	NIT2	0.2608	0.1519
YPL058C	PDR12	YDR481C	PHO8	0.2608	0.0001
YJL171C	TOH1	YKL073W	LHS1	0.2607	0.0048
YLL038C	ENT4	YLR443W	ECM7	0.2607	0.0899
YEL076W-C	Unknown	YMR123W	PKR1	0.2602	-0.0564
YFR013W	IOC3	YPL001W	HAT1	0.2602	0.0064
YJL204C	RCY1	YGR168C	YGR168C	0.2602	0.0005
YIL166C	YIL166C	YGL035C	MIG1	0.2601	-0.0038
YOR142W	LSC1	YER026C	CHO1	0.2601	0.0058
YML121W	GTR1	YGL226C-A	OST5	0.2600	-0.1353
YBR196C	PGI1	YLR257W	YLR257W	0.2599	-0.0649
YGL176C	YGL176C	YPL058C	PDR12	0.2599	-0.0930
YBL014C	RRN6	YER026C	CHO1	0.2598	-0.0875
YDL097C	RPN6	YDL164C	CDC9	0.2598	0.0566
YML098W	TAF13	YML079W	YML079W	0.2598	0.0184
YBL014C	RRN6	YGR157W	CHO2	0.2597	-0.0601
YIL171W	YIL171W	YLL061W	MMP1	0.2597	0.0052
YGL201C	MCM6	YGR001C	EFM5	0.2595	0.0785
YML096W	YML096W	YDL103C	QRI1	0.2595	-0.0983
YNL124W	NAF1	YLR430W	SEN1	0.2595	0.0215
YNL215W	IES2	YBR071W	YBR071W	0.2594	0.0327
YJL154C	VPS35	YNL329C	PEX6	0.2593	0.0001
YJR097W	JJJ3	YGR258C	RAD2	0.2593	0.0568
YJL030W	MAD2	YOR371C	GPB1	0.2592	-0.1060
YML036W	CGI121	YML098W	TAF13	0.2591	0.1334
YIL111W	COX5b	YLR237W	THI7	0.2588	0.1056
YML036W	CGI121	YBL017C	PEP1	0.2588	-0.0010
YOR142W	LSC1	YDR530C	APA2	0.2588	0.1620
YDL103C	QRI1	YOL092W	YPQ1	0.2587	-0.0759
YER046W	SPO73	YGL051W	MST27	0.2587	0.1516

YJL047C	RTT101	YDL164C	CDC9	0.2587	-0.0023
YLR342W	FKS1	YJR025C	BNA1	0.2587	-0.0486
YLR430W	SEN1	YLR096W	KIN2	0.2586	0.1147
YPR015C	YPR015C	YDR319C	YFT2	0.2586	0.0652
YGR184C	UBR1	YLR430W	SEN1	0.2585	0.0353
YBR071W	YBR071W	YJL187C	SWE1	0.2583	0.0451
YOL088C	MPD2	YKL096W	CWP1	0.2583	0.0078
YOR051C	ETT1	YOR048C	RAT1	0.2583	0.0471
YOR103C	OST2	YLR426W	TDA5	0.2582	0.1215
YKL021C	MAK11	YDR180W	SCC2	0.2581	0.1474
YHR039C	MSC7	YBR069C	TAT1	0.2580	0.1549
YGL035C	MIG1	YDL133W	SRF1	0.2578	-0.0159
YJL218W	YJL218W	YHR027C	RPN1	0.2576	0.0226
YER088C	DOT6	YJL204C	RCY1	0.2575	0.0082
YLR368W	MDM30	YER046W	SPO73	0.2574	-0.0021
YBL029W	YBL029W	YBL017C	PEP1	0.2573	0.0012
YOR196C	LIP5	YML098W	TAF13	0.2572	0.0226
YPL071C	YPL071C	YJL149W	DAS1	0.2572	-0.0001
YJR088C	EMC2	YBR161W	CSH1	0.2569	-0.0303
YLR096W	KIN2	YKR051W	YKR051W	0.2568	-0.0307
YOR371C	GPB1	YBL017C	PEP1	0.2568	0.0398
YDR383C	NKP1	YOL093W	TRM10	0.2566	0.0198
YDR321W	ASP1	YML007W	YAP1	0.2564	-0.0828
YLR059C	REX2	YJL208C	NUC1	0.2563	0.0080
YDR180W	SCC2	YDL217C	TIM22	0.2562	0.1331
YKL116C	PRR1	YOR088W	YOR088W	0.2562	-0.2225
YNL329C	PEX6	YOL092W	YPQ1	0.2562	0.0453
YDR319C	YFT2	YHR023W	MYO1	0.2559	-0.0802
YDR524C	AGE1	YLR430W	SEN1	0.2559	-0.1403
YJL218W	YJL218W	YOR196C	LIP5	0.2559	0.0111
YDR321W	ASP1	YGR222W	PET54	0.2558	-0.0353
YOL153C	YOL153C	YER150W	SPI1	0.2558	0.0000
YBR239C	ERT1	YFR047C	BNA6	0.2557	0.0713
YKR051W	YKR051W	YDR319C	YFT2	0.2555	-0.1164
YJR074W	MOG1	YBR168W	PEX32	0.2552	-0.0156
YOR043W	WHI2	YOR196C	LIP5	0.2552	0.0273
YCL026C-A	FRM2	YDR481C	PHO8	0.2551	0.0967
YDR367W	KEI1	YKL183W	LOT5	0.2551	-0.0042
YLR064W	PER33	YJL140W	RPB4	0.2549	0.0479
YFR047C	BNA6	YGL195W	GCN1	0.2548	0.1090
YDR351W	SBE2	YML110C	COQ5	0.2545	-0.0599
YGL226C-A	OST5	YBR157C	ICS2	0.2543	0.0689
YLR455W	PDP3	YER046W	SPO73	0.2543	0.0058
YFL049W	SWP82	YFL047W	RGD2	0.2542	0.0230
YHR209W	CRG1	YPL001W	HAT1	0.2541	0.1702
YIL089W	YIL089W	YBR168W	PEX32	0.2541	0.0606
YOL094C	RFC4	YOL088C	MPD2	0.2541	0.1667

YER088C	DOT6	YLR455W	PDP3	0.2540	0.0964
YBR087W	RFC5	YLR237W	THI7	0.2537	-0.1823
YDL111C	RRP42	YBR137W	YBR137W	0.2537	0.0044
YDL002C	NHP10	YDL167C	NRP1	0.2537	0.0605
YJR073C	OPI3	YGR258C	RAD2	0.2537	-0.1064
YLR443W	ECM7	YIL166C	YIL166C	0.2537	0.1343
YDL089W	NUR1	YJL171C	TOH1	0.2535	-0.0024
YJL208C	NUC1	YFR037C	RSC8	0.2535	0.1219
YNL200C	NNR1	YIL112W	HOS4	0.2535	0.1431
YEL056W	HAT2	YNL321W	VNX1	0.2533	-0.0433
YHR054C	YHR054C	YJL178C	ATG27	0.2533	0.0491
YML091C	RPM2	YCR031C	RPS14a	0.2533	0.0322
YPL144W	POC4	YFR004W	RPN11	0.2533	0.1130
YBR067C	TIP1	YLR430W	SEN1	0.2532	0.0106
YEL056W	HAT2	YER144C	UBP5	0.2532	0.0910
YLR287C	YLR287C	YDR513W	GRX2	0.2532	-0.0667
YJL171C	TOH1	YLR237W	THI7	0.2531	-0.0407
YLR452C	SST2	YBR225W	YBR225W	0.2530	0.0266
YBL020W	RFT1	YMR046C	YMR046C	0.2529	-0.0145
YDL155W	CLB3	YOL147C	PEX11	0.2529	-0.1016
YFL056C	AAD6	YLR443W	ECM7	0.2529	0.0353
YBR137W	YBR137W	YOL130W	ALR1	0.2528	-0.0162
YHR027C	RPN1	YJL036W	SNX4	0.2527	0.0512
YJL217W	REE1	YJL204C	RCY1	0.2524	0.1074
YJL118W	YJL118W	YOR142W	LSC1	0.2524	-0.0419
YOR080W	DIA2	YJL163C	YJL163C	0.2524	0.0912
YNL321W	VNX1	YKR051W	YKR051W	0.2522	0.1287
YOR264W	DSE3	YDR528W	HLR1	0.2520	-0.0231
YBL029W	YBL029W	YBR239C	ERT1	0.2519	0.1473
YGL226W	MTC3	YML007W	YAP1	0.2518	0.0542
YIL158W	AIM20	YDL002C	NHP10	0.2518	0.0278
YCL024W	KCC4	YGR152C	RSR1	0.2516	0.0546
YJL187C	SWE1	YEL068C	YEL068C	0.2515	-0.0886
YFL056C	AAD6	YJL051W	IRC8	0.2513	0.1172
YGR184C	UBR1	YOR043W	WHI2	0.2513	-0.0290
YOR264W	DSE3	YNL329C	PEX6	0.2512	0.0622
YDR513W	GRX2	YGR157W	CHO2	0.2511	-0.1115
YLR375W	STP3	YNL035C	YNL035C	0.2510	-0.0420
YDL167C	NRP1	YER088C	DOT6	0.2507	-0.1005
YJR088C	EMC2	YBL020W	RFT1	0.2507	-0.0479
YKL116C	PRR1	YKR080W	MTD1	0.2506	-0.0497
YGR222W	PET54	YKL067W	YNK1	0.2505	0.0322
YJR074W	MOG1	YGR288W	MAL13	0.2505	-0.0517
YDL227C	Ho	YNR034W	SOL1	0.2502	-0.0062
YGR209C	TRX2	YNL138W	SRV2	0.2502	0.0171
YBL040C	ERD2	YGR001C	EFM5	0.2501	0.0043
YDL047W	SIT4	YFR013W	IOC3	0.2500	0.1675

YDR524C	AGE1	YOL130W	ALR1	0.2499	0.0009
YBL040C	ERD2	YIL009W	FAA3	0.2497	-0.1203
YBR225W	YBR225W	YBR037C	SCO1	0.2497	0.0725
YHR050W	SMF2	YIL112W	HOS4	0.2495	-0.0794
YBR077C	SLM4	YJL036W	SNX4	0.2494	0.0952
YBR274W	CHK1	YGL208W	SIP2	0.2494	-0.0285
YDL002C	NHP10	YLR435W	TSR2	0.2493	0.0866
YLR248W	RCK2	YLL061W	MMP1	0.2493	-0.0454
YKR085C	MRPL20	YAR028W	YAR028W	0.2491	-0.2246
YNR034W	SOL1	YGR157W	CHO2	0.2491	-0.0404
YLR047C	FRE8	YGL192W	IME4	0.2489	0.0561
YML038C	YMD8	YML013W	UBX2	0.2489	0.0384
YDL089W	NUR1	YPL144W	POC4	0.2488	-0.0494
YJL217W	REE1	YOL026C	MIM1	0.2488	-0.0001
YKL096W	CWP1	YBR239C	ERT1	0.2484	-0.1616
YKL067W	YNK1	YLR057W	MNL2	0.2482	-0.0898
YPL144W	POC4	YLR046C	YLR046C	0.2481	0.0413
YLR257W	YLR257W	YHR209W	CRG1	0.2480	0.1287
YOL094C	RFC4	YHR054C	YHR054C	0.2479	0.1676
YPR022C	SDD4	YHR209W	CRG1	0.2478	-0.0463
YLL040C	VPS13	YFL018C	LPD1	0.2477	0.1246
YPL183C	RTT10	YML096W	YML096W	0.2475	0.1389
YBL017C	PEP1	YDR351W	SBE2	0.2474	0.1116
YEL076W-C	Unknown	YOR131C	YOR131C	0.2474	-0.0145
YBL017C	PEP1	YOL081W	IRA2	0.2473	0.1297
YLR449W	FPR4	YNR012W	URK1	0.2473	0.2410
YDR497C	ITR1	YHR054C	YHR054C	0.2472	-0.1012
YLR059C	REX2	YHR063C	PAN5	0.2472	-0.0632
YNL135C	FPR1	YML079W	YML079W	0.2471	0.0155
YBR037C	SCO1	YFL016C	MDJ1	0.2470	0.0128
YKR052C	MRS4	YBR147W	RTC2	0.2466	0.0001
YMR192W	GYL1	YCR083W	TRX3	0.2466	-0.0398
YHR115C	DMA1	YOR092W	ECM3	0.2465	-0.1327
YML004C	GLO1	YOL088C	MPD2	0.2464	0.0897
YBR137W	YBR137W	YOL092W	YPQ1	0.2463	-0.0168
YML048W	GSF2	YER026C	CHO1	0.2463	0.0923
YML098W	TAF13	YNL322C	KRE1	0.2461	-0.0019
YDL135C	RDI1	YGL051W	MST27	0.2460	0.0242
YLR206W	ENT2	YGR149W	GPC1	0.2458	0.0082
YMR158W	MRPS8	YFR037C	RSC8	0.2457	0.0086
YBR071W	YBR071W	YJL204C	RCY1	0.2455	-0.0377
YGL035C	MIG1	YKR075C	YKR075C	0.2451	0.0078
YLR046C	YLR046C	YPL144W	POC4	0.2451	0.0848
YOR117W	RPT5	YER144C	UBP5	0.2450	-0.0135
YER046W	SPO73	YGR258C	RAD2	0.2449	0.1166
YOR142W	LSC1	YDL047W	SIT4	0.2449	-0.0521
YPL001W	HAT1	YJR056C	YJR056C	0.2449	0.0610

YDL164C	CDC9	YMR215W	GAS3	0.2448	0.0874
YGR258C	RAD2	YGL207W	SPT16	0.2447	0.0423
YER026C	CHO1	YPL234C	VMA11	0.2446	0.1726
YNL041C	COG6	YGL208W	SIP2	0.2443	-0.0335
YBR161W	CSH1	YDR530C	APA2	0.2442	-0.0920
YGL207W	SPT16	YLR248W	RCK2	0.2442	0.0626
YEL058W	PCM1	YGL035C	MIG1	0.2436	-0.0136
YDL164C	CDC9	YFR037C	RSC8	0.2435	0.0766
YOR264W	DSE3	YDL047W	SIT4	0.2435	0.1100
YDR420W	HKR1	YJL204C	RCY1	0.2433	0.0974
YJL212C	OPT1	YJR086W	STE18	0.2432	-0.1494
YJR008W	MHO1	YKL183W	LOT5	0.2429	-0.1219
YML013W	UBX2	YER023W	PRO3	0.2428	0.1087
YLR231C	BNA5	YOL088C	MPD2	0.2426	0.0001
YDR531W	CAB1	YGL176C	YGL176C	0.2425	-0.1321
YBR154C	RPB5	YLL040C	VPS13	0.2424	-0.0433
YDR367W	KEI1	YLR047C	FRE8	0.2423	0.0929
YER144C	UBP5	YHL007C	STE20	0.2423	0.1197
YGL025C	PGD1	YKL096W	CWP1	0.2421	-0.0571
YFR047C	BNA6	YLL035W	GRC3	0.2420	-0.1006
YLR257W	YLR257W	YOL113W	SKM1	0.2420	0.0139
YML016C	PPZ1	YDL111C	RRP42	0.2419	0.0007
YGL025C	PGD1	YOR131C	YOR131C	0.2418	0.0554
YIL166C	YIL166C	YBL030C	PET9	0.2416	-0.0445
YOL018C	TLG2	YFR004W	RPN11	0.2416	0.0010
YER182W	FMP10	YLR257W	YLR257W	0.2413	0.0011
YFR011C	MIC19	YGR222W	PET54	0.2413	0.0146
YER049W	TPA1	YJR056C	YJR056C	0.2411	0.2417
YGL017W	ATE1	YFL047W	RGD2	0.2411	0.0040
YHR023W	MYO1	YNL138W	SRV2	0.2410	0.0733
YFR037C	RSC8	YPL064C	CWC27	0.2408	0.1409
YER186C	YER186C	YOR092W	ECM3	0.2407	0.0999
YIL089W	YIL089W	YGR168C	YGR168C	0.2407	0.0850
YLR176C	RFX1	YMR123W	PKR1	0.2406	0.1018
YDL103C	QRI1	YDR339C	FCF1	0.2405	0.0750
YLR443W	ECM7	YOR131C	YOR131C	0.2404	-0.0056
YDR058C	TGL2	YOR196C	LIP5	0.2403	0.1329
YHR027C	RPN1	YDR515W	SLF1	0.2403	0.1549
YHR043C	DOG2	YGR157W	CHO2	0.2399	0.0482
YIL171W	YIL171W	YIL015C-A	YIL015C-A	0.2399	0.1517
YOR142W	LSC1	YMR152W	YIM1	0.2399	0.0010
YCL026C-A	FRM2	YKL096W	CWP1	0.2396	-0.1345
YPL237W	SUI3	YJL178C	ATG27	0.2394	0.2544
YDR441C	APT2	YNL280C	ERG24	0.2392	-0.1107
YLR443W	ECM7	YNL135C	FPR1	0.2392	-0.0862
YER049W	TPA1	YHL007C	STE20	0.2391	0.1077
YHL048W	COS8	YJR074W	MOG1	0.2391	-0.0161

YML098W	TAF13	YJL118W	YJL118W	0.2391	0.0009
YDR528W	HLR1	YMR067C	UBX4	0.2390	0.0161
YKL183W	LOT5	YHR054C	YHR054C	0.2390	0.0561
YLR443W	ECM7	YBR077C	SLM4	0.2389	0.0093
YNL135C	FPR1	YIL043C	CBR1	0.2389	0.0282
YDR524C	AGE1	YDR515W	SLF1	0.2384	0.0110
YML096W	YML096W	YML048W	GSF2	0.2383	0.1683
YNL034W	YNL034W	YDR383C	NKP1	0.2383	0.0073
YER144C	UBP5	YPL058C	PDR12	0.2381	-0.0883
YMR192W	GYL1	YDR497C	ITR1	0.2379	-0.0484
YFR013W	IOC3	YGR209C	TRX2	0.2378	-0.0844
YLR059C	REX2	YNL329C	PEX6	0.2377	0.0923
YGR152C	RSR1	YLR368W	MDM30	0.2376	-0.0828
YML004C	GLO1	YDL111C	RRP42	0.2376	-0.0002
YMR154C	RIM13	YHR063C	PAN5	0.2376	-0.0773
YDL111C	RRP42	YER186C	YER186C	0.2375	0.0620
YDL133W	SRF1	YOR092W	ECM3	0.2372	0.0623
YIL171W	YIL171W	YLR258W	GSY2	0.2372	0.0447
YGL004C	RPN14	YGR288W	MAL13	0.2371	0.0045
YIL089W	YIL089W	YBR069C	TAT1	0.2371	0.0268
YDR034C	LYS14	YIL112W	HOS4	0.2369	0.0365
YPL237W	SUI3	YJR088C	EMC2	0.2369	0.2484
YJL058C	BIT61	YGR289C	MAL11	0.2367	0.0296
YBL014C	RRN6	YLR342W	FKS1	0.2366	-0.0218
YKL110C	KTI12	YDR524C	AGE1	0.2365	0.0352
YER026C	CHO1	YFR011C	MIC19	0.2363	0.0666
YJR032W	CPR7	YMR152W	YIM1	0.2363	-0.0660
YKL021C	MAK11	YDL089W	NUR1	0.2363	-0.1216
YKR052C	MRS4	YMR067C	UBX4	0.2362	-0.0206
YBR229C	ROT2	YOR088W	YOR088W	0.2361	0.1519
YIL133C	RPL16a	YGR211W	ZPR1	0.2361	0.2123
YDL089W	NUR1	YBR239C	ERT1	0.2359	0.1838
YBR008C	FLR1	YJL204C	RCY1	0.2354	-0.0772
YKL019W	RAM2	YDR319C	YFT2	0.2354	0.0472
YOR043W	WHI2	YMR046C	YMR046C	0.2351	-0.0045
YER144C	UBP5	YBL017C	PEP1	0.2350	0.0009
YLR172C	DPH5	YJR073C	OPI3	0.2349	0.0048
YMR062C	ARG7	YDR481C	PHO8	0.2349	0.0898
YBL029W	YBL029W	YGR012W	MCY1	0.2348	0.0101
YJR068W	RFC2	YHR034C	PIH1	0.2348	0.1623
YNL322C	KRE1	YHR027C	RPN1	0.2348	0.0371
YOR051C	ETT1	YOL062C	APM4	0.2348	-0.0437
YBL017C	PEP1	YGR150C	CCM1	0.2347	0.0434
YDL171C	GLT1	YOL088C	MPD2	0.2347	-0.1023
YDR497C	ITR1	YGR184C	UBR1	0.2347	0.1065
YBR067C	TIP1	YJL051W	IRC8	0.2346	-0.0259
YBR161W	CSH1	YMR046C	YMR046C	0.2346	-0.0530

YKR052C	MRS4	YPL001W	HAT1	0.2346	-0.0830
YLL028W	TPO1	YER186C	YER186C	0.2345	0.1570
YDR481C	PHO8	YPL144W	POC4	0.2344	-0.1330
YDR528W	HLR1	YOL088C	MPD2	0.2344	0.0424
YGL178W	MPT5	YER116C	SLX8	0.2343	0.0585
YDR497C	ITR1	YLR047C	FRE8	0.2341	-0.0496
YHR027C	RPN1	YMR158W	MRPS8	0.2341	-0.0255
YLR464W	YLR464W	YJL158C	CIS3	0.2340	-0.0426
YHR115C	DMA1	YHR063C	PAN5	0.2338	-0.0651
YBR071W	YBR071W	YGL226C-A	OST5	0.2337	0.0149
YJL118W	YJL118W	YBR157C	ICS2	0.2334	0.0185
YOR264W	DSE3	YLR258W	GSY2	0.2331	0.0562
YER022W	SRB4	YKL092C	BUD2	0.2330	0.0514
YER023W	PRO3	YKL184W	SPE1	0.2328	0.0367
YJL204C	RCY1	YKL125W	RRN3	0.2327	-0.0909
YNL326C	PFA3	YNL280C	ERG24	0.2325	0.0229
YPL237W	SUI3	YGL226C-A	OST5	0.2325	0.1138
YEL056W	HAT2	YPL071C	YPL071C	0.2324	0.0336
YBR071W	YBR071W	YDR134C	YDR134C	0.2323	-0.0180
YGL192W	IME4	YDR530C	APA2	0.2323	0.0432
YHR023W	MYO1	YGL226C-A	OST5	0.2322	-0.1238
YIR022W	SEC11	YDR134C	YDR134C	0.2322	0.0191
YGR152C	RSR1	YNL321W	VNX1	0.2321	-0.0289
YNR012W	URK1	YAR027W	UIP3	0.2321	-0.2731
YOR251C	TUM1	YBL079W	NUP170	0.2320	-0.1980
YHL007C	STE20	YDR441C	APT2	0.2319	0.0443
YOR033C	EXO1	YDR351W	SBE2	0.2317	0.0864
YGL226C-A	OST5	YJR036C	HUL4	0.2316	-0.1307
YJL212C	OPT1	YBL017C	PEP1	0.2316	-0.0324
YOL062C	APM4	YJR008W	MHO1	0.2316	0.0095
YOR196C	LIP5	YAL051W	OAF1	0.2316	-0.2418
YIL040W	APQ12	YGR006W	PRP18	0.2313	0.0466
YKR085C	MRPL20	YIL066C	RNR3	0.2313	-0.0541
YJR025C	BNA1	YHR029C	YHI9	0.2312	0.0198
YBL017C	PEP1	YLR368W	MDM30	0.2311	0.0027
YDR497C	ITR1	YBR168W	PEX32	0.2311	0.0835
YNL124W	NAF1	YDL150W	RPC53	0.2311	0.0416
YOR088W	YOR088W	YMR209C	YMR209C	0.2310	0.1026
YMR010W	ANY1	YER186C	YER186C	0.2309	0.0305
YAR028W	YAR028W	YAR027W	UIP3	0.2308	0.0141
YML071C	COG8	YLR426W	TDA5	0.2308	-0.1132
YBR157C	ICS2	YNR034W	SOL1	0.2307	-0.0181
YOR389W	YOR389W	YBR163W	EXO5	0.2307	-0.0461
YIL015C-A	YIL015C-A	YMR112C	MED11	0.2306	0.0207
YKL184W	SPE1	YGL208W	SIP2	0.2301	-0.0934
YKL067W	YNK1	YOR371C	GPB1	0.2301	-0.0985
YJL208C	NUC1	YJR032W	CPR7	0.2300	0.0970

YLL040C	VPS13	YGR184C	UBR1	0.2299	0.0293
YOR264W	DSE3	YKL184W	SPE1	0.2297	-0.1308
YGL242C	YGL242C	YMR067C	UBX4	0.2295	-0.1599
YDR064W	RPS13	YHR054C	YHR054C	0.2293	0.0972
YGR023W	MTL1	YJL118W	YJL118W	0.2292	-0.0180
YLR452C	SST2	YPL226W	NEW1	0.2292	0.0021
YCL009C	ILV6	YFR013W	IOC3	0.2290	-0.0187
YDR319C	YFT2	YNL329C	PEX6	0.2288	0.0152
YOL018C	TLG2	YLR257W	YLR257W	0.2288	0.0841
YGL193C	YGL193C	YNL124W	NAF1	0.2287	-0.1023
YJL140W	RPB4	YML038C	YMD8	0.2287	-0.0008
YOL113W	SKM1	YGR012W	MCY1	0.2286	0.0091
YBR157C	ICS2	YML048W	GSF2	0.2285	-0.0014
YJL149W	DAS1	YAL051W	OAF1	0.2285	0.0198
YDL150W	RPC53	YBR077C	SLM4	0.2283	-0.0563
YKL110C	KTI12	YHR050W	SMF2	0.2283	-0.0964
YOR043W	WHI2	YBR071W	YBR071W	0.2283	-0.1411
YMR209C	YMR209C	YKR051W	YKR051W	0.2281	-0.0846
YBR132C	AGP2	YOL026C	MIM1	0.2279	-0.0517
YGL017W	ATE1	YER088C	DOT6	0.2279	0.0238
YJL058C	BIT61	YLR046C	YLR046C	0.2279	-0.0860
YML091C	RPM2	YGR207C	CIR1	0.2278	-0.0472
YGL201C	MCM6	YBR071W	YBR071W	0.2277	0.0790
YLL035W	GRC3	YGR006W	PRP18	0.2275	0.0000
YPL001W	HAT1	YFR004W	RPN11	0.2275	0.1808
YMR123W	PKR1	YBR168W	PEX32	0.2272	-0.0216
YNL322C	KRE1	YKR080W	MTD1	0.2272	-0.0883
YDR528W	HLR1	YNL138W	SRV2	0.2270	0.0889
YIL043C	CBR1	YDR319C	YFT2	0.2269	0.0558
YBR222C	PCS60	YDL116W	NUP84	0.2267	-0.1770
YJL217W	REE1	YKL067W	YNK1	0.2267	0.0460
YJR084W	YJR084W	YLR368W	MDM30	0.2267	-0.0214
YAL051W	OAF1	YJL212C	OPT1	0.2266	-0.0331
YNL074C	MLF3	YBR239C	ERT1	0.2266	-0.2715
YJR008W	MHO1	YOL093W	TRM10	0.2265	-0.0143
YML007W	YAP1	YOR092W	ECM3	0.2265	-0.1521
YER116C	SLX8	YFL016C	MDJ1	0.2263	-0.1534
YEL076W-C	Unknown	YKL116C	PRR1	0.2261	-0.0270
YKL160W	ELF1	YDR134C	YDR134C	0.2261	0.0607
YDR134C	YDR134C	YHR027C	RPN1	0.2260	0.0462
YHR054C	YHR054C	YKL184W	SPE1	0.2259	0.0891
YJR008W	MHO1	YJL187C	SWE1	0.2258	-0.0514
YGR288W	MAL13	YLR449W	FPR4	0.2257	-0.1629
YDL103C	QRI1	YLR046C	YLR046C	0.2255	-0.1130
YDR367W	KEI1	YMR112C	MED11	0.2253	0.0259
YNL329C	PEX6	YLR455W	PDP3	0.2253	-0.0123
YOL092W	YPQ1	YOR371C	GPB1	0.2253	0.1347

YPL147W	PXA1	YOR092W	ECM3	0.2252	-0.0221
YNL280C	ERG24	YNR034W	SOL1	0.2251	0.1963
YKL067W	YNK1	YLR258W	GSY2	0.2250	0.0338
YPR015C	YPR015C	YBR239C	ERT1	0.2249	-0.0530
YJL030W	MAD2	YJL158C	CIS3	0.2247	0.0364
YKR085C	MRPL20	YFL047W	RGD2	0.2247	0.0212
YML098W	TAF13	YNL321W	VNX1	0.2246	-0.1095
YHR029C	YHI9	YER185W	PUG1	0.2245	0.2902
YIL009W	FAA3	YDR180W	SCC2	0.2244	0.0640
YHL048W	COS8	YGL176C	YGL176C	0.2243	-0.0526
YLL040C	VPS13	YLR237W	THI7	0.2243	0.1025
YML091C	RPM2	YPL016W	SWI1	0.2239	0.1111
YOR117W	RPT5	YBL105C	PKC1	0.2239	0.1107
YEL020W-A	TIM9	YER116C	SLX8	0.2238	-0.2085
YLR452C	SST2	YDR420W	HKR1	0.2238	-0.0049
YDL116W	NUP84	YIL009W	FAA3	0.2237	0.1708
YNL041C	COG6	YJL047C	RTT101	0.2237	0.0908
YGL201C	MCM6	YIL112W	HOS4	0.2236	-0.0387
YJL058C	BIT61	YGL226C-A	OST5	0.2235	0.0054
YLR455W	PDP3	YBR225W	YBR225W	0.2234	-0.0071
YGL226W	MTC3	YML121W	GTR1	0.2233	0.0186
YOR033C	EXO1	YOR264W	DSE3	0.2233	0.0377
YCL024W	KCC4	YOL147C	PEX11	0.2232	-0.1446
YIL142W	CCT2	YDR180W	SCC2	0.2232	0.0094
YOR142W	LSC1	YDR134C	YDR134C	0.2232	0.1087
YGL208W	SIP2	YOR051C	ETT1	0.2230	-0.1601
YML121W	GTR1	YIL119C	RPI1	0.2228	0.1127
YIL089W	YIL089W	YJL051W	IRC8	0.2227	-0.0363
YJL217W	REE1	YOR088W	YOR088W	0.2227	-0.1220
YMR123W	PKR1	YHR209W	CRG1	0.2227	-0.0885
YPR015C	YPR015C	YEL071W	DLD3	0.2225	0.0386
YIL061C	SNP1	YML048W	GSF2	0.2224	0.0670
YNL034W	YNL034W	YIL009W	FAA3	0.2222	-0.0553
YDR367W	KEI1	YLL035W	GRC3	0.2221	-0.0412
YJR032W	CPR7	YKL073W	LHS1	0.2221	0.0712
YJR074W	MOG1	YNL280C	ERG24	0.2221	0.0684
YOL073C	DSC2	YCR083W	TRX3	0.2220	0.2930
YIL009W	FAA3	YDL171C	GLT1	0.2218	0.0236
YJR073C	OPI3	YML048W	GSF2	0.2218	0.0522
YMR067C	UBX4	YMR123W	PKR1	0.2218	0.0318
YBR157C	ICS2	YML091C	RPM2	0.2217	-0.0199
YLR452C	SST2	YFR013W	IOC3	0.2216	0.1058
YBR239C	ERT1	YOR048C	RAT1	0.2213	-0.0022
YFR013W	IOC3	YKL067W	YNK1	0.2212	-0.0398
YDR367W	KEI1	YGR006W	PRP18	0.2211	0.0445
YGR157W	CHO2	YJL126W	NIT2	0.2211	-0.0941
YNL329C	PEX6	YOL018C	TLG2	0.2211	0.0353

YBR239C	ERT1	YJR073C	OPI3	0.2210	0.0266
YFL047W	RGD2	YPL064C	CWC27	0.2210	-0.2173
YBR061C	TRM7	YER049W	TPA1	0.2209	0.0689
YJR073C	OPI3	YGR157W	CHO2	0.2209	0.0191
YEL071W	DLD3	YOR127W	RGA1	0.2207	0.2984
YML071C	COG8	YPL009C	RQC2	0.2207	0.1541
YGL051W	MST27	YJL126W	NIT2	0.2205	-0.0994
YOR264W	DSE3	YBR239C	ERT1	0.2205	-0.0375
YML071C	COG8	YJL047C	RTT101	0.2203	0.0095
YNL022C	RCM1	YML096W	YML096W	0.2203	0.1346
YMR067C	UBX4	YML096W	YML096W	0.2202	0.0206
YIR022W	SEC11	YFR047C	BNA6	0.2201	0.0182
YER185W	PUG1	YLR237W	THI7	0.2200	0.0433
YBR126C	TPS1	YDL171C	GLT1	0.2195	-0.1435
YDL133W	SRF1	YJL187C	SWE1	0.2191	0.0394
YLL035W	GRC3	YJR032W	CPR7	0.2191	0.0151
YPL234C	VMA11	YBR121C	GRS1	0.2190	0.1190
YGL017W	ATE1	YMR123W	PKR1	0.2189	0.0406
YDR319C	YFT2	YDL002C	NHP10	0.2188	0.0627
YPL144W	POC4	YGR001C	EFM5	0.2188	0.0712
YDR481C	PHO8	YJL171C	TOH1	0.2185	0.0550
YER186C	YER186C	YDR319C	YFT2	0.2184	0.0754
YFR004W	RPN11	YJR068W	RFC2	0.2183	0.1064
YIL082W	Unknown	YLR047C	FRE8	0.2182	-0.0214
YLR206W	ENT2	YNL135C	FPR1	0.2182	-0.0332
YFL018C	LPD1	YMR067C	UBX4	0.2181	-0.1112
YIR028W	DAL4	YOR101W	RAS1	0.2181	0.0159
YLR375W	STP3	YBR071W	YBR071W	0.2180	0.1778
YDL089W	NUR1	YLR176C	RFX1	0.2178	0.0430
YDR180W	SCC2	YIL009W	FAA3	0.2176	0.1275
YIL171W	YIL171W	YJL212C	OPT1	0.2176	-0.0631
YNR036C	MRPS12	YFR011C	MIC19	0.2175	0.0536
YOR371C	GPB1	YOL093W	TRM10	0.2174	-0.0087
YJR036C	HUL4	YPL012W	RRP12	0.2172	-0.0182
YMR209C	YMR209C	YML036W	CGI121	0.2172	0.0160
YOR103C	OST2	YMR112C	MED11	0.2172	0.0662
YHR039C	MSC7	YPL031C	PHO85	0.2169	0.0015
YJR086W	STE18	YMR112C	MED11	0.2169	0.0675
YJR103W	URA8	YDL167C	NRP1	0.2169	-0.0635
YNL215W	IES2	YNL329C	PEX6	0.2166	0.0069
YEL068C	YEL068C	YOL081W	IRA2	0.2165	-0.0341
YDL135C	RDI1	YDL150W	RPC53	0.2164	0.0559
YGR150C	CCM1	YKR080W	MTD1	0.2164	0.0520
YNL280C	ERG24	YOL147C	PEX11	0.2162	0.1216
YLR257W	YLR257W	YER037W	PHM8	0.2158	0.1193
YFR004W	RPN11	YOR103C	OST2	0.2157	0.0254
YFL049W	SWP82	YIL009W	FAA3	0.2156	0.1193

YOL081W	IRA2	YDL211C	YDL211C	0.2156	-0.0776
YJR088C	EMC2	YMR067C	UBX4	0.2155	-0.0042
YLR096W	KIN2	YFR047C	BNA6	0.2155	-0.0270
YNL321W	VNX1	YGR001C	EFM5	0.2153	-0.1198
YHL048W	COS8	YDL133W	SRF1	0.2152	-0.0888
YKL160W	ELF1	YOR131C	YOR131C	0.2151	0.0615
YIL009W	FAA3	YML036W	CGI121	0.2148	0.0685
YOR054C	VHS3	YBR225W	YBR225W	0.2148	0.0641
YLR064W	PER33	YOL073C	DSC2	0.2144	0.0124
YNL215W	IES2	YFR013W	IOC3	0.2144	-0.1570
YBR067C	TIP1	YBR087W	RFC5	0.2141	-0.0237
YKR085C	MRPL20	YLR443W	ECM7	0.2141	-0.2074
YOR088W	YOR088W	YKL110C	KTI12	0.2140	-0.0306
YOL089C	HAL9	YAR027W	UIP3	0.2138	0.0133
YHR023W	MYO1	YLR452C	SST2	0.2135	0.0026
YDL167C	NRP1	YGR288W	MAL13	0.2132	-0.0373
YGR149W	GPC1	YOL097C	WRS1	0.2130	-0.0244
YLR248W	RCK2	YDL047W	SIT4	0.2130	-0.1749
YER185W	PUG1	YOL147C	PEX11	0.2127	0.1074
YLL035W	GRC3	YDR441C	APT2	0.2127	0.0172
YKL183W	LOT5	YMR209C	YMR209C	0.2125	0.0002
YOL081W	IRA2	YGR023W	MTL1	0.2124	0.1609
YGR012W	MCY1	YOR131C	YOR131C	0.2123	-0.1818
YOL018C	TLG2	YJL047C	RTT101	0.2123	0.0033
YER186C	YER186C	YNL041C	COG6	0.2122	0.0423
YLR172C	DPH5	YHR054C	YHR054C	0.2122	-0.0363
YBR229C	ROT2	YNR034W	SOL1	0.2121	-0.1118
YDR144C	MKC7	YDL116W	NUP84	0.2121	0.0588
YML013W	UBX2	YGL226W	MTC3	0.2121	0.1476
YGR023W	MTL1	YJL163C	YJL163C	0.2119	0.1304
YNR034W	SOL1	YNL280C	ERG24	0.2119	0.0091
YAR027W	UIP3	YNL124W	NAF1	0.2117	-0.0507
YGR150C	CCM1	YER182W	FMP10	0.2115	0.0516
YML091C	RPM2	YLR442C	SIR3	0.2114	-0.1341
YNL215W	IES2	YER035W	EDC2	0.2114	-0.0028
YKR080W	MTD1	YLR057W	MNL2	0.2112	0.0462
YPL009C	RQC2	YFR037C	RSC8	0.2111	0.0373
YDL047W	SIT4	YOR264W	DSE3	0.2110	0.0272
YDL227C	Ho	YGR001C	EFM5	0.2109	0.0827
YDR531W	CAB1	YER023W	PRO3	0.2107	-0.1243
YER046W	SPO73	YEL076W-C	Unknown	0.2107	0.0143
YJL047C	RTT101	YGL051W	MST27	0.2107	-0.1062
YBL017C	PEP1	YBR239C	ERT1	0.2106	0.0843
YER026C	CHO1	YML048W	GSF2	0.2106	0.0881
YKL067W	YNK1	YNL321W	VNX1	0.2105	-0.0845
YMR112C	MED11	YOL164W	BDS1	0.2105	-0.1664
YDR180W	SCC2	YDR351W	SBE2	0.2101	0.2074

YLR046C	YLR046C	YJL149W	DAS1	0.2101	0.1076
YIL009W	FAA3	YER116C	SLX8	0.2100	-0.0881
YOL088C	MPD2	YGR023W	MTL1	0.2100	0.1289
YOL094C	RFC4	YDL217C	TIM22	0.2099	0.0284
YGR207C	CIR1	YGL017W	ATE1	0.2098	-0.0078
YDR144C	MKC7	YIL142W	CCT2	0.2095	0.1269
YJL212C	OPT1	YHR034C	PIH1	0.2093	-0.0007
YLR059C	REX2	YER020W	GPA2	0.2093	0.0455
YIL066C	RNR3	YJL187C	SWE1	0.2091	0.0273
YKL160W	ELF1	YIL009W	FAA3	0.2091	-0.0426
YLR442C	SIR3	YJL051W	IRC8	0.2089	-0.1068
YOL043C	NTG2	YPL144W	POC4	0.2088	-0.0301
YPR022C	SDD4	YGL208W	SIP2	0.2087	0.0000
YNL322C	KRE1	YIL009W	FAA3	0.2086	-0.0231
YDL089W	NUR1	YDL133W	SRF1	0.2084	0.0825
YGL192W	IME4	YNL022C	RCM1	0.2084	-0.0788
YLL040C	VPS13	YML036W	CGI121	0.2081	-0.2198
YJL171C	TOH1	YKR080W	MTD1	0.2080	-0.0065
YKL183W	LOT5	YML110C	COQ5	0.2080	-0.0581
YBR077C	SLM4	YBR087W	RFC5	0.2079	0.0621
YDL211C	YDL211C	YBR008C	FLR1	0.2079	0.0013
YDR528W	HLR1	YOR088W	YOR088W	0.2078	-0.0155
YFL056C	AAD6	YJL171C	TOH1	0.2077	0.0678
YOR043W	WHI2	YML036W	CGI121	0.2077	-0.0936
YOL153C	YOL153C	YDR513W	GRX2	0.2075	0.2804
YBR274W	CHK1	YLR047C	FRE8	0.2074	0.1012
YDL132W	CDC53	YNL322C	KRE1	0.2074	-0.0673
YDL111C	RRP42	YBR225W	YBR225W	0.2074	-0.0308
YJL183W	MNN11	YOR144C	ELG1	0.2074	0.0013
YLR287C	YLR287C	YJR068W	RFC2	0.2073	0.0148
YGR152C	RSR1	YJL187C	SWE1	0.2072	0.0164
YDR420W	HKR1	YPL016W	SWI1	0.2071	0.0131
YGL035C	MIG1	YIL061C	SNP1	0.2070	-0.0081
YNL278W	CAF120	YOL164W	BDS1	0.2070	-0.1421
YFR004W	RPN11	YGR012W	MCY1	0.2069	0.0114
YGR023W	MTL1	YOL088C	MPD2	0.2069	0.1422
YLR176C	RFX1	YLR172C	DPH5	0.2068	-0.0264
YLR248W	RCK2	YGR149W	GPC1	0.2068	0.1118
YBR229C	ROT2	YBR154C	RPB5	0.2067	0.0588
YDR481C	PHO8	YFR037C	RSC8	0.2067	-0.1576
YJR074W	MOG1	YJL158C	CIS3	0.2067	0.0801
YLL061W	MMP1	YIL009W	FAA3	0.2065	0.0001
YJL212C	OPT1	YLL035W	GRC3	0.2063	0.0365
YIL066C	RNR3	YER049W	TPA1	0.2060	-0.0077
YJL212C	OPT1	YJL149W	DAS1	0.2060	-0.0378
YKL110C	KTI12	YGL192W	IME4	0.2060	-0.0568
YAL051W	OAF1	YJL047C	RTT101	0.2059	0.1032

YBR126C	TPS1	YKL110C	KTI12	0.2059	-0.1266
YGL257C	MNT2	YJL212C	OPT1	0.2058	-0.0257
YMR067C	UBX4	YHR023W	MYO1	0.2058	-0.0075
YMR010W	ANY1	YBR069C	TAT1	0.2057	0.1689
YNR012W	URK1	YGR288W	MAL13	0.2057	-0.0531
YLR435W	TSR2	YLR206W	ENT2	0.2056	-0.1118
YBL029W	YBL029W	YLR047C	FRE8	0.2055	-0.0373
YOR251C	TUM1	YBR274W	CHK1	0.2055	-0.0884
YLR368W	MDM30	YLR052W	IES3	0.2054	-0.0259
YOL113W	SKM1	YDR134C	YDR134C	0.2053	-0.1433
YEL056W	HAT2	YJL212C	OPT1	0.2052	-0.1455
YBR193C	MED8	YDR339C	FCF1	0.2051	-0.0103
YHR050W	SMF2	YIL166C	YIL166C	0.2050	-0.1340
YLR237W	THI7	YDR441C	APT2	0.2050	0.0738
YML004C	GLO1	YDL164C	CDC9	0.2050	-0.0265
YOR131C	YOR131C	YER116C	SLX8	0.2050	-0.1210
YIL119C	RPI1	YLR368W	MDM30	0.2048	0.0481
YIL119C	RPI1	YLL035W	GRC3	0.2046	-0.0129
YLR096W	KIN2	YDR441C	APT2	0.2046	-0.0956
YOR371C	GPB1	YER185W	PUG1	0.2045	0.0826
YPL016W	SWI1	YPL001W	HAT1	0.2045	0.0306
YBR037C	SCO1	YCR083W	TRX3	0.2043	0.1612
YBR087W	RFC5	YOL164W	BDS1	0.2043	-0.1488
YKL073W	LHS1	YGR149W	GPC1	0.2043	0.0609
YIL158W	AIM20	YDL116W	NUP84	0.2041	0.0294
YJL183W	MNN11	YDR064W	RPS13	0.2041	0.0073
YFL056C	AAD6	YDL150W	RPC53	0.2039	0.0277
YPL234C	VMA11	YBR161W	CSH1	0.2039	0.1118
YPL031C	PHO85	YMR112C	MED11	0.2039	0.0819
YNL138W	SRV2	YOR371C	GPB1	0.2035	0.0444
YOL093W	TRM10	YNL215W	IES2	0.2035	0.0092
YDR144C	MKC7	YPL064C	CWC27	0.2034	-0.1341
YDR481C	PHO8	YJL158C	CIS3	0.2034	-0.0045
YLR258W	GSY2	YBR071W	YBR071W	0.2033	-0.2462
YBR249C	ARO4	YKL096W	CWP1	0.2032	-0.1226
YBL007C	SLA1	YHR054C	YHR054C	0.2030	0.0901
YKL019W	RAM2	YGR258C	RAD2	0.2030	-0.0364
YFR013W	IOC3	YGL195W	GCN1	0.2029	0.0613
YNR012W	URK1	YHR063C	PAN5	0.2029	0.1214
YIR022W	SEC11	YDR367W	KEI1	0.2028	0.0487
YAL051W	OAF1	YFR037C	RSC8	0.2027	0.0662
YEL058W	PCM1	YLR287C	YLR287C	0.2027	-0.0002
YER022W	SRB4	YNL124W	NAF1	0.2026	-0.0799
YER026C	CHO1	YDR513W	GRX2	0.2026	0.0812
YDR180W	SCC2	YAR027W	UIP3	0.2025	-0.2373
YJL218W	YJL218W	YLR176C	RFX1	0.2022	-0.0441
YGR012W	MCY1	YOR088W	YOR088W	0.2020	0.0221

YDR530C	APA2	YGL169W	SUA5	0.2019	-0.0392
YLR330W	CHS5	YBR008C	FLR1	0.2019	-0.0745
YOL113W	SKM1	YGL035C	MIG1	0.2019	-0.0391
YHL048W	COS8	YLR342W	FKS1	0.2018	0.0517
YPL009C	RQC2	YML071C	COG8	0.2016	0.0067
YJL212C	OPT1	YKR051W	YKR051W	0.2015	0.0316
YGR006W	PRP18	YLR442C	SIR3	0.2014	0.0081
YGL051W	MST27	YJR073C	OPI3	0.2013	0.0049
YDR497C	ITR1	YGR001C	EFM5	0.2012	-0.0578
YER026C	CHO1	YIR022W	SEC11	0.2012	0.0947
YGL035C	MIG1	YGR222W	PET54	0.2011	0.0123
YLR442C	SIR3	YIL009W	FAA3	0.2009	-0.0014
YCL009C	ILV6	YGL176C	YGL176C	0.2007	-0.1017
YDL135C	RDI1	YDL111C	RRP42	0.2006	0.0003
YIL112W	HOS4	YLR047C	FRE8	0.2004	0.0298
YLR046C	YLR046C	YMR123W	PKR1	0.2003	-0.0453
YML098W	TAF13	YDR530C	APA2	0.2002	0.0226
YKR075C	YKR075C	YNL074C	MLF3	0.2001	0.0902
YAL056W	GPB2	YNR036C	MRPS12	0.1998	0.0055
YLR181C	VTA1	YLR375W	STP3	0.1998	-0.0074
YJL178C	ATG27	YIL066C	RNR3	0.1997	-0.0005
YER035W	EDC2	YGL193C	YGL193C	0.1996	0.0405
YMR067C	UBX4	YIL040W	APQ12	0.1996	0.0626
YGL208W	SIP2	YHR016C	YSC84	0.1995	0.0502
YGR023W	MTL1	YDR319C	YFT2	0.1994	-0.0899
YHR063C	PAN5	YOL088C	MPD2	0.1994	-0.0017
YML013W	UBX2	YDL164C	CDC9	0.1994	0.0137
YDR497C	ITR1	YBL007C	SLA1	0.1993	0.0601
YNL138W	SRV2	YOL088C	MPD2	0.1993	-0.0978
YCL009C	ILV6	YDR515W	SLF1	0.1992	-0.1245
YBR163W	EXO5	YOL026C	MIM1	0.1987	-0.1090
YGL035C	MIG1	YLR375W	STP3	0.1984	-0.0007
YIL166C	YIL166C	YDR034C	LYS14	0.1984	0.0076
YBR067C	TIP1	YGL051W	MST27	0.1983	0.0005
YGL193C	YGL193C	YIL009W	FAA3	0.1982	-0.0733
YEL058W	PCM1	YJL163C	YJL163C	0.1981	-0.0547
YIL046W	MET30	YHR063C	PAN5	0.1981	0.0550
YJR025C	BNA1	YDL167C	NRP1	0.1981	-0.0223
YNL200C	NNR1	YOL130W	ALR1	0.1981	-0.2819
YJL217W	REE1	YHR027C	RPN1	0.1980	0.0271
YER046W	SPO73	YNL041C	COG6	0.1979	0.0372
YGL035C	MIG1	YBL030C	PET9	0.1979	0.0035
YKL073W	LHS1	YPL144W	POC4	0.1977	-0.1083
YDR420W	HKR1	YPL144W	POC4	0.1975	-0.0438
YER020W	GPA2	YOL088C	MPD2	0.1975	0.1904
YOL153C	YOL153C	YAL056W	GPB2	0.1975	0.1077
YLR342W	FKS1	YBL014C	RRN6	0.1974	-0.0005

YOL164W	BDS1	YML036W	CGI121	0.1974	-0.0434
YOL018C	TLG2	YOL062C	APM4	0.1973	0.0423
YFR011C	MIC19	YER022W	SRB4	0.1971	0.1083
YER186C	YER186C	YEL056W	HAT2	0.1969	0.0001
YNL200C	NNR1	YGR209C	TRX2	0.1969	0.1454
YGL226C-A	OST5	YOL081W	IRA2	0.1968	-0.0042
YPR015C	YPR015C	YER026C	CHO1	0.1968	0.0001
YML079W	YML079W	YLR056W	ERG3	0.1966	-0.0123
YFL049W	SWP82	YPL058C	PDR12	0.1964	0.0342
YHR034C	PIH1	YJL051W	IRC8	0.1962	0.0795
YBR157C	ICS2	YOR131C	YOR131C	0.1961	-0.0128
YGL226W	MTC3	YGR157W	CHO2	0.1961	-0.0182
YIL112W	HOS4	YLL024C	SSA2	0.1959	-0.0126
YJL051W	IRC8	YOL093W	TRM10	0.1959	-0.0084
YLR057W	MNL2	YOL147C	PEX11	0.1958	0.0603
YBL079W	NUP170	YOR080W	DIA2	0.1956	-0.2069
YGL195W	GCN1	YFR013W	IOC3	0.1956	0.2332
YGL004C	RPN14	YJR025C	BNA1	0.1956	-0.0019
YBR168W	PEX32	YLR258W	GSY2	0.1955	0.0116
YKL160W	ELF1	YJL051W	IRC8	0.1955	0.0824
YOL113W	SKM1	YGR023W	MTL1	0.1955	-0.0754
YOR131C	YOR131C	YBL017C	PEP1	0.1955	-0.0461
YGR023W	MTL1	YKL073W	LHS1	0.1954	-0.0883
YJL171C	TOH1	YDR180W	SCC2	0.1954	-0.0014
YPL031C	PHO85	YJL163C	YJL163C	0.1954	0.1439
YML098W	TAF13	YGR222W	PET54	0.1953	-0.0486
YJL140W	RPB4	YPL234C	VMA11	0.1952	0.0559
YBR037C	SCO1	YML110C	COQ5	0.1951	0.0104
YMR152W	YIM1	YHR054C	YHR054C	0.1949	-0.0378
YPL226W	NEW1	YPL012W	RRP12	0.1949	0.0844
YOL130W	ALR1	YLR442C	SIR3	0.1948	0.0268
YOL093W	TRM10	YLR046C	YLR046C	0.1948	-0.1369
YGL017W	ATE1	YHR029C	YHI9	0.1947	0.0478
YGL176C	YGL176C	YGR023W	MTL1	0.1945	-0.0472
YJL212C	OPT1	YOR088W	YOR088W	0.1945	0.0035
YCL009C	ILV6	YML016C	PPZ1	0.1942	0.1651
YCR031C	RPS14a	YNL022C	RCM1	0.1939	0.0462
YOR196C	LIP5	YDL217C	TIM22	0.1939	0.1815
YML072C	TCB3	YDR319C	YFT2	0.1936	-0.0886
YPL031C	PHO85	YLR057W	MNL2	0.1935	-0.0594
YFL016C	MDJ1	YBR239C	ERT1	0.1934	0.1130
YKR051W	YKR051W	YOL062C	APM4	0.1933	0.0849
YML004C	GLO1	YGR152C	RSR1	0.1933	-0.0984
YDR441C	APT2	YJL047C	RTT101	0.1932	-0.0169
YIL040W	APQ12	YML110C	COQ5	0.1932	-0.0264
YJL217W	REE1	YHR063C	PAN5	0.1932	-0.0012
YDR064W	RPS13	YJL183W	MNN11	0.1931	0.0942

YOR103C	OST2	YMR215W	GAS3	0.1931	0.0335
YKR051W	YKR051W	YLR257W	YLR257W	0.1927	0.0104
YDR134C	YDR134C	YLR426W	TDA5	0.1926	-0.0575
YJR068W	RFC2	YJL183W	MNN11	0.1925	0.1319
YMR123W	PKR1	YPL237W	SUI3	0.1924	0.0039
YOR103C	OST2	YDR367W	KEI1	0.1922	0.0006
YEL056W	HAT2	YER186C	YER186C	0.1921	0.0270
YLR426W	TDA5	YLR047C	FRE8	0.1921	0.0423
YDL097C	RPN6	YIL158W	AIM20	0.1920	0.0308
YNL035C	YNL035C	YDL133W	SRF1	0.1920	-0.0902
YNL329C	PEX6	YPL234C	VMA11	0.1919	-0.0005
YOR043W	WHI2	YDR180W	SCC2	0.1915	-0.0039
YLR180W	SAM1	YOR196C	LIP5	0.1914	-0.0060
YML079W	YML079W	YGL169W	SUA5	0.1913	-0.1330
YJL208C	NUC1	YIL066C	RNR3	0.1912	-0.1548
YBR147W	RTC2	YGR211W	ZPR1	0.1911	-0.1868
YLR258W	GSY2	YKL067W	YNK1	0.1909	0.0595
YLR330W	CHS5	YDR420W	HKR1	0.1907	-0.0632
YLR389C	STE23	YLR046C	YLR046C	0.1906	0.0473
YOL093W	TRM10	YJL051W	IRC8	0.1905	-0.1192
YDL111C	RRP42	YDR144C	MKC7	0.1904	0.0310
YKL110C	KTI12	YJL212C	OPT1	0.1904	0.1687
YIL111W	COX5b	YGR023W	MTL1	0.1902	0.0254
YKL160W	ELF1	YOR051C	ETT1	0.1900	0.1073
YML110C	COQ5	YBR067C	TIP1	0.1900	0.0488
YDR524C	AGE1	YIL158W	AIM20	0.1899	0.0527
YNL022C	RCM1	YDL150W	RPC53	0.1899	0.1866
YDR528W	HLR1	YGR194C	XKS1	0.1897	-0.0639
YNL322C	KRE1	YJL212C	OPT1	0.1897	-0.1367
YPL183C	RTT10	YGL176C	YGL176C	0.1896	0.0021
YGL226W	MTC3	YPL144W	POC4	0.1895	0.0922
YGR157W	CHO2	YOR033C	EXO1	0.1895	0.0620
YKL019W	RAM2	YOL026C	MIM1	0.1894	-0.0533
YGL201C	MCM6	YJR032W	CPR7	0.1893	0.0772
YLR172C	DPH5	YBL014C	RRN6	0.1893	0.0525
YKL092C	BUD2	YML016C	PPZ1	0.1892	-0.0580
YGL169W	SUA5	YER037W	PHM8	0.1891	-0.0111
YJR040W	GEF1	YFR037C	RSC8	0.1889	0.1371
YDL155W	CLB3	YHR027C	RPN1	0.1887	-0.0573
YPR196W	YPR196W	YOL088C	MPD2	0.1887	-0.0532
YLL040C	VPS13	YJL149W	DAS1	0.1886	0.0933
YMR158W	MRPS8	YDL132W	CDC53	0.1886	-0.2263
YBR132C	AGP2	YOL092W	YPQ1	0.1885	0.0571
YIR022W	SEC11	YOR033C	EXO1	0.1883	-0.0177
YJL217W	REE1	YMR112C	MED11	0.1883	-0.0353
YDL002C	NHP10	YDR513W	GRX2	0.1878	0.0414
YOR131C	YOR131C	YBR071W	YBR071W	0.1878	0.0134

YLR180W	SAM1	YJR032W	CPR7	0.1876	-0.0078
YBL020W	RFT1	YOL147C	PEX11	0.1874	-0.1388
YJR084W	YJR084W	YGR152C	RSR1	0.1872	0.0145
YNL329C	PEX6	YOL147C	PEX11	0.1872	0.0953
YBR069C	TAT1	YOR264W	DSE3	0.1871	0.0031
YGL025C	PGD1	YGL176C	YGL176C	0.1870	0.0089
YLR096W	KIN2	YJR086W	STE18	0.1870	-0.0470
YAL051W	OAF1	YMR209C	YMR209C	0.1868	0.0457
YNL124W	NAF1	YML091C	RPM2	0.1866	-0.1108
YNL124W	NAF1	YOR371C	GPB1	0.1866	0.1014
YJL212C	OPT1	YKL110C	KTI12	0.1864	0.0008
YPR196W	YPR196W	YGR157W	CHO2	0.1864	-0.0586
YDL227C	Ho	YNL135C	FPR1	0.1863	0.0004
YLR455W	PDP3	YJR040W	GEF1	0.1863	0.0033
YCL039W	GID7	YLL035W	GRC3	0.1862	-0.0105
YIR022W	SEC11	YJL051W	IRC8	0.1862	-0.1826
YER019W	ISC1	YOL018C	TLG2	0.1861	0.0208
YLR052W	IES3	YBR147W	RTC2	0.1861	-0.0079
YBR239C	ERT1	YDL103C	QRI1	0.1860	-0.0430
YBL089W	AVT5	YHR016C	YSC84	0.1858	-0.0479
YDL111C	RRP42	YOL018C	TLG2	0.1857	-0.0033
YJL187C	SWE1	YIL066C	RNR3	0.1857	0.0452
YGR152C	RSR1	YLR330W	CHS5	0.1856	0.0282
YIL171W	YIL171W	YER116C	SLX8	0.1856	-0.0639
YGR207C	CIR1	YGL208W	SIP2	0.1855	0.0001
YJL154C	VPS35	YDL150W	RPC53	0.1855	-0.0185
YOR048C	RAT1	YBL079W	NUP170	0.1854	0.1437
YIL166C	YIL166C	YDL047W	SIT4	0.1853	-0.0340
YML071C	COG8	YML091C	RPM2	0.1852	0.0982
YBR077C	SLM4	YBR274W	CHK1	0.1851	0.0923
YKL021C	MAK11	YIR026C	YVH1	0.1850	0.1052
YPL147W	PXA1	YDR180W	SCC2	0.1850	0.0063
YPL183C	RTT10	YLR449W	FPR4	0.1849	0.2502
YFR013W	IOC3	YLR375W	STP3	0.1847	-0.0257
YJR103W	URA8	YPL001W	HAT1	0.1847	-0.1085
YOR103C	OST2	YMR046C	YMR046C	0.1845	-0.0360
YDL111C	RRP42	YDL211C	YDL211C	0.1843	-0.0595
YJR056C	YJR056C	YCL009C	ILV6	0.1843	-0.0603
YML007W	YAP1	YBR166C	TYR1	0.1842	0.0024
YOL081W	IRA2	YML038C	YMD8	0.1841	0.0197
YDL150W	RPC53	YBR069C	TAT1	0.1840	0.0623
YER144C	UBP5	YKL116C	PRR1	0.1839	0.0464
YLR342W	FKS1	YGL178W	MPT5	0.1836	0.1061
YDR064W	RPS13	YIL009W	FAA3	0.1834	0.2889
YHR209W	CRG1	YER116C	SLX8	0.1834	0.0399
YKL092C	BUD2	YPL144W	POC4	0.1834	0.0157
YFR047C	BNA6	YDL167C	NRP1	0.1832	-0.1716

YML098W	TAF13	YER186C	YER186C	0.1832	-0.0425
YBL017C	PEP1	YOR131C	YOR131C	0.1830	-0.0047
YGL201C	MCM6	YJL140W	RPB4	0.1829	-0.0035
YBL029W	YBL029W	YER020W	GPA2	0.1827	0.0347
YHR033W	YHR033W	YMR005W	TAF4	0.1826	0.0765
YML072C	TCB3	YGR152C	RSR1	0.1826	-0.0636
YDR034C	LYS14	YDL180W	YDL180W	0.1825	0.1078
YER186C	YER186C	YLR426W	TDA5	0.1823	0.1379
YGL035C	MIG1	YJL178C	ATG27	0.1821	-0.0743
YJL163C	YJL163C	YEL058W	PCM1	0.1820	-0.0957
YMR152W	YIM1	YDL150W	RPC53	0.1819	-0.0744
YOL043C	NTG2	YOR092W	ECM3	0.1818	0.0025
YOR371C	GPB1	YGL192W	IME4	0.1818	0.1093
YBR067C	TIP1	YOR088W	YOR088W	0.1816	-0.0957
YBR193C	MED8	YKL092C	BUD2	0.1816	-0.0187
YBR121C	GRS1	YPL234C	VMA11	0.1814	0.0690
YHR115C	DMA1	YKL184W	SPE1	0.1811	0.0831
YLR248W	RCK2	YLR375W	STP3	0.1810	0.0831
YOR142W	LSC1	YPL144W	POC4	0.1809	0.1160
YER019W	ISC1	YGR149W	GPC1	0.1808	0.0248
YLL040C	VPS13	YGL051W	MST27	0.1808	-0.0064
YBR147W	RTC2	YJL183W	MNN11	0.1807	-0.0264
YJL126W	NIT2	YDR034C	LYS14	0.1805	-0.0501
YOR196C	LIP5	YOL088C	MPD2	0.1805	-0.1967
YDR515W	SLF1	YHR027C	RPN1	0.1804	0.1462
YER026C	CHO1	YIL119C	RPI1	0.1803	0.0801
YFR004W	RPN11	YHR043C	DOG2	0.1803	0.1050
YHR104W	GRE3	YHR016C	YSC84	0.1803	0.1159
YJR068W	RFC2	YGL193C	YGL193C	0.1802	-0.0636
YFR013W	IOC3	YMR209C	YMR209C	0.1799	-0.0552
YEL071W	DLD3	YNR036C	MRPS12	0.1798	-0.0703
YOR117W	RPT5	YFR013W	IOC3	0.1797	0.0437
YJL126W	NIT2	YGR157W	CHO2	0.1795	-0.0430
YML048W	GSF2	YBL020W	RFT1	0.1794	0.0814
YIL061C	SNP1	YPL001W	HAT1	0.1791	-0.0666
YOR196C	LIP5	YDL133W	SRF1	0.1791	0.1082
YDR513W	GRX2	YLR257W	YLR257W	0.1790	0.1350
YLR180W	SAM1	YLR046C	YLR046C	0.1790	-0.0605
YLR452C	SST2	YER023W	PRO3	0.1790	0.0299
YGR006W	PRP18	YOR144C	ELG1	0.1787	0.0035
YJR088C	EMC2	YNL280C	ERG24	0.1786	0.0567
YER035W	EDC2	YOR196C	LIP5	0.1785	0.0405
YPL031C	PHO85	YOR103C	OST2	0.1781	0.0536
YKL092C	BUD2	YJL204C	RCY1	0.1780	0.0952
YOR117W	RPT5	YGR150C	CCM1	0.1773	-0.0271
YJL158C	CIS3	YLR426W	TDA5	0.1772	0.0775
YDL155W	CLB3	YDL150W	RPC53	0.1771	-0.0184

YMR158W	MRPS8	YDR420W	HKR1	0.1771	-0.1024
YNL280C	ERG24	YHR034C	PIH1	0.1771	0.0407
YMR067C	UBX4	YDL167C	NRP1	0.1770	0.0629
YBR121C	GRS1	YIL112W	HOS4	0.1765	-0.1713
YJL171C	TOH1	YLR257W	YLR257W	0.1765	0.0282
YLL028W	TPO1	YNL329C	PEX6	0.1764	0.0439
YLR231C	BNA5	YDR319C	YFT2	0.1763	0.1025
YLR375W	STP3	YGL192W	IME4	0.1762	0.1046
YBR274W	CHK1	YOL026C	MIM1	0.1760	-0.0272
YDL047W	SIT4	YPL009C	RQC2	0.1760	0.0415
YNL046W	YNL046W	YGL195W	GCN1	0.1760	0.0635
YPR080W	TEF1	YNL041C	COG6	0.1760	-0.0130
YLR052W	IES3	YCL024W	KCC4	0.1759	0.0454
YMR010W	ANY1	YPL001W	HAT1	0.1759	0.0913
YLR389C	STE23	YOL026C	MIM1	0.1758	0.0610
YJR056C	YJR056C	YIL009W	FAA3	0.1757	0.1262
YOR371C	GPB1	YEL020W-A	TIM9	0.1757	-0.0378
YJR036C	HUL4	YMR067C	UBX4	0.1756	-0.0412
YNL022C	RCM1	YGR211W	ZPR1	0.1756	0.2357
YMR067C	UBX4	YOR103C	OST2	0.1754	-0.0009
YBR132C	AGP2	YBR239C	ERT1	0.1752	0.0510
YLR248W	RCK2	YDL150W	RPC53	0.1752	0.0119
YBR137W	YBR137W	YDR339C	FCF1	0.1751	-0.1317
YFR042W	KEG1	YFR013W	IOC3	0.1751	-0.0945
YGL178W	MPT5	YPL144W	POC4	0.1749	-0.0259
YGR208W	SER2	YLR237W	THI7	0.1749	-0.0571
YIR026C	YVH1	YGR211W	ZPR1	0.1748	0.0275
YCR071C	IMG2	YGR207C	CIR1	0.1746	0.0617
YKR051W	YKR051W	YNL124W	NAF1	0.1745	-0.0530
YOR043W	WHI2	YDR441C	APT2	0.1745	-0.0501
YPL031C	PHO85	YNL124W	NAF1	0.1745	-0.0120
YNL280C	ERG24	YEL071W	DLD3	0.1744	-0.0623
YJR086W	STE18	YPL144W	POC4	0.1743	0.1637
YFL016C	MDJ1	YDL217C	TIM22	0.1742	0.0361
YJL118W	YJL118W	YIL066C	RNR3	0.1741	0.1025
YBR166C	TYR1	YFR004W	RPN11	0.1739	-0.0137
YAL051W	OAF1	YGL017W	ATE1	0.1738	0.0120
YDR528W	HLR1	YOR092W	ECM3	0.1738	0.0026
YHR043C	DOG2	YBR069C	TAT1	0.1736	0.0610
YLR368W	MDM30	YLR257W	YLR257W	0.1735	0.0307
YGR288W	MAL13	YIL166C	YIL166C	0.1734	0.0663
YLR172C	DPH5	YNL138W	SRV2	0.1734	0.0542
YLR426W	TDA5	YMR123W	PKR1	0.1734	0.0357
YBR225W	YBR225W	YER088C	DOT6	0.1731	0.0026
YIL112W	HOS4	YOL081W	IRA2	0.1729	0.1222
YNL138W	SRV2	YDR441C	APT2	0.1726	-0.0263
YOL104C	NDJ1	YFR013W	IOC3	0.1726	-0.2136

YCL009C	ILV6	YFL018C	LPD1	0.1725	0.0145
YFR004W	RPN11	YFR037C	RSC8	0.1725	0.0259
YPL237W	SUI3	YGR152C	RSR1	0.1724	0.2159
YJL030W	MAD2	YBR163W	EXO5	0.1722	0.0697
YBR121C	GRS1	YBR154C	RPB5	0.1721	0.0159
YIR022W	SEC11	YOR127W	RGA1	0.1721	-0.1003
YDL047W	SIT4	YHR029C	YHI9	0.1720	-0.0036
YIL171W	YIL171W	YDL047W	SIT4	0.1718	-0.0165
YKL092C	BUD2	YMR067C	UBX4	0.1718	-0.0632
YGL025C	PGD1	YOL147C	PEX11	0.1717	-0.0697
YIL043C	CBR1	YCL009C	ILV6	0.1717	0.0022
YBL079W	NUP170	YDR319C	YFT2	0.1716	-0.3030
YEL020W-A	TIM9	YBR168W	PEX32	0.1715	0.0247
YNR036C	MRPS12	YLR443W	ECM7	0.1715	-0.0362
YER049W	TPA1	YBR069C	TAT1	0.1714	0.3063
YFR037C	RSC8	YOR371C	GPB1	0.1714	-0.0386
YML038C	YMD8	YMR215W	GAS3	0.1713	0.0220
YEL020W-A	TIM9	YJR036C	HUL4	0.1712	-0.0805
YHR027C	RPN1	YJL126W	NIT2	0.1709	-0.0207
YGR028W	MSP1	YOL147C	PEX11	0.1708	0.1571
YJL208C	NUC1	YPL031C	PHO85	0.1706	-0.0187
YML079W	YML079W	YBR126C	TPS1	0.1706	-0.0726
YJR073C	OPI3	YLR180W	SAM1	0.1705	0.0413
YNL046W	YNL046W	YHR115C	DMA1	0.1705	0.0190
YGL226C-A	OST5	YHR027C	RPN1	0.1701	0.0752
YLR176C	RFX1	YGL051W	MST27	0.1699	-0.0719
YER116C	SLX8	YMR010W	ANY1	0.1696	-0.0082
YHR039C	MSC7	YLR449W	FPR4	0.1693	0.0323
YMR158W	MRPS8	YDR530C	APA2	0.1693	-0.1738
YOL130W	ALR1	YNL329C	PEX6	0.1691	0.0170
YOL081W	IRA2	YNL041C	COG6	0.1691	0.0042
YPL031C	PHO85	YBR077C	SLM4	0.1691	0.0496
YOR131C	YOR131C	YDR180W	SCC2	0.1689	-0.1067
YFL049W	SWP82	YGL207W	SPT16	0.1688	0.0527
YHR054C	YHR054C	YDR351W	SBE2	0.1687	-0.0272
YML072C	TCB3	YJR086W	STE18	0.1687	0.0660
YER144C	UBP5	YJL187C	SWE1	0.1686	0.0414
YOR371C	GPB1	YJR036C	HUL4	0.1681	0.1125
YBR087W	RFC5	YIL119C	RPI1	0.1680	-0.0001
YDR064W	RPS13	YER186C	YER186C	0.1678	0.1473
YHL007C	STE20	YMR152W	YIM1	0.1678	-0.1063
YMR152W	YIM1	YER049W	TPA1	0.1678	-0.0324
YDR530C	APA2	YLR206W	ENT2	0.1675	0.0487
YDR180W	SCC2	YOL153C	YOL153C	0.1669	-0.0986
YLL035W	GRC3	YJL126W	NIT2	0.1669	0.0801
YOL089C	HAL9	YML013W	UBX2	0.1669	0.0418
YFR013W	IOC3	YML036W	CGI121	0.1666	0.0129

YBR071W	YBR071W	YNL074C	MLF3	0.1665	0.0155
YDR034C	LYS14	YKL067W	YNK1	0.1664	-0.0731
YDR038C	ENA5	YFR037C	RSC8	0.1664	0.0140
YML038C	YMD8	YML110C	COQ5	0.1663	0.1008
YGL193C	YGL193C	YDL002C	NHP10	0.1661	0.0805
YDL133W	SRF1	YPL144W	POC4	0.1659	-0.0818
YLL035W	GRC3	YBL089W	AVT5	0.1658	-0.1017
YOR251C	TUM1	YGR157W	CHO2	0.1658	-0.0082
YGR012W	MCY1	YKL096W	CWP1	0.1657	-0.0204
YOL097C	WRS1	YBR249C	ARO4	0.1651	0.0646
YML091C	RPM2	YOR251C	TUM1	0.1650	-0.0173
YER182W	FMP10	YDL171C	GLT1	0.1648	-0.0011
YFR047C	BNA6	YER186C	YER186C	0.1648	0.0504
YGR149W	GPC1	YOR264W	DSE3	0.1648	0.0462
YGL226W	MTC3	YDR144C	MKC7	0.1647	-0.0280
YMR154C	RIM13	YMR112C	MED11	0.1645	0.0718
YKR080W	MTD1	YKL092C	BUD2	0.1644	-0.0171
YHR033W	YHR033W	YKR051W	YKR051W	0.1643	0.0116
YAR027W	UIP3	YER116C	SLX8	0.1642	0.0328
YBR161W	CSH1	YER185W	PUG1	0.1641	-0.1065
YDR513W	GRX2	YIL009W	FAA3	0.1638	0.1269
YML110C	COQ5	YLR046C	YLR046C	0.1638	-0.0140
YHL007C	STE20	YGL193C	YGL193C	0.1637	-0.0147
YOL026C	MIM1	YKL160W	ELF1	0.1637	0.0062
YEL055C	POL5	YCR031C	RPS14a	0.1636	0.0429
YLR443W	ECM7	YGL176C	YGL176C	0.1636	0.0305
YDR321W	ASP1	YGL208W	SIP2	0.1635	-0.1289
YHR016C	YSC84	YOR080W	DIA2	0.1634	0.1878
YJR068W	RFC2	YOL153C	YOL153C	0.1634	-0.0544
YBR071W	YBR071W	YER186C	YER186C	0.1633	-0.1086
YFL056C	AAD6	YOR051C	ETT1	0.1633	-0.0205
YNL326C	PFA3	YMR067C	UBX4	0.1633	0.0316
YOL018C	TLG2	YOR131C	YOR131C	0.1633	0.0092
YDR531W	CAB1	YDL171C	GLT1	0.1631	0.0822
YGR023W	MTL1	YNR034W	SOL1	0.1631	0.0069
YLR181C	VTA1	YOR103C	OST2	0.1631	0.0400
YPL071C	YPL071C	YJR074W	MOG1	0.1630	-0.0221
YMR067C	UBX4	YGL226W	MTC3	0.1628	0.0069
YKL184W	SPE1	YJL158C	CIS3	0.1626	-0.1433
YOL147C	PEX11	YGL051W	MST27	0.1625	-0.0234
YBR161W	CSH1	YPL234C	VMA11	0.1623	0.0023
YBR229C	ROT2	YER144C	UBP5	0.1623	-0.2510
YHR063C	PAN5	YJR025C	BNA1	0.1623	0.0464
YDL135C	RDI1	YER035W	EDC2	0.1622	-0.0033
YPL144W	POC4	YBR274W	CHK1	0.1622	-0.0886
YKL110C	KTI12	YKR051W	YKR051W	0.1621	-0.0015
YDL116W	NUP84	YDR441C	APT2	0.1620	0.0874

YNL280C	ERG24	YER182W	FMP10	0.1620	-0.0615
YDR351W	SBE2	YIL046W	MET30	0.1619	0.0837
YML048W	GSF2	YHR016C	YSC84	0.1619	-0.0593
YGR289C	MAL11	YCL024W	KCC4	0.1617	-0.0418
YIL112W	HOS4	YPL183C	RTT10	0.1615	-0.0016
YDL002C	NHP10	YGR012W	MCY1	0.1613	-0.0041
YDR524C	AGE1	YNR034W	SOL1	0.1613	0.0776
YEL068C	YEL068C	YBR087W	RFC5	0.1612	-0.0078
YJL171C	TOH1	YML013W	UBX2	0.1610	-0.0452
YDL150W	RPC53	YBR071W	YBR071W	0.1609	0.0395
YEL071W	DLD3	YKL096W	CWP1	0.1609	-0.0795
YHR054C	YHR054C	YJL187C	SWE1	0.1607	-0.0973
YGL193C	YGL193C	YKL073W	LHS1	0.1603	-0.0134
YEL058W	PCM1	YGR001C	EFM5	0.1601	0.0621
YNL022C	RCM1	YOR131C	YOR131C	0.1601	0.0067
YDL135C	RDI1	YDR178W	SDH4	0.1600	0.0923
YGR258C	RAD2	YNL280C	ERG24	0.1600	0.0164
YGR158C	MTR3	YBR249C	ARO4	0.1597	0.0199
YIL066C	RNR3	YEL056W	HAT2	0.1596	-0.0003
YJL183W	MNN11	YKR080W	MTD1	0.1594	-0.0383
YJL036W	SNX4	YOR088W	YOR088W	0.1591	0.0902
YPL016W	SWI1	YAL009W	SPO7	0.1591	-0.0398
YHR115C	DMA1	YHR209W	CRG1	0.1588	0.0028
YOL026C	MIM1	YLR176C	RFX1	0.1588	-0.1006
YLR057W	MNL2	YFL047W	RGD2	0.1587	0.0940
YMR067C	UBX4	YLR046C	YLR046C	0.1587	-0.0074
YDR064W	RPS13	YNL242W	ATG2	0.1583	-0.0665
YLR172C	DPH5	YOR131C	YOR131C	0.1583	0.1256
YDR321W	ASP1	YPL071C	YPL071C	0.1581	-0.0119
YER182W	FMP10	YER144C	UBP5	0.1580	0.0036
YGR157W	CHO2	YMR067C	UBX4	0.1580	-0.0129
YPL012W	RRP12	YOR048C	RAT1	0.1579	0.2620
YOR054C	VHS3	YKL116C	PRR1	0.1578	-0.0090
YPL009C	RQC2	YOR092W	ECM3	0.1577	0.1080
YBL014C	RRN6	YJL140W	RPB4	0.1575	0.0006
YJR084W	YJR084W	YBR239C	ERT1	0.1574	0.0648
YPL009C	RQC2	YDR180W	SCC2	0.1573	0.0738
YGR012W	MCY1	YLR287C	YLR287C	0.1571	-0.0346
YOL093W	TRM10	YLR455W	PDP3	0.1570	-0.1506
YOR127W	RGA1	YGR001C	EFM5	0.1569	0.0512
YJL217W	REE1	YER144C	UBP5	0.1568	0.0423
YGL176C	YGL176C	YKR051W	YKR051W	0.1564	0.0597
YGL242C	YGL242C	YGR150C	CCM1	0.1561	-0.0577
YPL144W	POC4	YJR086W	STE18	0.1559	0.0670
YMR215W	GAS3	YEL055C	POL5	0.1556	0.0389
YJL126W	NIT2	YBR147W	RTC2	0.1554	-0.0020
YDL111C	RRP42	YMR215W	GAS3	0.1553	-0.0732

YDL132W	CDC53	YER186C	YER186C	0.1551	-0.0448
YJL036W	SNX4	YGR012W	MCY1	0.1551	0.0088
YIL040W	APQ12	YER186C	YER186C	0.1550	0.0769
YKL019W	RAM2	YGR001C	EFM5	0.1550	0.1207
YGL201C	MCM6	YKL092C	BUD2	0.1549	-0.0560
YLR430W	SEN1	YBR069C	TAT1	0.1541	-0.0862
YML004C	GLO1	YIL009W	FAA3	0.1541	-0.1489
YLR426W	TDA5	YBR222C	PCS60	0.1539	0.0498
YMR192W	GYL1	YDR319C	YFT2	0.1539	0.0945
YDR497C	ITR1	YNR034W	SOL1	0.1538	-0.0209
YIL171W	YIL171W	YDR319C	YFT2	0.1538	-0.1010
YPR022C	SDD4	YFR013W	IOC3	0.1538	0.0232
YJL036W	SNX4	YJL187C	SWE1	0.1537	-0.0214
YDR420W	HKR1	YML098W	TAF13	0.1535	-0.0207
YJL212C	OPT1	YLR206W	ENT2	0.1535	-0.0279
YBL089W	AVT5	YNL329C	PEX6	0.1534	-0.0352
YGR258C	RAD2	YOL092W	YPQ1	0.1532	-0.1072
YIL166C	YIL166C	YGR158C	MTR3	0.1531	-0.0646
YBR161W	CSH1	YML048W	GSF2	0.1530	0.0552
YIL165C	YIL165C	YJL051W	IRC8	0.1530	-0.0851
YHR039C	MSC7	YDR420W	HKR1	0.1529	0.0475
YCL039W	GID7	YNL124W	NAF1	0.1527	0.0203
YCL026C-A	FRM2	YKL125W	RRN3	0.1527	0.0807
YIL040W	APQ12	YOL026C	MIM1	0.1527	-0.0924
YPL144W	POC4	YOL026C	MIM1	0.1526	0.0000
YGR208W	SER2	YBR137W	YBR137W	0.1524	0.0211
YPL009C	RQC2	YNL041C	COG6	0.1524	0.1053
YBR229C	ROT2	YOL088C	MPD2	0.1523	0.0801
YML072C	TCB3	YGR001C	EFM5	0.1523	-0.0134
YER019W	ISC1	YER026C	CHO1	0.1521	-0.0587
YML007W	YAP1	YDR351W	SBE2	0.1519	0.0003
YIL133C	RPL16a	YBL029W	YBL029W	0.1517	-0.1542
YLR047C	FRE8	YDR180W	SCC2	0.1516	0.0306
YCR041W	YCR041W	YOR371C	GPB1	0.1515	-0.0593
YER144C	UBP5	YLR257W	YLR257W	0.1515	0.0369
YGR207C	CIR1	YDR180W	SCC2	0.1512	-0.0360
YJL036W	SNX4	YHL007C	STE20	0.1512	0.0425
YOL092W	YPQ1	YPL009C	RQC2	0.1509	0.0428
YGL201C	MCM6	YDR319C	YFT2	0.1508	-0.0450
YHR029C	YHI9	YNL124W	NAF1	0.1508	0.1007
YEL055C	POL5	YER088C	DOT6	0.1505	-0.2520
YIL015C-A	YIL015C-A	YLR375W	STP3	0.1505	-0.0112
YDR530C	APA2	YIL112W	HOS4	0.1503	0.0125
YNL035C	YNL035C	YBR071W	YBR071W	0.1501	-0.0417
YLL040C	VPS13	YOR131C	YOR131C	0.1500	-0.0006
YDR319C	YFT2	YKL092C	BUD2	0.1499	-0.1500
YIL133C	RPL16a	YML013W	UBX2	0.1498	-0.2241

YBL014C	RRN6	YBR008C	FLR1	0.1497	0.0004
YBR157C	ICS2	YGR001C	EFM5	0.1496	-0.1473
YDR481C	PHO8	YOL147C	PEX11	0.1496	0.1137
YPL009C	RQC2	YFR013W	IOC3	0.1496	0.0196
YBR077C	SLM4	YDL150W	RPC53	0.1495	-0.0274
YGR028W	MSP1	YOL062C	APM4	0.1494	0.1037
YKL116C	PRR1	YDR441C	APT2	0.1493	-0.0515
YMR152W	YIM1	YNL242W	ATG2	0.1493	0.0475
YER022W	SRB4	YDL217C	TIM22	0.1492	-0.0186
YKL019W	RAM2	YKL073W	LHS1	0.1492	0.0193
YMR010W	ANY1	YOL062C	APM4	0.1490	0.0603
YMR115W	MGR3	YER116C	SLX8	0.1489	-0.0121
YNL074C	MLF3	YKL067W	YNK1	0.1489	-0.0529
YPL058C	PDR12	YJL149W	DAS1	0.1488	-0.0671
YDR524C	AGE1	YJL187C	SWE1	0.1481	0.0771
YIR022W	SEC11	YBR163W	EXO5	0.1480	-0.0505
YOL062C	APM4	YLR046C	YLR046C	0.1480	0.0167
YOR101W	RAS1	YMR192W	GYL1	0.1480	-0.0137
YDR531W	CAB1	YDR530C	APA2	0.1479	0.1393
YKL125W	RRN3	YNL022C	RCM1	0.1478	0.0008
YDR383C	NKP1	YNL138W	SRV2	0.1477	-0.1142
YJL158C	CIS3	YPL071C	YPL071C	0.1472	-0.0801
YJR097W	JJJ3	YBR087W	RFC5	0.1472	0.0184
YJL051W	IRC8	YIL009W	FAA3	0.1471	0.0739
YJR008W	MHO1	YJL178C	ATG27	0.1470	0.0750
YDL111C	RRP42	YKL092C	BUD2	0.1469	-0.0665
YML038C	YMD8	YER144C	UBP5	0.1469	0.0137
YER049W	TPA1	YOR048C	RAT1	0.1461	0.0075
YNL326C	PFA3	YKL092C	BUD2	0.1460	-0.0342
YHR043C	DOG2	YJR086W	STE18	0.1459	-0.0233
YBR249C	ARO4	YNR034W	SOL1	0.1457	0.1935
YLR442C	SIR3	YMR112C	MED11	0.1456	0.1441
YDR513W	GRX2	YLR206W	ENT2	0.1452	0.0939
YMR112C	MED11	YKL067W	YNK1	0.1452	-0.0525
YMR209C	YMR209C	YPL001W	HAT1	0.1450	-0.0333
YLR258W	GSY2	YDR134C	YDR134C	0.1449	0.2009
YNL046W	YNL046W	YFR013W	IOC3	0.1445	-0.1473
YKL019W	RAM2	YJR074W	MOG1	0.1442	0.0026
YPR080W	TEF1	YOR088W	YOR088W	0.1441	-0.0199
YCL026C-A	FRM2	YIR026C	YVH1	0.1440	0.0190
YJL204C	RCY1	YBR071W	YBR071W	0.1437	-0.0864
YGL017W	ATE1	YBL030C	PET9	0.1434	-0.0173
YIL043C	CBR1	YJL212C	OPT1	0.1431	0.0719
YOL097C	WRS1	YER023W	PRO3	0.1430	0.2436
YLR287C	YLR287C	YFL016C	MDJ1	0.1428	-0.0581
YGL226W	MTC3	YCR083W	TRX3	0.1427	0.1354
YCR083W	TRX3	YER144C	UBP5	0.1424	0.0761

YHL007C	STE20	YDR319C	YFT2	0.1417	-0.0754
YPR080W	TEF1	YDR319C	YFT2	0.1416	-0.0151
YGL193C	YGL193C	YPL058C	PDR12	0.1415	0.0173
YDL135C	RDI1	YKL067W	YNK1	0.1414	0.0082
YLL024C	SSA2	YMR067C	UBX4	0.1413	-0.0120
YGL051W	MST27	YOL088C	MPD2	0.1412	0.0577
YKR085C	MRPL20	YDL150W	RPC53	0.1412	0.0537
YML079W	YML079W	YNL124W	NAF1	0.1411	-0.1217
YDR383C	NKP1	YKL073W	LHS1	0.1409	-0.0871
YJR068W	RFC2	YDR528W	HLR1	0.1409	0.0125
YBR222C	PCS60	YHR063C	PAN5	0.1406	0.0783
YCR071C	IMG2	YGL226C-A	OST5	0.1406	0.0474
YIL040W	APQ12	YLR455W	PDP3	0.1406	0.0306
YKL096W	CWP1	YER144C	UBP5	0.1406	0.0379
YBR087W	RFC5	YDL103C	QRI1	0.1400	0.0055
YER020W	GPA2	YBL030C	PET9	0.1400	0.0244
YLR452C	SST2	YGL176C	YGL176C	0.1397	-0.1208
YJL178C	ATG27	YNL138W	SRV2	0.1396	0.0703
YIL171W	YIL171W	YML036W	CGI121	0.1394	-0.0906
YLR046C	YLR046C	YDR319C	YFT2	0.1394	0.0394
YNL280C	ERG24	YOR088W	YOR088W	0.1394	0.1783
YMR215W	GAS3	YKL073W	LHS1	0.1392	0.1642
YCR031C	RPS14a	YOR088W	YOR088W	0.1391	-0.1354
YMR154C	RIM13	YDL211C	YDL211C	0.1391	-0.0048
YGL017W	ATE1	YLR257W	YLR257W	0.1390	-0.0162
YLR248W	RCK2	YBL007C	SLA1	0.1384	0.0001
YPL012W	RRP12	YGR209C	TRX2	0.1382	-0.1780
YJR074W	MOG1	YER026C	CHO1	0.1379	-0.0022
YPL071C	YPL071C	YIL166C	YIL166C	0.1379	-0.0031
YPL064C	CWC27	YEL020W-A	TIM9	0.1379	0.0791
YBR157C	ICS2	YML036W	CGI121	0.1378	0.0806
YOR251C	TUM1	YML110C	COQ5	0.1377	0.0225
YBR121C	GRS1	YLR375W	STP3	0.1373	-0.1308
YPL031C	PHO85	YOL147C	PEX11	0.1373	-0.0356
YBL040C	ERD2	YLL035W	GRC3	0.1372	-0.0284
YBR168W	PEX32	YJL158C	CIS3	0.1371	0.0523
YLR059C	REX2	YBR061C	TRM7	0.1368	0.0481
YJL118W	YJL118W	YLR057W	MNL2	0.1367	-0.0107
YAL051W	OAF1	YER088C	DOT6	0.1365	0.0296
YDR064W	RPS13	YGL226C-A	OST5	0.1365	0.0532
YER185W	PUG1	YPL144W	POC4	0.1363	-0.1705
YAL051W	OAF1	YGL051W	MST27	0.1362	-0.0251
YBR161W	CSH1	YJR025C	BNA1	0.1360	-0.0001
YCL024W	KCC4	YLR047C	FRE8	0.1357	0.0026
YER144C	UBP5	YHR050W	SMF2	0.1352	0.0185
YBR229C	ROT2	YJR008W	MHO1	0.1346	-0.0563
YDR528W	HLR1	YPL001W	HAT1	0.1346	0.0897

YER116C	SLX8	YOL073C	DSC2	0.1342	0.0006
YJL187C	SWE1	YBR071W	YBR071W	0.1341	0.0366
YCL026C-A	FRM2	YGR152C	RSR1	0.1337	-0.0748
YDR319C	YFT2	YGL226C-A	OST5	0.1336	0.0416
YDR515W	SLF1	YIL142W	CCT2	0.1333	0.0085
YMR215W	GAS3	YER116C	SLX8	0.1329	-0.2465
YAL051W	OAF1	YPL016W	SWI1	0.1328	0.0328
YBL017C	PEP1	YJL140W	RPB4	0.1328	-0.0623
YBL020W	RFT1	YDR515W	SLF1	0.1327	0.0012
YFR011C	MIC19	YGL035C	MIG1	0.1326	0.0686
YIL158W	AIM20	YJL036W	SNX4	0.1322	0.0272
YOR054C	VHS3	YFR013W	IOC3	0.1320	0.1294
YDL155W	CLB3	YHL048W	COS8	0.1318	-0.0468
YDR180W	SCC2	YHR023W	MYO1	0.1318	0.0158
YBR225W	YBR225W	YMR112C	MED11	0.1315	-0.1435
YJL058C	BIT61	YDL002C	NHP10	0.1312	-0.0424
YNL024C	EFM6	YBR071W	YBR071W	0.1310	-0.0468
YBL014C	RRN6	YPL144W	POC4	0.1309	0.1464
YPL234C	VMA11	YDR144C	MKC7	0.1306	-0.0149
YOR051C	ETT1	YLR449W	FPR4	0.1305	0.0230
YJR056C	YJR056C	YHR027C	RPN1	0.1303	-0.0011
YAL051W	OAF1	YJR032W	CPR7	0.1296	-0.0673
YEL055C	POL5	YOL164W	BDS1	0.1296	-0.0630
YDL164C	CDC9	YBR008C	FLR1	0.1289	0.0318
YDL002C	NHP10	YLR430W	SEN1	0.1286	-0.1051
YOL113W	SKM1	YGL025C	PGD1	0.1284	0.1718
YMR046C	YMR046C	YFL047W	RGD2	0.1283	0.0105
YLR248W	RCK2	YNL138W	SRV2	0.1280	0.0079
YLL040C	VPS13	YDL217C	TIM22	0.1279	-0.0269
YGL201C	MCM6	YML048W	GSF2	0.1278	-0.0371
YJL163C	YJL163C	YNL329C	PEX6	0.1278	-0.0787
YKR051W	YKR051W	YOL147C	PEX11	0.1278	-0.1325
YOR092W	ECM3	YDR383C	NKP1	0.1277	0.0022
YFR042W	KEG1	YLR180W	SAM1	0.1272	-0.0248
YFL018C	LPD1	YKL092C	BUD2	0.1271	0.1030
YLR342W	FKS1	YOR131C	YOR131C	0.1271	-0.0985
YFR047C	BNA6	YDR180W	SCC2	0.1270	-0.0928
YMR067C	UBX4	YER116C	SLX8	0.1269	-0.1169
YNL135C	FPR1	YLR172C	DPH5	0.1269	0.0243
YBR222C	PCS60	YDL167C	NRP1	0.1267	-0.1058
YBR154C	RPB5	YOR144C	ELG1	0.1266	0.0077
YPL012W	RRP12	YLR096W	KIN2	0.1266	0.1889
YGL017W	ATE1	YDL103C	QRI1	0.1265	0.0357
YFR013W	IOC3	YMR123W	PKR1	0.1261	-0.0177
YBL079W	NUP170	YFR004W	RPN11	0.1259	0.0498
YGR157W	CHO2	YLR257W	YLR257W	0.1259	0.0261
YBR225W	YBR225W	YJR097W	JJJ3	0.1258	-0.2587

YOL018C	TLG2	YFR013W	IOC3	0.1254	0.0426
YDL147W	RPN5	YBR225W	YBR225W	0.1253	-0.0023
YPL001W	HAT1	YJL158C	CIS3	0.1252	0.0159
YBL079W	NUP170	YBR069C	TAT1	0.1248	0.3095
YGL207W	SPT16	YIL009W	FAA3	0.1248	0.0265
YNL215W	IES2	YML036W	CGI121	0.1246	-0.0608
YBR087W	RFC5	YOR371C	GPB1	0.1245	-0.0939
YML098W	TAF13	YBR069C	TAT1	0.1245	0.0562
YDR144C	MKC7	YIR026C	YVH1	0.1244	0.0697
YLR442C	SIR3	YKL125W	RRN3	0.1244	0.0093
YPL071C	YPL071C	YDR497C	ITR1	0.1243	0.0204
YDR524C	AGE1	YKR051W	YKR051W	0.1242	0.0042
YPL233W	NSL1	YER116C	SLX8	0.1242	0.0447
YDL103C	QRI1	YGR158C	MTR3	0.1240	-0.0057
YDL150W	RPC53	YJR036C	HUL4	0.1239	-0.1303
YHR016C	YSC84	YLR248W	RCK2	0.1239	0.1196
YJL178C	ATG27	YML016C	PPZ1	0.1238	-0.0506
YKL160W	ELF1	YNL329C	PEX6	0.1238	-0.0263
YHR209W	CRG1	YBR071W	YBR071W	0.1237	0.0776
YAL009W	SPO7	YOR088W	YOR088W	0.1236	-0.0319
YHR039C	MSC7	YGR258C	RAD2	0.1234	-0.0688
YOR131C	YOR131C	YGR001C	EFM5	0.1234	0.0816
YML007W	YAP1	YIL066C	RNR3	0.1230	0.0208
YMR215W	GAS3	YCR031C	RPS14a	0.1230	0.0226
YGL226W	MTC3	YIL166C	YIL166C	0.1229	-0.0784
YBR037C	SCO1	YNL041C	COG6	0.1228	-0.0173
YBL029W	YBL029W	YPL058C	PDR12	0.1226	0.0448
YBL030C	PET9	YEL071W	DLD3	0.1225	-0.0522
YJR068W	RFC2	YHR063C	PAN5	0.1225	0.0297
YEL058W	PCM1	YBR229C	ROT2	0.1223	-0.0281
YLL061W	MMP1	YJL163C	YJL163C	0.1222	-0.0299
YBL040C	ERD2	YOL088C	MPD2	0.1219	-0.0547
YJL140W	RPB4	YBR061C	TRM7	0.1219	-0.0046
YGL004C	RPN14	YKR080W	MTD1	0.1218	0.0005
YLR368W	MDM30	YMR046C	YMR046C	0.1217	0.0832
YER023W	PRO3	YOR142W	LSC1	0.1216	0.0211
YKL184W	SPE1	YDR441C	APT2	0.1211	0.0364
YKL073W	LHS1	YDL167C	NRP1	0.1211	-0.0244
YPL001W	HAT1	YLR342W	FKS1	0.1211	0.0114
YKL184W	SPE1	YDR351W	SBE2	0.1210	0.0806
YKL125W	RRN3	YER023W	PRO3	0.1209	-0.0331
YJL178C	ATG27	YAL009W	SPO7	0.1207	0.0471
YMR112C	MED11	YLR375W	STP3	0.1207	-0.0403
YDL164C	CDC9	YER144C	UBP5	0.1205	-0.0323
YNL280C	ERG24	YLR443W	ECM7	0.1200	0.1108
YGL004C	RPN14	YLL024C	SSA2	0.1198	-0.0668
YGR184C	UBR1	YLR455W	PDP3	0.1189	-0.0181

YDL150W	RPC53	YCL009C	ILV6	0.1187	-0.0327
YPL016W	SWI1	YCL024W	KCC4	0.1185	0.0279
YJL187C	SWE1	YFR047C	BNA6	0.1182	-0.1002
YJL183W	MNN11	YIL061C	SNP1	0.1180	-0.0757
YIL133C	RPL16a	YAL056W	GPB2	0.1178	-0.1246
YGR168C	YGR168C	YOL164W	BDS1	0.1176	-0.0045
YGR194C	XKS1	YBR037C	SCO1	0.1176	0.0561
YHR027C	RPN1	YER144C	UBP5	0.1172	-0.0792
YLR330W	CHS5	YML007W	YAP1	0.1171	-0.0065
YGR149W	GPC1	YJL178C	ATG27	0.1167	0.0502
YML096W	YML096W	YDL217C	TIM22	0.1166	0.0082
YDR351W	SBE2	YDR180W	SCC2	0.1162	0.0094
YPL016W	SWI1	YDR319C	YFT2	0.1161	-0.0430
YDL150W	RPC53	YGR258C	RAD2	0.1160	-0.0063
YJR073C	OPI3	YER049W	TPA1	0.1159	-0.0427
YLL024C	SSA2	YDR351W	SBE2	0.1158	-0.0276
YHR209W	CRG1	YLR047C	FRE8	0.1153	-0.0869
YGR208W	SER2	YGL176C	YGL176C	0.1152	0.0671
YHR027C	RPN1	YDR144C	MKC7	0.1148	0.0362
YEL076W-C	Unknown	YOL092W	YPQ1	0.1145	-0.0026
YDR531W	CAB1	YOL092W	YPQ1	0.1144	0.0219
YNR034W	SOL1	YLR342W	FKS1	0.1144	-0.0035
YMR115W	MGR3	YJL047C	RTT101	0.1141	0.1423
YCR041W	YCR041W	YDL167C	NRP1	0.1140	-0.0444
YOL073C	DSC2	YOR264W	DSE3	0.1140	-0.1088
YBR132C	AGP2	YOR092W	ECM3	0.1136	0.0210
YGR207C	CIR1	YOL088C	MPD2	0.1136	-0.0524
YGL226C-A	OST5	YNL280C	ERG24	0.1135	0.0096
YIL166C	YIL166C	YMR112C	MED11	0.1135	-0.0699
YML072C	TCB3	YKL067W	YNK1	0.1135	-0.0296
YIL133C	RPL16a	YGR001C	EFM5	0.1127	0.2798
YGR157W	CHO2	YGL242C	YGL242C	0.1123	0.1404
YER046W	SPO73	YML016C	PPZ1	0.1122	-0.0299
YDL002C	NHP10	YBR239C	ERT1	0.1121	-0.1387
YOL164W	BDS1	YOL147C	PEX11	0.1120	0.0199
YDR441C	APT2	YBR069C	TAT1	0.1117	0.0023
YIR028W	DAL4	YPL144W	POC4	0.1116	-0.1103
YPL144W	POC4	YOR264W	DSE3	0.1111	0.1055
YLR206W	ENT2	YDL047W	SIT4	0.1106	0.0181
YDL089W	NUR1	YDL150W	RPC53	0.1102	0.0138
YPR196W	YPR196W	YGR149W	GPC1	0.1098	-0.0042
YLR375W	STP3	YFR013W	IOC3	0.1097	-0.0181
YBR193C	MED8	YOL147C	PEX11	0.1094	-0.0610
YER023W	PRO3	YDL164C	CDC9	0.1086	0.0507
YDR321W	ASP1	YOR264W	DSE3	0.1085	0.1117
YGR157W	CHO2	YLR046C	YLR046C	0.1085	0.0108
YCR071C	IMG2	YIL009W	FAA3	0.1084	-0.0110

YHL007C	STE20	YOR051C	ETT1	0.1083	0.0014
YEL058W	PCM1	YLR426W	TDA5	0.1080	0.0985
YLL035W	GRC3	YFR037C	RSC8	0.1080	-0.0039
YLR342W	FKS1	YML110C	COQ5	0.1079	-0.0319
YCL018W	LEU2	YBR071W	YBR071W	0.1078	-0.0197
YLR449W	FPR4	YLR430W	SEN1	0.1076	0.0407
YML110C	COQ5	YLR455W	PDP3	0.1076	0.0355
YDR178W	SDH4	YPL001W	HAT1	0.1064	-0.0957
YHR039C	MSC7	YER023W	PRO3	0.1061	0.0076
YJL047C	RTT101	YJL212C	OPT1	0.1060	-0.0155
YKR080W	MTD1	YOR196C	LIP5	0.1058	-0.0344
YLR368W	MDM30	YHR063C	PAN5	0.1057	0.0018
YLR455W	PDP3	YDR420W	HKR1	0.1057	0.0670
YHR063C	PAN5	YML091C	RPM2	0.1053	-0.1217
YLR455W	PDP3	YOR088W	YOR088W	0.1050	-0.1298
YDR351W	SBE2	YDL132W	CDC53	0.1049	0.1293
YLR389C	STE23	YOR131C	YOR131C	0.1044	-0.0017
YGL017W	ATE1	YJR073C	OPI3	0.1043	0.0066
YER020W	GPA2	YHR063C	PAN5	0.1042	-0.0306
YOR043W	WHI2	YBR069C	TAT1	0.1042	-0.1112
YER185W	PUG1	YDR420W	HKR1	0.1040	-0.0811
YJR032W	CPR7	YPL144W	POC4	0.1037	0.0951
YJL204C	RCY1	YOR088W	YOR088W	0.1035	0.1288
YLR258W	GSY2	YER144C	UBP5	0.1034	0.1149
YLR231C	BNA5	YMR112C	MED11	0.1033	0.1143
YGL178W	MPT5	YDL002C	NHP10	0.1032	-0.0272
YLR287C	YLR287C	YOR051C	ETT1	0.1031	0.1089
YPR080W	TEF1	YOL026C	MIM1	0.1028	0.0010
YAL056W	GPB2	YDL150W	RPC53	0.1027	-0.0465
YHR016C	YSC84	YML036W	CGI121	0.1026	-0.0583
YJL126W	NIT2	YHR027C	RPN1	0.1024	-0.0074
YMR046C	YMR046C	YJL140W	RPB4	0.1023	-0.0303
YDL135C	RDI1	YLL035W	GRC3	0.1019	0.0552
YML110C	COQ5	YDR420W	HKR1	0.1010	-0.1156
YOL093W	TRM10	YML071C	COG8	0.1009	0.0454
YJL047C	RTT101	YBR071W	YBR071W	0.1006	-0.0623
YOL089C	HAL9	YDR319C	YFT2	0.1003	-0.0038
YDR528W	HLR1	YOL147C	PEX11	0.1001	-0.0095
YJL051W	IRC8	YLR442C	SIR3	0.1001	-0.0068
YJL126W	NIT2	YIL043C	CBR1	0.1000	-0.0329
YFR047C	BNA6	YJR032W	CPR7	0.0999	-0.1186
YMR115W	MGR3	YBR037C	SCO1	0.0999	-0.0417
YJR056C	YJR056C	YML036W	CGI121	0.0997	0.0327
YHL048W	COS8	YJL036W	SNX4	0.0994	0.0164
YOR101W	RAS1	YMR209C	YMR209C	0.0993	-0.0085
YML013W	UBX2	YHR063C	PAN5	0.0992	-0.0644
YIL089W	YIL089W	YML110C	COQ5	0.0991	-0.0198

YKL116C	PRR1	YJL051W	IRC8	0.0991	0.0098
YML091C	RPM2	YMR112C	MED11	0.0988	0.0566
YLR257W	YLR257W	YGR150C	CCM1	0.0987	0.0102
YLR443W	ECM7	YOL147C	PEX11	0.0986	0.0634
YNL326C	PFA3	YOL073C	DSC2	0.0984	-0.0190
YBR132C	AGP2	YDR420W	HKR1	0.0979	-0.0167
YIL111W	COX5b	YCR031C	RPS14a	0.0979	-0.0681
YMR062C	ARG7	YMR112C	MED11	0.0978	-0.0103
YFL016C	MDJ1	YDR515W	SLF1	0.0977	0.0867
YOR142W	LSC1	YGR152C	RSR1	0.0974	-0.1082
YIL015C-A	YIL015C-A	YOL088C	MPD2	0.0973	0.0633
YOR389W	YOR389W	YDR515W	SLF1	0.0973	0.0070
YFL049W	SWP82	YIL043C	CBR1	0.0972	-0.0058
YGL053W	PRM8	YLR047C	FRE8	0.0972	-0.0381
YOL147C	PEX11	YOL026C	MIM1	0.0970	-0.0567
YCL009C	ILV6	YML048W	GSF2	0.0968	0.0254
YJR056C	YJR056C	YOR092W	ECM3	0.0965	0.0298
YOR127W	RGA1	YOR264W	DSE3	0.0962	-0.0052
YHR063C	PAN5	YNL135C	FPR1	0.0961	-0.0350
YOL018C	TLG2	YMR209C	YMR209C	0.0960	-0.0472
YAL056W	GPB2	YOL092W	YPQ1	0.0957	0.0387
YIL119C	RPI1	YDR515W	SLF1	0.0956	0.0010
YCL039W	GID7	YDL103C	QRI1	0.0953	0.0017
YMR192W	GYL1	YPL144W	POC4	0.0953	0.0470
YLR057W	MNL2	YOL130W	ALR1	0.0952	0.0858
YIL165C	YIL165C	YGR152C	RSR1	0.0951	-0.0485
YKL184W	SPE1	YJR032W	CPR7	0.0951	0.1620
YCR041W	YCR041W	YOL147C	PEX11	0.0949	0.0900
YDR058C	TGL2	YBR168W	PEX32	0.0942	-0.1533
YPL226W	NEW1	YOR033C	EXO1	0.0942	0.0507
YLL035W	GRC3	YER186C	YER186C	0.0941	-0.0218
YDL147W	RPN5	YML036W	CGI121	0.0937	-0.0182
YOL026C	MIM1	YFR004W	RPN11	0.0934	0.0053
YBL089W	AVT5	YHR063C	PAN5	0.0928	-0.0334
YBR077C	SLM4	YDL047W	SIT4	0.0928	-0.0458
YGR168C	YGR168C	YOR092W	ECM3	0.0926	0.0054
YJL051W	IRC8	YGL176C	YGL176C	0.0925	0.0699
YKL021C	MAK11	YOL153C	YOL153C	0.0924	-0.0588
YDR339C	FCF1	YGR006W	PRP18	0.0923	-0.1952
YML110C	COQ5	YJL163C	YJL163C	0.0921	0.0302
YHL048W	COS8	YOR088W	YOR088W	0.0916	-0.0891
YFR013W	IOC3	YHR050W	SMF2	0.0915	-0.0098
YGR207C	CIR1	YJL171C	TOH1	0.0911	0.0709
YOL092W	YPQ1	YLL035W	GRC3	0.0911	0.0259
YMR112C	MED11	YMR158W	MRPS8	0.0909	0.0091
YFR047C	BNA6	YBR196C	PGI1	0.0908	0.0115
YML071C	COG8	YBR071W	YBR071W	0.0908	0.0785

YNL022C	RCM1	YER026C	CHO1	0.0903	-0.0926
YER185W	PUG1	YLR047C	FRE8	0.0900	-0.0021
YLR231C	BNA5	YBR008C	FLR1	0.0899	-0.0496
YLR206W	ENT2	YOL147C	PEX11	0.0897	-0.0070
YIL066C	RNR3	YPL144W	POC4	0.0894	-0.0395
YBR121C	GRS1	YJR086W	STE18	0.0893	-0.1601
YIL043C	CBR1	YDR420W	HKR1	0.0893	0.0241
YFR047C	BNA6	YHL007C	STE20	0.0887	0.0811
YDR144C	MKC7	YDR420W	HKR1	0.0886	0.2183
YGR194C	XKS1	YDR497C	ITR1	0.0883	-0.0384
YGR289C	MAL11	YDL217C	TIM22	0.0880	-0.0699
YNL200C	NNR1	YGR258C	RAD2	0.0879	0.1649
YPL237W	SUI3	YLR237W	THI7	0.0876	-0.0987
YHR054C	YHR054C	YHR027C	RPN1	0.0873	-0.0351
YFR037C	RSC8	YER116C	SLX8	0.0861	0.0583
YOR080W	DIA2	YHL007C	STE20	0.0859	0.0293
YKL125W	RRN3	YDR530C	APA2	0.0852	-0.1170
YLL024C	SSA2	YGR194C	XKS1	0.0851	0.0000
YIL111W	COX5b	YOL147C	PEX11	0.0847	0.0204
YOR144C	ELG1	YHR043C	DOG2	0.0839	-0.2641
YFR042W	KEG1	YOL088C	MPD2	0.0829	0.0325
YDR530C	APA2	YMR112C	MED11	0.0822	-0.0992
YFL056C	AAD6	YPL001W	HAT1	0.0822	-0.0385
YFL018C	LPD1	YGL051W	MST27	0.0822	-0.0203
YBR067C	TIP1	YKL184W	SPE1	0.0818	0.0307
YBR037C	SCO1	YKL073W	LHS1	0.0815	-0.0213
YNR036C	MRPS12	YMR192W	GYL1	0.0815	-0.2706
YJR068W	RFC2	YML036W	CGI121	0.0814	-0.1064
YBR037C	SCO1	YOR371C	GPB1	0.0812	-0.0067
YKL110C	KTI12	YGL051W	MST27	0.0807	0.0463
YMR192W	GYL1	YDL002C	NHP10	0.0805	0.0506
YBR067C	TIP1	YDL164C	CDC9	0.0802	-0.0096
YKL021C	MAK11	YJR097W	JJJ3	0.0795	0.0573
YNL138W	SRV2	YLL035W	GRC3	0.0795	-0.0022
YGR194C	XKS1	YDR530C	APA2	0.0792	0.0959
YIL112W	HOS4	YDR420W	HKR1	0.0791	0.0026
YIR026C	YVH1	YDR441C	APT2	0.0791	0.0370
YNL046W	YNL046W	YDL103C	QRI1	0.0789	0.0305
YOR371C	GPB1	YDL167C	NRP1	0.0788	-0.0333
YML036W	CGI121	YPL009C	RQC2	0.0784	0.0439
YNL242W	ATG2	YDR420W	HKR1	0.0782	0.0942
YAL051W	OAF1	YDR319C	YFT2	0.0778	-0.0493
YMR046C	YMR046C	YLR342W	FKS1	0.0778	-0.0001
YBR067C	TIP1	YLR180W	SAM1	0.0776	-0.0315
YOR251C	TUM1	YGL051W	MST27	0.0775	-0.0921
YPR022C	SDD4	YIL009W	FAA3	0.0775	0.0121
YJL051W	IRC8	YNL138W	SRV2	0.0771	0.0887

YJL187C	SWE1	YBL029W	YBL029W	0.0765	-0.0384
YOL089C	HAL9	YKL184W	SPE1	0.0765	0.0316
YDL164C	CDC9	YLL035W	GRC3	0.0760	0.0548
YDL164C	CDC9	YHR027C	RPN1	0.0759	0.1641
YGR207C	CIR1	YBR239C	ERT1	0.0756	-0.0299
YEL058W	PCM1	YDL217C	TIM22	0.0753	-0.0607
YKR085C	MRPL20	YML091C	RPM2	0.0753	0.0561
YGL192W	IME4	YER186C	YER186C	0.0751	0.0382
YOL153C	YOL153C	YJL212C	OPT1	0.0748	0.1298
YHR209W	CRG1	YML036W	CGI121	0.0744	-0.0017
YLR258W	GSY2	YDR319C	YFT2	0.0742	-0.0236
YDR178W	SDH4	YDR144C	MKC7	0.0741	-0.0921
YER020W	GPA2	YDR515W	SLF1	0.0738	-0.1029
YKL021C	MAK11	YDL211C	YDL211C	0.0737	0.0035
YJL036W	SNX4	YIL066C	RNR3	0.0736	-0.0002
YOR142W	LSC1	YDL164C	CDC9	0.0729	-0.0781
YNL200C	NNR1	YCR083W	TRX3	0.0726	0.1043
YBL020W	RFT1	YLL035W	GRC3	0.0722	0.0645
YDL135C	RDI1	YJL163C	YJL163C	0.0721	0.0026
YBL017C	PEP1	YDL217C	TIM22	0.0719	-0.0435
YMR209C	YMR209C	YDR515W	SLF1	0.0717	-0.0068
YCR071C	IMG2	YBR071W	YBR071W	0.0710	0.1074
YGL017W	ATE1	YFR004W	RPN11	0.0708	-0.0012
YOR127W	RGA1	YDR515W	SLF1	0.0708	-0.0539
YJL126W	NIT2	YNL138W	SRV2	0.0706	-0.0772
YPL234C	VMA11	YMR067C	UBX4	0.0701	-0.0294
YML071C	COG8	YDR367W	KEI1	0.0693	-0.0469
YGR288W	MAL13	YGR211W	ZPR1	0.0692	-0.0094
YEL068C	YEL068C	YER116C	SLX8	0.0691	0.0386
YGL208W	SIP2	YER144C	UBP5	0.0687	0.0099
YGR194C	XKS1	YJL051W	IRC8	0.0687	0.1019
YJR084W	YJR084W	YGR150C	CCM1	0.0687	-0.0176
YGR258C	RAD2	YIL009W	FAA3	0.0683	0.0924
YKR080W	MTD1	YDL002C	NHP10	0.0682	0.0421
YPL001W	HAT1	YJR032W	CPR7	0.0679	0.0575
YKL067W	YNK1	YER026C	CHO1	0.0678	0.0258
YDR441C	APT2	YMR067C	UBX4	0.0677	-0.0122
YLR237W	THI7	YDR319C	YFT2	0.0668	0.0268
YCR071C	IMG2	YLR172C	DPH5	0.0662	-0.0170
YBR274W	CHK1	YML110C	COQ5	0.0661	0.0358
YIL046W	MET30	YFR013W	IOC3	0.0660	-0.0281
YLR046C	YLR046C	YGL051W	MST27	0.0647	0.0532
YJL208C	NUC1	YOL026C	MIM1	0.0645	0.0887
YPL237W	SUI3	YLR176C	RFX1	0.0642	-0.0164
YDL227C	Ho	YIL009W	FAA3	0.0640	0.0314
YOR142W	LSC1	YHR063C	PAN5	0.0623	0.0005
YHR104W	GRE3	YGR149W	GPC1	0.0618	0.0157

YNL124W	NAF1	YHR027C	RPN1	0.0618	-0.0229
YJL171C	TOH1	YNL124W	NAF1	0.0616	0.0321
YPL233W	NSL1	YDR531W	CAB1	0.0616	-0.0018
YKR085C	MRPL20	YDL047W	SIT4	0.0613	-0.0007
YBR168W	PEX32	YGR194C	XKS1	0.0611	-0.0150
YMR010W	ANY1	YJL051W	IRC8	0.0606	-0.0072
YBL030C	PET9	YDR351W	SBE2	0.0604	0.0208
YBR161W	CSH1	YLL035W	GRC3	0.0602	-0.0942
YNL024C	EFM6	YOL147C	PEX11	0.0595	-0.0039
YJL178C	ATG27	YER182W	FMP10	0.0593	-0.0371
YBR077C	SLM4	YIL009W	FAA3	0.0583	-0.0352
YLR442C	SIR3	YDR180W	SCC2	0.0583	0.0476
YDR513W	GRX2	YDL047W	SIT4	0.0582	0.0282
YIR022W	SEC11	YDR351W	SBE2	0.0580	0.0251
YBR163W	EXO5	YDL217C	TIM22	0.0578	-0.0023
YGL208W	SIP2	YBR069C	TAT1	0.0573	0.0725
YLR435W	TSR2	YBR087W	RFC5	0.0565	-0.0079
YDR058C	TGL2	YIL112W	HOS4	0.0561	0.0033
YGL208W	SIP2	YLL035W	GRC3	0.0550	-0.0885
YDL147W	RPN5	YDR319C	YFT2	0.0545	0.1166
YMR046C	YMR046C	YDR144C	MKC7	0.0539	-0.0462
YGR028W	MSP1	YHR027C	RPN1	0.0534	-0.0507
YML071C	COG8	YBR168W	PEX32	0.0534	0.0016
YOL097C	WRS1	YPL001W	HAT1	0.0534	0.2336
YBR071W	YBR071W	YDL150W	RPC53	0.0529	0.0209
YMR209C	YMR209C	YGR150C	CCM1	0.0521	-0.0285
YBR249C	ARO4	YER144C	UBP5	0.0519	-0.1286
YJR040W	GEF1	YER116C	SLX8	0.0519	-0.0285
YDL164C	CDC9	YDR319C	YFT2	0.0517	0.1662
YKL184W	SPE1	YIL066C	RNR3	0.0513	0.0304
YOL147C	PEX11	YKL184W	SPE1	0.0511	0.0425
YOR127W	RGA1	YMR112C	MED11	0.0504	-0.0492
YMR062C	ARG7	YDL150W	RPC53	0.0495	-0.0078
YOR043W	WHI2	YGL051W	MST27	0.0489	0.0473
YOL113W	SKM1	YOR264W	DSE3	0.0486	-0.0731
YGR023W	MTL1	YFR013W	IOC3	0.0482	0.0146
YIL133C	RPL16a	YGR184C	UBR1	0.0479	-0.0867
YHR016C	YSC84	YIL142W	CCT2	0.0477	-0.0442
YCR071C	IMG2	YGR149W	GPC1	0.0475	-0.0259
YCR083W	TRX3	YNL124W	NAF1	0.0473	-0.0151
YBR225W	YBR225W	YDR531W	CAB1	0.0464	-0.0421
YGR168C	YGR168C	YFR013W	IOC3	0.0461	0.0251
YJR032W	CPR7	YER144C	UBP5	0.0458	-0.0100
YOL104C	NDJ1	YML110C	COQ5	0.0454	-0.0176
YBL017C	PEP1	YPL001W	HAT1	0.0445	-0.0891
YER186C	YER186C	YOL088C	MPD2	0.0441	0.1510
YBR069C	TAT1	YIL009W	FAA3	0.0436	-0.0245

YOR033C	EXO1	YGL051W	MST27	0.0433	-0.0835
YEL058W	PCM1	YIL009W	FAA3	0.0432	0.0392
YBR157C	ICS2	YMR215W	GAS3	0.0427	0.0013
YLR096W	KIN2	YFR004W	RPN11	0.0427	-0.0726
YPL001W	HAT1	YDR351W	SBE2	0.0425	0.0008
YPL064C	CWC27	YFR013W	IOC3	0.0424	0.0267
YDL171C	GLT1	YMR215W	GAS3	0.0422	-0.1285
YER022W	SRB4	YML036W	CGI121	0.0422	0.0456
YGL004C	RPN14	YOR264W	DSE3	0.0411	0.0262
YIL165C	YIL165C	YBR069C	TAT1	0.0406	-0.0395
YCR083W	TRX3	YOR131C	YOR131C	0.0405	0.0075
YLL028W	TPO1	YOL026C	MIM1	0.0397	-0.1062
YPL237W	SUI3	YEL020W-A	TIM9	0.0396	0.0155
YDL171C	GLT1	YOR088W	YOR088W	0.0391	0.0321
YGR157W	CHO2	YOL026C	MIM1	0.0385	0.0335
YJR056C	YJR056C	YMR215W	GAS3	0.0384	-0.0007
YHR027C	RPN1	YFR013W	IOC3	0.0382	0.0016
YJR025C	BNA1	YHR063C	PAN5	0.0382	0.0001
YNL024C	EFM6	YML036W	CGI121	0.0372	-0.0125
YOR048C	RAT1	YDR420W	HKR1	0.0369	-0.1612
YJR074W	MOG1	YGR152C	RSR1	0.0340	-0.0580
YDL116W	NUP84	YGR149W	GPC1	0.0339	-0.0141
YML079W	YML079W	YDR515W	SLF1	0.0333	-0.0486
YLR206W	ENT2	YIL009W	FAA3	0.0329	-0.0131
YNL278W	CAF120	YOL026C	MIM1	0.0328	-0.0503
YIL171W	YIL171W	YOL147C	PEX11	0.0319	-0.0306
YLR257W	YLR257W	YOR264W	DSE3	0.0306	0.0046
YFR037C	RSC8	YDR319C	YFT2	0.0294	-0.0304
YLL040C	VPS13	YIL009W	FAA3	0.0290	-0.0311
YDR528W	HLR1	YBR069C	TAT1	0.0276	0.0004
YHR039C	MSC7	YDL111C	RRP42	0.0264	0.0257
YPL234C	VMA11	YFR013W	IOC3	0.0258	0.0333
YJR108W	ABM1	YER116C	SLX8	0.0251	0.0007
YLR257W	YLR257W	YML110C	COQ5	0.0248	-0.0932
YNR034W	SOL1	YIL009W	FAA3	0.0247	0.0424
YBL007C	SLA1	YML036W	CGI121	0.0238	-0.0460
YLR449W	FPR4	YOL026C	MIM1	0.0226	0.1391
YDL217C	TIM22	YIL009W	FAA3	0.0201	-0.0628
YLR426W	TDA5	YDR420W	HKR1	0.0192	0.0298
YLL040C	VPS13	YLR047C	FRE8	0.0184	0.0082
YDL089W	NUR1	YFR013W	IOC3	0.0181	-0.0312
YGR209C	TRX2	YIL009W	FAA3	0.0156	0.0368
YOL153C	YOL153C	YOR088W	YOR088W	0.0104	0.0795
YOL153C	YOL153C	YBR071W	YBR071W	0.0079	-0.0950
YGR028W	MSP1	YDR319C	YFT2	0.0075	-0.0268

Table S3: List of subnetwork nodes.

Subnetwork 1	Subnetwork 2	Subnetwork 3	Subnetwork 4	Subnetwork 5	Subnetwork 6
MHO1	NUC1	RPI1	PPZ1	YBR137W	CCT2
ITR1	PRO3	MKC7	YPL071C	YMD8	RPT5
ARG7	RFT1	RGD2	AGP2	TPS1	SEN1
YGR012W	DMA1	MED11	PIH1	XKS1	RPN1
ILV6	SLM4	ATE1	YHR033W	PHM8	RPN5
SPE1	ROT2	NUR1	MTD1	GRE3	SPT16
CAB1	PEP1	RTC2	CIS3	SPI1	CDC53
LYS14	TUM1	TIP1	ISC1	YBR071W	RPN6
SDH4	SLA1	SST2	YNL046W	YBR225W	SMF2
RPB4	GRS1	HOS4	YAP1	YNL200C	
CIR1	IMG2	ICS2	DSE3		
ARO4	MRPS8	YML096W	COX5b		
YIL165C	PGI1	BNA6	KRE1		
FCF1	PRM8	BNA5	URA8		
YOR131C	PCS60	BNA1	MLF3		
AIM13	IME4	ECM3	EDC2		
OPI3	MDJ1				
YHI9	FMP10				
RSC8	FLR1				
DLD3	SCO1				
MOG1	NUP170				
LIP5	TIM9				
LPD1	MRPL20				
MNT2	SIP2				
ERT1					
NHP10					
CHO1					
LEU2					
PHO85					
PAN5					
LSC1					
DOG2					
THI7					
YLR455W					
PET9					
SBE2					
OPT1					

Subnetwork 7	Subnetwork 8	Subnetwork 9	Subnetwork 10	Subnetwork 11	Subnetwork 12
MSP1	MTR3	ELF1	YKR051W	YJL171C	KIN2
YDL211C	PCM1	EMC2	EXO1	YCR041W	RPM2
LHS1	MSC7	UBP5	SRB4	VMA11	YIL015C-A
YLR257W	PRR1	STE18	AVT2	YDR134C	YMR046C
SNP1	TAE2	DIA2	RNR3	PER33	MIG1
HLR1	APM4	OST5	ASP3-1		
SNX4	ERG3	YGL193C			
COG8	ERG24				

Table S4: Gene ontology enrichment results using DAVID analysis of subnetworks.

Subnetwork 1

Category	Term	Count	PValue	Benjamini	FDR
GOTERM_BP_FAT	GO:0009309~amine biosynthetic process	7	1.23E-04	3.99E-02	1.66E-01
GOTERM_BP_FAT	GO:0046394~carboxylic acid biosynthetic pr	7	3.51E-04	5.66E-02	4.75E-01
GOTERM_BP_FAT	GO:0016053~organic acid biosynthetic proce	7	3.51E-04	5.66E-02	4.75E-01
GOTERM_BP_FAT	GO:0008652~cellular amino acid biosyntheti	6	8.64E-04	9.12E-02	1.17E+00
SP_PIR_KEYWORDS	amino-acid biosynthesis	5	9.77E-04	9.22E-02	1.08E+00
GOTERM_BP_FAT	GO:0044271~nitrogen compound biosynthet	8	1.67E-03	1.29E-01	2.24E+00
GOTERM_MF_FAT	GO:0048037~cofactor binding	6	2.18E-03	2.50E-01	2.51E+00
GOTERM_CC_FAT	GO:0009295~nucleoid	3	4.75E-03	2.90E-01	4.84E+00
GOTERM_CC_FAT	GO:0042645~mitochondrial nucleoid	3	4.75E-03	2.90E-01	4.84E+00
GOTERM_BP_FAT	GO:0051186~cofactor metabolic process	6	5.38E-03	3.01E-01	7.05E+00
GOTERM_BP_FAT	GO:0009081~branched chain family amino a	3	7.49E-03	3.40E-01	9.69E+00
GOTERM_BP_FAT	GO:0006732~coenzyme metabolic process	5	9.73E-03	3.71E-01	1.24E+01
GOTERM_BP_FAT	GO:0055114~oxidation reduction	7	1.34E-02	4.28E-01	1.67E+01
GOTERM_CC_FAT	GO:0005625~soluble fraction	3	1.98E-02	5.13E-01	1.88E+01
GOTERM_BP_FAT	GO:0051188~cofactor biosynthetic process	4	2.27E-02	5.71E-01	2.67E+01
GOTERM_MF_FAT	GO:0050662~coenzyme binding	4	2.36E-02	7.93E-01	2.43E+01
GOTERM_CC_FAT	GO:0044429~mitochondrial part	7	2.48E-02	4.53E-01	2.30E+01
PIR_SUPERFAMILY	PIRSF000895:phosphoglycolate phosphatase	2	2.69E-02	5.22E-01	2.03E+01
KEGG_PATHWAY	sce00020:Citrate cycle (TCA cycle)	3	3.40E-02	5.48E-01	2.40E+01
GOTERM_BP_FAT	GO:0015931~nucleobase, nucleoside, nucleo	4	4.04E-02	7.46E-01	4.29E+01
GOTERM_BP_FAT	GO:0006551~leucine metabolic process	2	4.51E-02	7.52E-01	4.65E+01
INTERPRO	IPR006402:HAD-superfamily hydrolase, sub	2	4.69E-02	9.75E-01	3.98E+01

Subnetwork 2

Category	Term	Count	PValue	Benjamini	FDR
SP_PIR_KEYWORDS	mitochondrion	9	1.59E-03	1.27E-01	1.70E+00
GOTERM_BP_FAT	GO:0007005~mitochondrion organization	6	4.25E-03	7.23E-01	5.54E+00
GOTERM_BP_FAT	GO:0032543~mitochondrial translation	4	5.89E-03	5.89E-01	7.60E+00
GOTERM_CC_FAT	GO:0005739~mitochondrion	11	6.07E-03	4.25E-01	6.42E+00
SP_PIR_KEYWORDS	membrane	13	1.06E-02	3.65E-01	1.09E+01
GOTERM_CC_FAT	GO:0044429~mitochondrial part	7	1.51E-02	4.99E-01	1.53E+01
GOTERM_CC_FAT	GO:0000313~organellar ribosome	3	4.04E-02	7.13E-01	3.62E+01
GOTERM_CC_FAT	GO:0005761~mitochondrial ribosome	3	4.04E-02	7.13E-01	3.62E+01
GOTERM_CC_FAT	GO:0005759~mitochondrial matrix	4	4.82E-02	6.75E-01	4.17E+01
GOTERM_CC_FAT	GO:0031980~mitochondrial lumen	4	4.82E-02	6.75E-01	4.17E+01

Subnetwork 3

Category	Term	Count	PValue	Benjamini	FDR
GOTERM_BP_FAT	GO:0034627~de novo NAD biosynthetic pro	3	5.54E-05	6.52E-03	6.34E-02
GOTERM_BP_FAT	GO:0034354~de novo NAD biosynthetic pro	3	5.54E-05	6.52E-03	6.34E-02
SP_PIR_KEYWORDS	pyridine nucleotide biosynthesis	3	1.34E-04	5.88E-03	1.25E-01
GOTERM_BP_FAT	GO:0009110~vitamin biosynthetic process	4	3.62E-04	2.11E-02	4.13E-01
GOTERM_BP_FAT	GO:0042364~water-soluble vitamin biosynth	4	3.62E-04	2.11E-02	4.13E-01
GOTERM_BP_FAT	GO:0009435~NAD biosynthetic process	3	3.62E-04	1.42E-02	4.14E-01
GOTERM_BP_FAT	GO:0006767~water-soluble vitamin metabol	4	4.79E-04	1.40E-02	5.47E-01
GOTERM_BP_FAT	GO:0006766~vitamin metabolic process	4	5.70E-04	1.34E-02	6.51E-01
GOTERM_BP_FAT	GO:0019359~nicotinamide nucleotide biosyn	3	6.55E-04	1.28E-02	7.47E-01
GOTERM_BP_FAT	GO:0019363~pyridine nucleotide biosyntheti	3	8.33E-04	1.40E-02	9.49E-01
GOTERM_BP_FAT	GO:0019674~NAD metabolic process	3	1.62E-03	2.36E-02	1.84E+00
GOTERM_BP_FAT	GO:0044271~nitrogen compound biosynthet	5	5.74E-03	7.27E-02	6.38E+00
UP_SEQ_FEATURE	domain:DEP	2	6.04E-03	2.29E-01	5.48E+00
GOTERM_BP_FAT	GO:0046496~nicotinamide nucleotide metab	3	6.14E-03	7.01E-02	6.80E+00
GOTERM_BP_FAT	GO:0006769~nicotinamide metabolic proces	3	6.39E-03	6.64E-02	7.07E+00
GOTERM_BP_FAT	GO:0009820~alkaloid metabolic process	3	6.39E-03	6.64E-02	7.07E+00
SMART	SM00049:DEP	2	6.58E-03	3.25E-02	2.83E+00
GOTERM_BP_FAT	GO:0019362~pyridine nucleotide metabolic	3	6.89E-03	6.58E-02	7.61E+00
GOTERM_BP_FAT	GO:0019748~secondary metabolic process	3	9.10E-03	7.96E-02	9.93E+00
GOTERM_BP_FAT	GO:0006733~oxidoreduction coenzyme meta	3	1.00E-02	8.12E-02	1.09E+01
GOTERM_BP_FAT	GO:0043603~cellular amide metabolic proce	3	1.06E-02	8.06E-02	1.15E+01
GOTERM_BP_FAT	GO:0009108~coenzyme biosynthetic process	3	1.22E-02	8.69E-02	1.32E+01
INTERPRO	IPR000591:Pleckstrin/G-protein, interacting	2	1.28E-02	2.65E-01	9.80E+00
KEGG_PATHWAY	sce00380:Tryptophan metabolism	2	2.21E-02	4.38E-02	4.92E+00
GOTERM_BP_FAT	GO:0051188~cofactor biosynthetic process	3	2.73E-02	1.75E-01	2.72E+01
GOTERM_BP_FAT	GO:0009165~nucleotide biosynthetic process	3	3.43E-02	2.05E-01	3.30E+01
GOTERM_MF_FAT	GO:0030695~GTPase regulator activity	3	3.73E-02	8.56E-01	3.08E+01
GOTERM_BP_FAT	GO:0034654~nucleobase, nucleoside, nucleo	3	3.97E-02	2.23E-01	3.71E+01
GOTERM_BP_FAT	GO:0034404~nucleobase, nucleoside and nu	3	3.97E-02	2.23E-01	3.71E+01
GOTERM_BP_FAT	GO:0006414~translational elongation	4	4.70E-02	2.47E-01	4.23E+01

Subnetwork 4

Category	Term	Count	PValue	Benjamini	FDR
GOTERM_BP_FAT	GO:0009628~response to abiotic stimulus	5	1.79E-02	9.62E-01	2.00E+01
SP_PIR_KEYWORDS	cytoplasm	8	2.74E-02	8.21E-01	2.44E+01
GOTERM_MF_FAT	GO:0005199~structural constituent of cell w	2	3.53E-02	8.92E-01	3.04E+01
UP_SEQ_FEATURE	compositionally biased region:Poly-Thr	2	4.56E-02	9.27E-01	3.69E+01

Subnetwork 5

Category	Term	Count	PValue	Benjamini	FDR
GOTERM_BP_FAT	GO:0034605~cellular response to heat	5	2.61E-05	1.31E-03	2.52E-02
GOTERM_BP_FAT	GO:0009408~response to heat	5	4.12E-05	1.03E-03	3.97E-02
GOTERM_BP_FAT	GO:0009266~response to temperature stimul	5	5.36E-05	8.93E-04	5.16E-02
GOTERM_BP_FAT	GO:0007039~vacuolar protein catabolic proc	4	2.56E-04	3.20E-03	2.47E-01
GOTERM_BP_FAT	GO:0009628~response to abiotic stimulus	5	3.28E-04	3.27E-03	3.15E-01
GOTERM_BP_FAT	GO:0033554~cellular response to stress	5	2.17E-03	1.80E-02	2.08E+00
GOTERM_BP_FAT	GO:0019323~pentose catabolic process	2	6.15E-03	4.31E-02	5.77E+00
GOTERM_BP_FAT	GO:0044257~cellular protein catabolic proce	4	6.30E-03	3.87E-02	5.91E+00
GOTERM_BP_FAT	GO:0030163~protein catabolic process	4	6.98E-03	3.82E-02	6.53E+00
GOTERM_BP_FAT	GO:0042732~D-xylose metabolic process	2	7.37E-03	3.63E-02	6.88E+00
KEGG_PATHWAY	sce00040:Pentose and glucuronate interconv	2	9.71E-03	6.60E-02	4.91E+00
GOTERM_BP_FAT	GO:0044265~cellular macromolecule catabo	4	1.47E-02	6.52E-02	1.33E+01
GOTERM_BP_FAT	GO:0009057~macromolecule catabolic proce	4	1.66E-02	6.74E-02	1.49E+01
GOTERM_BP_FAT	GO:0019321~pentose metabolic process	2	2.32E-02	8.63E-02	2.02E+01

Subnetwork 6

Category	Term	Count	PValue	Benjamini	FDR
GOTERM_CC_FAT	GO:0005838~proteasome regulatory particle	4	4.32E-06	1.95E-04	4.07E-03
GOTERM_CC_FAT	GO:0022624~proteasome accessory complex	4	4.32E-06	1.95E-04	4.07E-03
GOTERM_CC_FAT	GO:0034515~proteasome storage granule	4	8.64E-06	1.94E-04	8.12E-03
GOTERM_CC_FAT	GO:0031597~cytosolic proteasome complex	4	8.64E-06	1.94E-04	8.12E-03
SP_PIR_KEYWORDS	proteasome	4	1.12E-05	4.15E-04	1.01E-02
GOTERM_CC_FAT	GO:0000502~proteasome complex	4	4.12E-05	6.17E-04	3.87E-02
KEGG_PATHWAY	sce03050:Proteasome	4	5.19E-05	1.56E-04	1.64E-02
GOTERM_CC_FAT	GO:0044445~cytosolic part	5	8.99E-05	1.01E-03	8.45E-02
GOTERM_BP_FAT	GO:0006511~ubiquitin-dependent protein ca	5	1.25E-04	1.37E-02	1.41E-01
GOTERM_CC_FAT	GO:0008541~proteasome regulatory particle	3	1.54E-04	1.38E-03	1.45E-01
GOTERM_BP_FAT	GO:0019941~modification-dependent protei	5	3.33E-04	1.82E-02	3.76E-01
GOTERM_BP_FAT	GO:0051603~proteolysis involved in cellular	5	3.85E-04	1.40E-02	4.34E-01
GOTERM_BP_FAT	GO:0043632~modification-dependent macro	5	4.42E-04	1.21E-02	4.98E-01
INTERPRO	IPR011991:Winged helix repressor DNA-bin	3	5.67E-04	1.24E-02	4.43E-01
GOTERM_BP_FAT	GO:0044257~cellular protein catabolic proce	5	1.47E-03	3.19E-02	1.65E+00
GOTERM_BP_FAT	GO:0030163~protein catabolic process	5	1.69E-03	3.05E-02	1.89E+00
GOTERM_BP_FAT	GO:0006508~proteolysis	5	1.85E-03	2.87E-02	2.07E+00
GOTERM_CC_FAT	GO:0005829~cytosol	5	1.96E-03	1.46E-02	1.83E+00
GOTERM_BP_FAT	GO:0044265~cellular macromolecule catabo	5	4.54E-03	6.07E-02	5.02E+00
GOTERM_BP_FAT	GO:0009057~macromolecule catabolic proce	5	5.33E-03	6.32E-02	5.86E+00
COG_ONTOLOGY	Posttranslational modification, protein turno	4	7.76E-03	3.07E-02	2.93E+00
SP_PIR_KEYWORDS	protein degradation	2	1.48E-02	2.41E-01	1.25E+01
UP_SEQ_FEATURE	domain:PCI	2	1.48E-02	3.21E-01	1.15E+01
INTERPRO	IPR000717:Proteasome component region P	2	1.49E-02	1.52E-01	1.11E+01
GOTERM_CC_FAT	GO:0008540~proteasome regulatory particle	2	1.51E-02	9.34E-02	1.34E+01
SP_PIR_KEYWORDS	acetylation	3	1.63E-02	1.83E-01	1.37E+01
SMART	SM00088:PINT	2	1.97E-02	7.63E-02	7.30E+00
GOTERM_BP_FAT	GO:0034621~cellular macromolecular comp	4	2.75E-02	2.64E-01	2.70E+01
GOTERM_BP_FAT	GO:0043933~macromolecular complex subu	4	4.48E-02	3.68E-01	4.04E+01

Subnetwork 7

Category	Term	Count	PValue	Benjamini	FDR
GOTERM_BP_FAT	GO:0015031~protein transport	4	9.29E-03	3.55E-01	8.48E+00
GOTERM_BP_FAT	GO:0045184~establishment of protein locali	4	1.14E-02	2.37E-01	1.03E+01
GOTERM_BP_FAT	GO:0008104~protein localization	4	1.51E-02	2.13E-01	1.35E+01

Subnetwork 8

Category	Term	Count	PValue	Benjamini	FDR
SP_PIR_KEYWORDS	sterol biosynthesis	2	2.03E-02	4.60E-01	1.60E+01
SP_PIR_KEYWORDS	oxidoreductase	3	2.10E-02	2.73E-01	1.66E+01
SP_PIR_KEYWORDS	Steroid biosynthesis	2	2.30E-02	2.08E-01	1.80E+01
GOTERM_BP_FAT	GO:0016129~phytosteroid biosynthetic proce	2	2.92E-02	9.83E-01	2.94E+01
GOTERM_BP_FAT	GO:0006696~ergosterol biosynthetic process	2	2.92E-02	9.83E-01	2.94E+01
KEGG_PATHWAY	sce00100:Steroid biosynthesis	2	3.10E-02	9.01E-02	9.43E+00
GOTERM_BP_FAT	GO:0008204~ergosterol metabolic process	2	3.16E-02	8.89E-01	3.15E+01
GOTERM_BP_FAT	GO:0016128~phytosteroid metabolic process	2	3.16E-02	8.89E-01	3.15E+01
GOTERM_BP_FAT	GO:0016126~sterol biosynthetic process	2	4.12E-02	8.53E-01	3.90E+01
GOTERM_BP_FAT	GO:0006694~steroid biosynthetic process	2	4.12E-02	8.53E-01	3.90E+01
SP_PIR_KEYWORDS	lipid synthesis	2	4.39E-02	2.86E-01	3.18E+01
GOTERM_CC_FAT	GO:0005783~endoplasmic reticulum	3	4.88E-02	8.84E-01	3.72E+01

Table S5: Results of YeastMine analysis of subnetwork 1.

GO Term	p-Value	Matches	Associated ORFs	GO Term ID
organic acid biosynthetic process [GO:0016053]	3.85E-08	11	YBR249C, YCL009C, YCL018W, YDR034C, YEL071W, YFL018C, YGR012W, YHR063C, YKL184W, YMR062C, YOR196C	GO:0016053
carboxylic acid biosynthetic process [GO:0046394]	3.85E-08	11	YBR249C, YCL009C, YCL018W, YDR034C, YEL071W, YFL018C, YGR012W, YHR063C, YKL184W, YMR062C, YOR196C	GO:0046394
single-organism biosynthetic process [GO:0044711]	4.17E-07	17	YDR034C, YDR531W, YEL071W, YER026C, YFL018C, YGR012W, YHR063C, YJR073C, YKL184W, YMR062C, YOR142W, YOR196C, YPL031C	GO:0044711
small molecule biosynthetic process [GO:0044283]	7.54E-07	12	YBR239C, YBR249C, YCL009C, YCL018W, YDR034C, YEL071W, YFL018C, YGR012W, YHR063C, YKL184W, YMR062C, YOR196C	GO:0044283
carboxylic acid metabolic process [GO:0019752]	1.90E-06	13	YBR249C, YCL009C, YCL018W, YDR034C, YDR178W, YEL071W, YFL018C, YGR012W, YHR063C, YKL184W, YMR062C, YOR142W, YOR196C	GO:0019752
oxoacid metabolic process [GO:0043436]	2.90E-06	13	YBR249C, YCL009C, YCL018W, YDR034C, YDR178W, YEL071W, YFL018C, YGR012W, YHR063C, YKL184W, YMR062C, YOR142W, YOR196C	GO:0043436
organic acid metabolic process [GO:0006082]	2.98E-06	13	YBR249C, YCL009C, YCL018W, YDR034C, YDR178W, YEL071W, YFL018C, YGR012W, YHR063C, YKL184W, YMR062C, YOR142W, YOR196C	GO:0006082
small molecule metabolic process [GO:0044281]	3.41E-06	17	YBR239C, YBR249C, YCL009C, YCL018W, YDR034C, YDR178W, YDR531W, YEL071W, YFL018C, YGR012W, YHR043C, YHR063C, YKL184W, YMR062C, YOR142W, YOR196C, YPL031C	GO:0044281
single-organism metabolic process [GO:0044710]	1.64E-05	22	YBL030C, YBR239C, YBR249C, YCL009C, YCL018W, YDR034C, YDR178W, YDR531W, YEL071W, YER026C, YFL018C, YGL257C, YGR012W, YGR207C, YHR043C, YHR063C, YJR073C, YKL184W, YMR062C,	GO:0044710
cellular amino acid biosynthetic process [GO:0008652]	1.91E-05	7	YBR249C, YCL009C, YCL018W, YDR034C, YFL018C, YGR012W, YMR062C	GO:0008652
alpha-amino acid metabolic process [GO:1901605]	2.26E-05	8	YCL009C, YCL018W, YDR034C, YFL018C, YGR012W, YHR063C, YKL184W, YMR062C	GO:1901605
branched-chain amino acid metabolic process [GO:0009081]	2.73E-05	4	YCL009C, YCL018W, YFL018C, YHR063C	GO:0009081
cellular amino acid metabolic process [GO:0006520]	2.77E-05	9	YBR249C, YCL009C, YCL018W, YDR034C, YFL018C, YGR012W, YHR063C, YKL184W, YMR062C	GO:0006520
valine metabolic process [GO:0006573]	3.98E-05	3	YCL009C, YFL018C, YHR063C	GO:0006573

alpha-amino acid biosynthetic process [GO:1901607]	1.35E-04	6	YCL009C, YCL018W, YDR034C, YFL018C, YGR012W, YMR062C	GO:1901607
cellular modified amino acid biosynthetic process [GO:0042398]	6.57E-04	3	YER026C, YHR063C, YKL184W	GO:0042398
monocarboxylic acid biosynthetic process [GO:0072330]	9.53E-04	4	YEL071W, YHR063C, YKL184W, YOR196C	GO:0072330
monocarboxylic acid metabolic process [GO:0032787]	1.26E-03	6	YCL018W, YEL071W, YFL018C, YHR063C, YKL184W, YOR196C	GO:0032787
ornithine metabolic process [GO:0006591]	1.43E-03	2	YKL184W, YMR062C	GO:0006591
L-serine metabolic process [GO:0006563]	1.78E-03	2	YFL018C, YGR012W	GO:0006563
pantothenate metabolic process [GO:0015939]	1.78E-03	2	YHR063C, YKL184W	GO:0015939
pantothenate biosynthetic process [GO:0015940]	1.78E-03	2	YHR063C, YKL184W	GO:0015940

Table S6: Gene expression values of RM and BY strains for genes in the phosphocholine network and statistical analysis.

Gene	MHO1	OPT1	ITR1
Nonsynonymous SNPs	A331T; F164I	A200V; V439I	C521F
SNPs in promoter region	4	4	2
(-1 to -500)	(-141; -169; -224; -285)	(-108; -142; -143; -333)	(-211, -286)
*RM Average	-0.1542	0.0317	0.0342
Fold Change(RM/BY)	1.1128	0.9783	0.9766
#Expresssion difference (RM-BY)	-0.2708	0.1200	0.1108
p-value	0.0019	0.3730	0.0782
95% CI (Expression Diff)	[-0.4260,-0.1157]	[-0.1574,0.3974]	[-0.0140,0.2357]
95% CI (Fold Change)	[0.7443,0.9229]	[-0.1574,0.3974]	[0.9903,1.1775]

Gene	OPI3	CHO1
Nonsynonymous SNPs	None	A9T; L234F
SNPs in promoter region	4	6
(-1 to -500)	(-1; -389; -395; -450)	(-78; -79; -213; -228; -375;-451)
*RM Average	-0.5958	-0.3100
Fold Change(RM/BY)	1.5113	1.2397
#Expresssion difference (RM-BY)	-0.4558	-0.1767
p-value	0.0078	0.0219
95% CI (Expression Diff)	[-0.7735,-0.1381]	[-0.3241,-0.0292]
95% CI (Fold Change)	[0.5850,0.9087]	[0.7988,0.9800]

* Average expression difference from 12 RM parent strains compared to a pool of BY strains - see "RM BY expression values" tab

Average expression difference from 12 RM parent strains compared to a pool of BY strains and 6 BY strains compared to the same pool of BY strains

Table S7: Identification of transcription factors controlling subnetworks using YEASTRACT.

Subnetwork 1

Transcription Factor	% in user set	% in Yeastract	p-value	Target ORF/Genes
Opi1p	21.62%	2.09%	1.07E-04	PET9 SDH4 ITR1 CHO1 MCY1 OPT1 MHO1 OPI3
Cup9p	37.84%	1.29%	1.74E-04	PET9 ILV6 LEU2 FCF1 ITR1 CAB1 DLD3 CHO1 AIM13 CIR1 PAN5 RPB4 THI7 PHO85
Pho4p	40.54%	1.06%	9.32E-04	ILV6 LEU2 FCF1 ITR1 CHO1 AIM13 DOG2 PAN5 YIL165C MHO1 OPI3 MOG1 ARG7 LIP5 PHO85
Phd1p	27.03%	1.31%	1.24E-03	PET9 ARO4 LEU2 LYS14 DLD3 CHO1 LPD1 RPB4 LSC1 PHO85
Met32p	21.62%	1.45%	1.58E-03	ARO4 DLD3 DOG2 YIL165C OPT1 MHO1 THI7 ARG7
Thi2p	27.03%	1.25%	1.79E-03	PET9 ILV6 LEU2 LYS14 SDH4 FCF1 ITR1 CAB1 AIM13 THI7
Xbp1p	37.84%	1.00%	2.84E-03	PET9 SDH4 DLD3 LPD1 CIR1 DOG2 PAN5 YIL165C MOG1 SPE1 ARG7 YOR131C LSC1 LIP5
Stp1p	24.32%	1.25%	2.92E-03	LEU2 LYS14 CAB1 MCY1 PAN5 OPT1 MHO1 THI7 ARG7
Aca1p	13.51%	1.75%	3.15E-03	PET9 ILV6 LEU2 CHO1 PHO85
Stp2p	21.62%	1.24%	4.66E-03	ERT1 LEU2 LYS14 MCY1 OPT1 MHO1 THI7 YOR131C
Put3p	21.62%	1.21%	5.43E-03	PET9 ILV6 LEU2 SDH4 CHO1 LPD1 AIM13 OPI3
Swi4p	29.73%	1.03%	5.98E-03	PET9 ERT1 NHP10 SDH4 FCF1 SBE2 MCY1 PAN5 THI7 PDP3 ARG7
Met28p	10.81%	1.74%	6.26E-03	MCY1 YIL165C OPT1 PHO85
Ace2p	83.78%	0.65%	6.30E-03	PET9 ARO4 ILV6 LEU2 NHP10 LYS14 SDH4 FCF1 SBE2 ITR1 CAB1 DLD3 CHO1 LPD1 AIM13 RSC8 MNT2 MCY1 YHI9 DOG2 PAN5 OPT1 MHO1 MOG1 SPE1 THI7 PDP3 ARG7 YOR131C LIP5 PHO85
Yap3p	13.51%	1.49%	6.94E-03	ILV6 LEU2 SDH4 CHO1 LIP5
Hap5p	16.22%	1.33%	7.48E-03	PET9 LEU2 LYS14 SDH4 CHO1 LPD1
Pdc2p	2.70%	3.85%	7.87E-03	THI7
Gcn4p	59.46%	0.73%	1.04E-02	ARO4 ILV6 LEU2 LYS14 SDH4 SBE2 ITR1 CAB1 DLD3 LPD1 CIR1 YHI9 DOG2 YIL165C OPT1 MHO1 OPI3 SPE1 THI7 ARG7 LIP5 PHO85
Wtm2p	8.11%	1.78%	1.09E-02	MCY1 MOG1 PDP3
Rgt1p	16.22%	1.20%	1.29E-02	ILV6 LEU2 CHO1 LPD1 RSC8 PHO85
Gln3p	29.73%	0.93%	1.37E-02	ILV6 LEU2 ITR1 CHO1 AIM13 RSC8 YHI9 DOG2 YIL165C OPT1 MOG1
Mig3p	37.84%	0.84%	1.50E-02	PET9 ILV6 FCF1 ITR1 DLD3 CHO1 MCY1 YHI9 DOG2 RPB4 OPT1 SPE1 THI7 LSC1

Hot1p	5.41%	1.96%	1.55E-02	LEU2 MHO1
Gal4p	29.73%	0.90%	1.64E-02	PET9 ARO4 ILV6 LEU2 FCF1 CHO1 AIM13 CIR1 RPB4 THI7 PHO85
Leu3p	18.92%	1.05%	1.87E-02	PET9 ARO4 ILV6 LEU2 PAN5 SPE1 ARG7
Tye7p	29.73%	0.89%	1.88E-02	ILV6 LEU2 FCF1 CAB1 AIM13 MNT2 CIR1 RPB4 SPE1 THI7 PHO85
Hap3p	10.81%	1.31%	1.99E-02	PET9 LEU2 SDH4 LPD1
Yrm1p	51.35%	0.73%	2.19E-02	ILV6 LYS14 SDH4 SBE2 ITR1 CAB1 DLD3 LPD1 AIM13 RSC8 CIR1 YHI9 PAN5 YIL165C RPB4 MOG1 SPE1 THI7 LSC1
Abf1p	54.05%	0.71%	2.46E-02	PET9 ERT1 ARO4 LEU2 LYS14 SDH4 FCF1 SBE2 CHO1 LPD1 AIM13 MNT2 YHI9 DOG2 PAN5 OPT1 SPE1 THI7 ARG7 PHO85
Tup1p	51.35%	0.71%	2.79E-02	PET9 ERT1 ARO4 ILV6 LYS14 SDH4 FCF1 SBE2 DLD3 LPD1 AIM13 MCY1 YHI9 PAN5 RPB4 OPT1 THI7 LIP5 PHO85
Kar4p	21.62%	0.93%	2.85E-02	ILV6 LEU2 DLD3 CHO1 LPD1 OPT1 THI7 PHO85
Gat1p	8.11%	1.32%	2.90E-02	YHI9 OPT1 MHO1
Ert1p	8.11%	1.31%	2.98E-02	PET9 ERT1 MCY1
YNR063W	2.70%	1.85%	3.16E-02	YOR131C
Rim101p	37.84%	0.75%	3.75E-02	ARO4 NHP10 SBE2 DLD3 CHO1 AIM13 RSC8 MCY1 CIR1 YHI9 YIL165C RPB4 THI7 ARG7
YKL222C	2.70%	1.67%	3.84E-02	MHO1
Dal81p	10.81%	1.08%	4.03E-02	LEU2 DLD3 MCY1 OPT1
Zap1p	32.43%	0.78%	4.04E-02	ILV6 LEU2 NHP10 DLD3 MNT2 DOG2 OPT1 MHO1 OPI3 THI7 ARG7 LIP5
Met4p	32.43%	0.77%	4.24E-02	LYS14 SBE2 ITR1 DLD3 CHO1 DOG2 YIL165C OPT1 MHO1 OPI3 MOG1 THI7
Sfp1p	72.97%	0.62%	4.26E-02	PET9 ARO4 ILV6 LEU2 SDH4 FCF1 SBE2 ITR1 CAB1 DLD3 CHO1 AIM13 RSC8 CIR1 YHI9 DOG2 PAN5 YIL165C RPB4 OPT1 MHO1 MOG1 THI7 PDP3 ARG7 LSC1 LIP5
Pho2p	24.32%	0.84%	4.26E-02	SDH4 ITR1 DLD3 RSC8 CIR1 PAN5 YIL165C OPI3 THI7
Ixr1p	35.14%	0.75%	4.61E-02	PET9 ARO4 ILV6 FCF1 DLD3 CHO1 LPD1 AIM13 CIR1 YHI9 THI7 PDP3 ARG7
Sok2p	43.24%	0.71%	4.76E-02	PET9 ERT1 ARO4 ILV6 LEU2 SDH4 FCF1 DLD3 AIM13 PAN5 YIL165C OPT1 MHO1 OPI3 THI7 ARG7
Dot6p	5.41%	1.26%	4.86E-02	FCF1 MCY1
Ino2p	16.22%	0.91%	5.04E-02	ITR1 CHO1 RPB4 MHO1 OPI3 ARG7
Rap1p	54.05%	0.67%	5.08E-02	PET9 ARO4 ILV6 LEU2 SBE2 ITR1 DLD3 CHO1 LPD1 MCY1 CIR1 YHI9 OPT1 OPI3 MOG1 SPE1 THI7 PDP3 LSC1 LIP5
Bas1p	54.05%	0.67%	5.10E-02	PET9 ERT1 LEU2 LYS14 SDH4 ITR1 DLD3 LPD1 AIM13 MCY1 CIR1 YHI9 OPT1 MHO1 OPI3 SPE1 THI7 PDP3 ARG7 LIP5
Arg80p	10.81%	1.01%	5.19E-02	SDH4 SBE2 MOG1 PDP3
YPR196W	5.41%	1.20%	5.48E-02	FCF1 MCY1

Rtg3p	16.22%	0.89%	5.54E-02	FCF1 DLD3 MCY1 PAN5 MOG1 ARG7
Rpn4p	35.14%	0.73%	5.81E-02	PET9 ARO4 ILV6 SDH4 CAB1 DLD3 YHI9 YIL165C OPI3 ARG7 YOR131C LSC1 PHO85
Ino4p	29.73%	0.75%	6.08E-02	LEU2 LYS14 ITR1 CHO1 AIM13 RPB4 MHO1 OPI3 MOG1 SPE1 THI7
Ume6p	29.73%	0.75%	6.30E-02	PET9 DLD3 CHO1 CIR1 YHI9 PAN5 YIL165C OPT1 OPI3 PDP3 ARG7
Ste12p	64.86%	0.62%	6.65E-02	PET9 ERT1 ARO4 ILV6 LEU2 LYS14 FCF1 SBE2 CAB1 CHO1 LPD1 AIM13 RSC8 MNT2 MCY1 YHI9 DOG2 YIL165C RPB4 THI7 PDP3 ARG7 YOR131C LSC1
Mga1p	16.22%	0.85%	6.67E-02	PET9 ARO4 LEU2 CAB1 DLD3 PAN5
Stb5p	24.32%	0.77%	6.73E-02	ILV6 LEU2 LYS14 CAB1 CHO1 LPD1 SPE1 LSC1 PHO85
YGR067C	5.41%	1.09%	6.83E-02	MCY1 PHO85
Tod6p	2.70%	1.19%	7.00E-02	MCY1
Sut2p	5.41%	1.08%	7.10E-02	MCY1 PHO85
Stb4p	2.70%	1.18%	7.15E-02	MCY1
Gal80p	2.70%	1.16%	7.29E-02	MCY1
Mig2p	8.11%	0.98%	7.30E-02	YHI9 DOG2 OPT1
Nrg1p	16.22%	0.83%	7.52E-02	PET9 FCF1 DLD3 AIM13 DOG2 PDP3
Ppr1p	5.41%	1.03%	7.84E-02	FCF1 MCY1
Upc2p	8.11%	0.94%	8.08E-02	ILV6 OPT1 THI7
Oaf1p	18.92%	0.78%	8.28E-02	PET9 FCF1 AIM13 MCY1 PAN5 ARG7 PHO85
Tos4p	5.41%	0.99%	8.61E-02	RSC8 PAN5
Rsc30p	2.70%	0.98%	9.77E-02	DOG2
Cha4p	5.41%	0.92%	1.01E-01	MCY1 PAN5
Nrg2p	8.11%	0.86%	1.03E-01	AIM13 DOG2 PDP3
Arg81p	8.11%	0.86%	1.06E-01	DLD3 MHO1 YOR131C
Arr1p	29.73%	0.69%	1.07E-01	ARO4 LYS14 SDH4 ITR1 DLD3 PAN5 OPT1 OPI3 MOG1 ARG7 PHO85
Cad1p	16.22%	0.75%	1.13E-01	DLD3 RSC8 YHI9 YIL165C PDP3 ARG7
Cat8p	5.41%	0.87%	1.15E-01	LEU2 DLD3
Gcr1p	29.73%	0.67%	1.20E-01	ARO4 ILV6 SDH4 DLD3 CHO1 LPD1 YHI9 RPB4 OPT1 MHO1 LIP5
Rdr1p	5.41%	0.85%	1.21E-01	FCF1 MCY1
Flo8p	18.92%	0.72%	1.25E-01	PET9 ERT1 LEU2 ITR1 CHO1 OPT1 ARG7
Hms1p	18.92%	0.70%	1.34E-01	ILV6 LEU2 FCF1 CHO1 AIM13 THI7 PHO85
Mot2p	2.70%	0.81%	1.35E-01	YIL165C
Ime1p	2.70%	0.81%	1.35E-01	RPB4
Sip4p	5.41%	0.81%	1.35E-01	MCY1 DOG2
Hap4p	18.92%	0.70%	1.36E-01	PET9 ERT1 SDH4 LPD1 ARG7 YOR131C LSC1
Yap7p	13.51%	0.72%	1.45E-01	ILV6 LEU2 YHI9 THI7 PHO85
Asg1p	5.41%	0.78%	1.48E-01	SDH4 FCF1
Sut1p	10.81%	0.73%	1.53E-01	LPD1 DOG2 MHO1 THI7
Usv1p	2.70%	0.75%	1.53E-01	ERT1
Eds1p	2.70%	0.74%	1.56E-01	FCF1
Met31p	8.11%	0.74%	1.59E-01	DLD3 YIL165C OPT1
YPR015C	2.70%	0.72%	1.60E-01	CHO1

Rds2p	10.81%	0.71%	1.63E-01	PET9 ILV6 LEU2 SDH4
Hal9p	5.41%	0.73%	1.67E-01	PET9 MHO1
Oaf3p	8.11%	0.71%	1.71E-01	PET9 MCY1 PHO85
YLR278C	8.11%	0.71%	1.76E-01	FCF1 MCY1 THI7
Sfl1p	2.70%	0.68%	1.79E-01	ARG7
Rox1p	16.22%	0.66%	1.80E-01	PET9 ARO4 LEU2 NHP10 ITR1 DLD3
Aro80p	8.11%	0.70%	1.80E-01	LEU2 MHO1 PHO85
Smp1p	5.41%	0.70%	1.84E-01	SBE2 MHO1
Swi5p	32.43%	0.62%	1.84E-01	PET9 ARO4 LEU2 ITR1 DLD3 CHO1 LPD1 OPT1 MHO1 SPE1 THI7 LSC1
Mot3p	10.81%	0.68%	1.86E-01	PET9 LEU2 CHO1 THI7
Rtg1p	5.41%	0.69%	1.89E-01	DLD3 LSC1
Yap1p	54.05%	0.58%	1.98E-01	PET9 ERT1 ARO4 ILV6 LYS14 SDH4 FCF1 ITR1 CAB1 DLD3 CHO1 YHI9 PAN5 YIL165C RPB4 MHO1 OPI3 MOG1 SPE1 ARG7
Mss11p	10.81%	0.66%	2.02E-01	LEU2 FCF1 PAN5 OPT1
Mig1p	8.11%	0.66%	2.06E-01	MCY1 DOG2 ARG7
Cst6p	43.24%	0.59%	2.14E-01	ARO4 ILV6 SDH4 FCF1 ITR1 CHO1 MCY1 YHI9 PAN5 RPB4 OPT1 MHO1 OPI3 THI7 LSC1 LIP5
Cin5p	35.14%	0.60%	2.14E-01	PET9 LEU2 LYS14 FCF1 ITR1 DLD3 AIM13 YHI9 PAN5 OPT1 MHO1 THI7 ARG7
Mac1p	8.11%	0.65%	2.19E-01	SDH4 YHI9 PHO85
Pip2p	21.62%	0.61%	2.25E-01	PET9 LEU2 FCF1 DLD3 AIM13 CIR1 RPB4 THI7
Rpi1p	2.70%	0.57%	2.28E-01	YIL165C
Uga3p	5.41%	0.63%	2.28E-01	FCF1 ARG7
Gcr2p	21.62%	0.61%	2.30E-01	ERT1 LYS14 SDH4 DLD3 OPT1 MHO1 SPE1 ARG7
Crz1p	10.81%	0.63%	2.34E-01	LEU2 NHP10 FCF1 OPI3
Plm2p	2.70%	0.54%	2.49E-01	PHO85
Ash1p	48.65%	0.56%	2.57E-01	PET9 ARO4 ILV6 LEU2 NHP10 LYS14 SDH4 SBE2 ITR1 LPD1 MCY1 CIR1 DOG2 PAN5 MOG1 THI7 ARG7 LIP5
Wtm1p	2.70%	0.53%	2.57E-01	ARG7
Azf1p	2.70%	0.51%	2.74E-01	PET9
Dal80p	2.70%	0.50%	2.80E-01	YHI9
Ifh1p	8.11%	0.58%	2.82E-01	DLD3 RSC8 YHI9
Fzf1p	2.70%	0.49%	2.86E-01	MCY1
Rlm1p	10.81%	0.56%	3.09E-01	ITR1 RSC8 MHO1 ARG7
Gat4p	2.70%	0.46%	3.13E-01	THI7
Tda9p	2.70%	0.45%	3.25E-01	MCY1
Gis1p	5.41%	0.51%	3.33E-01	ITR1 THI7
Rgm1p	5.41%	0.51%	3.38E-01	SDH4 AIM13
Cup2p	8.11%	0.52%	3.53E-01	SDH4 DLD3 MCY1
Tos8p	5.41%	0.49%	3.56E-01	CHO1 AIM13
Sef1p	2.70%	0.40%	3.78E-01	PDP3
Fkh1p	37.84%	0.53%	3.82E-01	PET9 LEU2 SDH4 DLD3 CHO1 RSC8 MNT2 DOG2 OPT1 MHO1 OPI3 THI7 PDP3 ARG7
Hac1p	8.11%	0.50%	3.84E-01	DOG2 OPT1 OPI3

Cbf1p	21.62%	0.52%	3.93E-01	ARO4 CAB1 LPD1 RSC8 YIL165C OPT1 MOG1 PDP3
Rsf2p	5.41%	0.46%	3.93E-01	PET9 YHI9
Sum1p	8.11%	0.48%	4.03E-01	MCY1 THI7 PHO85
Hsf1p	24.32%	0.52%	4.15E-01	ARO4 DLD3 MCY1 CIR1 YIL165C OPT1 MHO1 OPI3 ARG7
Dal82p	10.81%	0.49%	4.16E-01	LYS14 YIL165C OPI3 MOG1
Haa1p	8.11%	0.45%	4.62E-01	SDH4 MCY1 PDP3
Urc2p	2.70%	0.33%	4.69E-01	FCF1
Yox1p	18.92%	0.49%	4.69E-01	ILV6 FCF1 DLD3 AIM13 MNT2 OPI3 ARG7
Skn7p	13.51%	0.47%	4.76E-01	PET9 ILV6 LEU2 THI7 PHO85
Pdr1p	18.92%	0.49%	4.79E-01	DLD3 CHO1 RSC8 YHI9 ARG7 YOR131C LSC1
Yrr1p	16.22%	0.47%	5.10E-01	LEU2 FCF1 CAB1 LPD1 AIM13 MCY1
Yhp1p	16.22%	0.47%	5.13E-01	ILV6 FCF1 DLD3 MNT2 OPI3 ARG7
Aft2p	5.41%	0.38%	5.17E-01	MNT2 MCY1
Sko1p	10.81%	0.43%	5.35E-01	LEU2 FCF1 MNT2 MCY1
Mth1p	2.70%	0.29%	5.44E-01	MCY1
Pdr3p	13.51%	0.42%	5.88E-01	DLD3 RSC8 YHI9 PAN5 ARG7
Hap2p	18.92%	0.43%	6.22E-01	PET9 SDH4 SBE2 LPD1 THI7 PDP3 ARG7
Reb1p	10.81%	0.39%	6.23E-01	ARO4 MCY1 ARG7 LIP5
Adr1p	8.11%	0.36%	6.37E-01	PET9 YHI9 LSC1
Fkh2p	24.32%	0.44%	6.45E-01	PET9 ARO4 LEU2 FCF1 SBE2 MNT2 OPI3 MOG1 THI7
Rme1p	2.70%	0.24%	6.46E-01	THI7
Fhl1p	13.51%	0.40%	6.49E-01	LEU2 ITR1 DLD3 RSC8 YHI9
Mal33p	5.41%	0.31%	6.58E-01	MHO1 SPE1
Hcm1p	2.70%	0.23%	6.69E-01	PDP3
Yap5p	8.11%	0.34%	6.83E-01	MHO1 ARG7 PHO85
Rfx1p	5.41%	0.28%	7.22E-01	PET9 SPE1
Rph1p	5.41%	0.28%	7.28E-01	SDH4 MCY1
Tbf1p	2.70%	0.20%	7.49E-01	PDP3
Yap6p	8.11%	0.31%	7.65E-01	PET9 DLD3 MHO1
Ecm22p	2.70%	0.19%	7.75E-01	CAB1
Ndt80p	2.70%	0.17%	8.09E-01	THI7
Msn4p	27.03%	0.39%	8.33E-01	ARO4 LEU2 LYS14 CAB1 DLD3 DOG2 RPB4 MHO1 OPI3 THI7
Tec1p	43.24%	0.42%	8.63E-01	PET9 LYS14 SDH4 SBE2 ITR1 CHO1 MNT2 PAN5 YIL165C OPT1 OPI3 THI7 PDP3 ARG7 LSC1 PHO85
Spt23p	16.22%	0.33%	8.72E-01	DLD3 DOG2 MHO1 OPI3 MOG1 PHO85
Aft1p	10.81%	0.28%	8.80E-01	DLD3 CHO1 MCY1 ARG7
Mbp1p	2.70%	0.14%	8.90E-01	SPE1
Mcm1p	13.51%	0.30%	8.96E-01	ITR1 LPD1 YIL165C OPT1 ARG7
Msn2p	35.14%	0.38%	9.19E-01	LEU2 LYS14 FCF1 ITR1 CAB1 DLD3 AIM13 DOG2 RPB4 OPT1 MHO1 OPI3 THI7
Mga2p	5.41%	0.18%	9.42E-01	ARO4 PDP3

Subnetwork 2

Transcription Factor	% in user set	% in Yeastract	p-value	Target ORF/Genes
Plm2p	12.50%	1.62%	3.10E-03	RFT1 ROT2 IME4
Mcm1p	45.83%	0.66%	4.06E-03	SLA1 SCO1 SLM4 GRS1 PGI1 PRO3 MDJ1 PRM8 IME4 NUC1 TUM1
Ndd1p	8.33%	1.80%	5.85E-03	SCO1 SLM4
Ime1p	8.33%	1.61%	7.95E-03	GRS1 IME4
Ste12p	75.00%	0.47%	9.82E-03	SLA1 PEP1 NUP170 FLR1 SLM4 PGI1 PCS60 ROT2 IMG2 TIM9 PRO3 MDJ1 PRM8 IME4 SIP2 NUC1 MRPL20 MRPS8
Gcr1p	41.67%	0.61%	1.10E-02	SLA1 NUP170 SCO1 GRS1 PGI1 IMG2 PRO3 MDJ1 IME4 TUM1
Reb1p	29.17%	0.69%	1.47E-02	PCS60 ROT2 PRO3 MDJ1 PRM8 IME4 SIP2
Rtg1p	12.50%	1.03%	1.49E-02	SLA1 MDJ1 SIP2
Tye7p	33.33%	0.65%	1.51E-02	PEP1 FLR1 PGI1 PCS60 IMG2 MDJ1 DMA1 MRPL20
YKL222C	4.17%	1.67%	1.71E-02	NUP170
Abf1p	58.33%	0.50%	1.89E-02	SLA1 NUP170 FLR1 SLM4 GRS1 PGI1 PCS60 IMG2 PRO3 MDJ1 PRM8 IME4 DMA1 TUM1
Msn2p	66.67%	0.47%	1.96E-02	SLA1 PEP1 NUP170 FLR1 SCO1 SLM4 PGI1 IMG2 TIM9 FMP10 MDJ1 PRM8 SIP2 NUC1 MRPL20 MRPS8
Yap5p	25.00%	0.68%	2.17E-02	FLR1 SCO1 PGI1 FMP10 MDJ1 SIP2
Mal13p	4.17%	1.45%	2.22E-02	SCO1
YGR067C	8.33%	1.09%	2.26E-02	MDJ1 MRPS8
Wtm1p	8.33%	1.06%	2.46E-02	RFT1 FLR1
Tos4p	8.33%	0.99%	2.92E-02	PRO3 TUM1
Stb5p	29.17%	0.60%	3.13E-02	NUP170 FLR1 PGI1 PRO3 PRM8 DMA1 NUC1
Stb4p	4.17%	1.18%	3.27E-02	PCS60
Rap1p	58.33%	0.47%	3.50E-02	RFT1 NUP170 FLR1 SCO1 SLM4 GRS1 PGI1 ROT2 IMG2 TIM9 PRO3 MDJ1 SIP2 MRPL20
Cst6p	54.17%	0.48%	3.67E-02	RFT1 NUP170 GRS1 PGI1 ROT2 IMG2 PRO3 MDJ1 PRM8 DMA1 NUC1 MRPS8 TUM1
Cat8p	8.33%	0.87%	4.02E-02	PGI1 PRO3
Arg80p	12.50%	0.76%	4.12E-02	RFT1 PRM8 NUC1
Rgm1p	12.50%	0.76%	4.12E-02	NUP170 PRM8 NUC1
Sip4p	8.33%	0.81%	4.86E-02	PGI1 PRO3
Msn4p	50.00%	0.47%	5.24E-02	NUP170 FLR1 SCO1 GRS1 PGI1 IMG2 FMP10 MDJ1 PRM8 MRPL20 MRPS8 TUM1
Skn7p	25.00%	0.57%	5.40E-02	SLM4 GRS1 IMG2 MDJ1 SIP2 MRPS8
Mig1p	12.50%	0.66%	6.21E-02	GRS1 MDJ1 MRPL20
Put3p	16.67%	0.61%	6.45E-02	PGI1 PRO3 DMA1 MRPS8
Mac1p	12.50%	0.65%	6.71E-02	GRS1 PRM8 NUC1
Aca1p	8.33%	0.70%	6.88E-02	SIP2 MRPS8
Usv1p	4.17%	0.75%	7.39E-02	PCS60

Yap7p	16.67%	0.58%	7.72E-02	FLR1 PGI1 MDJ1 DMA1
Rgt1p	12.50%	0.60%	8.25E-02	PGI1 PRO3 MRPS8
Aft2p	12.50%	0.57%	9.44E-02	SLM4 MDJ1 DMA1
Ino4p	29.17%	0.48%	1.00E-01	SCO1 IMG2 MDJ1 SIP2 DMA1 MRPL20 MRPS8
Hms1p	20.83%	0.50%	1.06E-01	PEP1 PGI1 PCS60 MDJ1 SIP2
Wtm2p	4.17%	0.59%	1.10E-01	NUC1
Sfp1p	70.83%	0.39%	1.10E-01	SLA1 PEP1 RFT1 FLR1 SCO1 SLM4 GRS1 PGI1 PCS60 IMG2 PRO3 FMP10 PRM8 IME4 SIP2 NUC1 MRPL20
Rpi1p	4.17%	0.57%	1.15E-01	NUC1
Rds2p	12.50%	0.53%	1.15E-01	PGI1 MDJ1 DMA1
Ndt80p	12.50%	0.52%	1.21E-01	GRS1 DMA1 TUM1
Thi2p	16.67%	0.50%	1.23E-01	PGI1 IMG2 DMA1 TUM1
Yrm1p	45.83%	0.42%	1.24E-01	RFT1 FLR1 SCO1 IMG2 TIM9 MDJ1 IME4 SIP2 DMA1 NUC1 MRPS8
Sut2p	4.17%	0.54%	1.29E-01	MDJ1
Fkh1p	45.83%	0.42%	1.32E-01	NUP170 FLR1 SLM4 GRS1 PGI1 IMG2 TIM9 PRM8 IME4 DMA1 TUM1
Opi1p	8.33%	0.52%	1.35E-01	MDJ1 NUC1
Adr1p	16.67%	0.48%	1.38E-01	SLM4 PCS60 MDJ1 DMA1
Tup1p	45.83%	0.41%	1.42E-01	SLA1 RFT1 FLR1 GRS1 PGI1 TIM9 PRO3 MDJ1 IME4 DMA1 NUC1
Arr1p	29.17%	0.44%	1.51E-01	SCO1 GRS1 PCS60 ROT2 MDJ1 SIP2 TUM1
Gcn4p	50.00%	0.40%	1.58E-01	RFT1 FLR1 SCO1 SLM4 IMG2 TIM9 MDJ1 PRM8 IME4 NUC1 MRPL20 MRPS8
Crz1p	12.50%	0.47%	1.63E-01	SLM4 MDJ1 PRM8
Mal33p	12.50%	0.47%	1.63E-01	RFT1 SLM4 MRPL20
Aro80p	8.33%	0.47%	1.72E-01	PGI1 MRPS8
Gat1p	4.17%	0.44%	1.77E-01	RFT1
Ino2p	12.50%	0.45%	1.77E-01	SLA1 SCO1 MDJ1
Ert1p	4.17%	0.44%	1.79E-01	MDJ1
Gat3p	8.33%	0.45%	1.88E-01	GRS1 TIM9
Hap5p	8.33%	0.44%	1.90E-01	PGI1 MDJ1
Pdr3p	20.83%	0.42%	1.93E-01	SLA1 FLR1 SLM4 TIM9 NUC1
Gln3p	20.83%	0.42%	1.99E-01	GRS1 PGI1 SIP2 DMA1 MRPS8
Sok2p	37.50%	0.40%	1.99E-01	SLM4 GRS1 TIM9 PRO3 FMP10 MDJ1 PRM8 IME4 SIP2
Sef1p	4.17%	0.40%	2.07E-01	GRS1
Ume6p	25.00%	0.41%	2.11E-01	PGI1 IMG2 TIM9 IME4 MRPL20 MRPS8
Gal4p	20.83%	0.41%	2.14E-01	PGI1 PCS60 IMG2 SIP2 DMA1
Asg1p	4.17%	0.39%	2.15E-01	NUC1
Rds1p	4.17%	0.38%	2.19E-01	IMG2
Fkh2p	33.33%	0.39%	2.25E-01	FLR1 SCO1 SLM4 GRS1 PGI1 IMG2 TIM9 NUC1
Ash1p	50.00%	0.38%	2.32E-01	PEP1 NUP170 SCO1 GRS1 PGI1 ROT2 PRO3 PRM8 IME4 SIP2 MRPL20 MRPS8
Rpn4p	29.17%	0.39%	2.35E-01	SLA1 PEP1 FLR1 GRS1 PGI1 PCS60 MDJ1
Tbf1p	8.33%	0.40%	2.40E-01	NUP170 SCO1

Msn1p	4.17%	0.35%	2.51E-01	MDJ1
Yrr1p	20.83%	0.39%	2.55E-01	FLR1 SCO1 MDJ1 IME4 SIP2
Spt23p	29.17%	0.38%	2.57E-01	PGI1 ROT2 MDJ1 PRM8 IME4 SIP2 DMA1
Pip2p	20.83%	0.38%	2.68E-01	PGI1 PCS60 IMG2 SIP2 DMA1
Urc2p	4.17%	0.33%	2.70E-01	SLM4
Ace2p	70.83%	0.36%	2.73E-01	SLA1 PEP1 NUP170 FLR1 SCO1 SLM4 GRS1 PGI1 PCS60 ROT2 IMG2 FMP10 MDJ1 SIP2 NUC1 MRPL20 MRPS8
Hap3p	4.17%	0.33%	2.75E-01	IME4
Sut1p	8.33%	0.36%	2.81E-01	PCS60 FMP10
Swi4p	16.67%	0.37%	2.86E-01	SLM4 MDJ1 PRM8 MRPS8
Dal82p	12.50%	0.37%	2.90E-01	NUP170 PRM8 TUM1
Uga3p	4.17%	0.31%	2.92E-01	PRM8
Yap3p	4.17%	0.30%	3.12E-01	MRPS8
Mot3p	8.33%	0.34%	3.18E-01	NUP170 PGI1
Nrg2p	4.17%	0.29%	3.27E-01	MRPS8
Hap1p	4.17%	0.29%	3.28E-01	PRM8
Arg81p	4.17%	0.29%	3.31E-01	IMG2
Mss11p	8.33%	0.33%	3.34E-01	NUP170 MDJ1
Yox1p	20.83%	0.35%	3.46E-01	SLM4 MDJ1 PRM8 SIP2 DMA1
Pdr1p	20.83%	0.35%	3.53E-01	FLR1 SLM4 TIM9 FMP10 NUC1
Rox1p	12.50%	0.33%	3.62E-01	SCO1 PRM8 TUM1
Sko1p	12.50%	0.32%	3.80E-01	FLR1 MDJ1 PRM8
Rtg3p	8.33%	0.30%	4.00E-01	NUP170 FLR1
Tos8p	4.17%	0.25%	4.03E-01	SLM4
Met31p	4.17%	0.25%	4.03E-01	IME4
Fhl1p	16.67%	0.32%	4.13E-01	PGI1 ROT2 TIM9 MDJ1
Flo8p	12.50%	0.31%	4.22E-01	PRO3 FMP10 MDJ1
Rsf2p	4.17%	0.23%	4.34E-01	MRPS8
Hcm1p	4.17%	0.23%	4.35E-01	PRO3
Rlm1p	8.33%	0.28%	4.36E-01	PCS60 PRM8
Rim101p	25.00%	0.32%	4.38E-01	PGI1 IMG2 MDJ1 MRPL20 MRPS8 TUM1
Nrg1p	8.33%	0.28%	4.46E-01	TIM9 MRPS8
Cin5p	29.17%	0.32%	4.60E-01	FLR1 PGI1 PCS60 PRM8 SIP2 DMA1 NUC1
Gcr2p	16.67%	0.30%	4.61E-01	PGI1 PCS60 PRM8 NUC1
Mig3p	20.83%	0.30%	5.05E-01	FLR1 PGI1 ROT2 PRO3 MDJ1
Cup9p	12.50%	0.28%	5.09E-01	PGI1 PCS60 DMA1
Cad1p	8.33%	0.25%	5.13E-01	FLR1 MDJ1
Mga2p	12.50%	0.27%	5.25E-01	PGI1 MDJ1 PRM8
Aft1p	16.67%	0.28%	5.26E-01	PGI1 PCS60 MDJ1 IME4
Pho4p	16.67%	0.28%	5.28E-01	TIM9 IME4 DMA1 MRPL20
Ecm22p	4.17%	0.19%	5.44E-01	GRS1
Met32p	4.17%	0.18%	5.62E-01	PCS60
Oaf1p	8.33%	0.22%	5.92E-01	PCS60 MDJ1
Sum1p	4.17%	0.16%	6.30E-01	PGI1
Yhp1p	12.50%	0.23%	6.53E-01	MDJ1 PRM8 DMA1
Yap6p	8.33%	0.20%	6.57E-01	SLM4 MDJ1

Hap2p	16.67%	0.25%	6.61E-01	SLM4 GRS1 IMG2 MRPL20
Leu3p	4.17%	0.15%	6.70E-01	MDJ1
Hap4p	8.33%	0.20%	6.73E-01	PRO3 IME4
Haa1p	4.17%	0.15%	6.73E-01	GRS1
Swi5p	20.83%	0.26%	6.74E-01	GRS1 PGI1 NUC1 MRPL20 MRPS8
Yap1p	41.67%	0.29%	6.77E-01	SLA1 RFT1 NUP170 FLR1 SCO1 GRS1 ROT2 MDJ1 PRM8 TUM1
Mbp1p	4.17%	0.14%	6.96E-01	SLM4
Mga1p	4.17%	0.14%	7.01E-01	PRM8
Tec1p	45.83%	0.29%	7.11E-01	PEP1 RFT1 NUP170 SLM4 MDJ1 PRM8 IME4 SIP2 DMA1 NUC1 MRPL20
Pho2p	8.33%	0.19%	7.24E-01	PGI1 TIM9
Xbp1p	12.50%	0.21%	7.25E-01	PEP1 GRS1 PRM8
Ixr1p	16.67%	0.23%	7.28E-01	FLR1 IME4 DMA1 TUM1
Hsf1p	16.67%	0.23%	7.31E-01	SCO1 PGI1 MDJ1 PRM8
Phd1p	4.17%	0.13%	7.45E-01	PGI1
Cbf1p	12.50%	0.20%	7.87E-01	PCS60 PRM8 SIP2
Kar4p	4.17%	0.12%	8.05E-01	IME4
Zap1p	8.33%	0.13%	9.17E-01	PGI1 PRM8
Bas1p	25.00%	0.20%	9.35E-01	GRS1 PGI1 PCS60 FMP10 MDJ1 PRM8

Subnetwork 3

Transcription Factor	% in user set	% in Yeastract	p-value	Target ORF/Genes
Mig1p	37.50%	1.32%	2.75E-05	TIP1 RTC2 ICS2 RGD2 RPI1 BNA1
Ste12p	93.75%	0.39%	5.18E-05	TIP1 RTC2 ICS2 NUR1 MKC7 RGD2 ATE1 HOS4 RPI1 BNA1 BNA5 SST2 YML096W MED11 ECM3
Mot3p	37.50%	1.02%	1.49E-04	TIP1 ICS2 RPI1 BNA1 SST2 ECM3
Stp2p	37.50%	0.93%	2.62E-04	TIP1 ICS2 RPI1 BNA5 SST2 ECM3
Nrg1p	37.50%	0.83%	5.42E-04	TIP1 RTC2 ICS2 NUR1 RPI1 MED11
Gcr2p	50.00%	0.61%	7.81E-04	TIP1 ICS2 BNA6 RPI1 BNA1 BNA5 SST2 ECM3
Mga2p	43.75%	0.63%	1.32E-03	TIP1 RTC2 RPI1 BNA1 BNA5 SST2 ECM3
Tbs1p	12.50%	1.96%	1.39E-03	TIP1 HOS4
Met28p	18.75%	1.30%	1.41E-03	NUR1 MKC7 MED11
Hms2p	12.50%	1.74%	1.96E-03	TIP1 RPI1
Hcm1p	25.00%	0.92%	2.05E-03	NUR1 HOS4 RPI1 BNA5
Gln3p	43.75%	0.59%	2.11E-03	TIP1 RTC2 ICS2 MKC7 RPI1 BNA1 SST2
Sko1p	37.50%	0.65%	2.39E-03	TIP1 ICS2 RGD2 RPI1 BNA1 SST2
Sok2p	62.50%	0.44%	2.51E-03	TIP1 RTC2 ICS2 BNA6 HOS4 RPI1 BNA1 SST2 YML096W ECM3
Ash1p	75.00%	0.38%	3.18E-03	TIP1 ICS2 NUR1 MKC7 BNA6 HOS4 RPI1 BNA1 BNA5 SST2 MED11 ECM3
Msn1p	18.75%	1.05%	3.18E-03	TIP1 RPI1 ECM3
Yap6p	37.50%	0.61%	3.26E-03	TIP1 ICS2 MKC7 BNA6 RPI1 SST2
Ifh1p	25.00%	0.77%	4.49E-03	TIP1 ICS2 RPI1 BNA1
Dot6p	12.50%	1.26%	4.92E-03	ICS2 BNA5
Ixr1p	50.00%	0.46%	6.05E-03	TIP1 ICS2 RGD2 BNA6 ATE1 RPI1 BNA5 YML096W
Nrg2p	18.75%	0.86%	6.29E-03	TIP1 RTC2 RPI1
Stp4p	12.50%	1.14%	6.53E-03	BNA5 ECM3
Msn2p	75.00%	0.35%	6.72E-03	TIP1 RTC2 ICS2 NUR1 MKC7 RGD2 ATE1 RPI1 BNA1 SST2 YML096W ECM3
Msn4p	62.50%	0.39%	7.85E-03	TIP1 RTC2 ICS2 NUR1 MKC7 BNA6 ATE1 RPI1 SST2 ECM3
Bas1p	68.75%	0.37%	7.94E-03	TIP1 RTC2 ICS2 MKC7 HOS4 RPI1 BNA1 BNA5 SST2 YML096W ECM3
Kar4p	31.25%	0.58%	8.34E-03	TIP1 RTC2 ICS2 NUR1 BNA1
Stb5p	37.50%	0.52%	8.84E-03	TIP1 ICS2 MKC7 RGD2 RPI1 YML096W
Pdr8p	6.25%	1.54%	9.04E-03	TIP1
Sum1p	25.00%	0.65%	9.49E-03	RTC2 BNA6 BNA1 BNA5
Rgm1p	18.75%	0.76%	9.99E-03	RPI1 SST2 ECM3
Rox1p	31.25%	0.55%	1.05E-02	TIP1 MKC7 HOS4 RPI1 ECM3
Crz1p	25.00%	0.63%	1.08E-02	RPI1 BNA5 MED11 ECM3
Oaf3p	18.75%	0.71%	1.22E-02	TIP1 ICS2 RPI1
Leu3p	25.00%	0.60%	1.28E-02	ICS2 RPI1 BNA1 BNA5
Gat1p	12.50%	0.88%	1.31E-02	NUR1 MKC7

Cat8p	12.50%	0.87%	1.34E-02	TIP1 NUR1
Ace2p	87.50%	0.29%	1.45E-02	TIP1 RTC2 ICS2 MKC7 RGD2 BNA6 HOS4 RPI1 BNA1 BNA5 SST2 YML096W MED11 ECM3
Tod6p	6.25%	1.19%	1.48E-02	ECM3
Yhp1p	37.50%	0.47%	1.55E-02	TIP1 BNA6 RPI1 BNA1 SST2 ECM3
Rlm1p	25.00%	0.56%	1.69E-02	TIP1 RTC2 ICS2 RPI1
Mac1p	18.75%	0.65%	1.73E-02	HOS4 BNA1 SST2
Mcm1p	43.75%	0.42%	1.85E-02	TIP1 ICS2 NUR1 BNA5 SST2 MED11 ECM3
Sfp1p	81.25%	0.30%	2.12E-02	TIP1 RTC2 ICS2 NUR1 MKC7 RGD2 BNA6 RPI1 BNA1 BNA5 SST2 YML096W ECM3
Rsc30p	6.25%	0.98%	2.13E-02	BNA1
Tec1p	75.00%	0.31%	2.14E-02	TIP1 RTC2 ICS2 NUR1 RGD2 ATE1 HOS4 RPI1 BNA1 BNA5 SST2 YML096W
Xbp1p	37.50%	0.43%	2.46E-02	TIP1 BNA6 RPI1 BNA5 SST2 ECM3
Ecm22p	18.75%	0.56%	2.71E-02	TIP1 ICS2 SST2
Gcn4p	62.50%	0.33%	2.82E-02	TIP1 RTC2 MKC7 RGD2 BNA6 RPI1 BNA1 BNA5 SST2 ECM3
Cin5p	50.00%	0.37%	2.89E-02	TIP1 RTC2 ICS2 RGD2 BNA6 RPI1 BNA1 MED11
Mig2p	12.50%	0.65%	2.91E-02	ICS2 RPI1
Usv1p	6.25%	0.75%	3.54E-02	HOS4
Cup2p	18.75%	0.52%	3.56E-02	RPI1 BNA1 SST2
Eds1p	6.25%	0.74%	3.64E-02	ICS2
YPR015C	6.25%	0.72%	3.73E-02	TIP1
Mth1p	12.50%	0.58%	3.99E-02	SST2 MED11
Hap1p	12.50%	0.57%	4.02E-02	TIP1 RTC2
Sfl1p	6.25%	0.68%	4.24E-02	MKC7
Fhl1p	31.25%	0.40%	4.65E-02	ICS2 RPI1 BNA1 SST2 ECM3
YPR196W	6.25%	0.60%	5.27E-02	ECM3
Abf1p	56.25%	0.32%	5.32E-02	TIP1 RTC2 ICS2 MKC7 RGD2 RPI1 BNA1 BNA5 SST2
Wtm2p	6.25%	0.59%	5.39E-02	ECM3
YJL206C	6.25%	0.58%	5.56E-02	NUR1
Rpi1p	6.25%	0.57%	5.68E-02	RPI1
Flo8p	25.00%	0.41%	5.71E-02	ICS2 BNA6 RPI1 BNA5
Rtg3p	18.75%	0.44%	5.77E-02	BNA1 BNA5 MED11
Hap4p	25.00%	0.40%	6.15E-02	ICS2 RGD2 RPI1 BNA1
Rme1p	12.50%	0.48%	6.30E-02	MKC7 ATE1
Plm2p	6.25%	0.54%	6.33E-02	NUR1
Mga1p	18.75%	0.43%	6.55E-02	RPI1 SST2 ECM3
Wtm1p	6.25%	0.53%	6.57E-02	MKC7
YLR278C	12.50%	0.47%	6.59E-02	TIP1 ECM3
Hsf1p	37.50%	0.35%	7.06E-02	TIP1 ICS2 HOS4 RPI1 SST2 MED11
Stp1p	18.75%	0.42%	7.07E-02	RPI1 BNA5 MED11
Azf1p	6.25%	0.51%	7.13E-02	MKC7
Tos4p	6.25%	0.50%	7.38E-02	TIP1
Fzf1p	6.25%	0.49%	7.51E-02	MED11

Hap5p	12.50%	0.44%	7.57E-02	TIP1 BNA1
Pho4p	31.25%	0.35%	7.74E-02	TIP1 RTC2 HOS4 SST2 ECM3
Swi4p	25.00%	0.37%	7.88E-02	TIP1 BNA1 BNA5 SST2
Rap1p	56.25%	0.30%	8.13E-02	TIP1 RTC2 ICS2 BNA6 RPI1 BNA1 BNA5 SST2 ECM3
Yox1p	31.25%	0.35%	8.20E-02	TIP1 MKC7 RGD2 RPI1 SST2
Cup9p	25.00%	0.37%	8.36E-02	TIP1 RPI1 MED11 ECM3
Cha4p	6.25%	0.46%	8.36E-02	MKC7
Phd1p	18.75%	0.39%	8.37E-02	TIP1 ICS2 RPI1
Gat4p	6.25%	0.46%	8.43E-02	TIP1
Tda9p	6.25%	0.45%	8.83E-02	TIP1
Fkh1p	50.00%	0.30%	9.11E-02	TIP1 RTC2 ICS2 RPI1 BNA1 SST2 MED11 ECM3
Rim101p	37.50%	0.32%	9.50E-02	TIP1 ICS2 BNA6 RPI1 SST2 ECM3
Cad1p	18.75%	0.38%	9.50E-02	RTC2 ICS2 RPI1
Rdr1p	6.25%	0.43%	9.58E-02	MED11
Rgt1p	12.50%	0.40%	9.65E-02	TIP1 BNA5
Tbf1p	12.50%	0.40%	9.97E-02	ICS2 RPI1
Sip4p	6.25%	0.40%	1.04E-01	TIP1
Aft2p	12.50%	0.38%	1.08E-01	NUR1 MED11
Zap1p	31.25%	0.32%	1.10E-01	ICS2 RPI1 BNA1 BNA5 SST2
Rds1p	6.25%	0.38%	1.14E-01	ECM3
Swi5p	37.50%	0.31%	1.17E-01	TIP1 RTC2 ICS2 MKC7 HOS4 RPI1
Hal9p	6.25%	0.37%	1.23E-01	MKC7
Rds2p	12.50%	0.36%	1.27E-01	TIP1 RPI1
Arr1p	31.25%	0.31%	1.28E-01	TIP1 BNA6 ATE1 HOS4 BNA1
Tye7p	25.00%	0.32%	1.29E-01	TIP1 RTC2 SST2 MED11
Oaf1p	18.75%	0.34%	1.30E-01	TIP1 ICS2 RPI1
Aca1p	6.25%	0.35%	1.32E-01	TIP1
Smp1p	6.25%	0.35%	1.33E-01	ICS2
Gzf3p	6.25%	0.35%	1.35E-01	RTC2
Urc2p	6.25%	0.33%	1.45E-01	BNA5
Mss11p	12.50%	0.33%	1.49E-01	TIP1 MED11
Mig3p	31.25%	0.30%	1.50E-01	TIP1 NUR1 BNA6 HOS4 MED11
Pip2p	25.00%	0.31%	1.54E-01	TIP1 ICS2 BNA1 MED11
Upc2p	6.25%	0.31%	1.58E-01	ICS2
Uga3p	6.25%	0.31%	1.59E-01	MED11
Mal33p	12.50%	0.31%	1.68E-01	MKC7 SST2
Yap3p	6.25%	0.30%	1.72E-01	TIP1
Put3p	12.50%	0.30%	1.79E-01	TIP1 RTC2
Reb1p	18.75%	0.29%	1.85E-01	RTC2 MKC7 HOS4
Rpn4p	31.25%	0.28%	1.91E-01	TIP1 RTC2 RGD2 BNA1 SST2
Yap1p	56.25%	0.26%	1.91E-01	TIP1 RTC2 MKC7 RGD2 RPI1 BNA1 SST2 YML096W ECM3
Yap7p	12.50%	0.29%	1.99E-01	TIP1 RPI1
Dal81p	6.25%	0.27%	1.99E-01	MED11
Mbp1p	12.50%	0.29%	2.01E-01	RTC2 MKC7

Skn7p	18.75%	0.28%	2.04E-01	ICS2 RPI1 SST2
Pho2p	18.75%	0.28%	2.12E-01	TIP1 MKC7 BNA1
Tup1p	43.75%	0.26%	2.13E-01	TIP1 ICS2 RGD2 BNA6 ATE1 RPI1 SST2
Ino4p	25.00%	0.27%	2.18E-01	TIP1 NUR1 RPI1 BNA5
Gis1p	6.25%	0.26%	2.19E-01	TIP1
Ume6p	25.00%	0.27%	2.21E-01	ICS2 HOS4 RPI1 BNA1
Arg80p	6.25%	0.25%	2.22E-01	SST2
Aro80p	6.25%	0.23%	2.50E-01	TIP1
Thi2p	12.50%	0.25%	2.63E-01	TIP1 MED11
Pdr3p	18.75%	0.25%	2.65E-01	RTC2 RPI1 YML096W
Adr1p	12.50%	0.24%	2.82E-01	TIP1 RPI1
Gal4p	18.75%	0.25%	2.85E-01	TIP1 MED11 ECM3
Gcr1p	25.00%	0.24%	2.96E-01	TIP1 RTC2 BNA1 BNA5
Sut1p	6.25%	0.18%	3.53E-01	TIP1
Met32p	6.25%	0.18%	3.53E-01	BNA1
Yrm1p	37.50%	0.23%	3.60E-01	TIP1 ICS2 RGD2 BNA5 MED11 ECM3
Ndt80p	6.25%	0.17%	3.71E-01	TIP1
Hms1p	12.50%	0.20%	3.90E-01	TIP1 MED11
Hac1p	6.25%	0.17%	3.98E-01	MED11
Pdr1p	18.75%	0.21%	4.09E-01	RTC2 ICS2 RPI1
Ino2p	6.25%	0.15%	4.44E-01	BNA5
Haa1p	6.25%	0.15%	4.51E-01	SST2
Cbf1p	18.75%	0.20%	4.57E-01	TIP1 RTC2 RPI1
Fkh2p	25.00%	0.20%	5.00E-01	TIP1 ICS2 RPI1 ECM3
Hap2p	18.75%	0.19%	5.06E-01	BNA1 SST2 MED11
Dal82p	6.25%	0.12%	5.59E-01	MKC7
Yap5p	6.25%	0.11%	6.02E-01	YML096W
Spt23p	18.75%	0.16%	6.18E-01	TIP1 RPI1 SST2
Cst6p	31.25%	0.18%	6.19E-01	TIP1 RPI1 BNA1 BNA5 SST2
Aft1p	12.50%	0.14%	6.38E-01	TIP1 HOS4
Met4p	12.50%	0.13%	7.08E-01	RTC2 BNA1
Yrr1p	6.25%	0.08%	8.12E-01	ECM3

Subnetwork 4

Transcription Factor	% in user set	% in Yeastract	p-value	Target ORF/Genes
Ppr1p	18.75%	1.55%	7.47E-04	EDC2 MTD1 YAP1
Rdr1p	18.75%	1.28%	1.53E-03	MTD1 YAP1 YPL071C
Sip4p	18.75%	1.21%	1.84E-03	EDC2 COX5b YAP1
Yap7p	31.25%	0.72%	2.83E-03	EDC2 CIS3 URA8 MLF3 KRE1
Yap6p	37.50%	0.61%	3.26E-03	EDC2 YHR033W CIS3 URA8 MTD1 YAP1
Stp1p	31.25%	0.69%	3.46E-03	AGP2 EDC2 CIS3 YAP1 KRE1
Sfp1p	87.50%	0.32%	3.92E-03	AGP2 ISC1 EDC2 YHR033W PIH1 COX5b CIS3 URA8 MTD1 YAP1 YNL046W KRE1 DSE3 YPL071C
Aft2p	25.00%	0.76%	4.60E-03	AGP2 EDC2 YHR033W YAP1
Cup9p	37.50%	0.55%	6.08E-03	AGP2 YHR033W CIS3 MLF3 KRE1 YPL071C
Pho4p	43.75%	0.50%	6.42E-03	AGP2 ISC1 EDC2 YHR033W CIS3 MLF3 KRE1
Mga2p	37.50%	0.54%	6.74E-03	AGP2 EDC2 MTD1 YNL046W MLF3 DSE3
Spt23p	50.00%	0.44%	8.81E-03	EDC2 CIS3 MTD1 YAP1 PPZ1 YNL046W MLF3 KRE1
Opi1p	18.75%	0.79%	8.81E-03	URA8 MTD1 DSE3
Stb5p	37.50%	0.52%	8.84E-03	CIS3 URA8 MTD1 YAP1 MLF3 KRE1
Azf1p	12.50%	1.01%	9.03E-03	CIS3 YAP1
Sum1p	25.00%	0.65%	9.49E-03	EDC2 CIS3 MLF3 KRE1
Gis1p	18.75%	0.77%	9.64E-03	YHR033W COX5b YNL046W
Ino2p	25.00%	0.61%	1.24E-02	PIH1 URA8 MTD1 DSE3
Tye7p	37.50%	0.48%	1.25E-02	AGP2 ISC1 CIS3 MLF3 KRE1 YPL071C
Fhl1p	37.50%	0.48%	1.30E-02	ISC1 EDC2 YHR033W COX5b CIS3 YPL071C
Ace2p	87.50%	0.29%	1.45E-02	AGP2 ISC1 EDC2 YHR033W PIH1 COX5b CIS3 URA8 MTD1 YAP1 PPZ1 YNL046W DSE3 YPL071C
Hap2p	43.75%	0.43%	1.49E-02	EDC2 PIH1 CIS3 YNL046W KRE1 DSE3 YPL071C
Stb4p	6.25%	1.18%	1.51E-02	YAP1
Gal80p	6.25%	1.16%	1.55E-02	YAP1
Gcr1p	43.75%	0.43%	1.61E-02	AGP2 ISC1 EDC2 CIS3 URA8 YAP1 KRE1
Nrg1p	25.00%	0.55%	1.79E-02	AGP2 EDC2 YHR033W YAP1
Rsc30p	6.25%	0.98%	2.13E-02	COX5b
Rgt1p	18.75%	0.60%	2.19E-02	YHR033W CIS3 KRE1
Phd1p	25.00%	0.52%	2.22E-02	EDC2 YHR033W CIS3 KRE1
Ste12p	75.00%	0.31%	2.34E-02	ISC1 YHR033W PIH1 CIS3 URA8 MTD1 YAP1 YNL046W MLF3 KRE1 DSE3 YPL071C

Pho2p	31.25%	0.47%	2.39E-02	EDC2 COX5b MTD1 YNL046W DSE3
Aca1p	12.50%	0.70%	2.40E-02	CIS3 KRE1
Ndd1p	6.25%	0.90%	2.50E-02	CIS3
Ecm22p	18.75%	0.56%	2.71E-02	EDC2 COX5b YPL071C
Rpn4p	43.75%	0.39%	2.73E-02	EDC2 COX5b CIS3 MTD1 YAP1 YNL046W YPL071C
Urc2p	12.50%	0.66%	2.79E-02	AGP2 MTD1
Cin5p	50.00%	0.37%	2.89E-02	EDC2 YHR033W CIS3 URA8 MTD1 MLF3 KRE1 YPL071C
Yap1p	68.75%	0.32%	2.90E-02	AGP2 EDC2 YHR033W PIH1 COX5b CIS3 URA8 MTD1 YAP1 YNL046W DSE3
Met32p	18.75%	0.55%	3.02E-02	YHR033W CIS3 YAP1
Sok2p	50.00%	0.35%	3.60E-02	AGP2 ISC1 EDC2 YHR033W CIS3 URA8 YAP1 KRE1
Yap3p	12.50%	0.60%	3.65E-02	CIS3 KRE1
YPR015C	6.25%	0.72%	3.73E-02	PPZ1
Mot3p	18.75%	0.51%	3.76E-02	COX5b YNL046W MLF3
Yap5p	25.00%	0.46%	3.79E-02	YHR033W CIS3 MTD1 YNL046W
Nrg2p	12.50%	0.58%	3.99E-02	AGP2 YHR033W
Mth1p	12.50%	0.58%	3.99E-02	MTD1 YAP1
Oaf1p	25.00%	0.45%	4.05E-02	CIS3 MTD1 YAP1 KRE1
Gal4p	31.25%	0.41%	4.15E-02	CIS3 PPZ1 MLF3 KRE1 YPL071C
Cst6p	56.25%	0.33%	4.32E-02	AGP2 EDC2 YHR033W COX5b CIS3 MTD1 YAP1 PPZ1 YPL071C
Swi5p	43.75%	0.36%	4.42E-02	AGP2 YHR033W COX5b CIS3 YNL046W KRE1 DSE3
Sko1p	25.00%	0.43%	4.67E-02	YHR033W CIS3 YAP1 DSE3
Dot6p	6.25%	0.63%	4.83E-02	YAP1
YPR196W	6.25%	0.60%	5.27E-02	MTD1
Put3p	18.75%	0.46%	5.35E-02	EDC2 CIS3 KRE1
Wtm2p	6.25%	0.59%	5.39E-02	MTD1
Mig3p	37.50%	0.36%	5.82E-02	AGP2 YHR033W COX5b URA8 YAP1 KRE1
Mcm1p	37.50%	0.36%	5.86E-02	AGP2 EDC2 CIS3 URA8 PPZ1 YPL071C
Hms1p	25.00%	0.40%	6.06E-02	CIS3 MLF3 KRE1 YPL071C
Hap4p	25.00%	0.40%	6.15E-02	EDC2 COX5b CIS3 DSE3
YGR067C	6.25%	0.55%	6.21E-02	EDC2
Sut2p	6.25%	0.54%	6.39E-02	DSE3
YLR278C	12.50%	0.47%	6.59E-02	MTD1 DSE3
Rlm1p	18.75%	0.42%	6.81E-02	YHR033W COX5b CIS3
Hsf1p	37.50%	0.35%	7.06E-02	AGP2 EDC2 CIS3 MTD1 YAP1 DSE3
Gat3p	12.50%	0.45%	7.45E-02	YAP1 MLF3
Aft1p	31.25%	0.36%	7.68E-02	AGP2 YHR033W COX5b CIS3 YPL071C
Xbp1p	31.25%	0.36%	7.68E-02	AGP2 EDC2 COX5b CIS3 MLF3
Msn4p	50.00%	0.31%	7.89E-02	AGP2 YHR033W PIH1 COX5b MTD1 YAP1 PPZ1 YPL071C

Gcn4p	56.25%	0.30%	8.01E-02	EDC2 PIH1 CIS3 URA8 MTD1 YAP1 PPZ1 KRE1 DSE3
Rap1p	56.25%	0.30%	8.13E-02	ISC1 EDC2 YHR033W CIS3 URA8 MTD1 YAP1 YNL046W KRE1
Bas1p	56.25%	0.30%	8.15E-02	EDC2 YHR033W CIS3 URA8 MTD1 YAP1 PPZ1 MLF3 DSE3
Gat4p	6.25%	0.46%	8.43E-02	MLF3
Tda9p	6.25%	0.45%	8.83E-02	YAP1
Ino4p	31.25%	0.34%	9.04E-02	YHR033W PIH1 URA8 DSE3 YPL071C
Ert1p	6.25%	0.44%	9.17E-02	DSE3
Thi2p	18.75%	0.38%	9.53E-02	CIS3 KRE1 YPL071C
Tbf1p	12.50%	0.40%	9.97E-02	MTD1 YAP1
Cbf1p	31.25%	0.33%	1.06E-01	YHR033W CIS3 MTD1 YAP1 DSE3
Zap1p	31.25%	0.32%	1.10E-01	YHR033W COX5b URA8 MTD1 MLF3
Met4p	31.25%	0.32%	1.13E-01	ISC1 EDC2 YHR033W CIS3 YAP1
Gln3p	25.00%	0.34%	1.13E-01	YHR033W URA8 MTD1 KRE1
Rds1p	6.25%	0.38%	1.14E-01	COX5b
Kar4p	18.75%	0.35%	1.18E-01	ISC1 CIS3 KRE1
Sut1p	12.50%	0.36%	1.21E-01	COX5b KRE1
Rds2p	12.50%	0.36%	1.27E-01	CIS3 KRE1
Abf1p	50.00%	0.28%	1.32E-01	AGP2 ISC1 EDC2 YHR033W PIH1 CIS3 YAP1 KRE1
Smp1p	6.25%	0.35%	1.33E-01	CIS3
Yrr1p	25.00%	0.31%	1.46E-01	ISC1 EDC2 CIS3 YAP1
Yhp1p	25.00%	0.31%	1.47E-01	COX5b CIS3 MLF3 DSE3
Hac1p	12.50%	0.33%	1.48E-01	COX5b YPL071C
Hap3p	6.25%	0.33%	1.48E-01	YAP1
Mig2p	6.25%	0.33%	1.49E-01	YHR033W
Pip2p	25.00%	0.31%	1.54E-01	CIS3 MLF3 KRE1 YPL071C
Uga3p	6.25%	0.31%	1.59E-01	EDC2
Crz1p	12.50%	0.31%	1.68E-01	EDC2 YPL071C
Mal33p	12.50%	0.31%	1.68E-01	ISC1 MTD1
Stp2p	12.50%	0.31%	1.70E-01	AGP2 YAP1
Ixr1p	31.25%	0.29%	1.71E-01	AGP2 ISC1 COX5b URA8 DSE3
Msn2p	56.25%	0.26%	1.79E-01	AGP2 YHR033W PIH1 COX5b CIS3 URA8 MTD1 YAP1 PPZ1
Hap1p	6.25%	0.29%	1.82E-01	YAP1
Arg81p	6.25%	0.29%	1.84E-01	URA8
Reb1p	18.75%	0.29%	1.85E-01	CIS3 YAP1 KRE1
Rtg3p	12.50%	0.30%	1.88E-01	EDC2 YPL071C
Dal81p	6.25%	0.27%	1.99E-01	EDC2
Skn7p	18.75%	0.28%	2.04E-01	CIS3 YAP1 KRE1
Mga1p	12.50%	0.28%	2.05E-01	YHR033W YPL071C
Rfx1p	12.50%	0.28%	2.05E-01	KRE1 DSE3
Ume6p	25.00%	0.27%	2.21E-01	AGP2 COX5b CIS3 MLF3
Met31p	6.25%	0.25%	2.32E-01	PIH1
Tos8p	6.25%	0.25%	2.32E-01	MLF3

Rme1p	6.25%	0.24%	2.40E-01	YHR033W
Aro80p	6.25%	0.23%	2.50E-01	YAP1
Rsf2p	6.25%	0.23%	2.54E-01	ISC1
Hcm1p	6.25%	0.23%	2.55E-01	COX5b
Cad1p	12.50%	0.25%	2.63E-01	PIH1 MTD1
Mig1p	6.25%	0.22%	2.70E-01	EDC2
Arr1p	25.00%	0.25%	2.80E-01	AGP2 COX5b CIS3 MTD1
Adr1p	12.50%	0.24%	2.82E-01	AGP2 MTD1
Ifh1p	6.25%	0.19%	3.27E-01	MLF3
Gcr2p	18.75%	0.23%	3.39E-01	COX5b URA8 MTD1
Yrm1p	37.50%	0.23%	3.60E-01	ISC1 COX5b MTD1 PPZ1 DSE3 YPL071C
Cup2p	6.25%	0.17%	3.77E-01	YHR033W
Flo8p	12.50%	0.20%	3.79E-01	EDC2 YAP1
Tup1p	37.50%	0.23%	3.85E-01	YHR033W CIS3 URA8 MTD1 MLF3 KRE1
Mss11p	6.25%	0.17%	4.00E-01	YPL071C
Yox1p	18.75%	0.21%	4.03E-01	COX5b CIS3 DSE3
Ash1p	43.75%	0.22%	4.27E-01	YHR033W COX5b CIS3 URA8 MTD1 PPZ1 DSE3
Swi4p	12.50%	0.19%	4.39E-01	EDC2 CIS3
Leu3p	6.25%	0.15%	4.48E-01	YHR033W
Haa1p	6.25%	0.15%	4.51E-01	YPL071C
Mbp1p	6.25%	0.14%	4.74E-01	YNL046W
Rph1p	6.25%	0.14%	4.84E-01	AGP2
Fkh2p	25.00%	0.20%	5.00E-01	EDC2 YHR033W CIS3 YNL046W
Pdr3p	12.50%	0.17%	5.07E-01	EDC2 YAP1
Rox1p	6.25%	0.11%	6.20E-01	COX5b
Tec1p	43.75%	0.18%	7.04E-01	YHR033W PIH1 CIS3 YAP1 MLF3 KRE1 DSE3
Fkh1p	25.00%	0.15%	7.63E-01	YHR033W CIS3 MTD1 KRE1
Rim101p	12.50%	0.11%	8.27E-01	YNL046W DSE3
Pdr1p	6.25%	0.07%	8.63E-01	EDC2

Subnetwork 5

Transcription Factor	% in user set	% in Yeastract	p-value	Target ORF/Genes
Hot1p	30.00%	2.94%	7.75E-06	TPS1 SPI1 XKS1
Rlm1p	60.00%	0.84%	8.88E-06	YBR071W TPS1 PHM8 SPI1 GRE3 NNR1
Mig1p	50.00%	1.10%	1.07E-05	YBR071W TPS1 SPI1 GRE3 NNR1
Msn1p	40.00%	1.39%	2.17E-05	YBR071W TPS1 PHM8 GRE3
Gcr2p	70.00%	0.53%	4.11E-05	YBR071W TPS1 YBR137W SPI1 GRE3 YMD8 NNR1
Xbp1p	70.00%	0.50%	6.88E-05	YBR071W YBR137W SPI1 XKS1 GRE3 YMD8 NNR1
Bas1p	90.00%	0.30%	1.73E-04	YBR071W TPS1 YBR137W YBR225W PHM8 SPI1 GRE3 YMD8 NNR1
Spt23p	70.00%	0.38%	4.95E-04	YBR071W TPS1 YBR137W PHM8 SPI1 GRE3 NNR1
Sko1p	50.00%	0.54%	6.21E-04	TPS1 PHM8 SPI1 GRE3 NNR1
Aft1p	60.00%	0.43%	7.80E-04	TPS1 YBR225W PHM8 SPI1 GRE3 NNR1
Hap4p	50.00%	0.50%	9.38E-04	YBR071W PHM8 SPI1 XKS1 NNR1
Stb3p	10.00%	2.86%	1.03E-03	TPS1
Swi4p	50.00%	0.47%	1.37E-03	YBR071W PHM8 SPI1 GRE3 NNR1
Zap1p	60.00%	0.39%	1.39E-03	TPS1 PHM8 SPI1 XKS1 GRE3 NNR1
Wtm2p	20.00%	1.18%	1.39E-03	TPS1 SPI1
Rgm1p	30.00%	0.76%	1.50E-03	TPS1 SPI1 NNR1
Mga2p	50.00%	0.45%	1.66E-03	TPS1 YBR137W SPI1 GRE3 NNR1
YGR067C	20.00%	1.09%	1.75E-03	YBR071W TPS1
Oaf3p	30.00%	0.71%	1.87E-03	YBR071W TPS1 YBR137W
Stb5p	50.00%	0.43%	2.15E-03	TPS1 YBR137W PHM8 XKS1 NNR1
Pdr3p	50.00%	0.42%	2.30E-03	TPS1 PHM8 SPI1 GRE3 NNR1
Sok2p	70.00%	0.31%	2.32E-03	TPS1 YBR225W PHM8 SPI1 XKS1 GRE3 NNR1
Mcm1p	60.00%	0.36%	2.33E-03	YBR071W TPS1 YBR137W PHM8 SPI1 YMD8
Hsf1p	60.00%	0.35%	2.98E-03	YBR071W TPS1 PHM8 SPI1 XKS1 GRE3
Fhl1p	50.00%	0.40%	3.13E-03	YBR071W TPS1 YBR137W PHM8 SPI1
Ert1p	20.00%	0.87%	3.32E-03	YBR071W TPS1
Rpn4p	60.00%	0.34%	3.53E-03	TPS1 YBR137W SPI1 XKS1 GRE3 NNR1
Aft2p	30.00%	0.57%	4.20E-03	TPS1 SPI1 GRE3
Ash1p	80.00%	0.25%	4.23E-03	YBR071W TPS1 YBR137W YBR225W PHM8 SPI1 GRE3 NNR1
Sef1p	20.00%	0.79%	4.35E-03	YBR137W YMD8
Msn4p	70.00%	0.27%	5.86E-03	YBR071W TPS1 PHM8 SPI1 XKS1 GRE3 NNR1
Stb4p	10.00%	1.18%	5.94E-03	YBR225W
War1p	10.00%	1.14%	6.35E-03	TPS1
Yox1p	50.00%	0.35%	6.39E-03	YBR071W TPS1 SPI1 GRE3 NNR1
Gzf3p	20.00%	0.69%	6.39E-03	PHM8 SPI1

Pdr1p	50.00%	0.35%	6.65E-03	TPS1 PHM8 SPI1 GRE3 NNR1
Sfp1p	90.00%	0.21%	6.88E-03	YBR071W TPS1 YBR137W YBR225W PHM8 SPI1 XKS1 GRE3 NNR1
Fkh1p	70.00%	0.27%	6.99E-03	YBR071W TPS1 YBR137W PHM8 XKS1 GRE3 YMD8
Urc2p	20.00%	0.66%	7.22E-03	YBR137W PHM8
Yap1p	80.00%	0.23%	8.26E-03	YBR071W TPS1 YBR137W PHM8 SPI1 XKS1 GRE3 NNR1
Crz1p	30.00%	0.47%	8.64E-03	YBR071W TPS1 SPI1
Cst6p	70.00%	0.26%	9.05E-03	TPS1 PHM8 SPI1 XKS1 GRE3 YMD8 NNR1
Skn7p	40.00%	0.38%	9.36E-03	YBR071W TPS1 SPI1 NNR1
Put3p	30.00%	0.46%	9.63E-03	SPI1 GRE3 YMD8
Met4p	50.00%	0.32%	9.68E-03	YBR071W PHM8 SPI1 GRE3 NNR1
Haa1p	30.00%	0.45%	1.02E-02	PHM8 SPI1 NNR1
Mga1p	30.00%	0.43%	1.22E-02	YBR137W SPI1 GRE3
Rph1p	30.00%	0.42%	1.26E-02	TPS1 XKS1 NNR1
Gis1p	20.00%	0.51%	1.48E-02	PHM8 SPI1
Arg80p	20.00%	0.51%	1.52E-02	SPI1 NNR1
Met31p	20.00%	0.49%	1.65E-02	SPI1 GRE3
Gcn4p	70.00%	0.23%	1.71E-02	YBR071W TPS1 YBR225W PHM8 SPI1 GRE3 NNR1
Sfl1p	10.00%	0.68%	1.73E-02	SPI1
Rme1p	20.00%	0.48%	1.75E-02	SPI1 NNR1
Ace2p	90.00%	0.19%	1.81E-02	YBR071W YBR137W YBR225W PHM8 SPI1 XKS1 GRE3 YMD8 NNR1
Dot6p	10.00%	0.63%	1.98E-02	YBR071W
Adr1p	30.00%	0.36%	2.13E-02	TPS1 PHM8 SPI1
Yhp1p	40.00%	0.31%	2.15E-02	YBR071W TPS1 GRE3 NNR1
YPR196W	10.00%	0.60%	2.17E-02	SPI1
Rpi1p	10.00%	0.57%	2.34E-02	SPI1
Stp4p	10.00%	0.57%	2.39E-02	SPI1
Yap5p	30.00%	0.34%	2.58E-02	XKS1 YMD8 NNR1
Wtm1p	10.00%	0.53%	2.73E-02	TPS1
Azf1p	10.00%	0.51%	2.98E-02	NNR1
Swi5p	50.00%	0.26%	3.00E-02	YBR071W PHM8 SPI1 XKS1 NNR1
Dal80p	10.00%	0.50%	3.06E-02	YBR225W
Gat4p	10.00%	0.46%	3.56E-02	SPI1
Sut1p	20.00%	0.36%	3.63E-02	SPI1 GRE3
Flo8p	30.00%	0.31%	3.72E-02	TPS1 SPI1 GRE3
Cat8p	10.00%	0.44%	3.90E-02	SPI1
Met28p	10.00%	0.43%	3.93E-02	YBR071W
Cup2p	20.00%	0.35%	4.15E-02	SPI1 NNR1
Cbf1p	40.00%	0.26%	4.27E-02	TPS1 YBR225W SPI1 GRE3
Sum1p	20.00%	0.32%	4.94E-02	YBR071W TPS1
Rds1p	10.00%	0.38%	4.94E-02	SPI1
Abf1p	60.00%	0.21%	5.09E-02	YBR225W PHM8 SPI1 XKS1 GRE3 YMD8

Cin5p	50.00%	0.23%	5.16E-02	YBR071W TPS1 YBR225W SPI1 NNR1
Hap2p	40.00%	0.25%	5.33E-02	YBR137W SPI1 YMD8 NNR1
Mal33p	20.00%	0.31%	5.35E-02	YBR071W YBR137W
Ino2p	20.00%	0.30%	5.80E-02	YBR071W SPI1
Smp1p	10.00%	0.35%	5.83E-02	YBR071W
Mbp1p	20.00%	0.29%	6.64E-02	YBR071W SPI1
Rfx1p	20.00%	0.28%	6.78E-02	SPI1 NNR1
Upc2p	10.00%	0.31%	7.04E-02	SPI1
Stp1p	20.00%	0.28%	7.22E-02	YBR071W TPS1
Nrg2p	10.00%	0.29%	8.20E-02	NNR1
Hap1p	10.00%	0.29%	8.24E-02	GRE3
Arg81p	10.00%	0.29%	8.33E-02	YBR225W
Dal81p	10.00%	0.27%	9.12E-02	YBR071W
Opi1p	10.00%	0.26%	9.68E-02	SPI1
Tos8p	10.00%	0.25%	1.08E-01	GRE3
YLR278C	10.00%	0.24%	1.16E-01	SPI1
Pho4p	30.00%	0.21%	1.16E-01	YBR071W PHM8 SPI1
Oaf1p	20.00%	0.22%	1.19E-01	YBR071W TPS1
Rox1p	20.00%	0.22%	1.23E-01	SPI1 NNR1
Gat3p	10.00%	0.22%	1.26E-01	XKS1
Hap5p	10.00%	0.22%	1.28E-01	YBR137W
Mac1p	10.00%	0.22%	1.35E-01	SPI1
Msn2p	60.00%	0.18%	1.39E-01	TPS1 PHM8 SPI1 XKS1 GRE3 NNR1
Yap6p	20.00%	0.20%	1.47E-01	XKS1 NNR1
Rgt1p	10.00%	0.20%	1.51E-01	PHM8
Tbf1p	10.00%	0.20%	1.55E-01	PHM8
Reb1p	20.00%	0.20%	1.61E-01	SPI1 NNR1
Arr1p	30.00%	0.19%	1.68E-01	TPS1 PHM8 XKS1
Ecm22p	10.00%	0.19%	1.68E-01	YMD8
Met32p	10.00%	0.18%	1.77E-01	YBR225W
Gcr1p	30.00%	0.18%	1.78E-01	PHM8 SPI1 YMD8
Pho2p	20.00%	0.19%	1.82E-01	YBR137W SPI1
Rds2p	10.00%	0.18%	1.83E-01	SPI1
Ndt80p	10.00%	0.17%	1.89E-01	YMD8
Mot3p	10.00%	0.17%	1.97E-01	PHM8
Hac1p	10.00%	0.17%	2.05E-01	YBR137W
Ixr1p	30.00%	0.17%	2.08E-01	PHM8 SPI1 XKS1
Stp2p	10.00%	0.16%	2.26E-01	SPI1
Tec1p	60.00%	0.16%	2.36E-01	YBR071W YBR225W PHM8 SPI1 XKS1 GRE3
Rtg3p	10.00%	0.15%	2.43E-01	GRE3
Yap7p	10.00%	0.14%	2.53E-01	TPS1
Nrg1p	10.00%	0.14%	2.70E-01	NNR1
Pip2p	20.00%	0.15%	2.72E-01	TPS1 YBR137W
Yrm1p	40.00%	0.15%	2.83E-01	YBR137W SPI1 YMD8 NNR1
Phd1p	10.00%	0.13%	2.92E-01	SPI1
Dal82p	10.00%	0.12%	3.18E-01	YBR071W

Ino4p	20.00%	0.14%	3.38E-01	SPI1 GRE3
Hms1p	10.00%	0.10%	4.16E-01	TPS1
Mig3p	20.00%	0.12%	4.26E-01	SPI1 XKS1
Ste12p	50.00%	0.13%	4.79E-01	YBR071W YBR225W PHM8 SPI1 NNR1
Rim101p	20.00%	0.11%	5.04E-01	SPI1 GRE3
Gln3p	10.00%	0.08%	5.15E-01	YBR137W
Gal4p	10.00%	0.08%	5.29E-01	TPS1
Tye7p	10.00%	0.08%	5.39E-01	TPS1
Tup1p	30.00%	0.11%	5.49E-01	YBR071W SPI1 YMD8
Yrr1p	10.00%	0.08%	5.61E-01	PHM8
Ume6p	10.00%	0.07%	6.43E-01	YBR071W
Rap1p	30.00%	0.10%	6.69E-01	YBR071W TPS1 YMD8

Subnetwork 6

Transcription Factor	% in user set	% in Yeastract	p-value	Target ORF/Genes
Rpn4p	66.67%	0.34%	1.35E-03	RPN6 CDC53 RPN5 SPT16 RPN1 RPT5
Tos4p	22.22%	0.99%	1.66E-03	SPT16 CCT2
Mot2p	11.11%	0.81%	9.96E-03	SPT16
Oaf1p	33.33%	0.34%	1.82E-02	CDC53 SPT16 RPT5
Abf1p	66.67%	0.21%	2.31E-02	RPN5 SPT16 RPN1 SMF2 CCT2 RPT5
Reb1p	33.33%	0.29%	2.85E-02	RPN6 CDC53 RPT5
Mss11p	22.22%	0.33%	3.48E-02	SEN1 RPT5
Sip4p	11.11%	0.40%	3.66E-02	SEN1
Hap3p	11.11%	0.33%	5.41E-02	SMF2
Stp1p	22.22%	0.28%	5.45E-02	RPN5 SEN1
Nrg2p	11.11%	0.29%	6.77E-02	SEN1
Dal82p	22.22%	0.25%	7.33E-02	SPT16 SEN1
Dal81p	11.11%	0.27%	7.55E-02	CDC53
Sfp1p	77.78%	0.16%	7.71E-02	RPN6 RPN5 SPT16 SMF2 CCT2 SEN1 RPT5
Gis1p	11.11%	0.26%	8.39E-02	CDC53
Pdr1p	33.33%	0.21%	8.80E-02	CDC53 RPN5 SEN1
Met31p	11.11%	0.25%	9.00E-02	SMF2
Rme1p	11.11%	0.24%	9.35E-02	CDC53
Oaf3p	11.11%	0.24%	9.46E-02	SMF2
Cbf1p	33.33%	0.20%	1.05E-01	RPN6 SPT16 CCT2
Hap5p	11.11%	0.22%	1.07E-01	SMF2
Mac1p	11.11%	0.22%	1.13E-01	SMF2
Mcm1p	33.33%	0.18%	1.38E-01	RPN5 SMF2 CCT2
Swi4p	22.22%	0.19%	1.41E-01	SMF2 CCT2
Ecm22p	11.11%	0.19%	1.41E-01	RPT5
Met32p	11.11%	0.18%	1.49E-01	SEN1
Rim101p	33.33%	0.16%	1.85E-01	RPN5 SPT16 SEN1
Mal33p	11.11%	0.16%	1.90E-01	SEN1
Stp2p	11.11%	0.16%	1.92E-01	CDC53
Ino2p	11.11%	0.15%	2.00E-01	RPN6
Tup1p	44.44%	0.15%	2.13E-01	SPT16 RPN1 SMF2 CCT2
Mbp1p	11.11%	0.14%	2.18E-01	SEN1
Pip2p	22.22%	0.15%	2.18E-01	SPT16 RPN1
Rph1p	11.11%	0.14%	2.24E-01	CCT2
Nrg1p	11.11%	0.14%	2.30E-01	SEN1
Cst6p	44.44%	0.15%	2.32E-01	RPN6 RPN1 CCT2 SEN1
Pho4p	22.22%	0.14%	2.55E-01	RPN1 SMF2
Yox1p	22.22%	0.14%	2.63E-01	CDC53 SPT16
Adr1p	11.11%	0.12%	2.81E-01	SPT16
Cin5p	33.33%	0.14%	2.84E-01	SPT16 SEN1 RPT5
Hap2p	22.22%	0.12%	3.33E-01	RPN1 SMF2
Gcr1p	22.22%	0.12%	3.41E-01	RPN6 CCT2

Yap6p	11.11%	0.10%	3.55E-01	RPT5
Mig3p	22.22%	0.12%	3.55E-01	RPN5 SMF2
Flo8p	11.11%	0.10%	3.56E-01	RPN6
Hms1p	11.11%	0.10%	3.63E-01	RPN6
Hap4p	11.11%	0.10%	3.65E-01	SMF2
Ace2p	66.67%	0.13%	3.85E-01	RPN5 SPT16 SMF2 CCT2 SEN1 RPT5
Skn7p	11.11%	0.09%	3.95E-01	RPN1
Pho2p	11.11%	0.09%	4.03E-01	SEN1
Cup9p	11.11%	0.09%	4.09E-01	RPN1
Spt23p	22.22%	0.11%	4.17E-01	RPN5 SEN1
Mga2p	11.11%	0.09%	4.19E-01	RPN5
Fkh1p	33.33%	0.11%	4.39E-01	CDC53 RPN1 SMF2
Stb5p	11.11%	0.09%	4.45E-01	SEN1
Yap1p	44.44%	0.12%	4.62E-01	RPN6 RPN5 RPN1 CCT2
Tye7p	11.11%	0.08%	4.80E-01	SPT16
Yrr1p	11.11%	0.08%	5.02E-01	SPT16
Yhp1p	11.11%	0.08%	5.03E-01	RPN5
Xbp1p	11.11%	0.07%	5.55E-01	SMF2
Aft1p	11.11%	0.07%	5.55E-01	RPT5
Rap1p	33.33%	0.10%	5.69E-01	CDC53 RPN5 SPT16
Ino4p	11.11%	0.07%	5.80E-01	RPN6
Ume6p	11.11%	0.07%	5.83E-01	CCT2
Zap1p	11.11%	0.06%	6.10E-01	RPT5
Met4p	11.11%	0.06%	6.14E-01	CCT2
Yrm1p	22.22%	0.08%	6.98E-01	SMF2 SEN1
Swi5p	11.11%	0.05%	7.51E-01	CDC53
Fkh2p	11.11%	0.05%	7.78E-01	SMF2
Tec1p	33.33%	0.08%	8.11E-01	CDC53 SPT16 SEN1
Ash1p	22.22%	0.06%	8.46E-01	SPT16 SEN1
Msn2p	22.22%	0.06%	8.88E-01	CDC53 SMF2
Msn4p	11.11%	0.04%	8.91E-01	SMF2
Gcn4p	11.11%	0.03%	9.44E-01	CDC53
Bas1p	11.11%	0.03%	9.45E-01	SMF2
Ste12p	11.11%	0.03%	9.89E-01	RPN1

Subnetwork 7

Transcription Factor	% in user set	% in Yeastract	p-value	Target ORF/Genes
Rpi1p	25.00%	1.15%	7.30E-04	YDL211C YLR257W
Cin5p	75.00%	0.28%	1.46E-03	YDL211C HLR1 MSP1 SNX4 LHS1 YLR257W
Hac1p	37.50%	0.50%	2.70E-03	HLR1 MSP1 LHS1
Tup1p	75.00%	0.23%	5.42E-03	MSP1 SNP1 SNX4 LHS1 YLR257W COG8
Tec1p	87.50%	0.18%	6.73E-03	YDL211C HLR1 MSP1 SNP1 SNX4 LHS1 YLR257W
Ste12p	87.50%	0.18%	7.22E-03	HLR1 MSP1 SNP1 SNX4 LHS1 YLR257W COG8
Mot2p	12.50%	0.81%	7.84E-03	YDL211C
Aro80p	25.00%	0.47%	9.64E-03	YDL211C LHS1
Wtm2p	12.50%	0.59%	1.42E-02	YLR257W
YJL206C	12.50%	0.58%	1.47E-02	LHS1
YGR067C	12.50%	0.55%	1.66E-02	YLR257W
Ppr1p	12.50%	0.52%	1.85E-02	YLR257W
Met32p	25.00%	0.36%	1.90E-02	YDL211C HLR1
Skn7p	37.50%	0.28%	2.05E-02	HLR1 SNP1 YLR257W
Hsf1p	50.00%	0.23%	2.45E-02	YDL211C HLR1 MSP1 LHS1
Gat1p	12.50%	0.44%	2.49E-02	SNX4
Sum1p	25.00%	0.32%	2.63E-02	YDL211C YLR257W
Msn2p	75.00%	0.18%	2.68E-02	YDL211C HLR1 MSP1 SNX4 YLR257W COG8
Crz1p	25.00%	0.31%	2.85E-02	YDL211C YLR257W
Mal33p	25.00%	0.31%	2.86E-02	YDL211C LHS1
Ino2p	25.00%	0.30%	3.11E-02	MSP1 LHS1
Haa1p	25.00%	0.30%	3.21E-02	MSP1 YLR257W
Hal9p	12.50%	0.37%	3.51E-02	YDL211C
Rfx1p	25.00%	0.28%	3.67E-02	SNP1 LHS1
Aca1p	12.50%	0.35%	3.80E-02	YDL211C
Swi5p	50.00%	0.21%	3.92E-02	YDL211C HLR1 SNX4 LHS1
Ace2p	87.50%	0.15%	4.04E-02	YDL211C HLR1 SNP1 SNX4 LHS1 YLR257W COG8
Hap3p	12.50%	0.33%	4.33E-02	LHS1
Phd1p	25.00%	0.26%	4.54E-02	HLR1 LHS1
Uga3p	12.50%	0.31%	4.67E-02	YLR257W
Fkh2p	50.00%	0.20%	4.77E-02	MSP1 SNX4 YLR257W COG8
Cad1p	25.00%	0.25%	5.08E-02	YDL211C YLR257W
Pho4p	37.50%	0.21%	5.39E-02	MSP1 SNX4 YLR257W
Arg81p	12.50%	0.29%	5.52E-02	LHS1
Yox1p	37.50%	0.21%	5.65E-02	YDL211C HLR1 MSP1
Dal81p	12.50%	0.27%	6.08E-02	YLR257W
Kar4p	25.00%	0.23%	6.16E-02	YDL211C SNX4

Gcn4p	62.50%	0.17%	6.32E-02	YDL211C HLR1 SNP1 LHS1 YLR257W
Yap5p	25.00%	0.23%	6.43E-02	HLR1 COG8
Oaf1p	25.00%	0.22%	6.73E-02	HLR1 YLR257W
Rgm1p	12.50%	0.25%	6.89E-02	YLR257W
Cbf1p	37.50%	0.20%	6.97E-02	HLR1 LHS1 YLR257W
Sok2p	50.00%	0.18%	7.16E-02	YDL211C HLR1 SNX4 YLR257W
Zap1p	37.50%	0.19%	7.18E-02	YDL211C LHS1 YLR257W
Rme1p	12.50%	0.24%	7.55E-02	YDL211C
Hcm1p	12.50%	0.23%	8.14E-02	HLR1
Gat3p	12.50%	0.22%	8.53E-02	YLR257W
Hap5p	12.50%	0.22%	8.63E-02	HLR1
Mig1p	12.50%	0.22%	8.73E-02	HLR1
Reb1p	25.00%	0.20%	9.30E-02	LHS1 YLR257W
Mcm1p	37.50%	0.18%	9.32E-02	HLR1 MSP1 YLR257W
Ifh1p	12.50%	0.19%	1.11E-01	MSP1
Aft2p	12.50%	0.19%	1.12E-01	YLR257W
Mga2p	25.00%	0.18%	1.14E-01	MSP1 LHS1
Spt23p	37.50%	0.16%	1.23E-01	MSP1 SNX4 YLR257W
Cup2p	12.50%	0.17%	1.33E-01	YLR257W
Gln3p	25.00%	0.17%	1.35E-01	LHS1 YLR257W
Cst6p	50.00%	0.15%	1.48E-01	MSP1 SNP1 LHS1 COG8
Yhp1p	25.00%	0.16%	1.62E-01	HLR1 YLR257W
Mbp1p	12.50%	0.14%	1.80E-01	HLR1
Rph1p	12.50%	0.14%	1.86E-01	LHS1
Rlm1p	12.50%	0.14%	1.87E-01	LHS1
Stp1p	12.50%	0.14%	1.90E-01	YLR257W
Xbp1p	25.00%	0.14%	1.97E-01	LHS1 YLR257W
Ino4p	25.00%	0.14%	2.15E-01	MSP1 LHS1
Ume6p	25.00%	0.14%	2.17E-01	SNX4 LHS1
Thi2p	12.50%	0.13%	2.23E-01	YDL211C
Dal82p	12.50%	0.12%	2.29E-01	YDL211C
Met4p	25.00%	0.13%	2.42E-01	SNX4 COG8
Ash1p	50.00%	0.13%	2.54E-01	YDL211C SNX4 LHS1 COG8
Rox1p	12.50%	0.11%	2.69E-01	COG8
Gcr1p	25.00%	0.12%	2.70E-01	LHS1 YLR257W
Mig3p	25.00%	0.12%	2.82E-01	YDL211C YLR257W
Flo8p	12.50%	0.10%	3.02E-01	YLR257W
Hap4p	12.50%	0.10%	3.11E-01	HLR1
Msn4p	37.50%	0.12%	3.15E-01	YDL211C HLR1 MSP1
Rpn4p	25.00%	0.11%	3.23E-01	SNP1 YLR257W
Yrm1p	37.50%	0.12%	3.27E-01	SNX4 LHS1 COG8
Yap1p	50.00%	0.12%	3.30E-01	YDL211C SNX4 LHS1 YLR257W
Sfp1p	62.50%	0.12%	3.32E-01	YDL211C MSP1 SNX4 YLR257W COG8
Fkh1p	37.50%	0.11%	3.35E-01	SNX4 YLR257W COG8
Rim101p	25.00%	0.11%	3.47E-01	SNX4 YLR257W
Cup9p	12.50%	0.09%	3.51E-01	SNX4
Pdr3p	12.50%	0.08%	3.91E-01	MSP1

Tye7p	12.50%	0.08%	4.17E-01	YDL211C
Fhl1p	12.50%	0.08%	4.21E-01	MSP1
Yrr1p	12.50%	0.08%	4.37E-01	MSP1
Bas1p	37.50%	0.10%	4.55E-01	YDL211C SNX4 LHS1
Pdr1p	12.50%	0.07%	5.02E-01	MSP1
Arr1p	12.50%	0.06%	5.67E-01	HLR1
Ixr1p	12.50%	0.06%	6.15E-01	SNP1
Abf1p	25.00%	0.07%	6.74E-01	LHS1 YLR257W
Rap1p	25.00%	0.07%	7.27E-01	MSP1 YLR257W

Subnetwork 8

Transcription Factor	% in user set	% in Yeastract	p-value	Target ORF/Genes
Upc2p	25.00%	0.63%	4.16E-03	ERG3 ERG24
Yap3p	25.00%	0.60%	4.82E-03	PRR1 ERG3
Aro80p	25.00%	0.47%	9.64E-03	PRR1 ERG24
Mig1p	25.00%	0.44%	1.12E-02	PCM1 ERG24
Rpi1p	12.50%	0.57%	1.50E-02	MSC7
Plm2p	12.50%	0.54%	1.69E-02	MTR3
Ecm22p	25.00%	0.38%	1.74E-02	MSC7 ERG3
Wtm1p	12.50%	0.53%	1.76E-02	MTR3
Fzf1p	12.50%	0.49%	2.03E-02	TAE2
Ndt80p	25.00%	0.35%	2.12E-02	ERG3 APM4
Cup9p	37.50%	0.28%	2.26E-02	PCM1 PRR1 ERG3
Tda9p	12.50%	0.45%	2.43E-02	ERG3
Rpn4p	50.00%	0.22%	2.75E-02	PCM1 PRR1 ERG3 ERG24
Put3p	25.00%	0.30%	3.09E-02	PCM1 ERG3
Leu3p	25.00%	0.30%	3.17E-02	PRR1 ERG3
Gal4p	37.50%	0.25%	3.30E-02	PCM1 PRR1 ERG3
Tye7p	37.50%	0.24%	3.51E-02	PCM1 PRR1 ERG3
Aca1p	12.50%	0.35%	3.80E-02	ERG3
Swi5p	50.00%	0.21%	3.92E-02	MTR3 MSC7 PRR1 ERG3
Yhp1p	37.50%	0.23%	4.01E-02	MSC7 PRR1 ERG3
Ace2p	87.50%	0.15%	4.04E-02	PCM1 MTR3 MSC7 PRR1 ERG3 APM4 TAE2
Pip2p	37.50%	0.23%	4.21E-02	PCM1 PRR1 ERG3
Tec1p	75.00%	0.16%	5.35E-02	PCM1 MSC7 PRR1 ERG24 APM4 TAE2
Hap1p	12.50%	0.29%	5.47E-02	ERG3
Yox1p	37.50%	0.21%	5.65E-02	MSC7 PRR1 ERG3
Bas1p	62.50%	0.17%	6.40E-02	PCM1 MSC7 PRR1 ERG3 TAE2
Opi1p	12.50%	0.26%	6.46E-02	MSC7
Tos8p	12.50%	0.25%	7.27E-02	MSC7
Hcm1p	12.50%	0.23%	8.14E-02	PCM1
Arr1p	37.50%	0.19%	8.15E-02	PRR1 ERG3 ERG24
Gat3p	12.50%	0.22%	8.53E-02	APM4
Hms1p	25.00%	0.20%	8.80E-02	PCM1 ERG3
Mac1p	12.50%	0.22%	9.14E-02	ERG3
Mig3p	37.50%	0.18%	9.29E-02	MSC7 ERG24 APM4
Rgt1p	12.50%	0.20%	1.03E-01	ERG3
Swi4p	25.00%	0.19%	1.05E-01	ERG3 TAE2
Tbf1p	12.50%	0.20%	1.06E-01	ERG3
Ifh1p	12.50%	0.19%	1.11E-01	MSC7
Mga2p	25.00%	0.18%	1.14E-01	MTR3 ERG3
Yrm1p	50.00%	0.15%	1.25E-01	MTR3 ERG3 APM4 TAE2
Rds2p	12.50%	0.18%	1.26E-01	ERG3
Stb5p	25.00%	0.17%	1.28E-01	PCM1 ERG3

Rim101p	37.50%	0.16%	1.28E-01	PCM1 ERG3 ERG24
Gln3p	25.00%	0.17%	1.35E-01	MTR3 PRR1
Tup1p	50.00%	0.15%	1.35E-01	PCM1 MTR3 PRR1 ERG3
Mot3p	12.50%	0.17%	1.37E-01	ERG3
Hac1p	12.50%	0.17%	1.43E-01	ERG3
Cst6p	50.00%	0.15%	1.48E-01	PCM1 MTR3 PRR1 ERG3
Mal33p	12.50%	0.16%	1.57E-01	PRR1
Ino2p	12.50%	0.15%	1.65E-01	ERG3
Haa1p	12.50%	0.15%	1.69E-01	MSC7
Yap7p	12.50%	0.14%	1.79E-01	ERG3
Mga1p	12.50%	0.14%	1.83E-01	PCM1
Rfx1p	12.50%	0.14%	1.83E-01	ERG3
Rlm1p	12.50%	0.14%	1.87E-01	PCM1
Aft1p	25.00%	0.14%	1.97E-01	ERG3 ERG24
Pho4p	25.00%	0.14%	1.98E-01	PRR1 APM4
Cin5p	37.50%	0.14%	2.05E-01	PCM1 PRR1 ERG3
Ino4p	25.00%	0.14%	2.15E-01	ERG3 TAE2
Thi2p	12.50%	0.13%	2.23E-01	ERG3
Sok2p	37.50%	0.13%	2.25E-01	PCM1 ERG3 ERG24
Dal82p	12.50%	0.12%	2.29E-01	ERG24
Zap1p	25.00%	0.13%	2.39E-01	ERG3 ERG24
Kar4p	12.50%	0.12%	2.50E-01	ERG3
Oaf1p	12.50%	0.11%	2.64E-01	PCM1
Rox1p	12.50%	0.11%	2.69E-01	ERG3
Gcr1p	25.00%	0.12%	2.70E-01	PCM1 ERG3
Mcm1p	25.00%	0.12%	2.83E-01	PCM1 ERG3
Yap6p	12.50%	0.10%	3.01E-01	APM4
Hap4p	12.50%	0.10%	3.11E-01	PCM1
Msn4p	37.50%	0.12%	3.15E-01	MSC7 ERG3 TAE2
Sfp1p	62.50%	0.12%	3.32E-01	PCM1 MTR3 MSC7 ERG3 TAE2
Pho2p	12.50%	0.09%	3.45E-01	TAE2
Fhl1p	12.50%	0.08%	4.21E-01	PCM1
Yrr1p	12.50%	0.08%	4.37E-01	MTR3
Rap1p	37.50%	0.10%	4.55E-01	PCM1 PRR1 ERG3
Xbp1p	12.50%	0.07%	4.88E-01	ERG3
Pdr1p	12.50%	0.07%	5.02E-01	MTR3
Ash1p	37.50%	0.09%	5.16E-01	ERG3 APM4 TAE2
Yap1p	37.50%	0.09%	6.02E-01	ERG3 ERG24 TAE2
Ixr1p	12.50%	0.06%	6.15E-01	PRR1
Hsf1p	12.50%	0.06%	6.17E-01	TAE2
Spt23p	12.50%	0.05%	6.48E-01	ERG3
Ste12p	37.50%	0.08%	7.21E-01	PRR1 ERG3 ERG24
Gcn4p	25.00%	0.07%	7.25E-01	PCM1 APM4
Msn2p	25.00%	0.06%	8.26E-01	ERG3 TAE2
Abf1p	12.50%	0.04%	8.88E-01	ERG3

Subnetwork 9

Transcription Factor	% in user set	% in Yeastract	p-value	Target ORF/Genes
Mcm1p	71.43%	0.30%	9.22E-04	UBP5 YGL193C OST5 STE18 DIA2
Yrr1p	57.14%	0.31%	2.87E-03	UBP5 YGL193C OST5 EMC2
Rsc30p	14.29%	0.98%	4.06E-03	STE18
Hms2p	14.29%	0.87%	5.13E-03	EMC2
Hap2p	57.14%	0.25%	8.18E-03	YGL193C STE18 ELF1 DIA2
Dot6p	14.29%	0.63%	9.63E-03	STE18
Reb1p	42.86%	0.29%	1.00E-02	YGL193C OST5 STE18
Fkh1p	71.43%	0.19%	1.21E-02	UBP5 YGL193C OST5 STE18 EMC2
Wtm1p	14.29%	0.53%	1.34E-02	EMC2
Abf1p	71.43%	0.18%	1.75E-02	UBP5 OST5 STE18 EMC2 ELF1
Tda9p	14.29%	0.45%	1.86E-02	ELF1
Tye7p	42.86%	0.24%	2.03E-02	STE18 EMC2 ELF1
Pip2p	42.86%	0.23%	2.46E-02	YGL193C STE18 ELF1
Rfx1p	28.57%	0.28%	2.47E-02	UBP5 EMC2
Mga1p	28.57%	0.28%	2.47E-02	EMC2 DIA2
Msn1p	14.29%	0.35%	2.96E-02	UBP5
Aft1p	42.86%	0.21%	3.16E-02	UBP5 YGL193C STE18
Hap3p	14.29%	0.33%	3.34E-02	YGL193C
Thi2p	28.57%	0.25%	3.47E-02	STE18 ELF1
Upc2p	14.29%	0.31%	3.59E-02	EMC2
Uga3p	14.29%	0.31%	3.61E-02	ELF1
Yap3p	14.29%	0.30%	3.95E-02	OST5
Mth1p	14.29%	0.29%	4.21E-02	ELF1
Kar4p	28.57%	0.23%	4.22E-02	YGL193C ELF1
Hap1p	14.29%	0.29%	4.23E-02	YGL193C
Tos8p	14.29%	0.25%	5.66E-02	EMC2
Flo8p	28.57%	0.20%	5.87E-02	STE18 DIA2
YLR278C	14.29%	0.24%	6.09E-02	ELF1
Hap4p	28.57%	0.20%	6.18E-02	YGL193C OST5
Aro80p	14.29%	0.23%	6.20E-02	ELF1
Hsf1p	42.86%	0.17%	6.49E-02	UBP5 OST5 DIA2
Hap5p	14.29%	0.22%	6.75E-02	YGL193C
Tup1p	57.14%	0.15%	7.26E-02	UBP5 YGL193C ELF1 DIA2
Cup9p	28.57%	0.18%	7.68E-02	STE18 ELF1
Cst6p	57.14%	0.15%	8.06E-02	UBP5 YGL193C ELF1 DIA2
Stb5p	28.57%	0.17%	9.06E-02	UBP5 EMC2
Rds2p	14.29%	0.18%	9.98E-02	ELF1
Gal4p	28.57%	0.16%	1.01E-01	STE18 ELF1
Fhl1p	28.57%	0.16%	1.08E-01	UBP5 STE18
Hac1p	14.29%	0.17%	1.13E-01	ELF1
Mss11p	14.29%	0.17%	1.14E-01	ELF1
Rap1p	57.14%	0.13%	1.18E-01	UBP5 OST5 EMC2 DIA2
Crz1p	14.29%	0.16%	1.24E-01	UBP5

Mal33p	14.29%	0.16%	1.25E-01	ELF1
Stp2p	14.29%	0.16%	1.26E-01	STE18
Put3p	14.29%	0.15%	1.31E-01	ELF1
Leu3p	14.29%	0.15%	1.33E-01	ELF1
Haa1p	14.29%	0.15%	1.34E-01	OST5
Rtg3p	14.29%	0.15%	1.36E-01	ELF1
Yox1p	28.57%	0.14%	1.49E-01	STE18 ELF1
Dal82p	14.29%	0.12%	1.85E-01	UBP5
Adr1p	14.29%	0.12%	1.91E-01	UBP5
Mig3p	28.57%	0.12%	2.11E-01	YGL193C STE18
Yrm1p	42.86%	0.12%	2.26E-01	UBP5 YGL193C DIA2
Rpn4p	28.57%	0.11%	2.45E-01	YGL193C EMC2
Hms1p	14.29%	0.10%	2.54E-01	ELF1
Spt23p	28.57%	0.11%	2.57E-01	UBP5 DIA2
Skn7p	14.29%	0.09%	2.79E-01	OST5
Swi4p	14.29%	0.09%	2.83E-01	DIA2
Pho2p	14.29%	0.09%	2.85E-01	STE18
Tec1p	57.14%	0.10%	2.89E-01	UBP5 YGL193C STE18 DIA2
Swi5p	28.57%	0.10%	2.92E-01	OST5 ELF1
Mga2p	14.29%	0.09%	2.98E-01	UBP5
Gln3p	14.29%	0.08%	3.30E-01	YGL193C
Cin5p	28.57%	0.09%	3.66E-01	STE18 ELF1
Yhp1p	14.29%	0.08%	3.69E-01	STE18
Ash1p	42.86%	0.09%	3.85E-01	UBP5 OST5 DIA2
Xbp1p	14.29%	0.07%	4.15E-01	OST5
Pho4p	14.29%	0.07%	4.17E-01	DIA2
Pdr1p	14.29%	0.07%	4.28E-01	OST5
Sfp1p	57.14%	0.09%	4.37E-01	UBP5 YGL193C STE18 DIA2
Ino4p	14.29%	0.07%	4.38E-01	UBP5
Cbf1p	14.29%	0.07%	4.61E-01	OST5
Zap1p	14.29%	0.06%	4.66E-01	DIA2
Arr1p	14.29%	0.06%	4.90E-01	YGL193C
Msn4p	28.57%	0.08%	4.91E-01	STE18 DIA2
Gcr1p	14.29%	0.06%	5.02E-01	OST5
Ste12p	42.86%	0.08%	5.87E-01	UBP5 YGL193C STE18
Gcn4p	28.57%	0.07%	6.23E-01	ELF1 DIA2
Fkh2p	14.29%	0.05%	6.40E-01	OST5
Msn2p	28.57%	0.06%	7.38E-01	OST5 STE18
Yap1p	28.57%	0.06%	7.47E-01	UBP5 DIA2
Ace2p	42.86%	0.06%	8.31E-01	UBP5 OST5 ELF1
Bas1p	14.29%	0.03%	8.67E-01	UBP5

Subnetwork 10

Transcription Factor	% in user set	% in Yeastract	p-value	Target ORF/Genes
Ppr1p	33.33%	1.03%	3.73E-04	SRB4 YKR051W
YNR063W	16.67%	1.85%	8.28E-04	ASP3-1
Rfx1p	50.00%	0.43%	1.20E-03	SRB4 RNR3 ASP3-1
Ngg1p	16.67%	1.45%	1.35E-03	ASP3-1
Gal80p	16.67%	1.16%	2.09E-03	AVT2
Ime1p	16.67%	0.81%	4.30E-03	ASP3-1
Hap5p	33.33%	0.44%	4.32E-03	AVT2 RNR3
YPR015C	16.67%	0.72%	5.30E-03	RNR3
Gsm1p	16.67%	0.59%	7.86E-03	ASP3-1
Cup2p	33.33%	0.35%	8.83E-03	ASP3-1 EXO1
Sut2p	16.67%	0.54%	9.47E-03	ASP3-1
Azf1p	16.67%	0.51%	1.07E-02	RNR3
Cha4p	16.67%	0.46%	1.27E-02	ASP3-1
Ino2p	33.33%	0.30%	1.28E-02	RNR3 ASP3-1
Gat4p	16.67%	0.46%	1.29E-02	RNR3
Gat1p	16.67%	0.44%	1.39E-02	ASP3-1
Sip4p	16.67%	0.40%	1.63E-02	SRB4
Sef1p	16.67%	0.40%	1.70E-02	ASP3-1
Asg1p	16.67%	0.39%	1.78E-02	ASP3-1
Cad1p	33.33%	0.25%	2.15E-02	AVT2 RNR3
Adr1p	33.33%	0.24%	2.38E-02	RNR3 ASP3-1
Upc2p	16.67%	0.31%	2.64E-02	RNR3
Kar4p	33.33%	0.23%	2.65E-02	AVT2 RNR3
Uga3p	16.67%	0.31%	2.65E-02	RNR3
Yap5p	33.33%	0.23%	2.77E-02	RNR3 ASP3-1
Mth1p	16.67%	0.29%	3.11E-02	EXO1
Hap1p	16.67%	0.29%	3.12E-02	RNR3
Arg81p	16.67%	0.29%	3.16E-02	SRB4
Gis1p	16.67%	0.26%	3.90E-02	RNR3
Hap4p	33.33%	0.20%	3.93E-02	RNR3 EXO1
Rgm1p	16.67%	0.25%	3.97E-02	ASP3-1
Arg80p	16.67%	0.25%	3.97E-02	EXO1
Tos8p	16.67%	0.25%	4.20E-02	RNR3
Rim101p	50.00%	0.16%	4.31E-02	RNR3 ASP3-1 EXO1
Rme1p	16.67%	0.24%	4.37E-02	RNR3
YLR278C	16.67%	0.24%	4.53E-02	EXO1
Hcm1p	16.67%	0.23%	4.72E-02	SRB4
Cup9p	33.33%	0.18%	4.94E-02	SRB4 RNR3
Gat3p	16.67%	0.22%	4.97E-02	RNR3
Gcn4p	66.67%	0.13%	5.08E-02	RNR3 YKR051W ASP3-1 EXO1
Mig1p	16.67%	0.22%	5.09E-02	RNR3
Bas1p	66.67%	0.13%	5.14E-02	RNR3 YKR051W ASP3-1 EXO1
Rgt1p	16.67%	0.20%	6.07E-02	EXO1

Gln3p	33.33%	0.17%	6.21E-02	RNR3 ASP3-1
Sut1p	16.67%	0.18%	7.23E-02	RNR3
Met32p	16.67%	0.18%	7.23E-02	RNR3
Mot3p	16.67%	0.17%	8.17E-02	RNR3
Mss11p	16.67%	0.17%	8.59E-02	SRB4
Mal33p	16.67%	0.16%	9.46E-02	ASP3-1
Yox1p	33.33%	0.14%	9.92E-02	RNR3 YKR051W
Haa1p	16.67%	0.15%	1.02E-01	EXO1
Ino4p	33.33%	0.14%	1.05E-01	RNR3 YKR051W
Ume6p	33.33%	0.14%	1.07E-01	RNR3 ASP3-1
Yap7p	16.67%	0.14%	1.09E-01	RNR3
Mga1p	16.67%	0.14%	1.12E-01	ASP3-1
Rph1p	16.67%	0.14%	1.14E-01	ASP3-1
Rlm1p	16.67%	0.14%	1.14E-01	ASP3-1
Cbf1p	33.33%	0.13%	1.16E-01	ASP3-1 EXO1
Zap1p	33.33%	0.13%	1.19E-01	RNR3 ASP3-1
Hap2p	33.33%	0.12%	1.34E-01	ASP3-1 EXO1
Yrm1p	50.00%	0.12%	1.35E-01	AVT2 RNR3 YKR051W
Fkh1p	50.00%	0.11%	1.39E-01	AVT2 SRB4 RNR3
Mig3p	33.33%	0.12%	1.45E-01	AVT2 RNR3
Mcm1p	33.33%	0.12%	1.45E-01	RNR3 ASP3-1
Ixr1p	33.33%	0.12%	1.58E-01	AVT2 RNR3
Hsf1p	33.33%	0.12%	1.60E-01	AVT2 RNR3
Rox1p	16.67%	0.11%	1.70E-01	RNR3
Sko1p	16.67%	0.11%	1.77E-01	EXO1
Spt23p	33.33%	0.11%	1.79E-01	RNR3 YKR051W
Yap6p	16.67%	0.10%	1.93E-01	EXO1
Hms1p	16.67%	0.10%	1.99E-01	RNR3
Reb1p	16.67%	0.10%	2.06E-01	AVT2
Rap1p	50.00%	0.10%	2.09E-01	AVT2 RNR3 ASP3-1
Swi4p	16.67%	0.09%	2.23E-01	RNR3
Pho2p	16.67%	0.09%	2.25E-01	ASP3-1
Ash1p	50.00%	0.09%	2.50E-01	RNR3 YKR051W ASP3-1
Pdr3p	16.67%	0.08%	2.60E-01	ASP3-1
Gal4p	16.67%	0.08%	2.72E-01	RNR3
Tye7p	16.67%	0.08%	2.80E-01	YKR051W
Sok2p	33.33%	0.09%	2.87E-01	RNR3 YKR051W
Yhp1p	16.67%	0.08%	2.97E-01	RNR3
Pip2p	16.67%	0.08%	3.03E-01	RNR3
Xbp1p	16.67%	0.07%	3.38E-01	RNR3
Aft1p	16.67%	0.07%	3.38E-01	RNR3
Pdr1p	16.67%	0.07%	3.49E-01	AVT2
Ace2p	66.67%	0.08%	3.57E-01	AVT2 RNR3 YKR051W EXO1
Arr1p	16.67%	0.06%	4.05E-01	RNR3
Gcr1p	16.67%	0.06%	4.16E-01	EXO1
Cst6p	33.33%	0.07%	4.20E-01	ASP3-1 EXO1
Ste12p	50.00%	0.08%	4.22E-01	AVT2 RNR3 EXO1

Abf1p	33.33%	0.07%	4.45E-01	AVT2 YKR051W
Fkh2p	16.67%	0.05%	5.49E-01	RNR3
Sfp1p	50.00%	0.07%	5.61E-01	RNR3 YKR051W EXO1
Cin5p	16.67%	0.05%	5.92E-01	AVT2
Msn2p	33.33%	0.06%	6.16E-01	RNR3 ASP3-1
Yap1p	33.33%	0.06%	6.27E-01	RNR3 ASP3-1
Msn4p	16.67%	0.04%	6.99E-01	RNR3
Tec1p	33.33%	0.05%	7.20E-01	SRB4 EXO1
Tup1p	16.67%	0.04%	7.23E-01	RNR3

Subnetwork 11

Transcription Factor	% in user set	% in Yeastract	p-value	Target ORF/Genes
Crz1p	60.00%	0.47%	2.97E-04	YCR041W YDR134C TOH1
Aca1p	40.00%	0.70%	5.95E-04	YCR041W PER33
Cad1p	60.00%	0.38%	7.09E-04	YDR134C TOH1 VMA11
Hap4p	60.00%	0.30%	1.69E-03	YCR041W TOH1 VMA11
Gat3p	40.00%	0.45%	2.22E-03	YCR041W PER33
Cup9p	60.00%	0.28%	2.37E-03	YCR041W YDR134C PER33
Stb5p	60.00%	0.26%	3.06E-03	YCR041W YDR134C PER33
Gsm1p	20.00%	0.59%	5.32E-03	VMA11
Stp4p	20.00%	0.57%	5.76E-03	VMA11
Dal80p	20.00%	0.50%	7.47E-03	YCR041W
Cha4p	20.00%	0.46%	8.67E-03	YCR041W
Arr1p	60.00%	0.19%	1.04E-02	YCR041W YDR134C PER33
Gcr1p	60.00%	0.18%	1.12E-02	YDR134C PER33 VMA11
Sef1p	20.00%	0.40%	1.16E-02	VMA11
Asg1p	20.00%	0.39%	1.21E-02	VMA11
Mig3p	60.00%	0.18%	1.22E-02	YDR134C TOH1 PER33
Dal82p	40.00%	0.25%	1.24E-02	YDR134C VMA11
Rds1p	20.00%	0.38%	1.24E-02	YCR041W
Adr1p	40.00%	0.24%	1.30E-02	YDR134C PER33
Gzf3p	20.00%	0.35%	1.51E-02	YCR041W
Yap5p	40.00%	0.23%	1.52E-02	YCR041W VMA11
Hap3p	20.00%	0.33%	1.68E-02	YCR041W
Rim101p	60.00%	0.16%	1.81E-02	YDR134C TOH1 VMA11
Uga3p	20.00%	0.31%	1.82E-02	YCR041W
Yap3p	20.00%	0.30%	2.00E-02	YCR041W
Yap6p	40.00%	0.20%	2.06E-02	YCR041W TOH1
Flo8p	40.00%	0.20%	2.07E-02	YDR134C PER33
Msn2p	80.00%	0.12%	2.52E-02	YCR041W YDR134C TOH1 PER33
Gis1p	20.00%	0.26%	2.70E-02	TOH1
Oaf3p	20.00%	0.24%	3.07E-02	YDR134C
Hap5p	20.00%	0.22%	3.50E-02	YCR041W
Mac1p	20.00%	0.22%	3.72E-02	VMA11
Rgt1p	20.00%	0.20%	4.24E-02	PER33
Tec1p	80.00%	0.10%	4.39E-02	YCR041W YDR134C TOH1 VMA11
Pip2p	40.00%	0.15%	4.58E-02	YCR041W PER33
Ecm22p	20.00%	0.19%	4.77E-02	PER33
Ndt80p	20.00%	0.17%	5.45E-02	PER33
Cup2p	20.00%	0.17%	5.58E-02	VMA11
Pho4p	40.00%	0.14%	5.60E-02	YDR134C TOH1
Hac1p	20.00%	0.17%	6.03E-02	YDR134C
Sum1p	20.00%	0.16%	6.32E-02	PER33
Fkh1p	60.00%	0.11%	6.53E-02	YCR041W PER33 VMA11
Stp2p	20.00%	0.16%	6.77E-02	YCR041W

Tup1p	60.00%	0.11%	6.81E-02	YDR134C TOH1 PER33
Put3p	20.00%	0.15%	7.06E-02	YCR041W
Zap1p	40.00%	0.13%	7.07E-02	YCR041W TOH1
Ino2p	20.00%	0.15%	7.10E-02	TOH1
Haa1p	20.00%	0.15%	7.25E-02	YDR134C
Cst6p	60.00%	0.11%	7.46E-02	YDR134C PER33 VMA11
Mga1p	20.00%	0.14%	7.95E-02	YDR134C
Rfx1p	20.00%	0.14%	7.95E-02	YDR134C
Hap2p	40.00%	0.12%	8.00E-02	YDR134C VMA11
Rlm1p	20.00%	0.14%	8.13E-02	TOH1
Abf1p	60.00%	0.11%	8.30E-02	YDR134C TOH1 VMA11
Nrg1p	20.00%	0.14%	8.36E-02	YDR134C
Phd1p	20.00%	0.13%	9.20E-02	YDR134C
Hsf1p	40.00%	0.12%	9.70E-02	YDR134C PER33
Rap1p	60.00%	0.10%	1.04E-01	YDR134C TOH1 VMA11
Spt23p	40.00%	0.11%	1.10E-01	YDR134C TOH1
Oaf1p	20.00%	0.11%	1.21E-01	YDR134C
Hms1p	20.00%	0.10%	1.45E-01	YCR041W
Skn7p	20.00%	0.09%	1.62E-01	YCR041W
Swi4p	20.00%	0.09%	1.65E-01	YDR134C
Pho2p	20.00%	0.09%	1.66E-01	PER33
Cin5p	40.00%	0.09%	1.71E-01	TOH1 VMA11
Mga2p	20.00%	0.09%	1.75E-01	YDR134C
Sok2p	40.00%	0.09%	1.85E-01	YDR134C TOH1
Gal4p	20.00%	0.08%	2.04E-01	PER33
Tye7p	20.00%	0.08%	2.10E-01	VMA11
Fhl1p	20.00%	0.08%	2.12E-01	YDR134C
Aft1p	20.00%	0.07%	2.57E-01	TOH1
Xbp1p	20.00%	0.07%	2.57E-01	TOH1
Pdr1p	20.00%	0.07%	2.67E-01	YDR134C
Ino4p	20.00%	0.07%	2.73E-01	YDR134C
Cbf1p	20.00%	0.07%	2.91E-01	YDR134C
Met4p	20.00%	0.06%	2.98E-01	PER33
Mcm1p	20.00%	0.06%	3.34E-01	TOH1
Gcn4p	40.00%	0.07%	3.52E-01	YCR041W PER33
Bas1p	40.00%	0.07%	3.54E-01	TOH1 PER33
Rpn4p	20.00%	0.06%	3.68E-01	YDR134C
Ash1p	40.00%	0.06%	4.01E-01	PER33 VMA11
Fkh2p	20.00%	0.05%	4.43E-01	PER33
Yap1p	40.00%	0.06%	4.71E-01	YDR134C PER33
Msn4p	20.00%	0.04%	5.91E-01	YCR041W
Sfp1p	40.00%	0.05%	6.96E-01	YDR134C PER33
Ace2p	40.00%	0.04%	7.95E-01	YCR041W VMA11

Subnetwork 12

Transcription Factor	% in user set	% in Yeastract	p-value	Target ORF/Genes
Ume6p	80.00%	0.27%	3.72E-04	YIL015C-A KIN2 RPM2 YMR046C
Ixr1p	80.00%	0.23%	8.43E-04	MIG1 KIN2 RPM2 YMR046C
Mig1p	40.00%	0.44%	2.31E-03	MIG1 RPM2
Ert1p	20.00%	0.44%	9.63E-03	RPM2
Sef1p	20.00%	0.40%	1.16E-02	KIN2
Mig3p	60.00%	0.18%	1.22E-02	MIG1 YIL015C-A YMR046C
Bas1p	80.00%	0.13%	1.32E-02	MIG1 YIL015C-A RPM2 YMR046C
Hal9p	20.00%	0.37%	1.35E-02	RPM2
Yap5p	40.00%	0.23%	1.52E-02	MIG1 KIN2
Hap3p	20.00%	0.33%	1.68E-02	RPM2
Mig2p	20.00%	0.33%	1.69E-02	MIG1
Ash1p	80.00%	0.13%	1.78E-02	MIG1 YIL015C-A KIN2 RPM2
Rim101p	60.00%	0.16%	1.81E-02	MIG1 YIL015C-A RPM2
Nrg2p	20.00%	0.29%	2.14E-02	RPM2
Dal81p	20.00%	0.27%	2.40E-02	KIN2
Yap1p	80.00%	0.12%	2.67E-02	MIG1 YIL015C-A RPM2 YMR046C
Tos8p	20.00%	0.25%	2.91E-02	YIL015C-A
Hap5p	20.00%	0.22%	3.50E-02	RPM2
Rgt1p	20.00%	0.20%	4.24E-02	MIG1
Yhp1p	40.00%	0.16%	4.40E-02	RPM2 YMR046C
Pip2p	40.00%	0.15%	4.58E-02	KIN2 RPM2
Ste12p	80.00%	0.10%	4.59E-02	YIL015C-A KIN2 RPM2 YMR046C
Gcr2p	40.00%	0.15%	4.65E-02	MIG1 RPM2
Ecm22p	20.00%	0.19%	4.77E-02	YIL015C-A
Met32p	20.00%	0.18%	5.07E-02	RPM2
Rds2p	20.00%	0.18%	5.28E-02	RPM2
Ndt80p	20.00%	0.17%	5.45E-02	YMR046C
Xbp1p	40.00%	0.14%	5.57E-02	MIG1 YIL015C-A
Aft1p	40.00%	0.14%	5.57E-02	YIL015C-A RPM2
Cup2p	20.00%	0.17%	5.58E-02	KIN2
Yox1p	40.00%	0.14%	5.81E-02	RPM2 YMR046C
Mss11p	20.00%	0.17%	6.06E-02	KIN2
Yrm1p	60.00%	0.12%	6.30E-02	MIG1 YIL015C-A RPM2
Crz1p	20.00%	0.16%	6.68E-02	MIG1
Zap1p	40.00%	0.13%	7.07E-02	YIL015C-A YMR046C
Leu3p	20.00%	0.15%	7.19E-02	RPM2
Haa1p	20.00%	0.15%	7.25E-02	KIN2
Hap2p	40.00%	0.12%	8.00E-02	KIN2 RPM2
Nrg1p	20.00%	0.14%	8.36E-02	RPM2
Rpn4p	40.00%	0.11%	1.04E-01	MIG1 KIN2
Rox1p	20.00%	0.11%	1.23E-01	YIL015C-A
Ace2p	80.00%	0.08%	1.35E-01	MIG1 YIL015C-A KIN2 RPM2
Yap6p	20.00%	0.10%	1.41E-01	YIL015C-A

Hap4p	20.00%	0.10%	1.46E-01	RPM2
Reb1p	20.00%	0.10%	1.51E-01	YMR046C
Skn7p	20.00%	0.09%	1.62E-01	RPM2
Msn2p	60.00%	0.09%	1.62E-01	YIL015C-A RPM2 YMR046C
Pho2p	20.00%	0.09%	1.66E-01	YIL015C-A
Tye7p	20.00%	0.08%	2.10E-01	YMR046C
Yrr1p	20.00%	0.08%	2.23E-01	RPM2
Msn4p	40.00%	0.08%	2.51E-01	YIL015C-A YMR046C
Fkh1p	40.00%	0.08%	2.66E-01	MIG1 RPM2
Pdr1p	20.00%	0.07%	2.67E-01	RPM2
Tup1p	40.00%	0.08%	2.72E-01	RPM2 YMR046C
Ino4p	20.00%	0.07%	2.73E-01	MIG1
Met4p	20.00%	0.06%	2.98E-01	MIG1
Arr1p	20.00%	0.06%	3.14E-01	MIG1
Gcr1p	20.00%	0.06%	3.23E-01	YMR046C
Gcn4p	40.00%	0.07%	3.52E-01	RPM2 YMR046C
Rap1p	40.00%	0.07%	3.54E-01	KIN2 YMR046C
Hsf1p	20.00%	0.06%	3.54E-01	YMR046C
Spt23p	20.00%	0.05%	3.80E-01	YMR046C
Swi5p	20.00%	0.05%	4.15E-01	RPM2
Fkh2p	20.00%	0.05%	4.43E-01	RPM2
Cin5p	20.00%	0.05%	4.84E-01	RPM2
Sok2p	20.00%	0.04%	5.05E-01	RPM2
Tec1p	40.00%	0.05%	5.66E-01	RPM2 YMR046C
Abf1p	20.00%	0.04%	6.54E-01	KIN2
Sfp1p	40.00%	0.05%	6.96E-01	MIG1 RPM2
